# Supplementary material for: Developing a quality framework for community pharmacy: a systematic review of international literature
Source: BMJ Open. 2024 Feb 15;14(2):e079820. doi: 10.1136/bmjopen-2023-079820 (PMC10875530; doi:10.1136/bmjopen-2023-079820)
Supplement: Supplementary data [file bmjopen-2023-079820supp001.pdf]

Supplementary File 1 – Database searches

PubMed		
Search	Query	Items found
#1	"quality"[Title/Abstract]	1,287,702
#2	"healthcare quality"[Title/Abstract]	3,376
#3	"health care quality"[Title/Abstract]	3,827
#4	assessment, healthcare quality[MeSH Terms]	349,704
#5	"quality assurance"[Title/Abstract]	29,169
#6	"quality assessment"[Title/Abstract]	27,324
#7	"quality improvement"[Title/Abstract]	49,716
#8	"quality of health care"[Title/Abstract]	5,355
#9	"quality of healthcare"[Title/Abstract]	1,915
#10	#1 OR #2 OR #3 OR #4 OR #5 OR #6 OR #7 OR #8 OR #9	1,569,851
#11	"retail pharmac*"[Title/Abstract]	767
#12	"community pharmac*"[Title/Abstract]	8,652
#13	"community pharmacy services"[MeSH Terms]	5,564
#14	"pharmacies"[MeSH Terms]	9,347
#15	#11 OR #12 OR #13 OR #14	18,246
#16	#10 AND #15<br>from 2005 - 2022	**2,317**

Scopus		
Search	Query	Items found
#1	TITLE-ABS-KEY (quality)	4,526,623
#2	TITLE-ABS-KEY ("community pharmac*")	14,435
#3	TITLE-ABS-KEY ("retail pharmac*")	1,182
#4	#8 OR #9	15,432
#5	#1 AND #4<br>from 2005 – 2022	**2,327**

*Using TITLE-ABS-KEY “quality” captures: TITLE-ABS-KEY ("quality improvement") TITLE-ABS-KEY ("quality assessment") TITLE-ABS-KEY ("quality assurance") TITLE-ABS-KEY (quality W/3 care)

Embase		
Search	Query	Items found
#1	(quality adj5 care).ab,kw,ti.	148,342
#2	"health care quality".mp. or health care quality/	264,992
#3	"healthcare quality".mp.	4243
#4	"quality improvement".ab,kw,ti.	77715
#5	"quality assessment".ab,kw,ti.	34799
#6	"quality assurance".ab,kw,ti.	44810
#7	quality.ab,kw,ti.	175,1710
#8	#1 or #2 or #3 or #4 or #5 or #6 or #7	191,6548
#9	community pharmacy/ or "community pharmac*".mp.	23925
#10	"retail pharmac*".ab,kw,ti.	1422
#11	#9 OR #10	24978
#13	#8 AND #11<br>from 2005-2022	**3,631**

**CINAHL**		
Search	Query	Items found
#1	(MH "Quality of Care Research") OR (MH "Quality of Health Care") OR (MH "Quality Improvement") OR (MH "Quality Assessment") OR (MH "Quality Assurance") OR "quality"	603,463
#2	"community pharmac*"	6,041
#3	(MH "Pharmacy, Retail") OR ""retail pharmac*""	8,275
#4	#2 OR #3	11,488
#5	#1 AND #4<br>Publication year 2005-2022	**1,302**

- Using “quality” as a keyword captures (MH "Quality of Care Research") OR (MH "Quality of Health Care") OR (MH "Quality Improvement") OR (MH "Quality Assessment") OR (MH "Quality Assurance")

PsycINFO<br>Search	 Query	 Items found
#1	exp "Quality of Services"/ or quality.mp. or exp "Quality of Care"/	316648
#2	"community pharmac*".mp.	1245
#3	"retail pharmac*".mp. [mp=title, abstract, heading word, table of contents, key concepts, original title, tests & measures, mesh word]	143
#4	2 or 3	1366
#5	1 and 4<br>Specific range 2005-2022	**207**

- Mapping the search term “quality” to “quality of services” and “quality of care”, covers: (quality adj5 care) OR "quality assessment" OR "quality improvement" OR "quality assurance"

Web of Science<br>Search	 Query	 Items found
#1	quality (Topic)	3,205,809
#2	"community pharmac*" (Topic)	10,595
#3	"retail pharmac*" (Topic)	823
#4	#2 OR #3	11,310
#5	#1 AND #4<br>Publication years 2005-2022	**1,794**

- *Using “quality (Topic)” covers:* (quality) NEAR/3 (care or healthcare) (Topic) OR "quality assessment" (Topic) OR "quality improvement" (Topic) OR "quality assurance" (Topic)
