# Supplementary material for: Developing a quality framework for community pharmacy: a systematic review of international literature
Source: BMJ Open. 2024 Feb 15;14(2):e079820. doi: 10.1136/bmjopen-2023-079820 (PMC10875530; doi:10.1136/bmjopen-2023-079820)
Supplement: Supplementary data [file bmjopen-2023-079820supp002.pdf]

Author(s) & year*	Country	Purpose	Study design	Sample	Pharmacy service(s)	Key findings mapped under quality dimensions
Puumalainen et al. (2005)[102]	Finland	To develop a validated, easy-to-use patient counselling quality assurance instrument for community pharmacists.	Delphi	2 expert panels: Panel 1, consisting of experienced pharmacy practitioners (n = 10), and Panel 2, consisting of academic and professional experts (n = 10).	• Medicines supply	• **Person-centred care:** Patient experience: Indicators that enable practitioners to understand patients' specific information and communication needs.<br>• **Competence:** Competence in the dispensing process: indicators relevant to technical skills in dispensing prescriptions; incorporation of patient counselling in the dispensing process.<br>• **Environment:** Physical resources: availability and accessibility of information sources (manual, electronic); guidelines on patient counselling practices in the pharmacy; guidelines on patient counselling with local healthcare personnel
Worley (2006)[44]	USA	To test a pharmacist-patient relationship quality model in a group of older persons with diabetes from the	Survey	311 community-dwelling older persons (65 years of age and older), using at least one prescription medication and primarily	• Professional pharmacy service	• **Person-centred care** Patient-pharmacist relationship: contains measurement items from 5 study constructs -

		patient's perspective. Pharmacist-patient relationship commitment was the outcome of relationship quality studied.		obtaining their prescriptions from some type of nonmail order pharmacy		1. Pharmacist participative behaviour/patient-centeredness of relationship<br>2. Patient participative behaviour<br>3. Pharmacist-patient interpersonal communication<br>4. Relationship quality<br>5. Relationship commitment
Vilako et al. (2007)[81]	Estonia	To assess the preferences of pharmacy customers when choosing a pharmacy and their expectations of the service and comparing these with the opinions of pharmacists.	Survey	Patients (n=1979) in cities (in 3 community pharmacies), towns (in 2 community pharmacies), and in small towns (in 2 community pharmacies). The survey was also carried out among community pharmacists (n=135) in different regions of Estonia.	• Not specified	Survey items:<br>• **Access**<br>Physical access: Parking space near pharmacy; Comfortable entering to pharmacy<br>Availability of medicines: wide choice of products<br>Waiting time: Quick service<br>• **Environment**<br>Waiting area: Comfortable staying in pharmacy.<br>Private consultation area: privacy and discretion<br>• **Competence**<br>Clinical knowledge and diagnostic skills: Professional consulting;

						Help with choosing right medicine
James et al. (2008)[103]	UK	To develop explicit criteria against which the quality of Medicine Use Review (i.e. MUR; a planned patient-pharmacist consultation to discuss medicines use) referral documentation can be assessed.	Delphi	Sixteen panellists (these were 10 out of 14 MUR accreditation tutors who were invited to take part and six pharmacy practitioners from a possible 22 primary care organisations in Wales)	• Professional pharmacy service	• **Safety**<br>Documentation of care: consensus was achieved for 20 quality indicators that correspond with that of documenting a patient-pharmacist consultation.
Benrimoj et al. (2009)[116]	Australia	To implement nationally a quality improvement package in relation to the Standards of Practice for the Provision of Non-Prescription Medicines.	Randomly selected pharmacies were coached on the implementation of the Standards of Practice for the Provision of Non-Prescription Medicines. Pre and post measurements of the level of adherence to the Standards were taken.	2,706 pharmacies	• Medicines supply<br>• Professional pharmacy service (supply of OTC medicines following minor illness consultation)	Standards used in this study defined and described the professional activities required for the provision of medicines at a consistent and measurable level of practice.<br><br>**Environment:** Appearance of the pharmacy (3 statements); Physical resources (1 statement); Private consultation area (1 statement)<br><br>**Person-centred care:** Professionalism (3 statements)<br><br>**Competence:** Competence in dispensing process/clinical knowledge and diagnostic skills (8 statements)<br><br>**Safety:** Documentation of care (2 statements); Systems for

						ensuring safety and safety. (1 statement)
Rapport et al. (2009)[45]	UK	Identifying the extent to which pharmacy spaces are aligned to good professional practice, enhance a professional's sense of self and meet the demands of the public.	Mixed-methods approach employing biographic and photographic techniques	16 pharmacists	• Medicines supply<br>• Professional pharmacy service	• **Environment**<br>Dispensary: Essential to be well organised and to have control over the space and the way it functions. Barriers for dispensing to function in an orderly fashion: unwanted interruptions, undesired observation, lack of formality, lack of room and changes to the order and running of things brought about by others'<br><br>Private consultation area: Some have reservations about its size and positioning. In the smaller settings, particularly within Independent and dedicated pharmacies, consultation rooms are shoehorned into an already limited workspace, bringing additional pressure overflow storage.<br><br>Appearance of the pharmacy: Brings into question the pharmacist's position and professional status. The sales counter is particularly problematic when it comes to distractions with members of the public attempting to overcome

						the divide between pharmacist, dispensary and sales floor.
Sakurai et al. (2009)[82]	Japan	To investigate how pharmacy functions and services affect patient satisfaction	Survey	30186 Patients from 178 pharmacies whose purpose of use of the pharmacies was not only for prescription dispensing but also OTC medicines	• Medicines supply<br>• Sale of OTC medicines following minor illness consultation	Survey items:<br>• **Access:**<br>Opening hours: opening hours<br>Physical access: location<br>Waiting time: average waiting time; maximum waiting time<br>Availability of medicines: amount of pharmaceutical stock<br>• **Environment:**<br>Private consultation area: Privacy considerations<br>Waiting area: number of waiting chairs<br>Physical resources: number of blood pressure, bone density and other measuring instruments
Feletto et al. (2010)[49]	Australia	To determine the needs of pharmacies that were important and the elements requiring improvement when implementing and delivering cognitive pharmaceutical services.	Survey	355 community pharmacies	• Professional pharmacy service	Survey items covered the following areas:<br>• **Access:** opening hours (1)<br>• **Environment:** Private consultation area (3); appearance of the pharmacy (2)<br>• **Competence:** training of pharmacy staff (2)<br>• **Integration** (1)

Harding et al. (2010)[114]	UK	To explore existing mechanism to ensure quality assurance of medicine use reviews (MURs), and to identify those parameters of an MUR that community pharmacists consider as indicators of quality.	Survey	50 pharmacists, a third of which were from locum pharmacists.	• Professional pharmacy service	Individual survey items were not provided but the analysis of findings were mapped on the quality dimensions:<br>• **Competence:** Clinical knowledge and diagnostic skills: The single most frequently reported determinant for undertaking an MUR was the pharmacists' judgement (84% n = 42). Over 70% (n = 35) of respondents considered that undertaking MURs required specialist skills
Scahill et al. (2010)[46]	New Zeeland	To develop a multi-constituent model of organizational effectiveness for community pharmacy.	Face to face brainstorming to generate statements describing what constitutes an effective community pharmacy, and sorting of the statements into themes with rating of each statement for importance	14 stakeholders representing policy-makers and health care providers including; community pharmacy, professional pharmacy organizations, primary health care funders and policy-makers, general practitioners and general practice support organizations	• Medicines supply<br>• Professional pharmacy service	• **Safety:** statements addressed how an organisation could promote safe and effective workflow; and ensure safe use of medicines.<br>• **Integration:** statements addressed how an organisation could focus on patient needs; and better integrate within primary care.

Snyder et al (2010)[75]	USA	To describe the professional exchanges that occurred between community pharmacists and physicians engaged in successful collaborative working relationships (CWRs), using a published conceptual model and tool for quantifying the extent of collaboration	Semi structured interviews, and completion of the Pharmacist-Physician Collaborative Index	Five pairs of community pharmacists and physician colleagues	• n/a	• **Integration**<br><br>*Interprofessional collaboration*: Pharmacists were the primary initiator of these CWRs. Initial conversations were usually (but not always) conducted face-to-face and often scheduled in advance by the pharmacist.<br><br>Establishing trust was the provision of high-quality recommendations that improved patient outcomes. Both professionals commented on how seeing these positive outcomes was key to the success of their relationship.<br><br>Resistance manifested passively, as lack of physician response to recommendations, and actively, as refusal to provide patient laboratory data in spite of signed medical releases and hesitations to provide referrals for clinical services beyond patient education.
Trap et al. (2010)[104]	Ethiopia, Uganda and Zimbabwe.	To develop an indicator-based tool for systematic assessment and reporting of good pharmacy practice (GPP).	direct observations, record reviews, interviews and simulated clients in surveyed facilities		• Medicines supply<br><br>• Professional pharmacy service	Indicators developed focusing on:<br><br>• **Safety**<br>Documentation of care (5 indicators); Dispensing (14 indicators)<br><br>• **Access**

						Availability of medicines (1 indicator)<br><br>• **Environment**<br>Appearance of the pharmacy (7 indicators); physical resources (1 indicator)<br><br>• **Competence**<br>Competence in the dispensing process (4 indicators), Clinical knowledge and diagnostic skills (2 indicators)
Urbonas et al. (2010)[47]	Lithuania	To analyse pharmacy specialists' attitudes toward the quality of pharmaceutical services at Lithuanian community pharmacies.	Survey	471 Lithuanian community pharmacy specialists	• Medicines supply	Survey covered two quality dimensions:<br>• **Competence**<br>Competency in the dispensing process (5 indicators covering side effects, time spent with patient, information about drug therapy and healthy lifestyle)<br><br>• **Person-centred care**<br>Patient experience (5 indicators covering consideration of financial capabilities, patient needs, helpfulness to each patient, willingness to get patient to come back to pharmacy)
White et al. (2010)[48]	Australia	To investigate the views of a range of stakeholders regarding the effectiveness of	In-depth interviews	20 in-depth interviews were conducted with various stakeholders, including community	• Medicines supply	• **Environment:**<br>Appearance of the pharmacy: Maximizing the visibility of health ranges

		service quality as a differentiating position for community pharmacy.		pharmacists, managers of pharmacy groups, and industry advisers	• Professional pharmacy service	• **Person-centred care:** Patient experience: Greeting customers by name<br><br>• **Competence:** Competence in the dispensing process: sitting with customer to discuss prescription and health needs<br><br>Clinical knowledge and diagnostic skills: potential for community pharmacy to become more service orientated by offering home medication reviews, screening program and disease state management.
De Bie et al. (2011)[105]	Netherlands	To develop a national system of quality indicators for community pharmacy care, reported by community pharmacies.	Delphi	14 pharmacy practice experts and 76 practising pharmacists	• Medicines supply	Indicators focused on:<br>• **Competence** Competence in the dispensing process (7 indicators)<br><br>• **Safety** Systems for ensuring safety quality (15 indicators); compounding (7 indicators); dispensing (13 indictors)
Horvat et al. (2011)[83]	Slovenia	To identify content of pharmacy performance	Interviews + Delphi	Phase 1: interviews with 43 pharmacy users were conducted to identify	• Medicines supply	• **Person-centred care:**

		relevant to patient satisfaction.		patients' experiences and expectations relating to pharmacies.<br><br>Phase 2: a 10 member expert panel was employed in a two round Delphi technique to rate the importance of each item for the patient satisfaction.		Patient-pharmacist relationship (1 item); Professionalism (17 items)<br><br>• **Competence:**<br>Competence in the dispensing process (18 items)<br><br>• **Access:**<br>Waiting time (1); Opening hours (2); Availability of medicine (3); Physical access (3); availability of pharmacist (1)<br><br>• **Environment**: private consultation area (1); waiting area (2); appearance of the pharmacy (10); appearance of the pharmacy (4)
AHRQ (2012)[50]	USA	The Agency for Healthcare Research and Quality (AHRQ) funded the development of the Community Pharmacy Survey on Patient Safety Culture. This survey is designed specifically for community pharmacy providers and other staff and asks for their opinions about the	Survey	Details on development of survey not provided	Not specified	The survey includes 36 items that measure 11 composites of<br><br>• Safety culture:<br>Physical space and environment (3 items);<br>Teamwork (3 items);<br>Staff training and skills (4 items);<br>Communication openness (3 items);<br>Patient counselling (3 items);<br>Staffing, work pressure and pace (4 items);

		culture of patient safety in their pharmacy.				Communication about prescriptions across shifts (3 items);<br>Communication about mistakes (3 items);<br>Response to mistakes (4 items)<br>Organizational learning (3 items);<br>Overall perceptions of patient safety (3 items)
Halsall et al. (2012)[27]	UK	To develop a conceptual framework characterizing healthcare quality in the community pharmacy setting.	Focus groups	10 focus group discussions with 47 participants (patients and their carers, pharmacists and pharmacy staff, and NHS staff who commissioned pharmacy services) were conducted across the northwest of England, United Kingdom.	• Medicines supply	• **Access**<br>Availability of medicines: Patient awareness of available medicines<br>Physical access: whether patients could physically access care.<br><br>• **Environment**<br>Physical resources: Pharmacy personnel having access to adequate structures to provide care and that these should be continually reviewed.<br><br>• **Competence:**<br>Competence in the dispensing process: Supplying medicines appropriately and providing individualized advice to patients.<br><br>• **Person-centred care:**<br>Patient experience: Ensuring patients/carers at the point of care have a positive perception of the experience

Dadfar et al. (2012)[51]	Iran	To assess the quality of Tehran pharmacies' services and their impacts on the pharmaceutical firms, and suggest some improvements	Survey + in-depth interviews	127 pharmacy users completed the questionnaire.<br><br>32 interviews with Pharmacists (n=10),<br>Pharmaceutical managers<br>(n=8);<br>patient<br>(n=9)<br>Physician<br>(n=4);<br>MOH authority (n=1)	• Medicines supply	Survey covered the following dimensions of quality:<br>• **Competence:** Competence in the dispensing process (5 items)<br>• **Person-centred care:** Professionalism (5 items)<br>• **Environment:** Physical resources (1 item); appearance of the pharmacy (1 item); appearance of the pharmacy (1 item)<br>• **Access:** Opening hours (1 item); waiting time (1 item)
White et al. (2012)[52]	Australia	To explore the perceptions of pharmacy staff regarding the factors that constitute a high level of service quality using the service quality determinants proposed by the Conceptual Model of Service Quality	Structured interviews	27 pharmacy assistants and 6 pharmacists in 3 community pharmacies in Sydney.	Not specified	• **Safety:** All the participants acknowledged the existence of some form of internal quality control programs, but provided inconsistent answers and uncertainty regarding frequency, process, and content of such programs, within and across the pharmacies
Phipps et al. (2012)[53]	UK	To evaluate the internal reliability, factor structure and construct validity of the Pharmacy	Survey	A total of 4105 members of the community pharmacy workforce, all drawn from one of the	Not specified	24 items emerged relative to<br>• **Safety**

		Safety Climate Questionnaire (PSCQ) when applied to a pan-European sample of community pharmacies.		five participating countries (Denmark, Germany, the Netherlands, Portugal and Great Britain)		Safety culture: Organizational learning (13 items); Blame culture (4 items); Working conditions (4 items); Safety focus (3 items).
Rubio-Valera et al (2012)[71]	Spain	To identify and analyse factors affecting GP-CP collaboration.	Semi-structured interviews	18 GPs, 19 community pharmacists	n/a	• **Integration**<br>Communication mechanisms and information systems: 1) having a coordinator and 2) sharing a clinical chart to have access to patient information
Kelly et al. (2013)[76]	Canada	To capture the opinions of family physicians and community pharmacists in Newfoundland and Labrador (NL) regarding collaborative practice.	Survey	407 pharmacists & 462 family doctors	n/a	• **Integration**<br>Communication mechanisms and information systems: Pharmacists preferred telephone or face to-face communication over paper correspondence with GPs. GPs preferred telephone communication. Pharmacists believed that electronic transfer of information should be explored.<br><br>Interprofessional collaboration: GPs believed that the most important pharmacist functions were to help improve patient adherence and fill prescriptions. Pharmacists would like to participate more in decisions regarding identification and management of drug-related problems—managing drug interactions, providing drug

						information to inform decisions around patient drug therapy and assisting to modify drug therapy to resolve patient-specific problems.<br><br><u>Incentivisation:</u> Lack of compensation and the need to collaborate with multiple GPs/pharmacists to provide care for patients were viewed as the 2 most significant barriers.
Patterson et al. (2013)[84]	USA	To describe and identify significant relationships among pharmacy service use, general and service-specific patient satisfaction, pharmacy patronage motives, and marketing awareness in a service-oriented, independent community pharmacy	Survey	241 patients	• Medicines supply	Survey items coverage:<br>• **Access:** Waiting time (2 items); Availability of pharmacist (1 item)<br>• **Patient-experience:** Professionalism (6 items); patient experience (2 item); Patient-pharmacist relationship (1 item)<br>• **Competence:** Competence in the dispensing process (7 items)<br>• **Environment:** Appearance of the pharmacy (1 item); private consultation area (1 item)
Merks et al. (2014)[85]	Poland	To compare factors that influence a patient's choice of pharmacy in Poland and in the UK, to identify which of them are components of pharmaceutical care, and to relate them to	Survey	417 patients from 36 pharmacies in Poland and 405 patients from 56 pharmacies in the UK.	• Not specified	• **Access**<br><br><u>Physical access:</u> The convenient location of pharmacy was one of the most frequently reported factors by Polish and British respondents.<br><br>• **Person-centred care**

		patient loyalty to the same pharmacy				Professionalism: professional service was one of the most frequently reported factors by Polish and British respondents. British respondents were more likely than the Polish to choose a pharmacy because of a professional service.<br><br>• **Environment**<br><br>Private consultation area: British respondents were more likely than the Polish to choose a pharmacy because a possibility to discuss their health problems in a separate consultation room<br>Appearance of the pharmacy: Polish respondents were more likely than the British to base their choice of pharmacy on the aesthetic decoration of the pharmacy. Aesthetic decoration of the pharmacy was more important to respondents who often visited a pharmacy (one to two times a week) than to those who visited a pharmacy less frequently.<br><br>• **Competence**<br>Clinical knowledge and diagnostic skills: Good advice received in a pharmacy was one of the most frequently reported factors by the British respondents. British respondents were more likely

						than the Polish to choose a pharmacy because of a possibility to receive good advice.
McMilan et al. (2014)[86]	Australia	To explore the attributes of pharmacy choice for people with chronic conditions.	Semi-structured interviews	97 interviews (patients=70, carer n=8, patient/carer n=19)	• Not specified	• **Person-centred care**<br><br>Patient experience: Taking the time to ensure that the person's individual needs were met and not identifying people solely by their condition(s) were exemplars of caring pharmacy staff. The provision of information was viewed positively by participants and deemed essential when obtaining a new medication. Some consumers from culturally diverse backgrounds sought a pharmacy where a staff member spoke the same language or had the same cultural background<br><br>Patient-pharmacist relationship: Continuity of care was another reason for utilising a regular pharmacy, as this facilitated awareness of the person's medical history. For others, medication safety was a key priority for them and hence, using a regular pharmacy was seen as a way to optimise this via continuity of care.<br><br>Professionalism: Staff approachability facilitated a

						relaxed environment for consumers to ask questions and seek advice, thus supporting patient empowerment and resulting in continued use of that pharmacy.<br><br>• **Access**<br>Physical access: The majority of participants selected a conveniently located pharmacy, e.g. close to their home or doctor, to use regularly, in order to reduce the time accessing care.
Mehralian et al. (2014)[87]	Iran	To assess pharmacy customers' priorities and satisfaction with community pharmacy services in Tehran	Survey	800 pharmacy customers of 200 community pharmacies in 22 districts of Tehran	• Medicines supply<br><br>• Professional pharmacy service (Sale of D medicines following minor illness consultation)	Survey items looked at:<br>• **Person-centred care:** professionalism (2)<br>• **Competence:** competency in the dispensing process (3); Clinical knowledge and diagnostic skills (5)<br>• **Access:** Availability of medicines (3), waiting time (1)<br>• **Environment:** appearance of the pharmacy (1), waiting area (1), private consultation area (1)
Odukoya et al. (2014)[54]	USA	To examine factors influencing quality of patient interaction at community pharmacy	Non-participant observation (quantitative approach)	22 community pharmacies	• Medicines supply	• **Access:**<br><br>Physical access: The key enabling variables affecting amount of time pharmacists spent with

		drive-through and walk-in counselling areas				patients were location of interaction (drive-through or walk-in) and level of pharmacy busyness. Pharmacists spent less time with patients at the drive-through compared to the walk-in counselling area.
Chen et al. (2015)[88]	China	Examines the impact of service quality and the mediating effects of customer satisfaction and customer loyalty on willingness to pay more.	Survey	479 retail pharmacy users in China	• Not specified	Survey items looked at:<br>• **Environment**<br>Appearance of the pharmacy (2 items)<br>• **Access**<br>Physical location (2 items); Availability of medicines (3 items); Waiting times 3 items); Opening hours (1 item)<br>• **Person-centred care**<br>Professionalism (3 items)<br>• **Safety**<br>Documentation of care (1 item)
Hattingh et al. (2015)[55]	Australia	To explore the unique privacy and confidentiality requirements of mental health consumers and carers in the Australian community pharmacy context	In-depth interviews and focus groups	There were 98 participants consisting of consumers and carers (n = 74), health professionals (n = 13) and representatives from consumer organisations (n = 11).	• Professional pharmacy service	• **Environment:**<br>Private consultation area: Consumers and carers expressed concerns that their anonymity and right to receiving sensitive information were breached when other customers were present in the pharmacy.

						Due to the highly accessible nature of community pharmacy services and services being provided in a public space, there is a fear of being recognised by colleagues, friends and neighbours when collecting medication<br><br>The use of a private consultation room or area was seen as a main facilitator for overcoming privacy and confidentiality issues during pharmacy interactions.
Schoenmakers et al. (2015)[106]	Netherlands	To assess the validity of 52 quality indicators (QI) for community pharmacies using the Indicator Assessment Framework (IAF)	An expert panel applied the IAF criteria to the set of QIs collected in 1,807 Dutch community pharmacies on their performance in 2011.	Expert panel consisted of 6 pharmacists from urban as well as rural areas and from different settings, such as independent pharmacies, pharmacies in pharmacy chains, or pharmacies in health centres	• Medicines supply	Indicators focused on the following domains:<br><br>• **Competence**<br>Competence in the dispensing process (3 indicators)<br>Training of pharmacy staff (1 indicator)<br><br>• **Safety**<br>Systems for ensuring safety (21 indicators); Compounding (3 indicators); dispensing (21 indicators); documentation of care (2)<br>• **Integration** (1 indicator)

Alhomoud et al. (2016)[89]	UAE	Assessed patients' experiences and satisfaction with community pharmacy services in the UAE, which can be used as an indicator to improve services.	Survey	415 patients	• Medicines supply.	Questionnaire comprised of Items on:<br>• **Person-centred care:** Professionalism (4); patient experience (2)<br>• **Competence:** competency in the dispensing process (5)<br>• **Access:** Availability of pharmacy staff (1)
Arkaravichien et al. (2016)[108]	Thailand	Test a quality indicators tool for feasibility by applying it in two pharmacy settings; accredited independent community pharmacies and accredited chain community pharmacies,	Observation and interviewing pharmacist in charge	60 pharmacies enrolled in the study of which 34 were independent pharmacies and 26 chain pharmacies	• Medicines supply	The tool comprised of indicators covering:<br>• **Environment:** appearance of the pharmacy (1 indicator); appearance of the pharmacy (2 indicators); dispensary (3 indicators); physical resources (1 indicator)<br>• **Access:** availability of pharmacy staff (2 indicators), availability of medicines (7 indicators)<br>• **Competence:** Competence in the dispensing process (18 indicators)
Grey et al. (2016)[103]	UK	To ask key stakeholders to confirm, and rank the importance of, a set of characteristics of good pharmaceutical service provision	Delphi	22 participants (DPs, CPs and patients/lay member)	• Medicines supply<br>• Professional pharmacy service	A set of 23 characteristics for providing good pharmaceutical services in CPs and DPs was developed:<br>• **Safety:** medicine supply (6)<br>• **Person-centred care**: patient experience (6)<br>• **Environment**: Appearance of the pharmacy (1); waiting area (1)

						• **Competence:** Clinical knowledge and diagnostic skills (2)<br>• **Integration:** Interprofessional collaboration (3)
Hashemian et al. (2016)[79]	Iran	To investigate the collaborative working relationship between pharmacists and GPs in terms of their attitudes, role perceptions, experience with collaborative practice, preferred method of communication, areas of current and further collaboration, and perceived barriers to interprofessional collaboration in a sample of the Iranian population	Survey	132 pharmacists and 99 general practitioners	• n/a	• **Integration**<br>Communication mechanisms and information systems: The preferred method of communication for collaborative practice for both groups was by telephone or face to face rather than by letter.<br>Interprofessional collaboration: Both groups reported that the most frequent collaboration is to "manage drug interactions", "provide patient counselling" and "manage side-effects of medications". Both groups were willing to collaborate in decision making on patients' pharmacotherapy and management of drug interactions.<br><br>Information sharing and access: Neither group was overly concerned about "liability for shared information", "lack of compensation" or "dealing with multiple care professionals" as barriers to collaboration

Nilugal et al. (2016)[90]	Malaysia	To investigate patient's attitudes, and satisfaction towards community pharmacist's role in Selangor, Malaysia	Survey	180 patients at three different community pharmacies in three different regions of Selangor state	• Medicines supply.<br>• Professional pharmacy service	Questionnaire items covered the following:<br>• **Competence:** Competency in dispensing process (7); clinical knowledge and diagnostic skills (7)<br>• **Access:** waiting time (3)<br>• **Person-centred care:** patient experience (2); professionalism (2)
Shiyanbola et al. (2016)[56]	USA	To describe older adults' perception of a quality pharmacy including their expectations of a quality pharmacy and their preferences in a quality pharmacy.	Focus groups	Six focus groups (60 patients) held in community centres and senior residence facilities in Wisconsin	• Medicines supply	• **Access:** Opening hours; availability of pharmacy staff; physical access<br>• **Person-centred care:** Patient-pharmacist relationship: Interpersonal relationship with pharmacist/pharmacy staff; familiarity with pharmacy/pharmacist staff;<br>Professionalism: friendliness and helpfulness of staff; pharmacist courtesy<br>• **Competence:** Competence in the dispensing process: ensuring medication safety; facilitates medication adherence; readily available to clarify questions

Teichert et al. (2016)[107]	Netherlands	To present a comprehensive quality indicator set for community pharmacies and to report the scores for these indicators as supplied by the majority of Dutch community pharmacies	Community pharmacists in the Netherlands were invited in 2013 to provide information for the set of 2012.Quality indicators were mapped by categories relevant for pharmaceutical care and defined for structures, processes and dispensing outcomes	Information was provided by 1739 of the 1981 Dutch community pharmacies (88 %)	• Medicines supply	Indicators focused on the following domains:<br>• **Competence**<br>Competence in the dispensing process (27 indicators); Training of pharmacy staff (5 indicators)<br>• **Safety**<br>Systems for ensuring safety quality (24 indicators); Compounding (4 indicators); medicine supply (3 indicators); documentation of care (1 indicator)<br>• **Integration** (3 indicators)
Weiss et al (2016)[57]	UK	To investigate the similarities and differences in how pharmaceutical services are provided by community pharmacies (CPs) and dispensing doctor practices (DPs) and (b) to identify the issues relevant to determining the quality of pharmaceutical services in these settings.	Mixed methods: A postal questionnaire of DPs and CPs. A subsection<br>of questionnaire respondent sites were selected<br>d to take part in case studies, which involved<br>documentary analyses, observation and staff interviews	Questionnaire: 52 CPs, 31 DPs<br>There were three CP and four DP case study sites, with 17 staff interviews	• Medicines supply	• **Person-centred care:**<br>Patient experience - providers' underlying values and commitment to providing patient-centred care. At the supermarket pharmacy, for example, staff would always strive to fulfil a patient's needs as they saw this as not only good for business but also their duty as a service provider.<br>• **Safety**<br>Medicine supply: Effective systems of work in relation to the checking of prescribed items.<br>Systems for ensuring safety: Effective systems of work in

						relation to the way in which dispensing errors were managed.
Koster et al. (2016)[58]	Netherlands	To provide insight into the agreement about quality of pharmaceutical care, measured both by a patient questionnaire and video observations	Pharmaceutical encounters in four pharmacies were video-recorded. Patients completed a questionnaire based upon the Consumer Quality Index. An observation protocol was used to code the recorded encounters. Agreement between video observation and patients' experiences was calculated.	109 encounters were included for analysis	• Medicines supply	• **Competence:** Competence in the dispensing process: Information provision (3 items); Medication counselling (4 items)<br>• **Person-centred care:** Professionalism: Pharmacy staff's communication style (8 items)
Feehan et al. (2017)[91]	USA	To gauge patient preferences explicitly for primary healthcare services that could be delivered through community pharmacy set-tings in the USA	Questionnaire (Discrete Choice Experiment)	10006 adults who had to have a minimal repeat use of a pharmacy for health care needs—defined as filled at least three or more prescriptions for	• Medicines supply.<br>• Professional pharmacy service	Attributes covered:<br>• **Access** Opening hours: Hours of operation Availability of medicines: Prescription ordering, availability and information

				themselves, at a pharmacy in the past 12months		Waiting time: Service logistics (I.e. walk in vs appointment)<br><br>• **Integration:** pharmacy has access to and can enter prescriptions and health information into your (the patient's) electronic medical record<br><br>• **Competence:**<br><br>Clinical and diagnostic skills: Physical examinations; Diagnostic testing; Preventive services; prescribing; Medication review services
Júnior et al. (2017)[110]	Brazil	To characterize the profiles and activities of community pharmacists, as well as the quality indicators of private community pharmacies in Paraná State - Brazil	Survey	533 pharmacists in Paraná State - Brazil	• Medicines supply<br><br>• Professional pharmacy service	• Five indicators relative to **environment:** Waiting area (1); Private consultation area (1); Physical resources (3).
Löffler et al. (2017)[78]	Germany	Investigating pharmacists' and general practitioners' views on barriers to interprofessional collaboration in the German health care system.	Interviews and focus groups	Six pharmacists were interviewed and four pharmacists took part in the focus group discussion.<br><br>Seven GPs were interviewed and eight	• n/a	• **Integration**<br>Communication mechanisms and information systems: The majority of pharmacists stated to encounter recurring difficulties getting GPs on the phone and receiving an answer to their query.<br>Interprofessional collaboration: GPs felt challenged in means of treating patients under time

				GPs participated in the focus group discussions.		constraints and avoiding or limiting polypharmacy. Most physicians perceived that community pharmacists were not able to respond to this challenge. GPs felt that pharmacists don't have background information on patients' medical history and/or professional knowledge to understand and reconstruct physicians' reasoning in many cases.<br>Proximity: Pharmacists employed in rural and provincial regions often experienced long-lasting working relationships to local GPs that were mostly characterized by mutual trust and appreciation. In contrast, in cities interprofessional collaboration was constrained by urban anonymity: Quite often, pharmacists hardly knew the GPs they tried to contact.<br>Incentivisation: Some GPs thought that pharmacists would have their own agenda trying to profit from patients with long-term conditions
Aziz et al. (2018)[92]	Pakistan	To assess pharmacies services with regard to patient's need	Survey	1088 patients of 544 community pharmacies	• Medicines supply.	41 Items on satisfaction covering the following dimensions:

						• **Access:** Physical access (3); opening hours (2); availability of medicines (3); waiting time (1)<br>• **Environment:** appearance of the pharmacy (1), waiting area (1), private consultation area (2); appearance of the pharmacy (1)<br>• **Person-centred care:** professionalism (2); patient experience (1)<br>• **Competence:** competency in the dispensing process (19)
Jacobs et al. (2018)[15]	UK	To explore stakeholder perceptions of the organisational and extra-organisational factors associated with service quality and quantity in community pharmacy as an established exemplar of private sector organisations providing publicly-funded healthcare.	Semi-structured interviews	Forty semi-structured interviews were conducted with service commissioners, superintendent and front-line pharmacists, purposively selected from across nine geographical areas and a range of community pharmacy organisational types in England.	• Medicines supply<br>• Professional pharmacy service	• **Competence:** Competence in the dispensing process: For dispensing, speed and accuracy were the most commonly cited elements of service quality. However, for pharmacists themselves, and for many service commissioners, accuracy was paramount.<br>Clinical knowledge and diagnostic skills: clinical aspects were considered by a number of pharmacists and commissioners to be an important element of quality either through counselling or the clinical check.<br>• **Integration**

						Interprofessional collaboration: Positive relationships between community pharmacies and local GP surgeries were seen to help nurture interdisciplinary practice, foster closer working around patients, increase effective signposting and improving communication.
Newlands et al. (2018)[59]	UK	To systematically identify and prioritise community pharmacy services in Scotland which required improvement and/or guideline development	A modified nominal group technique (NGT) was used for topic generation followed by an electronic Delphi survey	Stakeholder group comprising community pharmacists, policy makers, lay and pharmacy organisation representatives.	• Medicines supply<br>• Professional pharmacy service	Consensus reached on guideline development for:<br>• **Competence:**<br>Clinical knowledge and diagnostic skills: promoting the appropriate sale and supply of over-the-counter medicines; promotion and delivery of a Minor Ailment Scheme.<br>Competence in the dispensing process: Patient counselling for prescribed medication; evidence-based strategies to promote medication adherence; enhancing medication use for vulnerable patients (including high risk, sheltered housing residents, immigrants, homeless)<br>•

Tran et al. (2018)[99]	Vietnam	To determine the pattern of pharmacy customers' viewpoints regarding their satisfaction with the quality of services of community pharmacies in Vietnam	Q-methodology	144 pharmacy customers from 40 pharmacies in four Vietnamese cities.	• Medicines supply.	Statements covered the following dimensions:<br>• **Competence:** Competency in the dispensing process (22)<br>• **Environment:** Appearance of the pharmacy (1); waiting area (1); private consultation area (1); appearance of the pharmacy (1); dispensing (1)<br>• **Access:** opening hours (3); availability of medicines (2); physical access (1); availability of pharmacist (1)<br>• **Person-centred care:** professionalism (3)
Watson et al. (2018)[29]	UK	Exploration of service providers' attitudes and beliefs of quality and quality improvement in the community pharmacy setting in the UK.	Semi-structured interviews and focus groups	42 service providers. Four focus group discussions were undertaken with 38 pharmacists/pharmacy support staff and semi-structured interviews with four key informants from pharmacy organisations across the UK	• Medicines supply<br>• Professional pharmacy service(Supply of OTC medicines following minor illness consultation)	Quality was described in terms of:<br>• **Person-centred care:** Professionalism: showing empathy Patient-pharmacist relationship: developing rapport. The issue of good continuity of staff was also identified as being associated with better quality because it was believed to be important for developing trust and rapport between patients and pharmacy personnel.<br>• **Competence** Clinical knowledge and diagnostic skills: eliciting specific information during consultations; providing the right

						information/advice; prompt resolution of symptoms<br>• **Environment**<br>Private consultation area: The physical environment of pharmacies such as having a counter was also identified as a potential barrier to asking questions.
Fujita et al. (2019)[60]	Japan	To establish the quality dimensions of home pharmaceutical care (HPC) from the perspectives of home healthcare professionals	Semi-structured interviews and focus groups	Semi-structured interviews and focus groups were carried out with nine home healthcare teams, comprising 61 multidisciplinary professionals including pharmacists, doctors, nurses, care managers, home helpers, medical social workers and other relevant stakeholders involved in home healthcare.	• Professional pharmacy service	• **Environment:** physical resources<br>• **Competence:**<br>*Clinical knowledge and diagnostic skills:* pharmacist factors (professionalism, effectiveness, experience); during home pharmaceutical care (provision of medication review; frequency of visiting home; time spent at home)<br>Impact on patients (humanistic outcomes; clinical outcomes, economic outcomes); impact on other healthcare professional (task shifting, operational efficiency); recognition of benefits of home pharmaceutical care.<br>• **Integration:**

						Communication mechanisms and information systems: Before home pharmaceutical care (attendance at meetings; collaborative visiting schedule arrangements); after home pharmaceutical care (information sharing, timeliness)
Guhl et al (2019)[61]	Germany	Examines the value created by community pharmacies-defined as perceived customer value-in the prescription drug market through varying elements of service quality.	Survey	289 pharmacy users	• Medicines supply	Dimensions covered in the survey: • **Environment:** physical resources (1 item); appearance of the pharmacy (2 items), cleanliness & hygiene (1 item) • **Person-centred care:** professionalism (4 items); • **Access:** Waiting time (1 item); availability of medicines (2 items), opening hours (1 item); physical access (3 items) • **Competence:** Competence in the dispensing process (4 items) • **Safety:** medicine supply (1 item)
Halit et al. (2019)[115]	Lebanon	developing good pharmacy practice(GPP) guidelines to be applied by community pharmacists for	In January 2018, the OPL Scientific Committee decided to elaborate GPP guidelines for		• Medicines supply • Professional pharmacy service (sale of	The GPP standards comprised of sections that addressed the following dimensions: • **Environment:**

		services' quality improvement	community pharmacists and created the Community Pharmacy Practice Subcommittee, which was in charge of this project. To create the Lebanese GPP guidelines, the committee relied on the guidelines already implemented in several countries and tailored them to the Lebanese situation		OTC medicines following minor illness consultation)	Appearance of the pharmacy; appearance of the pharmacy; Private consultation area; dispensary.<br><br>Physical resources (availability of a refrigerator and other equipment, equipment status, routine maintenance, Availability of drug information systems; availability of medical devices and complementary medicines)<br><br>• **Access**<br>Availability of medicines: purchasing, storage, and maintenance of quality<br><br>• **Safety:**<br>Compounding; operating procedures, documentation, and raw material handling.<br>Documentation of care: Documentation systems (patient medication profile, formulary systems, policies and standard operating procedures, documentation of interventions)<br><br>• **Competence:**<br>Competence in the dispensing process:<br>Provision of medicines (prescription availability, patient identification, and dispensing).

						Interaction and communication (communication skills of pharmacist and staff, provision of advice, promotion of good health, and provision of written information)<br><br><u>Clinical knowledge and diagnostic skills:</u><br>Supply of OTC medicines (advice on selection and use, responding to minor ailments)<br><br>Health promotion (engagement in health promotion, participation in health promotion campaigns)<br><br>Diagnostics (provision of diagnostic tests, documentation of tests done)<br><br><u>Training of pharmacy staff:</u><br>Research and professional development (participation in research projects, participation in continuing education)<br>Trainees (acceptance of trainees, monitoring and documentation, activity description)<br><br>• **Integration:**<br>Interprofessional collaboration: Development of pharmaceutical care plans, patient monitoring, identification of medication-

						related problems, interaction with other prescribers, and other healthcare professionals
Hindi et al. (2019)[75]	UK	To examine the views of patients, pharmacists and GPs on how community pharmacies are currently used and to identify how community pharmacy services may be better integrated within the primary care pathway for people with long-term conditions	Focus groups	Two focus groups were conducted with respiratory patients (n=6, 5) and two with type 2 diabetes patients (both= 5). Two focus groups were held with pharmacists (n=7, 5) and two with GPs (both n = 5).	• n/a	• **Integration** Communication mechanisms and information systems: All stakeholder groups believed pharmacists required more access to patient information (i.e. medical records) to have a better overall understanding of patients' conditions.
Hindi et al. (2019)[93]	UK	Identify factors that could influence patients to make better use of community pharmacies within the primary care pathway	Survey	289 Patients with asthma or chronic obstructive pulmonary disease registered at two GP practices.	• Medicines supply<br>• Professional pharmacy service	Questionnaire Items looked at factors influencing patient's likelihood to use community pharmacy services:<br>• **Person-centred care:** Professionalism (3); patient-pharmacist relationship (2)<br>• **Environment:** private consultation area (3)<br>• **Access:** physical access (1)<br>• **Integration:** communication mechanisms and information systems – information sharing (6); proximity (2)
Mirzaei et al (2019)[62]	Australia	To build a theory-grounded model of service quality in	Stage 1 dealt with item generation using theory,		• Medicines supply	Dimensions of service quality covered:

		community pharmacies and to create a valid survey instrument to measure consumers' perceptions of service quality.	prior research and qualitative interviews with pharmacy consumers. Selected items were then subjected to content validity and face validity. Stages 2 and 3 included psychometric testing among English-speaking adult consumers of Australian pharmacies. Exploratory factor analysis was used for item reduction and to explain the domains of SQ			• **Person-centred care:** Relationship: Trusting relationship (Relationship) Professionalism: Friendliness/Helpfulness<br>• **Access:** Availability of the pharmacist, waiting time<br>• **Competence:** Competence in the dispensing process: advice, expertise, effectiveness/knowledge, patient health outcome Clinical knowledge and diagnostic skills: services such as blood pressure testing or diabetes support services were minimally discussed and of least importance to patients<br>• **Environment:** physical resources, appearance of the pharmacy; appearance of the pharmacy.
Patterson et al. (2019)[101]	USA	To measure the relative strength of patient preferences for community pharmacy attributes and to describe associations between patient sociodemograph and health characteristics	Questionnaire (Discrete Choice Experiment)	773 American adults (≥18 years) who had filled a prescription at a pharmacy, other than a mail-order pharmacy, within the last 12 months.	• Medicines supply	Quality dimensions covered:<br>• **Access**: opening hours (1)<br>• **Person-centred care:** professionalism (1); patient-pharmacist relationship (1).<br>• **Competence:** Competence in the dispensing process (2)

		and pharmacy preferences				
Watson et al (2019)[28]	UK	Explored citizens' perspectives about the quality of community pharmacy services in the UK and whether and how the quality of community pharmacy services should be measured.	Semi-structured interviews and focus groups	20 individuals participated (Scotland (n=7) all interviewed individually; England (one focus group (n=4) and four individual interviews); and Wales (one focus group (n=5))	• Medicines supply<br>• Professional pharmacy service (supply of OTC medicines following minor illness consultation)	• **Person-centred care:**<br>Patient experience: Friendly caring service,<br>Patient pharmacist relationship: continuity of care, and staff knowing the individual.<br>Professionalism: Professional approach in customer appearance, including behaviour and appearance<br>• **Environment:**<br>Private consultation area: Physical characteristics of the pharmacy in supporting privacy, with either a separate consultation room or dedicated private area, and the need to have confidential conversations with the pharmacist.
Abu Hagar et al (2020)[63]	UAE	To evaluate the present status of risk<br>occurrence in community pharmacies in Abu Dhabi and investigate the protective plans that are followed in risky<br>cases to generate an overall view of risk management plans	Survey	322 licensed community pharmacists in<br>Abu Dhabi	• Medicines supply	• **Environment**<br>Private consultation area: Provide a private consultation area.<br>Appearance of the pharmacy: temperature should be maintained to avoid discomfort.<br>Appearance of the pharmacy: Sufficient and well distributed lights across<br>• **Safety**

		in concurrent pharmacy practice.				Systems for ensuring safety: Keep patient data in separate filing; do not share patient information; discard documents containing patients' data in a proper way<br>• **Competence**<br>Clinical knowledge and diagnostic skills: The most reported reason for adverse drug reactions was lack of knowledge about side effects (cannot recognize ADR cases to report them)<br>• **Integration**<br>The most reported protective measure to avoid medication errors was contacting the GP
Al-Jumaili et al. (2020)[95]	Iraq	To evaluate patient satisfaction with community pharmacy services and measure the relationships between patient satisfaction and pharmacy/pharmacist characteristics and patient quality of life	Survey	400 patients at 20 community pharmacies in 10 different geographical areas in Baghdad city	• Medicines supply.	The questionnaire included:<br>• **Access:** Availability of pharmacist (1)<br>• **Person-centred care:** professionalism (2), patient-pharmacist relationship (1); patient experience (3)<br>• **Competence:** competence in the dispensing process (11)<br>• **Environment:** appearance of the pharmacy (1); private consultation area (1)
Aizpurua-Arruti et al (2020)[66]	Spain	Confirm if the elderly people who go to the pharmacies still think	Focus group	10 elderly people in San Sebastian	• Medicines supply	• **Person-centred care**

		that the commitments that define the Friendly Pharmacy are the ones previously identified				Patient experience: Patients valued patient experience based on trust and intimacy<br>• **Environment**<br>Private consultation area: Participants highlight the positive aspects of accessible spaces with personalised service areas<br>Waiting area: Participants highlight the positive aspects seating areas that facilitate the wait.<br>• **Competence**<br>Competence in the dispensing process: Patients valued advice on use of medicines<br>• **Integration:**<br>Communication mechanisms and information systems: Patients valued good communication with other health care settings; and referral to health and social care.
Badro et al. (2020)[117]	Lebanon	To assess good pharmacy practice (GPP) aspects and compare GPP scores among community pharmacies in Lebanon, using a tool developed jointly by the International Pharmaceutical	Survey which included 109 questions	A team of 10 licensed inspectors who work at the Lebanese Order of Pharmacists and visited 276 community<br>pharmacies across Lebanon	• Medicines supply<br>• Professional pharmacy service	The questionnaire was adapted to the Lebanese context and included questions on:<br>• **Safety:** Documentation of care (25); Systems for ensuring safety (5)<br>• **Competence**: competence in the dispensing process (51); clinical

		Federation (FIP) and the World Health Organization (WHO) to improve and maintain standards of pharmacy practice				knowledge and diagnostic skills (19)<br>• **Environment:** appearance of the pharmacy (1); private consultation area (1)<br>• **Access:** availability of medicines (5); physical resources (3); availability of pharmacy staff (1); appearance of the pharmacy (7)
Bratkowska et al. (2020)[94]	Poland	To evaluate patient satisfaction with services provided in independent pharmacies and pharmacy chains in Poland	Survey	117 patients randomly selected from four community pharmacies in Poland (2 chain pharmacies and 2 independent pharmacies)	• Medicines supply	items divided into the following:<br>• **Access:** Waiting time (1); availability of medicines (2)<br>• **Environment:** Appearance of the pharmacy (1); private consultation area (1)<br>• **Competence:** Competency in the dispensing process (6)<br>• **Person-centred care:** Patient-pharmacist relationship (1); patient experience (2); professionalism (1)<br>•
Goto et al (2020)[65]	Japan	To examine how a patient's continuity with the same pharmacist and pharmacy is associated with their evaluation of the quality of pharmacy services.	Questionnaire	3,492 Patients who regularly visited pharmacies	• Medicines supply<br>• Professional pharmacy service	Survey items covered the following dimensions:<br>• **Person-centred care:**<br>Patient experience (3 items); patient-pharmacist relationship (1 item); professionalism (2 items)

						• **Competence:**<br><br>Competence in the dispensing process (1 item); Clinical knowledge and diagnostic skills (1 item)<br><br>• **Access:**<br><br>Availability of pharmacy staff (1 item), availability of medicines (1 item)
Jacobs et al (2020)[26]	UK	To investigate organisational factors associated with variation in safety climate, patient satisfaction and self-reported medicines adherence in English community pharmacies.	Multivariable regressions were conducted using data from two cross-sectional surveys (1. PSCQ and 2. patient satisfaction with visit)	277 pharmacists and 971 patients visiting 39 pharmacies, across 9 diverse geographical areas.	• Medicines supply<br><br>• Professional pharmacy service	• **Safety:** Safety climate was associated with organisational culture.
Sato et al (2020)[64]	Japan	In Japan, a new system called Health Support Pharmacy (HSP) was introduced in 2016, to promote responsible self-medication with non-prescription medicines and increase awareness of public health activities provided through community pharmacies.	Semi-structured interviews	Twenty-four community pharmacists from across Japan.	• Professional pharmacy service	• **Pharmacy environment:** physical resources<br><br>• **Competence:**<br><br>Clinical knowledge and diagnostic skills: professional expertise (effectiveness, professionalism, teamwork, scope and duration of expertise).

		This study aimed to identify factors that can impact on the quality of HSP services provided by community pharmacists in Japan.				Provision of community health education and other events (health promotion techniques, event planning).<br><br>Impact on individuals and the general public (economic outcomes; clinical outcomes humanistic outcomes; health behaviour chance, pharmacy as a community hub); impact on other professional (reassurance and operation efficiency)<br><br>• **Integration**<br>Interprofessional collaboration: interactions between the community and the pharmacy; collaboration with other professionals
Tran et al. (2020)[100]	Vietnam	To understand elderly pharmacy users' satisfaction on the community pharmacy services in Ho Chi Minh City, Vietnam.	Q- methodology	32 pharmacy users, aged over 60, was recruited in four pharmacies in Ho Chi Minh City, Vietnam,	• Medicines supply.	Statements divided into the dimensions of quality:<br><br>• **Competence:** Competency in the dispensing process (22)<br>• **Environment:** Appearance of the pharmacy (1); waiting area (1); private consultation area (1); appearance of the pharmacy (1); dispensing (1)<br>• **Access:** opening hours (3); availability of medicines (2);

						physical access (1); availability of pharmacist (1)<br>• **Person-centred care:** professionalism (3)
Waltering et al. (2020)[111]	Germany	To develop indicators for assessing the quality of medication review in public pharmacies	Delphi	The expert group in the Delphi survey consisted of 22 participants. These were pharmacists, representatives of the health insurance companies, scientific Staff members of various institutes and one member each from an association of panel doctors and a chamber of pharmacists	• Professional pharmacy service	After two rounds of Delphi, a final set of indicators consisting of<br>• **Safety:** Documentation of care (1); Systems for ensuring safety (1)<br>• **Competence:** clinical knowledge and diagnostic skills (4)
Watson et al (2020)[30]	UK	To conceptualise GPs' perceptions and beliefs about the quality of community pharmacy services in general and, more specifically, using the concept of 'always events' and the management of acute consultations.	Semi-structured interviews	20 GPs (Scotland n=12, England n= 8)	• Medicines supply<br>• Professional pharmacy service (sale of OTC medicines following minor illness consultation)	• **Access**<br>Physical access: GPs suggested that pharmacies should be accessible and near to the population that they serve<br>Opening hours: Pharmacies should have extended opening hours for the convenience of patients and known to GPs.<br>Availability of medicines: Most GPs said that pharmacies should hold an adequate, well managed stock of medication (and alternatives) and other medical devices or be able to obtain them quickly.

						• **Person-centred care:**<br>Patient experience: GPs suggested that pharmacists should offer personalised care, with good listening and communication skills and all staff should have a positive orientation to patients/customers. They should involve people in decisions, treat them with sincerity, and always check their understanding of treatment; they should be kind but not "paternalistic".<br><br>• **Competence:**<br>Clinical knowledge and diagnostic skills: pharmacists should identity problems relating to drug efficacy, side effects and compliance.<br><br>• **Environment:**<br>Private consultation area: have at least on dedicated room, or at least a spate/private area, and to take a proactive approach in offering it to patients/customers.<br><br>• **Integration:**<br>Communication mechanisms and information systems: There were mixed views around the recording and sharing patient information.

Abebe et al. (2021)[69]	USA	To characterize documentations in community pharmacies and to examine factors that contribute to perceived documentation of care quality.	Survey	445 community pharmacists in Wisconsin	• Medicines supply	• **Safety**<br>Documentation of care: 20<br>Survey items covered: Handover procedures; frequency of handovers; Handover training; Handover outcomes; Technology; Handover resolution
Clabaugh et al (2021)[67]	USA	To determine pharmacists' perceptions of working conditions while controlling for respondent (years of experience, degree, work status) and workplace variables (prescription volume, type of community setting).	Survey	1222 pharmacists<br><br>48 of 50 states	Not specified	Qualitative analysis of survey identified features relative to<br><br>• **Safety:** Supervision, work design, quality emphasis, group behaviour.<br>• **Person-centred care:** patient expectations.
Thang et al. (2021)[96]	Vietnam	To identify factors that affect the overall satisfaction of customers visiting community pharmacies in Vietnam.	Survey	354 patients at 13 randomly selected community pharmacies in five districts in Hanoi, Vietnam.	• Medicines supply.<br><br>• Professional pharmacy service (sale of OTC medicines following minor illness consultation)	Questionnaire items covering:<br>• **Person-centred care:** professionalism (2); personalised care (1)<br>• **Competence:** competence in the dispensing process (6); clinical knowledge and diagnostic skills (2)<br>• **Access:** physical location (2); opening hours (1); availability of medicines (1)

						• **Environment:** appearance of the pharmacy (1); appearance of the pharmacy and hygiene (1); private consultation area (1)
Fernandes et al. (2021)[113]	Brazil	To develop an instrument to evaluate the quality of services provided in community pharmacies, as well as to test its application through a geographic information system for the visualization of the results	After a review of the scientific literature, a set of quality indicators was submitted to expert analysis. From the final constructed matrix, observational and self-administered questionnaires were elaborated and applied in pharmacies belonging to a city of the South eastern region of Brazil.		• Medicines supply	Items covered 5 dimensions of quality:<br>• **Access:** opening hours; physical access<br>• **Environment:** Physical resources; dispensary; private consultation area; appearance of the pharmacy<br>• **Competence:** Competency in the dispensing process<br>• **Safety:** Systems for ensuring safety; dispensing
Loo et al (2021)[68]	UK	Explored the content of online feedback provided by patients from across the UK in relation to their experiences of their interaction with pharmacy staff and pharmacy services	Patient stories published on Care Opinion, a national online patient feedback platform, for a one-year period were searched for all content relating to patients'	237 patient stories	• Medicines supply<br>• Professional pharmacy service	Online feedback provided by patients mapped on the following dimensions of quality:<br>• **Person-centred care:** Made up the highest proportion of patient feedback relating to community pharmacy with most feedback being positive. Helpfulness, professionalism, kindness, friendliness, politeness were

			pharmacy experiences.			common terms used to describe pharmacy staff across all settings.<br><br>• **Competence:** Patient feedback was overall positive regarding the services that pharmacies offer such as healthcare advice, clinical services (e.g. community pharmacy blood pressure checks, minor ailment services) as well as ordering repeat prescriptions and delivery services.<br><br>• **Safety:** Medication errors were only mentioned in community pharmacy related stories whereby medications were mistakenly dispensed.<br><br>• **Access:** Patient stories often related to the accessibility of the pharmacy. Overall, accessibility was positively (28/40, 70%) described by patients. Several stories related to the convenience of community pharmacies attached to or within GP surgeries. Opening times of community pharmacies were appreciated in a small number of stories.<br><br>• **Environment:** A minority of patient feedback related to the environment of the pharmacy and these were mainly negative.

						Patients complained of crowding within some community pharmacies
Mohamud et al. (2021)[97]	Sudan	To explore patients' satisfaction level with pharmacist's communication, consulting and service delivery qualities.	Survey	385 patients from 229 community pharmacies in Khartoum	• Medicines supply.	Questionnaire items covered:<br>• **Person-centred care:** patient experience (3); professionalism (3)<br>• **Access:** waiting time (1); availability of pharmacy staff (1)<br>• **Competence:** competency in the dispensing process (6)<br>• **Environment:** private consultation area (1)
Nneoma et al. (2021)[112]	Nigeria	To develop quality indicators for assessing pharmaceutical care performance in the Nigerian community pharmacies.	Delphi	A panel of 10 pharmacy experts in Nigeria	• Medicines supply	Indicators categorised under the following dimensions:<br>• **Safety:** Systems for ensuring safety (9); Documentation of care (4); medicine supply (3)<br>• **Competence:** Competency in the dispensing process (5); Training of pharmacy staff (2)
Sepp et al. (2021)[118]	Estonia	To identify the implementation of the Community Pharmacy	Questionnaire	The cross-sectional electronic surveys were conducted among community pharmacies	• Medicines supply	Items covered 4 dimensions of quality:<br>• **Safety:** Documentation of care

		Services Quality Guidelines (CPSQG) as a profession-driven initiative towards improving and harmonizing community pharmacy services in Estonia.		in Estonia in 2014 (N = 478 pharmacies), 2016 (N = 493), and 2019 (N = 494)	• Professional pharmacy service	• **Competence:** Competency in the dispensing process ; clinical and diagnostic skills<br>• **Environment**: Private consultation area indicators; waiting area
Wongvedvanij et al (2022)[70]	Thailand	To investigate how patients perceive different dimensions of service quality, especially for non-prescription medicines during the COVID-19 outbreak.	Survey	378 Thai patients during the spread of COVID-19.	• Professional pharmacy service (sale of OTC medicines following minor illness consultation)	Survey items covered 4 domains of quality:<br>• **Competence**: Clinical knowledge and diagnostic skills (6 items)<br>• **Access:** opening hours (1 item)<br>• **Safety:** documentation of care (2 items)<br>• **Person-centred care**: Patient experience (7 items); professionalism (6 items)
Schomer et al (2022)[71]	USA	This study applied a human factors and ergonomics approach to describe community-based pharmacy personnel perspectives regarding how work environment characteristics affect the ability to perform the duties necessary for optimal patient care and how contributors to	Survey	4606 pharmacists and pharmacy technicians	Not specified	There were 12 items developed for the survey that focused on **safety**

		stress affect the ability to ensure patient safety.				
Wongvedvanij et al (2022)[72]	Thailand	To explore pharmacists' and patients' perception of potential pharmacy service quality for dispensing non-prescription medicines.	Semi-structured interviews	14 pharmacists and 20 patients.	• Professional pharmacy service (sale of OTC medicines following minor illness consultation)	• **Competence:**<br>Clinical knowledge and diagnostic skills:<br>ability, skills, knowledge, expertise to diagnose patient symptoms before dispensing non-prescription medicines, pharmacist must obtain accurate personal information using different communication channels<br>• **Person-centred care:**<br>Patient-pharmacist relationship: pharmacists having ongoing interactions and developing personal relationships with their patients over a period of time. This relationship encompasses mutual trust, loyalty respect and knowledge.<br>Patient experience: Pharmacists pay attention to individual patients and treat them based on their personal health conditions and requirements<br>• **Environment:**<br>Cleanliness & hygiene*:* clean and hygienic physical space and equipment used to handle pharmaceutical products

Kummer et al (2022)[73]	Serbia	To examine patients' perceptions of an incident that occurred in community pharmacies using CIT and determine recommendations for improving the quality of pharmacy services.	Interviews	20 patients from 3 community pharmacies in Serbia	• Medicines supply	A total of 68 critical incidents were collected and divided into two groups: positive (37) and negative (31), depending on patients' satisfaction/dissatisfaction with community pharmacy services. Critical incidents covered:<br>• **Access:** opening hours, availability of pharmacy staff, physical access, waiting time; availability of medicines<br>• **Competence:** Competence in the dispensing process<br>• **Person-centred care**: professionalism; patient experience<br>• **Safety:** Compounding
Parinyarux et al. (2022)[98]	Thailand	to explore the satisfaction of the community pharmacy users with the facilities and services received from drugstores under the GPP standards and examine the impact of satisfaction toward each GPP domain on overall satisfaction (OS) and the intention to receive the pharmacy services as the first choice in the	Survey	388 community pharmacy users	• Medicines supply.<br>• Professional pharmacy service	Questionnaire items categorised under the following dimensions:<br>• **Safety:** Systems for ensuring safety (2); medicine supply (4)<br>• **Environment:** dispensary (3); private consultation area (1); physical resources (2)<br>• **Competence:** competence in the dispensing process (4); clinical knowledge and diagnostic skills (1)

		case of common and non-serious illnesses (IntR).				• **Person-centred care:** professionalism (4)
Sepp et al (2022)[74]	Estonia	To evaluate to what extent the patient-centred care (PCC) principles are included in the Community Pharmacy Services Quality Guidelines (CPSQG) in Estonia	Deductive content analysis was performed using the PCC framework developed by Santana et al.		• Medicines supply<br>• Professional pharmacy service	• **Access**. This included availability of medicines and more broadly access to diagnostic testing such as Blood pressure blood sugar measurement<br>• **Person-centred care:** Cultivating communication; respectful and compassionate care; engaging patients and managing their care; integration of care

**studies are ordered chronologically*
