# Supplementary material for: Developing a quality framework for community pharmacy: a systematic review of international literature
Source: BMJ Open. 2024 Feb 15;14(2):e079820. doi: 10.1136/bmjopen-2023-079820 (PMC10875530; doi:10.1136/bmjopen-2023-079820)
Supplement: Supplementary data [file bmjopen-2023-079820supp003.pdf]

Supplementary File 3: Critical Appraisal

Critical appraisal of qualitative studies using the JBI checklist for qualitative research

- No, Unclear = 0
- Yes = 1

	**1** *Philosophical perspective*	**2.** *Research question*	**3.** *Methods*	**4.** *Data analysis*	**5.** *Results*	**6.** Researcher orientation	**7.** *Researcher influence*	**8.** *Participants*	**9.** *Ethics*	**10.** *Conclusions*	**Total score (max: 10)**
Aizpurua-Arruti et al (2020)[66]	N	Y	Y	N	N	N	N	Y	N	N	3
White et al. (2010)[48]	Y	Y	Y	Y	N	N	N	N	N	N	4
Scahill et al. (2010)[46]	Y	Y	Y	Y	Y	N	N	N	N	Y	6
Löffler et al. (2017)[78]	Y	Y	Y	Y	N	N	N	N	Y	Y	6
Loo et al (2021)[68]	Y	N	N	Y	Y	N	N	Y	Y	Y	6
Shiyanbola et al. (2016)[56]	Y	Y	Y	Y	Y	N	N	N	Y	Y	7

Watson et al (2019)[28]	Y	Y	Y	Y	Y	N	N	N	Y	Y	7
Kummer et al (2022)[73]	N	Y	Y	Y	Y	N	N	Y	Y	Y	7
Rapport et al. (2009)[45]	Y	Y	Y	Y	Y	Y	N	Y	N	Y	8
Halsall et al. (2012)[27]	Y	Y	Y	Y	Y	N	N	Y	Y	Y	8
White et al. (2012)[52]	Y	Y	Y	Y	Y	N	N	Y	Y	Y	8
Jacobs et al. (2018)[15]	Y	Y	Y	Y	Y	N	N	Y	Y	Y	8
Fujita et al. (2019)[60]	Y	Y	Y	Y	Y	N	N	Y	Y	Y	8
Sato et al (2020)[64]	Y	Y	Y	Y	Y	N	N	Y	Y	Y	8
Wongvedvanij et al (2022)[72]	Y	Y	Y	Y	Y	N	N	Y	Y	Y	8
Rubio-Valera et al (2012)[71].	Y	Y	Y	N	Y	Y	Y	Y	Y	Y	9

McMilan et al. (2014)[86]	Y	Y	Y	Y	Y	Y	N	Y	Y	Y	9
Hattingh et al. (2015)[55]	Y	Y	Y	Y	Y	N	Y	Y	Y	Y	9
Watson et al. (2018)[29]	Y	Y	Y	Y	Y	Y	N	Y	Y	Y	9
Hindi et al. (2019)[75]	Y	Y	Y	Y	Y	Y	Y	Y	Y	Y	10
Watson et al (2020)[30]	Y	Y	Y	Y	Y	Y	Y	Y	Y	Y	10
**Mean**	**0.9**	**0.9**	**0.9**	**0.9**	**0.9**	**0.3**	**0.2**	**0.8**	**0.8**	**0.9**	**7.5**

Description of criteria

1. *Congruity between the stated philosophical perspective and the research methodology.*
2. *Congruity between the research methodology and the research question or objectives.*
3. *Congruity between the research methodology and the methods used to collect data.*
4. *Congruity between the research methodology and the representation and analysis of data.*
5. *There is congruence between the research methodology and the interpretation of results.*
6. *Locating the researcher culturally or theoretically.*
7. *Influence of the researcher on the research, and vice-versa, is addressed.*
8. *Representation of participants and their voices.*
9. *Ethical approval by an appropriate body.*
10. *Relationship of conclusions to analysis, or interpretation of the data.*

Critical appraisal of quantitative studies using the JBI checklist for analytical cross-sectional studies

- No, Unclear = 0
- Yes = 1

	1. Sample	2. Subjects and setting	3. Exposure	4. Standard criteria	5. Confounding	6. Cofounding effects	7. Outcomes	8. statistical analysis	**Total score (max: 8)**
Harding et al. (2010)[114]	N	Y	N	N	N	N	N	Y	2
Al-Jumaili et al. (2020)[95]	Y	N	N	N	N	N	N	Y	2
Wongvedvanij et al (2022)[70]	N	N	N	N	N	N	Y	Y	2
Júnior et al. (2017)[110]	Y	Y	N	N	N	N	N	N	2
Vilako et al. (2007)[81]	Y	Y	N	N	N	N	N	Y	3
Hashemian et al. (2016)[79]	N	N	Y	Y	N	N	Y	N	3
Bratkowska et al. (2020)[94]	Y	Y	N	N	N	N	N	Y	3
Arkaravichien et al. (2016)[108]	Y	N	N	N	N	N	Y	Y	3

Kelly et al. (2013)[76]	Y	Y	N	N	N	N	Y	Y	4
Odukoya et al. (2014)[54]	N	Y	N	N	Y	Y	N	Y	4
Chen et al. (2015)[88]	N	Y	Y	N	Y	Y	N	N	4
Alhomoud et al. (2016)[89]	Y	Y	Y	Y	N	N	N	N	4
Parinyarux et al. (2022)[98]	N	N	N	N	Y	Y	Y	Y	4
Badro et al. (2020)[117]	Y	Y	N	N	Y	Y	N	N	4
Urbonas et al. (2010)[47]	N	Y	Y	Y	N	N	Y	Y	5
Schomer et al (2022)[71]	Y	Y	Y	Y	N	N	Y	N	5
Sepp et al. (2021)[118]	Y	N	Y	Y	N	N	Y	Y	5
Nilugal et al. (2016)[90]	Y	Y	Y	Y	N	N	Y	N	5
Hindi et al. (2019)[93]	Y	Y	N	N	Y	Y	N	Y	5
Jacobs et al 2020	Y	Y	N	N	Y	Y	N	Y	5

Clabaugh et al (2021)[67]	Y	Y	N	N	Y	Y	N	Y	5
Thang et al. (2021)[96]	Y	Y	N	N	Y	Y	Y	N	5
Feehan et al. (2017)[91]	Y	Y	Y	Y	N	N	N	Y	5
Phipps et al. (2012)[53]	Y	Y	Y	Y	N	N	Y	Y	6
Worley (2006)[44]	Y	Y	Y	Y	N	N	Y	Y	6
Feletto et al. (2010)[49]	Y	Y	Y	Y	N	N	Y	Y	6
Patterson et al. (2013)[84]	Y	Y	Y	Y	N	N	Y	Y	6
Aziz et al. (2018)[92]	Y	Y	Y	Y	N	N	Y	Y	6
Abu Hagar et al (2020)[63]	Y	Y	Y	Y	N	N	Y	Y	6
Goto et al (2020)[65]	Y	Y	Y	Y	N	N	Y	Y	6
Mohamud et al. (2021)[97]	Y	Y	Y	Y	N	N	Y	Y	6
Patterson et al. (2019)[101]	Y	N	Y	Y	Y	Y	N	Y	6

Koster et al	Y	Y	Y	Y	N	N	Y	Y	6
Sakurai et al. (2009)[82]	Y	Y	Y	Y	Y	N	Y	Y	7
Mehralian et al. (2014)[87]	N	Y	Y	Y	Y	Y	Y	Y	7
Abebe et al. (2021)[69]	Y	Y	Y	Y	Y	Y	N	Y	7
Merks et al. (2014)[85]	Y	Y	Y	Y	Y	Y	Y	Y	8
Guhl et al (2019)[61]	Y	Y	Y	Y	Y	Y	Y	Y	8
**Mean**	**0.8**	**0.8**	**0.6**	**0.6**	**0.4**	**0.3**	**0.6**	**0.8**	**4.9**

Description of criteria

1. Were the criteria for inclusion in the sample clearly defined?
2. Were the study subjects and the setting described in detail?
3. Was the exposure measured in a valid and reliable way?
4. Were objective, standard criteria used for measurement of the condition?
5. Were confounding factors identified?
6. Were strategies to deal with confounding factors stated?
7. Were the outcomes measured in a valid and reliable way?
8. Was appropriate statistical analysis used?

Critical appraisal of Delphi studies using the Conducting and REporting of DElphi Studies (CREDES) checklist

- No, Unclear = 0
- Yes = 1
- N.A = Not counted in total score

	**1.** Rationale	**2.** Expert panel	**3.** Methods	**4.** Procedure	**5.** Consensus	**6.** Results	**7.** Discussion	**8.** Conclusions	**9.** Dissemination	**Total score (max: 9)**
Nneoma et al. (2021)[112]	Y	N	Y	N	Y	N	N	Y	N	4
Horvat et al. (2011)[83]	Y	Y	N	N	Y	Y	N	N	Y	5
Fernandes et al. (2021)[113]	Y	N	Y	N	N	N	Y	Y	Y	5
Grey et al. (2016)[103]	Y	N	N	N	Y	Y	Y	Y	Y	6
Puumalainen et al. (2005)[102]	Y	N	Y	N	Y	Y	Y	Y	Y	7
Waltering et al. (2020)[111]	Y	N	N	Y	Y	Y	Y	Y	Y	7
James et al. (2008)[103]	Y	Y	Y	Y	Y	Y	Y	Y	N	8
Newlands et al. (2018)[59]	Y	Y	Y	N	Y	Y	Y	Y	Y	8
De Bie et al. (2011)[105]	Y	Y	Y	Y	Y	Y	Y	Y	Y	9
**Mean**	1	0.4	0.7	0.3	0.9	0.8	0.8	0.9	0.8	**6.6**

Description of criteria:

1. *Purpose and rationale: The purpose of the study should be clearly defined and demonstrate the appropriateness of the use of the Delphi technique as a method to achieve the research aim. A rationale for the choice of the Delphi technique as the most suitable method needs to be provided.*
2. *Expert Panel: Criteria for the selection of experts and transparent information on recruitment of the expert panel, sociodemographic details including information on expertise regarding the topic in question, (non)response and response rates over the ongoing iterations should be reported.*
3. *Description and methods: The methods employed need to be comprehensible; this includes information on preparatory steps (How was available evidence on the topic in question synthesised?), piloting of material and survey instruments, design of the survey instrument(s), the number and design of survey rounds, methods of data analysis, processing and synthesis of experts' responses to inform the subsequent survey round and methodological decisions taken by the research team throughout the process.*
4. *Procedure: Flow chart to illustrate the stages of the Delphi process, including a preparatory phase, the actual 'Delphi rounds', interim steps of data processing and analysis, and concluding steps*
5. *Definition and attainment of consensus: It needs to be comprehensible to the reader how consensus was achieved throughout the process, including strategies to deal with non-consensus.*
6. *Results: Reporting of results for each round separately is highly advisable in order to make the evolving of consensus over the rounds transparent. This includes figures showing the average group response, changes between rounds, as well as any modifications of the survey instrument such as deletion, addition or modification of survey items based on previous rounds.*
7. *Discussion and limitation: Reporting should include a critical reflection of potential limitations and their impact of the resulting guidance.*
8. *Adequacy of conclusions: The conclusions should adequately reflect the outcomes of the Delphi study with a view to the scope and applicability of the resulting practice guidance.*

9. *Publication and dissemination: The resulting guidance on good practice should be clearly identifiable from the publication, including recommendations for transfer into practice and implementation.*

Critical appraisal of mixed methods studies using the Mixed Methods Appraisal Tool (MATT)

- No, Can't tell [CT] = 0
- Yes = 1

	Screening		Qualitative part				Quantitative part					Mixed Methods Part					
**Study**	**1**	**2.**	**3.**	**4.**	**5.**	**6.**	**7.**	**8.**	**9.**	**10.**	**11.**	**12.**	**13.**	**14**	**15**	**16.**	**17.**	**Total score (Max: 17)**
Dadfar et al. (2012)[51]	Y	Y	N	Y	N	N	Y	CT	N	Y	CT	Y	Y	N	N	Y	N	8
Snyder et al (2010)[75]	Y	Y	Y	Y	Y	Y	N	N	Y	N	N	Y	Y	N	N	Y	N	10
Weiss et al (2016)[57]	Y	Y	Y	Y	Y	Y	Y	Y	Y	Y	Y	Y	Y	Y	Y	Y	Y	17
Mirzaei et al (2019)[62]	Y	Y	Y	Y	Y	Y	Y	Y	Y	Y	Y	Y	Y	Y	Y	Y	Y	17
**Mean**	1	1	0.75	1	0.75	0.75	0.75	0.5	0.75	0.75	0.5	1	1	0.5	0.5	1	0.5	**13**

<u>Screening questions</u>
1. Are there clear research questions?
2. Do the collected data allow to address the research questions?

<u>Qualitative questions</u>

3. Is the qualitative approach appropriate to answer the research question?
4. Are the qualitative data collection methods adequate to address the research question?
5. Are the findings adequately derived from the data?
6. Is the interpretation of results sufficiently substantiated by data?
7. Is there coherence between qualitative data sources, collection, analysis and interpretation?

Quantitative questions

8. Is the sampling strategy relevant to address the research question?
9. Is the sample representative of the target population?
10. Are the measurements appropriate?
11. Is the risk of nonresponse bias low?
12. Is the statistical analysis appropriate to answer the research question?

Mixed Methods questions

13. Is there an adequate rationale for using a mixed methods design to address the research question?
14. Are the different components of the study effectively integrated to answer the research question?
15. Are the outputs of the integration of qualitative and quantitative components adequately interpreted?
16. Are divergences and inconsistencies between quantitative and qualitative results adequately addressed?
17. Do the different components of the study adhere to the quality criteria of each tradition of the methods involved?
