# Supplementary material for: Developing a quality framework for community pharmacy: a systematic review of international literature
Source: BMJ Open. 2024 Feb 15;14(2):e079820. doi: 10.1136/bmjopen-2023-079820 (PMC10875530; doi:10.1136/bmjopen-2023-079820)
Supplement: Supplementary data [file bmjopen-2023-079820supp004.pdf]

Supplementary file 4: Input from PPI group summarised under the dimensions/sub-dimensions of quality framework

Dimensions	Input from PPI Group	Incorporation of PPI input into framework dimensions
**Patient Experience**	Professionalism:<br>- Mannerisms of staff in some pharmacies is poor.<br>- Anyone who has any interaction with customers' needs to be polite.<br>- Staff do not have IDs which makes it hard to distinguish between staff.<br>Patient experience:<br>- Patients need to be put at the forefront of decision making – where is the patient voice?	Professionalism:<br>- *Pharmacy staff being distinguishable by wearing a name badge with their role.*<br>- *Mannerisms of pharmacy staff*<br>*Patient Experience:*<br>- *Involving patients in decision making*
**Integration**	- Integration is a huge element of quality. No sense that GPs/pharmacists speak to each other. GPs direct patients to pharmacies but pharmacies seem to be unaware. Pharmacies then direct patients back to GPs.<br>.	Communication mechanisms and information systems*:*<br>- *Bi-directional communication between CPs and other providers.*
**Access**	Waiting times<br>- Services have gone downhill at community pharmacy. It takes longer to get a prescription.<br>Availability of medicines<br>- Difficult to encourage public to go to pharmacy for advice if they can't even get medications<br>- Free prescription is not always available<br>Availability of pharmacy staff<br>- Huge tensions around single pharmacists having to do everything but not accessible to patients.	Waiting times<br>- *Importance of waiting time for receiving prescriptions*<br>Availability of medicines<br>- *Availability of medicines and offering alternatives.*<br>Availability of pharmacy staff<br>- *Availability of pharmacy staff.*<br>- *Having adequate numbers and appropriately qualified pharmacy staff*

	- Training received by pharmacy staff questionable.<br>- There needs to be more training of pharmacy staff and this needs to be funded.	
**Environment**	Appearance of the pharmacy<br>Unlike GP or dentist, going to community pharmacy is like going to a shop. Not a healthcare environment.<br><br>Private consultation area<br>- Privacy issues – asking details such as address, DOB in front of people.<br>- Providing room in pharmacies for consultations is a good thing	Private consultation area<br>- *The importance of privacy and having a consultation area.*
