# Supplementary material for: Developing a quality framework for community pharmacy: a systematic review of international literature
Source: BMJ Open. 2024 Feb 15;14(2):e079820. doi: 10.1136/bmjopen-2023-079820 (PMC10875530; doi:10.1136/bmjopen-2023-079820)
Supplement: Supplementary data [file bmjopen-2023-079820supp005.pdf]

Supplementary File 5: Summary of key dimensions identified in this review

Dimension	Type*
**Access**	
• **Opening hours:** availability of pharmacy services during stated opening hours and extended opening hours	**S**
• **Waiting time:** minimising wait times for healthcare services	**P**
• **Availability of pharmacy staff:** *Having adequate numbers and appropriately qualified pharmacy staff for the community pharmacy to operate*	**S**
• **Physical access:** *parking space near the pharmacy; accessibility for people with special needs (e.g. visually impaired, people with baby carriages); geographical proximity and location (e.g. ease of access of community pharmacies via public transportation; work/home; other healthcare facilities)*	**S**
• **Availability of medicines:** *maintaining an adequate, well managed stock of essential medicines as well as other medical devices. Pharmacists providing patients with information about alternative medicines and their prices.*	**S**
**Environment**	
• **Appearance of the pharmacy:** p*harmacy appearing health service orientated by clearly displaying medicines and informational materials. Pharmacy having sufficient counters for dispensing medicines and adequate physical space for pharmacy staff to provide health promotion, education, consultation or screening services to individuals or groups.* Cleanliness & hygiene *of the pharmacy maintained to promote a good impression of the pharmacy.*	**S**
• **Waiting area:** *Ensuring that the waiting area has sufficient space and seating. Informing patients of waiting times and the reasons for any delays.*	**S**
• **Dispensary:** *Well organised and spacious designed to ensure efficient processing of prescriptions. Storage shelves clearly labelled with drug classifications and medicines are kept according to the drug classifications*	**S**
• **Physical resources:** *having drug information systems and resources to ensure provision of additional healthcare services.*	**S**
• **Private consultation area:** *Having a sufficiently sized dedicated area for consultations in the pharmacy and proactively offering it to patients.*	**S**
**Competence**	
• **Competence in the dispensing process:** *Accuracy of dispensing; appropriate advice on medication (usage, storage etc.) and non-medication (diet, exercise) aspects; gathering essential patient information as part of the dispensing process.*	**P**
• **Clinical knowledge and diagnostic skills:** *Having knowledge in disease areas and diagnostic skills to assess patients and provide effective treatment. To be able to assess and refer patients to a GP or other health care provider if necessary.*	**P**

**Person-centred care**	
• **Patient experience:** *understanding individual needs, involving patient in decision around medications, tailoring delivery of services to people with special needs or minority groups. Involving patients in decision making*	**P**
• **Patient-pharmacist relationship** *built on trust, friendliness/helpfulness, continuity of care, and availability of the pharmacist.*	**P**
• **Professionalism:** *pharmacy staff treating patients with respect, showing empathy, expressing honest opinions, regarding patient benefit as top priority. Pharmacy staff being distinguishable by wearing a name badge with their role.*	**P**
**Safety**	
• **Compounding:** *Availability of standard operating procedures to ensure accuracy in compounding.*	**S**
• **Dispensing:** *having clear standard operating procedure for checking prescriptions; dispensing medications (particularly high-risk medications); availability of protocols and guidelines for asking patients about potential drug contraindications and drug-drug interactions. Having structured safety protocols for OTC consultations, including safeguarding.*	**S**
• **Systems for ensuring safety:** *having a quality and safety management system in place for: registering errors made during dispensing; handling near-misses and dispensing errors; evaluating patient experiences and recording the number of patient complaints.*	**S**
• **Documentation of care:** *Accurate recording of relevant information such as medical history and use of medication in a way that can be read and interpreted by other healthcare professionals. Ensuring patient personal information is stored in a confidential manner and discarded properly. Ensuring exchange of information, responsibility, and accountability when a pharmacist concludes a shift, and another replaces this outgoing pharmacist at the beginning of a new shift within the same pharmacy*	**P**
**Integration**	
• **Interprofessional collaboration**: *Establishing an active relationship between community pharmacy and wider healthcare team based on a shared understanding of competences, roles and responsibilities.*	**P**
• **Communication mechanisms and information systems:** having predefined and clear ways to communicate with other healthcare providers. Having shared communication systems between community pharmacy and the rest of the healthcare system. Ensuring bi-directional communication with other healthcare providers	**P**

**S= structure, P= process*
