# Supplementary material for: Developing a quality framework for community pharmacy: a systematic review of international literature
Source: BMJ Open. 2024 Feb 15;14(2):e079820. doi: 10.1136/bmjopen-2023-079820 (PMC10875530; doi:10.1136/bmjopen-2023-079820)
Supplement: Author's manuscript [file bmjopen-2023-079820.draft_revisions.pdf]

BMJ Open is committed to open peer review. As part of this commitment we make the peer review history of every article we publish publicly available.

When an article is published we post the peer reviewers' comments and the authors' responses online. We also post the versions of the paper that were used during peer review. These are the versions that the peer review comments apply to.

The versions of the paper that follow are the versions that were submitted during the peer review process. They are not the versions of record or the final published versions. They should not be cited or distributed as the published version of this manuscript.

BMJ Open is an open access journal and the full, final, typeset and author-corrected version of record of the manuscript is available on our site with no access controls, subscription charges or pay-per-view fees (http://bmjopen.bmj.com).

If you have any questions on BMJ Open's open peer review process please email info.bmjopen@bmj.com

BMJ Open

Developing a quality framework for community pharmacy: A systematic review of international literature

Journal:	*BMJ Open*
Manuscript ID	bmjopen-2023-079820
Article Type:	Original research
Date Submitted by the Author:	13-Sep-2023

Complete List of Authors:	Hindi, Ali; The University of Manchester, Centre for Pharmacy Workforce Studies, Division of Pharmacy and Optometry<br>Jacobs, Sally; Manchester Univ.<br>Schafheutle, Ellen; The University of Manchester, Stopford Building, Oxford Road, Manchester, Division of Pharmacy, School of Health Sciences, Faculty of Biology Medicine and Health<br>Campbell, Stephen; University of Manchester, Centre for Primary Care; Sefako Makgatho Health Sciences University, Department of Public Health Pharmacy and Management, School of Pharmacy

Keywords:	Health policy < HEALTH SERVICES ADMINISTRATION & MANAGEMENT, Quality in health care < HEALTH SERVICES ADMINISTRATION & MANAGEMENT, Systematic Review

SCHOLARONE™
Manuscripts

I, the Submitting Author has the right to grant and does grant on behalf of all authors of the Work (as defined in the below author licence), an exclusive licence and/or a non-exclusive licence for contributions from authors who are: i) UK Crown employees; ii) where BMJ has agreed a CC-BY licence shall apply, and/or iii) in accordance with the terms applicable for US Federal Government officers or employees acting as part of their official duties; on a worldwide, perpetual, irrevocable, royalty-free basis to BMJ Publishing Group Ltd ("BMJ") its licensees and where the relevant Journal is co-owned by BMJ to the co-owners of the Journal, to publish the Work in this journal and any other BMJ products and to exploit all rights, as set out in our licence.

The Submitting Author accepts and understands that any supply made under these terms is made by BMJ to the Submitting Author unless you are acting as an employee on behalf of your employer or a postgraduate student of an affiliated institution which is paying any applicable article publishing charge ("APC") for Open Access articles. Where the Submitting Author wishes to make the Work available on an Open Access basis (and intends to pay the relevant APC), the terms of reuse of such Open Access shall be governed by a Creative Commons licence – details of these licences and which Creative Commons licence will apply to this Work are set out in our licence referred to above.

Other than as permitted in any relevant BMJ Author's Self Archiving Policies, I confirm this Work has not been accepted for publication elsewhere, is not being considered for publication elsewhere and does not duplicate material already published. I confirm all authors consent to publication of this Work and authorise the granting of this licence.

For peer review only

Developing a quality framework for community pharmacy: A systematic review of international literature

Authors: Ali M.K. Hindi* (PhD),[1] Stephen M. Campbell (PhD),[2,3] Sally Jacobs (PhD),[1] Ellen I. Schafheutle (PhD)[1]

*Author for correspondence

Full postal address for corresponding author:

1. Centre for Pharmacy Workforce Studies, Division of Pharmacy and Optometry; School of Health Sciences; Faculty of Biology, Medicine and Health, The University of Manchester, Oxford Road, Manchester M13 9PT, United Kingdom

   Email: ali.hindi@manchester.ac.uk

2. School of Health Sciences; Faculty of Biology, Medicine and Health, The University of Manchester, Oxford Road, Manchester M13 9PT, United Kingdom

3. Department of Public Health Pharmacy and Management, School of Pharmacy, Sefako Makgatho Health Sciences University, Molotlegi Street, Pretoria 0208, South Africa

For peer review only

Abstract

Background: Community pharmacy (CP) is the most frequently used primary care service globally and can be more accessible than medical care. However, without agreement on what constitutes high quality CP services, efforts to ensure or improve the quality of healthcare in this expanding sector may be hampered.

Objective: To identify the defining features of the quality of CP services and synthesise these into an evidence-based quality framework.

Method: International research evidence (2005 onwards) identified from six electronic databases (Embase, PubMed, Scopus, CINAHL, Web of Science, PsycINFO) was reviewed systematically. Search terms related to "community pharmacy" and "quality". Titles and abstracts were screened against inclusion/exclusion criteria followed by full-text screening by at least two authors. A narrative synthesis was undertaken. Following narrative synthesis, a patient and public involvement event was held to further refine the quality framework.

Results: Following title and abstract screening of 11,493 papers, a total of 81 studies (qualitative and quantitative) included. Of the 81 included studies, 43 investigated quality dimensions and/or factors influencing CP service quality; 21 studies assessed patient satisfaction with and/or preferences for CP; 17 studies reported development/assessment of quality indicators/standards/guidelines for CPs; which can help define quality.

The quality framework emerging from the global literature consisted of six dimensions: person-centred care, access, environment, safety, competence, and integration within local health care systems. Quality was defined as having timely and physical access to personalised care in a suitable environment, which is safe and effective with staff competent in the dispensing process, and pharmacy professionals' possessing clinical knowledge and diagnostic skills to assess and advise patients relative to pharmacists' increasingly clinical roles.

Conclusion: The emerging framework could be used to measure and improve the quality of CP services. Further research and feasibility testing are needed to validate the framework according to local healthcare context.

For peer review only

Strengths and limitations of this study:

This section should be no more than 5 bullet points relating specifically to the methods - not the results of the study. This will be published as a summary box after the abstract in the final published article.

- This review deployed a comprehensive and systematic search of the international literature which sought to identify defining features of quality of community pharmacy healthcare services and synthesise these into a quality framework.
- For data extraction, a two-step selection process was conducted: two authors (AMKH, SMC) screened all 11,493 papers independently of each other, and the two other authors (SJ, EIS) reviewed all papers with discrepancies and/or queries.
- To ensure relevance of findings to patients, members of the public who use community pharmacy services were consulted on the findings and their feedback was used to further refine the dimensions/sub-dimensions of the quality framework.
- Quality of the papers was not critically appraised.

Background

Faced with growing patient needs, workforce shortages and financial constraints, the necessity for healthcare systems worldwide to focus on delivering "high quality care" and meeting demand for primary care has never been greater with evidence of wide variation in quality between and within countries.[1 2] Health policy in the past few decades has focused on measuring and improving the quality and safety of healthcare services[3] as well as on improving the quality of care via a wider workforce approach (i.e. distribution of clinical responsibilities between professions) and local integration of health and social care globally[4]. The aim is to improve and strengthen a quality health and care system by joining up planning, commissioning and delivery of health and care services to provide seamless locally based integrated care that meets people's needs promptly and effectively.[3 5 6]

In relation to this, in the past two decades, policymakers have increased the range of healthcare services provided by community pharmacies (CP), over and above their more traditional medicines supply function, to relieve burden on general medical practice and expand capacity within primary care systems.[7] Community pharmacies are accessible and convenient, offering extended and weekend opening hours. Unlike other primary care providers, patients can access community pharmacies without the need for an appointment.

For peer review only

Hence, community pharmacies are well positioned to improve patient access to care and may assist in reaching patients in deprived areas.[8]

With a view to increasing patient access and choice, healthcare systems worldwide, most notably in countries such as the UK,[9 10] Canada,[11] United States,[12] Australia,[13] and New Zealand,[14] have invested in expanding the range of healthcare (i.e. medicines-related and public health) services offered by CP alongside the sale of over-the-counter (OTC) medicines and other items. However, the quality of some CP services, for example dispensing and medication review services, has been inconsistent.[15-17] Given the increasing range and volume of services provided by CP, it is important to consider how the quality of care can be improved and made equitably accessible. To be able to assess the quality of healthcare provided by CP, an agreed definition and framework are needed.[16]

Different definitions and frameworks of healthcare quality have emerged across healthcare over the years. One of the most influential models stems from Donabedian's structure-process-outcome framework (1980).[18] "Structure" involves the setting of care (e.g. physical facility, human resources, equipment), "process" encompasses the actions taken during service provision (e.g. diagnosis, treatment), and "outcome" is the results of actions taken (e.g. clinical changes to health, patient satisfaction). Donabedian proposed that structure, process, and outcomes are closely linked and influence each other, and his three components are the basis for many quality frameworks.[19-22]

In 2001, the US Institute of Medicine (IOM) developed a health care quality framework which involved six dimensions (i.e. safety, effectiveness, patient-centredness, timely, efficient, and equitable).[23] The IOM's framework has been widely recognised and since its inception different organisations have proposed quality frameworks which often use a combination of these six dimensions. Notably, the Organization for Economic Cooperation and Development (OECD) Health Care Quality Indicators Project (2006)[24] and Lord Darzi's Next Stage Review (2008)[25] defined quality under the three dimensions of safety, effectiveness and patient-centredness. More recently, similar to the IOM's quality framework but also acknowledging the importance of integration, the World Health Organization (WHO) Framework on Integrated People-centred Health Services (2018) described high quality care as care that is safe, effective, people-centred, timely, efficient, equitable and integrated.[3]

Since the early 2000s, definitions of quality in healthcare have been developed and continue to be refined. However, quality is still not well defined in CP,[15 26] with little known about what quality in CP means or how to measure it.[26] In 2012, Halsall et al. characterized healthcare quality in UK community pharmacy under three dimensions: "accessibility"; "effectiveness"; and "positive perceptions of the experience".[27] More recently, Watson et al. characterised

For peer review only

quality under dimensions of: person-centred; professionalism; and privacy.[28-30] A US based study looking at patients' understanding of what constitutes a "quality pharmacy" identified themes focusing on patient care and trust in pharmacists.[31] However, the dimensions of quality proposed in these studies were mainly related to pharmacists' more traditional role of medicines supply. Furthermore, these studies did not seek to develop a quality framework for CP health service provision as part of an integrated primary healthcare system. As the expansion of community pharmacy away from a primarily medicines supply role and into an extended range of professional services gathers pace,[32] there is a need to shed light on ways community pharmacies could work effectively with other primary care providers to provide better quality healthcare services.

Community pharmacy provides an exemplar of a (part) publicly funded private sector provider, in a mixed market healthcare system. Similar to community pharmacy, quality is poorly defined in other private sector primary care providers such as dentistry[22 33] and optometry.[34] As stated in the WHO report, *"For if quality of care is not ensured, what is the point of expanding access to care?".*[1] In line with the policy drive to increase patient choice and access to a wider range of services and service providers, it is important to develop a better understanding of quality in these sectors.[10 35]

> *"We cannot assess quality until we have decided with what meanings to invest the concept. A clear definition of quality is the foundation upon which everything is built". (Donabedian, 1985)*

The **aim of this study** is to identify the defining features of the quality of CP services and synthesise these into an evidence-based CP quality framework.

Methods

Search Strategy

Six electronic databases were searched (i.e. Embase, PubMed, Scopus, CINAHL, Web of Science, PsycINFO) using search terms relating to "community pharmacy" and "quality" **(Table 1)**. Specific search strategies for each database are provided in **Supplementary File 1**. Database searches were reviewed with The University of Manchester library's team. In addition, references of included studies were scanned for further relevant studies. The search strategy included studies published between 2005 and January 2023.

[INSERT TABLE 1 HERE]

For peer review only

Data screening

A two-step selection process was conducted by two reviewers (AH and SC) independently of each other (conventional double screening). Non-English papers were translated. Titles and abstracts were initially screened against the inclusion/exclusion criteria by AH and SC followed by subsequent full-text screening **(Table 2)**. During the double-screening process, two additional reviewers (SJ and ES) were consulted where there was discrepancy between AH and SC, and/or queries arose.

[INSERT TABLE 2 HERE]

Data extraction and synthesis of results

Data from included papers were extracted using NVivo as a data extraction grid. The process of synthesising the literature was iterative as follows. The first author (AH) initially catalogued the different dimensions/theoretical concepts of quality arising from the literature. Data relevant to quality of community pharmacy healthcare services generated from the literature was then categorised across these identified dimensions of quality. All authors independently assessed each dimension. Iterative revisions were made based on discussions between all authors.

A narrative synthesis was then undertaken by the first author, to provide a descriptive account of both qualitative and quantitative research evidence. Synthesis involved integrating and drawing on findings from studies that addressed: quality dimensions; factors influencing the quality of community pharmacy healthcare services; factors influencing integration of services with wider healthcare system. Synthesis also involved studies which developed quality indicators/standards for community pharmacy, and studies which assessed patient satisfaction with and/or preferences for community pharmacy, when they provide findings of relevance to the aim of the review. As the focus of this review was to synthesise findings into dimensions which are relevant to quality, findings emerging from the data from different methodological approaches were combined to contribute to an emerging quality framework.

Patient and Public Involvement

Following synthesis of findings, a patient and public involvement event was held in April 2023 with seven members of the public who use community pharmacies. The event gathered feedback on the dimensions/sub-dimensions of the quality framework emerging from the review. The feedback provided was used to further refine the dimensions/sub-dimensions of the quality framework.

For peer review only

Results

Study selection

A total of 11,493 papers were identified for initial screening after duplicates had been removed. Following title and abstract screening, 165 papers were assessed for eligibility via full-text reading, with 74 studies included in the review. Manual searching of reference lists identified 7 additional studies after eligibility screening **(Figure 1).**

[INSERT FIGURE 1]

For peer review only

Definition of pharmacy services

Multiple terms were used in the literature to describe aspects of community pharmacy practice and healthcare service provision. For consistency, we have broken down community pharmacy healthcare services into 1) medicines supply 2) professional pharmacy services **(Table 3)**.

[INSERT TABLE 3]

Some medicines are available to buy without a prescription, commonly referred to as **over the counter (OTC) medicines**. Data from studies which focused on sale/ supply (be that on prescription or in response to a request for sale) of OTC medicines were grouped under "medicines supply". Data from studies which looked at sale/ supply of OTC medicines involving professional/clinical judgement, e.g. as part of a service, were included under "professional pharmacy service".

Study characteristics

Of the 81 studies included in the review, 43 investigated quality dimensions and/or factors influencing the quality of community pharmacy services.[15 26-30 39-75] Twenty-one studies assessed patient satisfaction with and/or preferences for community pharmacy.[76-95] Thirteen studies reported development/assessment of quality indicators for community pharmacies.[96-108] Four studies described and defined standards or guidelines for good pharmacy practice which can be used to help define quality.[109-112]

Multiple methods were used including: surveys (n=43);[26 39 42 44 45 48 56 58 60 62 64-66 72 74 76 77 79 80 82-93 96 97 99 103-108 111-113] qualitative interviews (n=9);[15 30 43 47 59 67 68 71 81] focus groups;[27 51 61 75] pre- and post-measurement of adherence to standards;[110] biographic and photographic techniques;[40] participant observations;[49] nominal group technique;[41] applying indicators in practice;[100 101] Q-methdology[94 95] stakeholder event;[109] deductive content analysis;[69] patient stories[63] and mixed methods (n=14);[28 29 46 50 52-55 57 70 73 78 98 102]

Most of the studies were from UK (n=15),[15 26-30 40 52 54 63 75 88 97 103 108] USA (n=11),[39 45 49 51 62 64 66 70 79 86 113] and Australia (n=7).[43 44 47 50 57 81 110] Of the remaining studies, four each were from: Japan,[55 59 60 77] the Netherlands,[53 99-101] Thailand,[65 67 93 102] three each from Germany,[56 73 105], Estonia,[69 76 112] Iran,[46 74 82] Vietnam,[91 94 95] two each were from Lebanon ,[109 111] UAE ,[58 84] Brazil,[104 107] and Spain.[61 71] One each were from Canada,[72] Finland,[96] New Zealand, One each from Canada,[72] Finland,[96] New Zealand,[41] Lithuania,[42] Malaysia,[85] Poland,[89] Slovenia,[78]

For peer review only

Serbia,[68] Sudan,[92] Nigeria,[106] Iraq,[90] Pakistan,[87] and China.[83] One study involved five European countries (Denmark, Germany, Netherlands, Poland, Great Britain) to validate a pan-European questionnaire.[48] One study was conducted amongst three African countries: Ethiopia, Uganda, and Zimbabwe,[98] and another compared questionnaire findings between community pharmacy users in Poland and UK.[80]

Most of the literature explored the views/expectations of community pharmacy staff[15 26 27 29 40 42-48 52-54 58 59 62 64 66 67 75-78 96 99 100 103-108 112] and patients.[26-28 39 45 46 50 51 53 54 56 57 60 61 63 65 67 68 75-85 87-95 103 113] GPs' views on quality in community pharmacy were explored in seven studies.[41 46 52 55 75 78 103] The views of pharmacy organizations and primary healthcare funders and policy makers were explored in just seven studies.[15 27 29 30 41 54 105 111] Five studies which developed quality indicators explored the views of pharmacy academics.[96 97 99 105 107] Summary of study characteristics are provided in **Supplementary File 2.**

Quality framework

Data relevant to identifying concepts and dimensions of quality of care for CP identified from the literature were synthesised and themed under six dimensions (person-centred care, access, environment, competence, safety, integration) to develop a quality framework **(Figure 2)**. The narrative synthesis below is themed under these six dimensions.

[INSERT FIGURE 2]

For peer review only

Access: structural and procedural components of quality such as opening hours, waiting time, physical access, availability of medicines and availability of pharmacy staff to provide services

Opening hours

Availability of pharmacy services during stated and extended opening hours are commonly identified as key features of quality in CP. [46 56 60 65 68 77 87 88 94 95 107 113] Patients, pharmacists and GPs suggest that CPs should aim to offer extended opening hours outside regular hours.[27 28 30 51 75]

Waiting time

Minimal waiting time for pharmacy services (particularly for picking up medicines dispensed on prescriptions) is commonly cited as an important procedural feature of quality of care in CP .[27 46 63 94 95] Studies exploring the views of patients on quality of care in CP suggest that pharmacies should aim to minimise wait times to get medicines dispensed.[63 68]

Physical access

Five studies describe “parking space near the pharmacy” as a feature of quality in community pharmacy.[28 68 76 78 87] Three studies highlight the importance of CPs being accessible for people with special needs such as the elderly, visually impaired, people with baby carriages.[68 78 107] Ease of access of community pharmacies via public transportation,[56 83] work/home,[51 94] and other healthcare facilities are important features of quality as perceived by patients.[68 77]

Availability of pharmacy staff

Having adequate numbers and appropriately qualified pharmacy staff is described as a hallmark characteristic of a quality CP.[51 57 68 87 90 102 113] Studies commonly measure the availability of a pharmacist (on-site) to provide advice and answer medication-relates queries.[87 90 102 112] The availability of pharmacy staff on the phone is addressed in two studies.[60 68]

Availability of medicines

Studies in this review indicate that pharmacies should hold an adequate, well managed stock of medicines as well as medical devices.[59 77 109] Studies also emphasise on pharmacies having a stock management system that helps control stock orders and expiry dates and using contingency plans for purchases in an emergency.[77 100 102 106 111] Furthermore, community pharmacies should have available records for expired drugs, as well as having specific procedures for disposal of expired products.[93 98 100 106 111]

For peer review only

Patients, pharmacists and GPs highlight the importance of pharmacies maintaining adequate stock and/or being able to obtain medicines quickly, to avoid patients having to return. [26 30 57] Patients also perceive reasonable/affordable cost of medications and notification of discounts as an important determinant of CP service quality.[51 56 68 81 83 86 87 89 91 95] Patients expect pharmacists to provide them with information about alternative medicines and their prices.[94 95]

Environment: the impact of facilities, equipment, and pharmacy layout on the quality of healthcare service provision

Appearance of the pharmacy

The appearance of the community pharmacy is an important structural feature of quality health service provision. Studies suggest that community pharmacies need to appear health service orientated by clearly displaying medicines and informational material (such as adverts, leaflets).[78 79 93] The pharmacy should also be positioned in a manner which is visible and accessible to patients with clearly defined boundaries. In supermarkets, it should be clear where the general shop or supermarket ends and the pharmacy begins.[40]

Studies also highlight that every pharmacy should have sufficient counters for dispensing medicines[95] and adequate physical space for pharmacy staff to provide professional services (health promotion, education, consultation or screening services to individuals or groups).[40 102] It is also important to ensure that premises are tidy[46] and lightning of the pharmacy is well distributed.[58]

Cleanliness and hygiene of the pharmacy is commonly highlighted as a feature of quality of care.[57 83 87 89 91 95 102 109] A few studies specifically mention “ensuring room/air temperature is appropriate”[58 67] and “avoidance of unpleasant smells[28 57 67 78] ” as a means to promote a good first impression of the pharmacy.

Waiting area

Studies suggest that a good quality pharmacy should ensure that the waiting area has sufficient space and seating.[52 61 76-78 82 95 112] The importance of informing patients of waiting times and the reasons for any delays was addressed in one study.[52]

Dispensary

Studies suggest that the dispensary should be well organised and spacious designed to ensure efficient processing of prescriptions.[40] Storage shelves/drawers should be clearly labelled with drug classifications and medicines are kept according to the drug classifications.[102] Pharmacies are required to have a system in place to prevent unauthorized access into areas where controlled drugs are stored.[102 110]

For peer review only

Physical resources (equipment)

Studies highlight the importance of having drug information systems and resources to ensure provision of high quality services.[27 46 55 56 65 109 110] Only two studies specifically mention resources needed to provide professional pharmacy services, such as scales, digital blood pressure monitoring equipment, finger tip sugar equipment,.[93 102]

Private consultation area

Having a private area for consultations is perceived to be a key facilitator for overcoming privacy issues.[28 30 40 41 50 51 58 61 75 87 88 91 93-95 110-112] Pharmacies without a designated consultation room increase the risk of patient conversations being overheard.[30 50 58 88 94 95] Pharmacies in countries such as the UK are required to have at least one dedicated consultation room and it is noted that pharmacists should be proactive in offering it to patients.[30 40 75] Relative to pharmacy size, where possible, the room should be spacious, ensuring it is clutter-free and gives the impression of a professional consultation room.[40 75]

Competence: of pharmacy staff in the dispensing process, pharmacy professionals' clinical knowledge and diagnostic skills to assess and refer patients

Competence in the dispensing process

Pharmacists' ability, knowledge and expertise (i.e. competence) to deliver counselling on prescription medicines is often used to describe quality of health service delivery in community pharmacy. [27 29 30 41 46 55 57 59 65 67 79 90 91 93 95 101 106 107 109 110 113] Patients and community pharmacists suggest that providing high quality care requires pharmacists having knowledge and skills to dispense the most effective medicines and provide accurate, clear, and complete information for a specific medicine. [15 28 30 59 67] Studies also commonly mention speed of dispensing,[87 106] accuracy of dispensing,[68 82 84 87 98 100-102 111] and gathering essential patient information as elements of an effective dispensing process.[53 87 93 100-102 111]

Clinical knowledge and diagnostic skills

Only four studies (three of which looked at OTC consultations and one at home care supply) describe competence as knowledge and skills which extends beyond traditional dispensing and medicine supply and are particularly relevant for pharmacists' increasingly clinical roles and professional pharmacy services. These studies emphasise on the need for pharmacists to have knowledge in specific disease areas[59] and diagnostic skills to provide effective treatment options with correct instructions for medicine usage and storage.[30 55 67] Moreover, GPs expect pharmacists providing professional services to be competent to assess and refer patients to a GP or other health care provider if necessary.[30 41]

For peer review only

Some studies highlight pharmacy staff needing more opportunities to enhance clinical knowledge via participation in training programs, CPD courses and/or seminars.[15 44 77 102 106 109 112] Making use of all the skill sets of employees (i.e. skill mix) was suggested as important for improving the quantity and quality of professional services in community pharmacy.[15 41 75] Upskilling pharmacy technicians to free up pharmacists to move from medicine supply to professional pharmacy services was suggested in one study.[62]

Person-centred care: pharmacy staff providing patients with a positive patient experience; establishing a patient-pharmacist relationship; and demonstrating professionalism at all times

Patient experience

Many studies identified in this review highlight the importance of a positive patient experience when looking at quality of care in CP. A positive patient experience is often described by patients as pharmacists taking the time to understand patients' individual needs and involving patients in decisions around their medications.[15 30 39 41 42 51 60 65 68 75 91 94 95 103 107] This includes tailoring delivery of services to people with special needs or minority groups[51 68 107] for example by *"adjusting the tone of voice when addressing patients with hearing difficulty"* or *"using capital letters on written materials if the patient has vision problems"*.[68] Patients, pharmacists and GPs perceived sole trader (independent) CPs to provide more personalised care compared to pharmacy chains due to greater pharmacist autonomy in the former. [15 30 62 89]

Professionalism

Professionalism shown by pharmacy staff was perceived by patients as a hallmark feature of good quality service provision. Professionalism encompassed attributes such as courtesy, empathy and trustworthiness.[28 46 51 55 56 59 63 65 68 78 79 81 88-91 95 106 110] Studies suggest that patients expect pharmacy staff to treat them with courtesy and respect and spend as much time as necessary during each encounter.[51 55 63 68 78 79 81 88-90 95 110] On the other hand, patients perceive a lack of empathy shown by pharmacy staff to reduce service quality.[51 68] Patients valued pharmacists expressing honest opinions regarding patient benefit as a high priority.[56 65 106] In terms of professional appearance, two studies suggest that pharmacists should be distinguishable from the rest of the staff for example by wearing a name badge with their role.[28 102]

Patient-pharmacist relationship

Studies investigating the views of patients, pharmacists and GPs on community pharmacy frequently cite the patient-pharmacist relationship as an important feature of service quality. Trust, friendliness/helpfulness and availability of the pharmacist have been found to influence

For peer review only

the quality of the patient-pharmacist relationship as perceived by patients.[29 39 51 55 57 61 65 75 81] Continuity of care (i.e. patients seeing the same pharmacist over time), is perceived to facilitate development of trust and rapport between patients and pharmacists.[26 29 30 81]

Safety: identifying errors and intervening; accuracy in dispensing and compounding; adequate information sharing between pharmacy staff when exchanging shifts; and having systems for ensuring safety

Compounding

Studies suggest labelling of compounded preparations (i.e. preparation of a custom medication) with detailed instructions and clear expiry dates,[68 109] as well as availability of standard operating procedure (SOPs) to ensure accuracy in compounding.[99-101]

Dispensing

Studies commonly mention ensuring accuracy of dispensing so errors are prevented.[68 82 84 87 98 100-102 111] Identifying and resolving dispensing errors is also seen a key characteristic of good quality health service provision in community pharmacy. This requires pharmacies having clear standard operating procedure (SOPs) for checking prescriptions and dispensing medications (particularly high-risk medications).[100 101 103] Studies also suggest having protocols and guidelines for asking patients about potential drug contraindications and drug-drug interactions. [99-101 106]

Systems for ensuring safety

Recording prescription data and patient information on computer systems to avoid errors and safety incidents are mentioned in included papers.[98 101 107] The literature also suggests that pharmacies should have an internal quality and safety management system in place for: registering errors made during dispensing, evaluating patient experiences, and recording the number of patient complaints.[99-101 106] Three studies also highlight the importance of investigating and learning from incidents, education and training about safety, staffing, and management commitment to patient safety. [41 45 48]

Documentation of care

Studies looking at documentation of patient care focus on accurate recording of relevant information such as medical history and medication [30 59 65 109-111] in a way that can be read and interpreted by other healthcare professionals.[107] Furthermore, these studies measure whether patients' personal information is stored and disposed of in a confidential manner.[58 59 86]

One study measured handovers defined as "exchange of information, responsibility, and accountability when a pharmacist concludes a shift and another replaces them at the

For peer review only

beginning of a new shift within the same pharmacy".[64] The study identified that in almost half of the time, handoffs that occur in a community pharmacy setting were inaccurate or incomplete.[64]

Integration: ways for CP to establish and sustain relationships with the wider healthcare team by having interprofessional collaboration, communication mechanisms and information systems.

Interprofessional collaboration

The ability of community pharmacists to establish a relationship with the local GP was perceived as a fundamental part of community pharmacy integration with the wider healthcare system.[15 52 70 71 75] Building a relationship required a shared understanding of competences, roles and responsibilities.[72 73 75] The perceived benefit of having closer CP-GP working relationships was improved communication, effective signposting and prompt resolution of prescription isuses,[15] handling near-misses and dispensing errors, and ensuring errors and near misses are recorded and disused regularly.[100 101 103]

Communication mechanisms and information systems

GPs' and community pharmacists' preference for communication methods (e.g. telephone, face-to-face) has been explored but findings are inconclusive.[72 74] One study highlights that pharmacists express preference for predefined and clear ways to communicate with GPs, given difficulties getting GPs on the phone and receiving an answer to their query.[73] Having a lead responsible for linking GP and CPs is suggested in one study as a potential way to facilitate CP-GP collaboration.[71]

Whether the community pharmacy should have not only read but also write access to shared medical records has been debated. This would allow pharmacists to view relevant information about a patient's medical history to inform their assessment and clinical judgement, enable them to add prescription and medical/ intervention details in the patient's medical record, so doctors and the wider general practice team are aware.[30 55 71 72 74 75 86 88] Pharmacists in some studies argue they require better access to patient information to provide safe and effective health care services.[72 74 75 86] Equally, in the UK, patients and GPs have raised concerns over read/write access to medical records, considering the sharing of patient information with commercial organisations, with limited control over who has access, as problematic.[30 72 75]

Three Commonwealth studies highlight the importance of having shared communication systems between community pharmacy and the rest of the healthcare system to facilitate community pharmacy integration.[41 63 75] In one of these studies, GPs argue that it is difficult to

For peer review only

refer patients to community pharmacy given that interactions at community pharmacy are not documented or communicated to them.[75]

Patient and Public Involvement

When members of the public were presented with findings and asked for input on dimensions/sub-dimensions of the quality framework emerging from the review, most were dissatisfied with waiting times at CP to collect their medicines. There were tensions around the only pharmacist on site not being accessible to patients.

In addition, the CP retail environment was a perceived barrier to good quality service provision mainly due to privacy issues (e.g. asking details such as address, date of birth in front of customers). All members highlighted the importance of CP staff being professional and distinguishable by wearing a name badge with their role.

Furthermore, integration was seen as a key element of quality where members described the lack of collaboration/communication between GPs and pharmacists. Lastly, members of the public mentioned that CPs are unaware when patients are directed towards them by GPs and vice-versa. This input from PPI group was used to further refine the dimensions/sub-dimensions of the quality framework **(Supplementary file 3)**.

Definition of quality of care in community pharmacy

Based on findings in this review, quality of care in CP can be defined as having timely and physical access to person-centred professional services in a suitable environment, which is safe, integrated and effective. **Supplementary file 4** summarises key dimensions in this review linked to Donabedian's structure-process-outcome components.

Discussion

In the absence of a universally agreed quality framework looking at health service provision in community pharmacy, this review aimed to collate and synthesise concepts explored in the literature which are relevant to defining quality of care in CP. Upon synthesising the findings of 81 papers, quality was conceptualised by the inter-related dimensions of person-centred care, access, environment, competence, safety, and integration.

The dimensions of quality identified in this review resonate with the IOM's six dimensions of quality,[23] OECD's proposed definition of quality,[24] and the WHO Framework on Integrated People-centred Health Services.[114] The dimensions common to all frameworks were person centeredness, effectiveness, access, and safety. In line with the WHO framework, the framework developed here for quality in CP also included an integration dimension, the

For peer review only

importance and relevance of which for community pharmacy is discussed below.[3 115] Unlike these other frameworks, however, "environment" was conceptualised as a separate dimension. Lack of privacy in community pharmacy was commonly highlighted by this review as a barrier to providing high quality healthcare services. The "shop" appearance of community pharmacies and whether premises are fit-for-purpose may prohibit some CPs from meeting all aspects of the framework.[40] One way of being able to ensure privacy when appropriate (e.g for professional services) is having a dedicated consultation area with adequate space.[116]

This review, which adopts a broad view of features of quality of care in CP, draws out important considerations for defining quality community pharmacy to ensure high quality patient care, experience and outcomes. To begin with, CP is one of the most accessible settings in which to receive healthcare services.[17] However, geography alone does not guarantee patients will receive the healthcare services they need. Corroborating findings from this review, previous literature reviews suggest that improving access further involves having adequate staffing levels, strategies for managing medicines supply as well as shortages, and efficient workflow procedures to reduce waiting times.[8 117-119]

The responsiveness of health systems to the needs of the population is a central pillar of healthcare quality and a crucial perspective is through patients' evaluations of the care they receive.[120] In line with findings from the wider primary and secondary care literature,[121 122] the person-centred care dimension in this review highlights a positive patient experience, good patient-pharmacist relationship, relational continuity of care, and professionalism as key attributes of quality from a patient perspective. A systematic review looking at a wide range of primary and secondary care settings found that patient experience is positively associated with clinical effectiveness and safety.[121] Moving forward, quality initiatives in CP need to prioritise collecting patient feedback with emphasis on organisations using that data as one aspect of ongoing quality improvement.

In this review, the competence dimension mainly covered pharmacy staff's ability to effectively perform the dispensing procedure, with dispensing remaining a significant part of community pharmacies, even where (funded) professional services are emerging. Although many studies did not cover professional services, much of the medicine supply process is now expected be performed by the pharmacy support team, and important part of freeing pharmacists' time for professional services. As the scope of professional community pharmacy services continues to expand in many countries, more research is needed to develop quality indicators which consider pharmacy professionals' clinical knowledge and diagnostic skills for providing professional (clinical and public health) services.

For peer review only

The dimensions of access, person-centred care, competence, and environment mirrored those of existing CP frameworks by Halsall[27] and Watson.[28-30] However, compared to previous studies conceptualising quality in CP, the "integration" dimension was unique in our framework. Six studies synthesised in this review and PPI members describe community pharmacy integration within the wider healthcare system as an important dimension of a quality framework. Our study suggests that an integration dimension needs to consider interprofessional collaborations and information sharing between community pharmacy and other primary care providers such as general practice. The 'interprofessional collaboration' element of our integration dimension resembles Valentijin's taxonomy of integrated primary care[123] where the term 'professional integration' is used to describe "*inter-professional partnerships based on shared competences, roles, responsibilities and accountability to deliver a comprehensive continuum of care to a defined population*". The communication mechanisms and information systems of our integration dimension closely aligns with Valentijin's 'functional integration', defined as "*key support functions and activities (i.e. financial, management and information systems) structured around the primary process of service delivery, to coordinate and support accountability and decision making between organisations and professionals to add overall value to the system".*[123]

To the authors' knowledge, this is the first systematic review of the international literature which sought to identify defining features of the quality of community pharmacy healthcare services and synthesise these into a quality framework. The framework emerging from this review contributes to knowledge of improving access to, and the healthcare of, the population through privately owned businesses which provide publicly funded primary healthcare services. The strength of this paper is the comprehensive and systematic search of the international literature deployed by the lead author (AH) with conventional double screening by an expert in quality of care (SC). Furthermore, an expert in community pharmacy policy research (ES) reviewed all papers at the full-text review stage where there were disagreements/ uncertainty between AH and SC. Another expert in community pharmacy policy research (SJ) undertook this process on all papers where discrepancies remained. Moreover, input from public contributors was used to further refine the dimensions/sub-dimensions of the quality framework. In terms of limitations, included papers were not critically appraised due to considerably varying methodologies and findings. Given this review sought to develop a broad framework covering different dimensions of healthcare quality, the word "integration" was not used as a key word in the search strategy which could explain the low number of papers identified relative to integration.

For peer review only - http://bmjopen.bmj.com/site/about/guidelines.xhtml

Conclusion

This review defines quality of CP and provides a dimensional framework of quality of CP services consisting of six dimensions: patient experience, access, environment, safety, competence, integration. As CP expands in the UK and other countries beyond a primarily medicines supply function, the quality dimensions need to be validated and refined locally with a particular emphasis on integration. Integration is particularly relevant for professional services, where roles and responsibilities for joined-up services are shared across primary care providers, making collaboration and two-directional information sharing particularly important. Once quality dimensions are validated and refined, the next step will be using the framework to develop and feasibility test summative "quality assurance" and formative "quality improvement" mechanisms.

Funding statement: This work was funded by The National Institute for Health and Care Research (NIHR) School of Primary Care (Grant reference:**C066)**.

Competing interest: None.

Acknowledgements: We would like to thank The National Institute for Health and Care Research (NIHR) School of Primary Care for funding the fellowship We would also like to thank our patient and public contributors for providing their input on the quality framework emerging from this systematic review.

Contributors: AH, ES, SC and SJ conceptualised the study.

AH ran database searches, title and abstract screening. AH and SC undertook independent full-text review. ES reviewed all papers at the full-text review stage, where there were disagreements/ uncertainty between AH and SC; SJ undertook this process on all papers where discrepancies remained. All authors discussed and agreed inclusion and exclusion criteria, and judgements on all papers at the full-text review stage, to reach a final decision.

AH ran the data extraction process which was refined by SC, SJ and ES. AH facilitated the patient and public involvement event, which SJ co-facilitated.

AH wrote the first full draft of the manuscript. All co-authors reviewed and discussed drafts iteratively, providing critical and intellectual contributions to analysis, interpretation, and framing.

Ethics Approval: Not required

Patient consent form: As formal consent form is not required for PPI events, PPI contributors were informed of the purpose of the study and their contribution, and their consent was confirmed verbally.

For peer review only

Data sharing statement: Data are available on reasonable request, by contacting the corresponding author.

Registration and protocol: The review was not registered.

References

1. World Health Organization. Delivering quality health services: a global imperative for universal health coverage. 2019. Available: https://www.who.int/publications/i/item/9789241513906
2. Schneider EC, Shah A, Doty MM, et al. Mirror, Mirror 2021 — Reflecting Poorly: Health Care in the U.S. Compared to Other High-Income Countries: The Commonwealth Fund, 2021. Available: https://www.commonwealthfund.org/sites/default/files/2021-08/Schneider_Mirror_Mirror_2021.pdf
3. World Health Organization. Handbook for national quality policy and strategy: A practical approach for developing policy and strategy to improve quality of care. 2018. https://www.who.int/publications-detail-redirect/9789241565561
4. World Health O. Global strategy on human resources for health: workforce 2030. Geneva: World Health Organization. Available from: https://apps.who.int/iris/bitstream/handle/10665/250368/9789241511131-eng.pdf
5. Department of Health & Social Care. Joining up care for people, places and populations: The government's proposals for health and care integration, 2022. Available from: https://www.gov.uk/government/publications/health-and-social-care-integration-joining-up-care-for-people-places-and-populations/health-and-social-care-integration-joining-up-care-for-people-places-and-populations
6. World Health Organization. MONITORING THE BUILDING BLOCKS OF HEALTH SYSTEMS: A HANDBOOK OF INDICATORS AND THEIR MEASUREMENT STRATEGIES. 2010. Available from: https://apps.who.int/iris/bitstream/handle/10665/258734/9789241564052-eng.pdf
7. The Pharmaceutical Group of the European Union. PHARMACY 2030: A Vision for Community Pharmacy in Europe. Available from: https://www.pgeu.eu/wp-content/uploads/2019/04/Pharmacy-2030_-A-Vision-for-Community-Pharmacy-in-Europe.pdf.
8. Pantasri T. Expanded roles of community pharmacists in COVID-19: A scoping literature review. *J Am Pharm* 2022;62:649-57. doi: https://doi.org/10.1016/j.japh.2021.12.013
9. PSNC. Community Pharmacy: FORWARD VIEW. 2016. Available from: https://cpe.org.uk/wp-content/uploads/2016/08/CPFV-Aug-2016.pdf
10. GOV UK. New pharmacy referral service to help patients avoid hospital readmission 2020. Available from: https://www.gov.uk/government/news/new-pharmacy-referral-service-to-help-patients-avoid-hospital-readmission.
11. Raiche T, Pammett R, Dattani S, et al. Community pharmacists' evolving role in Canadian primary health care: a vision of harmonization in a patchwork system. *Pharm Pract (Granada)* 2020;18:2171. doi: 10.18549/PharmPract.2020.4.2171

12. Salgado TM, Rosenthal MM, Coe AB, et al. Primary healthcare policy and vision for community pharmacy and pharmacists in the United States. *Pharm Pract (Granada)* 2020;18:2160. doi: 10.18549/PharmPract.2020.3.2160

13. Dineen-Griffin S, Benrimoj SI, Garcia-Cardenas V. Primary health care policy and vision for community pharmacy and pharmacists in Australia. *Pharm Pract (Granada)* 2020;18:1967. doi: 10.18549/PharmPract.2020.2.1967

14. Smith AJ, Scahill SL, Harrison J, et al. Service provision in the wake of a new funding model for community pharmacy. *BMC Health Serv Res* 2018;18:307. doi: 10.1186/s12913-018-3120-z

15. Jacobs S, Fegan T, Bradley F, et al. How do organisational configuration and context influence the quantity and quality of NHS services provided by English community pharmacies? A qualitative investigation. *PLOS ONE* 2018;13:e0204304. doi: 10.1371/journal.pone.0204304

16. Hann M, Schafheutle EI, Bradley F, et al. Organisational and extraorganisational determinants of volume of service delivery by English community pharmacies: a cross-sectional survey and secondary data analysis. *BMJ OPEN* 2017;7. doi: 10.1136/bmjopen-2017-017843

17. Valliant SN, Burbage SC, Pathak S, et al. Pharmacists as accessible health care providers: quantifying the opportunity. *J Manag Care Spec Pharm* 2022;28:85-90. doi: 10.18553/jmcp.2022.28.1.85

18. Donabedian A. The Definition of Quality and Approaches to Its Assessment. Ann Arbor: Health Administration Press 1980.

19. Campbell SM, Roland MO, Buetow SA. Defining quality of care. *Soc Sci Med* 2000;51:1611-25. doi: 10.1016/s0277-9536(00)00057-5

20. Santana MJ, Manalili K, Jolley RJ, et al. How to practice person-centred care: A conceptual framework. *Health Expect* 2018;21:429-40. doi: 10.1111/hex.12640

21. Lau P, Ryan S, Abbott P, et al. Protocol for a Delphi consensus study to select indicators of high-quality general practice to achieve Quality Equity and Systems Transformation in Primary Health Care (QUEST-PHC) in Australia. *PLoS One* 2022;17:e0268096. doi: 10.1371/journal.pone.0268096

22. Campbell S, Tickle M. What is quality primary dental care? *Br Dent J* 2013;215:135-9. doi: 10.1038/sj.bdj.2013.740

23. Institute of Medicine Committee on Quality of Health Care in A. Crossing the Quality Chasm: A New Health System for the 21st Century. Crossing the Quality Chasm: A New Health System for the 21st Century. Washington (DC): National Academies Press (US)

24. Arah OA, Westert GP, Hurst J, et al. A conceptual framework for the OECD Health Care Quality Indicators Project. *Int J Qual Health Care*2006;18(suppl_1):5-13. doi: 10.1093/intqhc/mzl024

25. Darzi A. High Quality Care For All: NHS Next Stage Review Final Report, 2008. Available from: https://assets.publishing.service.gov.uk/government/uploads/system/uploads/attachment_data/file/228836/7432.pdf

26. Jacobs S, Hann M, Bradley F, et al. Organisational factors associated with safety climate, patient satisfaction and self-reported medicines adherence in community pharmacies. *Res Social Adm Pharm* 2020;16:895-903. doi: https://dx.doi.org/10.1016/j.sapharm.2019.09.058

27. Halsall D, Noyce PR, Ashcroft DM. Characterizing healthcare quality in the community pharmacy setting: Insights from a focus group study. *Res Social Adm Pharm* 2012;8:360-70. doi: https://dx.doi.org/10.1016/j.sapharm.2011.09.001

For peer review only

28. Watson MC, Silver K, Watkins R. How does the public conceptualise the quality of care and its measurement in community pharmacies in the UK: A qualitative interview study. *BMJ Open* 2019;9:e027198. doi: https://dx.doi.org/10.1136/bmjopen-2018-027198

29. Watson MC, Skea ZC. Jugglers and tightrope walkers: The challenge of delivering quality community pharmacy services. *PLOS ONE* 2018;13:e0200610. doi: 10.1371/journal.pone.0200610

30. Watson M, Silver K, Watkins R. "What counts can't always be measured": a qualitative exploration of general practitioners' conceptualisation of quality for community pharmacy services. *BMC family practice* 2020;21:244. doi: https://dx.doi.org/10.1186/s12875-020-01319-2

31. Warholak TL, Patel M, Rosenthal M, et al. Patient perceptions of a pharmacy star rating model. *J Am Pharm* 2017;57:311-17. doi: https://doi.org/10.1016/j.japh.2017.01.011

32. Piquer-Martinez C, Urionagüena A, Benrimoj SI, et al. Integration of community pharmacy in primary health care: The challenge. *Res Social Adm Pharm* 2022;18:3444-47. doi: https://doi.org/10.1016/j.sapharm.2021.12.005

33. Byrne MJ, Tickle M, Glenny AM, et al. A systematic review of quality measures used in primary care dentistry. *Int Dent J* 2019;69:252-64. doi: 10.1111/idj.12453

34. National Academies of Sciences E, Medicine, Health, et al. The National Academies Collection: Reports funded by National Institutes of Health. In: Welp A, Woodbury RB, McCoy MA, et al., eds. Making Eye Health a Population Health Imperative: Vision for Tomorrow. Washington (DC): National Academies Press (US)

35. Royal College of General Practitioners. Fit for the Future: A vision for general practice. 2019. Available from: https://www.rcgp.org.uk/getmedia/ff0f6ea4-bce1-4d4e-befc-d8337db06d0e/RCGP-fit-for-the-future-report-may-2019.pdf

36. PSNC. NHS Community Pharmacy Contractual Framework Essential Service – Dispensing. Available from: https://psnc.org.uk/wp-content/uploads/2013/07/service20spec20es12020dispensing20_v1201020oct2004_.pdf.

37. Paulino E, Thomas D, Lee SWH, et al. Chapter 8 - Dispensing Process, Medication Reconciliation, Patient Counseling, and Medication Adherence. In: Thomas D, ed. Clinical Pharmacy Education, Practice and Research: Elsevier 2019:109-20.

38. Moullin JC, Sabater-Hernández D, Fernandez-Llimos F, et al. Defining professional pharmacy services in community pharmacy. *Res Social Adm Pharm* 2013;9:989-95. doi: https://doi.org/10.1016/j.sapharm.2013.02.005

39. Worley MM. Testing a pharmacist-patient relationship quality model among older persons with diabetes. *Res Social Adm Pharm* 2006;2:1-21. doi: 10.1016/j.sapharm.2005.12.006

40. Rapport F, Doel MA, Jerzembek GS. "Convenient space" or "a tight squeeze": insider views on the community pharmacy. *Health & place* 2009;15:315-22. doi: 10.1016/j.healthplace.2008.06.002

41. Scahill SL, Harrison J, Carswell P. What constitutes an effective community pharmacy?--development of a preliminary model of organizational effectiveness through concept mapping with multiple stakeholders. *Int J Qual Health Care* 2010;22:324-32. doi: 10.1093/intqhc/mzq033

42. Urbonas G, Jakušovaitė I, Savickas A. Pharmacy specialists' attitudes toward pharmaceutical service quality at community pharmacies. *Medicina (Kaunas)* 2010;46(10):686-92. [published Online First: 2010/01/01]

For peer review only

43. White L, Clark C. Stakeholders' views of service quality in community pharmacy: a qualitative study. *Health Mark Q* 2010;27:48-65. doi: 10.1080/07359680903519750

44. Feletto E, Wilson LK, Roberts AS, et al. Building capacity to implement cognitive pharmaceutical services: Quantifying the needs of community pharmacies. *Res Social Adm Pharm* 2010;6:163-73. doi: 10.1016/j.sapharm.2009.08.003

45. AHRQ. Community Pharmacy Survey on Patient Safety Culture, 2012. Available at: www.ahrq.gov/qual/patientsafetyculture/pharmsurvindex.htm.

46. Dadfar H, Brege S. Differentiation by improving quality of services at the last touch point. I*nt J Qual Serv* 2012;4(4):345-63. doi: 10.1108/17566691211288331

47. White L, Klinner C. Service quality in community pharmacy: an exploration of determinants. *Res Social Adm Pharm* 2012;8:122-32. doi: 10.1016/j.sapharm.2011.01.002

48. Phipps DL, De Bie J, Herborg H, et al. Evaluation of the Pharmacy Safety Climate Questionnaire in European community pharmacies. *Int J Qual Health Care* 2012;24:16-22. doi: https://dx.doi.org/10.1093/intqhc/mzr070

49. Odukoya OK, Chui MA, Pu J. Factors influencing quality of patient interaction at community pharmacy drive-through and walk-in counselling areas. *Int J Pharm Pract* 2014;22:246-56. doi: 10.1111/ijpp.12073

50. Hattingh HL, Knox K, Fejzic J, et al. Privacy and confidentiality: perspectives of mental health consumers and carers in pharmacy settings. *Int J Pharm Pract* 2015;23:52-60. doi: 10.1111/ijpp.12114

51. Shiyanbola OO, Mott DA, Croes KD. The structural and process aspects of pharmacy quality: older adults' perceptions. *Int J Clin Pharm* 2016;38:96-106.

52. Weiss MC, Grey E, Harris M, et al. Dispensing doctor practices and community pharmacies: exploring the quality of pharmaceutical services. *Prim Health Care Res Dev* 2016;17:42-55. doi: 10.1017/s1463423615000092

53. Koster ES, Blom L, Overbeeke MR, et al. Quality of pharmaceutical care at the pharmacy counter: patients' experiences versus video observation. *Patient Prefer Adherence* 2016;10:363-9. doi: 10.2147/ppa.S102032

54. Newlands RS, Power A, Young L, et al. Quality improvement of community pharmacy services: a prioritisation exercise. *Int J Pharm Pract* 2018;26:39-48. doi: 10.1111/ijpp.12354

55. Fujita K, Kushida K, Moles RJ, et al. Home healthcare professionals' perspectives on quality dimensions for home pharmaceutical care in Japan. *Geriatr Gerontol Int* 2019;19:35-43. doi: 10.1111/ggi.13562

56. Guhl D, Blankart KE, Stargardt T. Service quality and perceived customer value in community pharmacies. *Health Serv Manage Res* 2019;32:36-48. doi: 10.1177/0951484818761730

57. Mirzaei A, Carter SR, Chen JY, et al. Development of a questionnaire to measure consumers' perceptions of service quality in community pharmacies. *Res Social Adm Pharm* 2019;15:346-57. doi: 10.1016/j.sapharm.2018.05.005 [

58. Abu Hagar R, El-Dahiyat F, El Refae G. Risk management in community pharmacy practice in Abu Dhabi Region: a cross-sectional study. *Journal of Pharmaceutical Health Services Research* 2020;11:275-85. doi: 10.1111/jphs.12364

59. Sato N, Fujita K, Kushida K, et al. Exploring the factors influencing the quality of "Health Support Pharmacy" services in Japan: Perspectives of community pharmacists. *Res Social Adm Pharm* 2020;16:1686-93. doi: 10.1016/j.sapharm.2020.02.012

For peer review only

60. Goto K, Sato T, Shikamura Y, et al. Examining the association between the "My Pharmacist" model and the service quality of community pharmacies. *Res Social Adm Pharm* 2020;16:958-66. doi: 10.1016/j.sapharm.2019.10.012

61. aizpurua arruti x. Confirmación de los compromisos de una Farmacia Amigable. *Pharmaceutical Care España* 2020;22:25-33.

62. Clabaugh M, Newlon JL, Illingworth Plake KS. Perceptions of working conditions and safety concerns in community pharmacy. *J Am Pharm Assoc (2003)* 2021;61:761-71. doi: 10.1016/j.japh.2021.06.011

63. Loo J, Greaves G, Lewis PJ. Exploring patients' pharmacy stories: an analysis of online feedback. *International Journal of Clinical Pharmacy* 2021;43:1584-93. doi: 10.1007/s11096-021-01287-2

64. Abebe E, Stone JA, Lester CA, et al. Quality of Handoffs in Community Pharmacies. *J Patient Saf* 2021;17:405-11. doi: 10.1097/pts.0000000000000382

65. Wongvedvanij R, Darawong C. Patients' perception toward the service quality of community pharmacies for non-prescription medicines during the COVID-19 pandemic. *International Journal of Healthcare Management* 2022:1-7.

66. Schommer JC, Lee S, Gaither CA, et al. Improving the Experience of Providing Care in Community-Based Pharmacies. *Pharmacy (Basel)* 2022;10 doi: 10.3390/pharmacy10040067

67. Wongvedvanij R, Darawong C. Enhancing the service quality of community pharmacies for non-prescription medicines based on the perspectives of pharmacists and patients: a qualitative study. *Journal of Communication in Healthcare* 2022:1-8. doi: 10.1080/17538068.2022.2026057

68. Kummer I, Mudric J, Cikaric T, et al. Patients' perception of the quality of community pharmacy services using the critical incident technique. *Vojnosanitetski Pregled* 2022;79:605-12. doi: https://dx.doi.org/10.2298/VSP200518123K

69. Sepp K, Cavaco A, Volmer D. The principles of person-centredness in quality patient care-Evaluation of the Community Pharmacy Services Quality Guidelines in Estonia. *Int J Health Plann Manage* 2022;37 Suppl 1:101-14. doi: 10.1002/hpm.3567

70. Snyder ME, Zillich AJ, Primack BA, et al. Exploring successful community pharmacist-physician collaborative working relationships using mixed methods. *Res Social Adm Pharm* 2010;6:307-23. doi: 10.1016/j.sapharm.2009.11.008

71. Rubio-Valera M, Jové AM, Hughes CM, et al. Factors affecting collaboration between general practitioners and community pharmacists: a qualitative study. *BMC Health Services Research* 2012;12:188. doi: 10.1186/1472-6963-12-188

72. Kelly DV, Bishop L, Young S, et al. Pharmacist and physician views on collaborative practice: Findings from the community pharmaceutical care project. *Can Pharm J (Ott)* 2013;146:218-26. doi: 10.1177/1715163513492642

73. Löffler C, Koudmani C, Böhmer F, et al. Perceptions of interprofessional collaboration of general practitioners and community pharmacists - a qualitative study. *BMC Health Serv Res* 2017;17:224. doi: 10.1186/s12913-017-2157-8

74. Hashemian F, Emadi F, Roohi E. Collaboration between pharmacists and general practitioners in the health care system in the Islamic Republic of Iran. *East Mediterr Health J* 2016;22:375-82. doi: 10.26719/2016.22.6.375

75. Hindi AMK, Schafheutle EI, Jacobs S. Community pharmacy integration within the primary care pathway for people with long-term conditions: a focus group study of patients', pharmacists'

For peer review only

and GPs' experiences and expectations. *BMC FAMILY PRACTICE* 2019;20 doi: 10.1186/s12875-019-0912-0

76. Villako P, Raal A. A survey of Estonian consumer expectations from the pharmacy service and a comparison with the opinions of pharmacists. *Pharm World Sci* 2007;29:546-50. doi: 10.1007/s11096-007-9102-6

77. Sakurai H, Nakajima F, Tada Y, et al. An investigation on pharmacy functions and services affecting satisfaction of patients with prescriptions in community pharmacies. *Yakugaku Zasshi* 2009;129:581-91. doi: 10.1248/yakushi.129.581

78. Horvat N, Kos M. Slovenian pharmacy performance: a patient-centred approach to patient satisfaction survey content development. *Int J Clin Pharm* 2011;33:985-96. doi: 10.1007/s11096-011-9572-4

79. Patterson BJ, Doucette WR, Urmie JM, et al. Exploring relationships among pharmacy service use, patronage motives, and patient satisfaction. *J Am Pharm Assoc* 2013;53:382-9. doi: 10.1331/JAPhA.2013.12100

80. Merks P, Kaźmierczak J, Olszewska AE, et al. Comparison of factors influencing patient choice of community pharmacy in Poland and in the UK, and identification of components of pharmaceutical care. *Patient Prefer Adherence* 2014;8:715-26. doi: 10.2147/ppa.S53829

81. McMillan SS, Sav A, Kelly F, et al. How to attract them and keep them: the pharmacy attributes that matter to Australian residents with chronic conditions. *International Journal of Pharmacy Practice* 2014;22(4):238-45. doi: 10.1111/ijpp.12075

82. Mehralian G, Rangchian M, Rasekh HR. Client priorities and satisfaction with community pharmacies: the situation in Tehran. *Int J Clin Pharm* 2014;36:707-15. doi: 10.1007/s11096-014-9928-7

83. Chen Y, Fu FQ. The behavioral consequences of service quality: an empirical study in the Chinese retail pharmacy industry. *Health Mark Q* 2015;32(1):14-30. doi: 10.1080/07359683.2015.1000706 [published Online First: 2015/03/10]

84. Alhomoud F, Kunbus A, Ameer A, et al. Quality Assessment of community pharmacy services provided in the United Arab Emirates: patient experience and satisfaction. *Journal of Applied Pharmaceutical Science* 2016;6:17-23. doi: 10.7324/JAPS.2016.60303

85. Nilugal KC, Kaur MJ, Molugulu N, et al. Patients' attitudes and satisfaction towards community pharmacy in Selangor, Malaysia. *Le Pharmacien Hospitalier et Clinicien* 2016;51:210-21. doi: https://doi.org/10.1016/j.phclin.2015.08.005

86. Feehan M, Walsh M, Godin J, et al. Patient preferences for healthcare delivery through community pharmacy settings in the USA: A discrete choice study. *J Clin Pharm Ther* 2017;42:738-49. doi: 10.1111/jcpt.12574

87. Aziz MM, Ji W, Masood I, et al. Patient Satisfaction with Community Pharmacies Services: A Cross-Sectional Survey from Punjab; Pakistan. *Int J Environ Res Public Health* 2018;15. doi: 10.3390/ijerph15122914 [published Online First: 2018/12/24]

88. Hindi AMK, Schafheutle EI, Jacobs S. Applying a whole systems lens to the general practice crisis: Cross-sectional survey looking at usage of community pharmacy services in England by patients with long-term respiratory conditions. *BMJ Open* 2019;9:e032310. doi: https://dx.doi.org/10.1136/bmjopen-2019-032310

89. Bratkowska K, Religioni U, Krysiński J, et al. Quality of Pharmaceutical Services in Independent Pharmacies and Pharmacy Chains in Poland from the Patient Perspective. *Patient Prefer Adherence* 2020;14:2459-67. doi: 10.2147/ppa.S284014

For peer review only

90. Al-Jumaili AA, Ameen IA, Alzubaidy DA. Influence of Pharmacy Characteristics and Customer Quality of Life on Satisfaction of Community Pharmacy Customers. *Innov Pharm* 2020;11. doi: 10.24926/iip.v11i1.2434

91. Thang D, Thuy L, Tien L, et al. Factors impacting on customer satisfaction with community pharmacies in vietnam. *Research in Social and Administrative Pharmacy* 2018;14:e55-e56. doi: 10.1016/j.sapharm.2018.05.123

92. Mohamud A, Shire F, Hussain K, et al. Assessing Patient Satisfaction with the Service Quality Provided in Community Pharmacies: Khartoum Locality, Sudan. *Journal of Young Pharmacists* 2021;13:405-10. doi: 10.5530/jyp.2021.13.98

93. Parinyarux P, Yotsombut K. Customers' satisfaction toward drugstore facilities and services based on the good pharmacy practice standard in Thailand. *Pharm Pract (Granada)* 2022;20:2601. doi: 10.18549/PharmPract.2022.1.2601

94. Tran VD, Dorofeeva V. Applying Q-methodology to study customer satisfaction with quality of community pharmacy services in Vietnam. *Tropical Journal of Pharmaceutical Research* 2018;17:2281-89. doi: 10.4314/tjpr.v17i11.25

95. Tran VD, Dorofeeva VV, Loskutova EE, et al. Elderly consumers’ satisfaction with the quality of community pharmacy services in Ho Chi Minh City, Vietnam: a Q-methodology study. *Pharmacia* 2020;67:303-09.

96. Puumalainen, II, Kause JM, Airaksinen MS. Quality assurance instrument focusing on patient counseling. *Ann Pharmacother* 2005;39:1220-6. doi: 10.1345/aph.1E629

97. James DH, Hatten S, Roberts D, et al. Identifying criteria for assessing the quality of medicines use review referral documentation by community pharmacists. *International Journal of Pharmacy Practice* 2008;16:365-74. doi: 10.1211/ijpp.16.6.0005

98. Trap B, Hansen E, Trap R, et al. A new indicator based tool for assessing and reporting on good pharmacy practice. *Southern Med Review* 2010;3:4-11.

99. De Bie J, Kijlstra NB, Daemen BJG, et al. The development of quality indicators for community pharmacy care. *BMJ Quality & Safety* 2011;20:666. doi: 10.1136/bmjqs.2010.045237

100. Schoenmakers TW, Teichert M, Braspenning J, et al. Evaluation of quality indicators for Dutch community pharmacies using a comprehensive assessment framework. *J Manag Care Spec Pharm* 2015;21:144-52. doi: 10.18553/jmcp.2015.21.2.144

101. Teichert M, Schoenmakers T, Kylstra N, et al. Quality indicators for pharmaceutical care: a comprehensive set with national scores for Dutch community pharmacies. *Int J Clin Pharm* 2016;38:870-9. doi: 10.1007/s11096-016-0301-x

102. Arkaravichien W, Wongpratat A, Lertsinudom S. Quality indicators to compare accredited independent pharmacies and accredited chain pharmacies in Thailand. *Int J Clin Pharm* 2016;38:899-907. doi: 10.1007/s11096-016-0307-4

103. Grey E, Harris M, Rodham K, et al. Characteristics of good quality pharmaceutical services common to community pharmacies and dispensing general practices. *Int J Pharm Pract* 2016;24:311-8. doi: 10.1111/ijpp.12253

104. Júnior E, Halila G, Reis W, et al. Quality indicators of pharmacists' services in community pharmacies in Paraná State, Brazil. *Brazilian Journal of Pharmaceutical Sciences* 2017;53 doi: 10.1590/s2175-97902017000116021

For peer review only

105. Waltering I, Scheppe S, Kurth V, et al. [Quality indicators for medication reviews in community pharmacies]. *Z Evid Fortbild Qual Gesundhwes* 2020;153-154:44-53. doi: 10.1016/j.zefq.2020.03.007

106. Okpalanma N, Obinna E. DEVELOPMENT OF QUALITY INDICATORS FOR PHARMACEUTICAL CARE PERFORMANCE ASSESSMENT IN NIGERIAN COMMUNITY PHARMACIES: A DELPHI PANEL. *International Journal of Pharmacy and Pharmaceutical Sciences* 2021:50-55. doi: 10.22159/ijpps.2021v13i2.39179

107. Fernandes B, Freitas R, Melchiors A, et al. Proposal of a quality assessment tool intended for community pharmacies with the use of a geographic information system. *Revista de Gestão em Sistemas de Saúde* 2021;10:253-74. doi: 10.5585/rgss.v10i3.17863

108. Harding G, Wilcock M. Community pharmacists' perceptions of medicines use reviews and quality assurance by peer review. *Pharm World Sci* 2010;32:381-5. doi: 10.1007/s11096-010-9381-1

109. Hallit S, Sacre H, Sarkis H, et al. Good Pharmacy Practice Standardized for Community Pharmacists: The Lebanese Order of Pharmacists Initiative. *J Res Pharm Pract* 2019;8:29-32. doi: 10.4103/jrpp.JRPP_18_96

110. Benrimoj SI, Gilbert AL, de Almeida Neto AC, et al. National implementation of standards of practice for non-prescription medicines in Australia. *Pharm World Sci* 2009;31:230-7. doi: 10.1007/s11096-008-9266-8

111. Badro DA, Sacre H, Hallit S, et al. Good pharmacy practice assessment among community pharmacies in Lebanon. *Pharm Pract (Granada)* 2020;18:1745. doi: 10.18549/PharmPract.2020.1.1745

112. Sepp K, Cavaco AM, Raal A, et al. Profession Driven Improvement of the Quality of Pharmacy Practice-Implementation of Community Pharmacy Services Quality Guidelines in Estonia. *Healthcare (Basel)* 2021;9. doi: 10.3390/healthcare9070804

113. Patterson JA, Holdford DA, Harpe SE. Patient preferences for objective quality metrics during community pharmacy selection: A discrete choice experiment. *Res Social Adm Pharm* 2019;15:641-49. doi: 10.1016/j.sapharm.2018.08.002

114. World Health Organization. Regional Office for E, European Observatory on Health S, Policies, et al. Improving healthcare quality in Europe: characteristics, effectiveness and implementation of different strategies. Copenhagen: World Health Organization. Regional Office for Europe 2019:419 p.

115. World Health Organization. Framework on integrated, people-centred health services: Report by the Secretariat, 2016.

116. Sim TF, Wright B, Hattingh L, et al. A cross-sectional survey of enhanced and extended professional services in community pharmacies: A pharmacy perspective. *Res Social Adm Pharm* 2020;16:511-21. doi: 10.1016/j.sapharm.2019.07.001

117. Alam S, Osama M, Iqbal F, et al. Reducing pharmacy patient waiting time. *International Journal of Health Care Quality Assurance* 2018;31:834-44. doi: 10.1108/IJHCQA-08-2017-0144

118. Patel PM, Vaidya V, Osundina F, et al. Determining patient preferences of community pharmacy attributes: A systematic review. *Journal of the American Pharmacists Association* 2020;60(2):397-404. doi: https://doi.org/10.1016/j.japh.2019.10.008

119. Hindi AMK, Jacobs S, Schafheutle EI. Solidarity or dissonance? A systematic review of pharmacist and GP views on community pharmacy services in the UK. *Health Soc Care Community* 2019;27(3):565-98. doi: 10.1111/hsc.12618

For peer review only

120. Manary MP, Boulding W, Staelin R, et al. The Patient Experience and Health Outcomes. *New England Journal of Medicine* 2012;368:201-03. doi: 10.1056/NEJMp1211775

121. Cathal D, Laura L, Derek B. A systematic review of evidence on the links between patient experience and clinical safety and effectiveness. *BMJ Open* 2013;3:e001570. doi: 10.1136/bmjopen-2012-001570

122. Nadia S, Claire G-S, Susan EB, et al. Quality indicator framework for primary care of patients with dementia. *Canadian Family Physician* 2022;68(9):e270. doi: 10.46747/cfp.6809e270

123. Valentijn PP, Vrijhoef HJM, Ruwaard D, et al. Towards an international taxonomy of integrated primary care: a Delphi consensus approach. *BMC Family Practice* 2015;16(1):64. doi: 10.1186/s12875-015-0278-x

For peer review only

Tables

Table 1: Search strategy

Concept	Key terms*
Healthcare quality	"Quality" OR "healthcare quality" OR "quality of health care" OR "quality improvement" OR "quality assessment" OR "quality assurance"
AND	"Community pharmacy" OR "retail pharmacy"
Community pharmacy	

*Different wildcards and truncations were used depending on the database

Table 2: Inclusion and exclusion criteria

Inclusion criteria	Exclusion criteria
**Setting:** Community pharmacy	non-community pharmacy setting
**Design/Study type:** Empirical studies	**Design/Study type:** Literature reviews.
**Location:** All regions	
**Publication date:** 2005 onwards	
**Publication type**:<br>Peer reviewed journal papers,<br>Reports on QI indicator development	**Publication type:**<br>Conference abstracts.<br>Commentary/opinion pieces /editorials<br>Reviews
**Focus of study:**<br>• Definitions/dimensions of quality in community pharmacy (including patient experience, environment, safety).<br>• Development/assessment of quality indicators/standards for community pharmacy healthcare services<br>• Patient satisfaction with community pharmacy healthcare services<br>• Factors influencing quality of care in	**Focus of study:**<br>• Advancing the scope of pharmacists and/or pharmacy technicians in practice<br>• Integrating pharmacists/pharmacy technicians in other healthcare settings.<br>• Pilot community pharmacy interventions/services<br>• Evaluations of individual services<br>• Impact of training<br>• Evaluations of pay-for-performance

For peer review only - http://bmjopen.bmj.com/site/about/guidelines.xhtml

community pharmacy	schemes<br><br>• Assessing approaches to measure quality (e.g. quality inspection reports, quality cards, administrative claims)

Table 3: Definition of pharmacy healthcare services

**Medicines supply**	*"the time between when the prescription is received by the pharmacy and the prescribed medicine(s) is supplied to the patient".*[36]<br><br>The dispensing process involves:<br>○ Receiving and validating the prescription<br>○ Assessing and reviewing the prescribed medicine<br>○ Selecting/preparing, packaging and checking the medicine<br>○ Labelling<br>○ Supplying and counselling the patients<br>○ Recording the intervention.[37]
**Professional pharmacy services**	*"A professional pharmacy service is an action or set of actions undertaken in or organized by a pharmacy, delivered by a pharmacist or other health practitioner, who applies their specialized health knowledge personally or via an intermediary, with a patient/client, population or other health professional, to optimize the process of care, with the aim to improve health outcomes and the value of healthcare".*[38]

For peer review

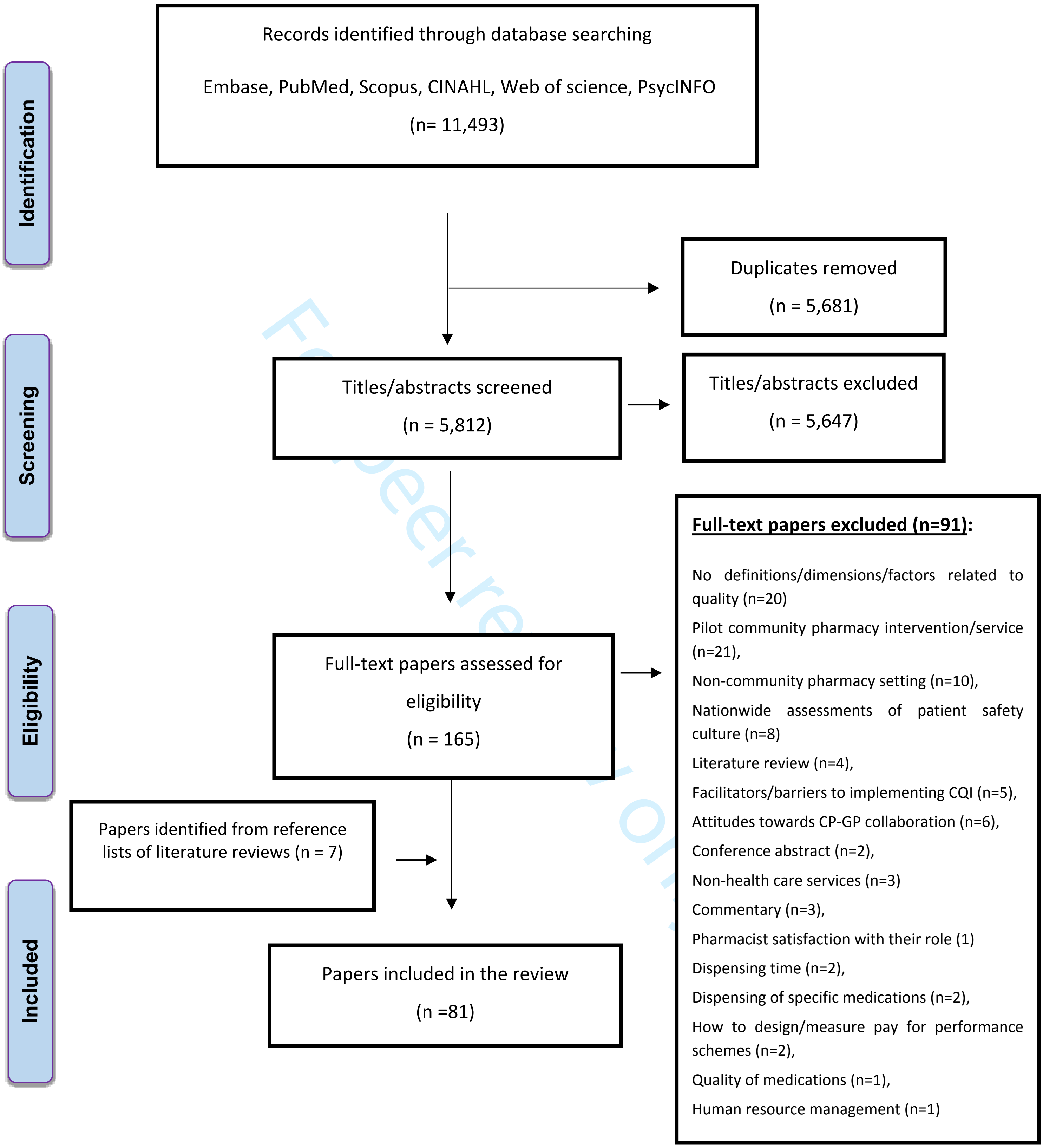

Figure 1: Flow diagram demonstrating the search procedure

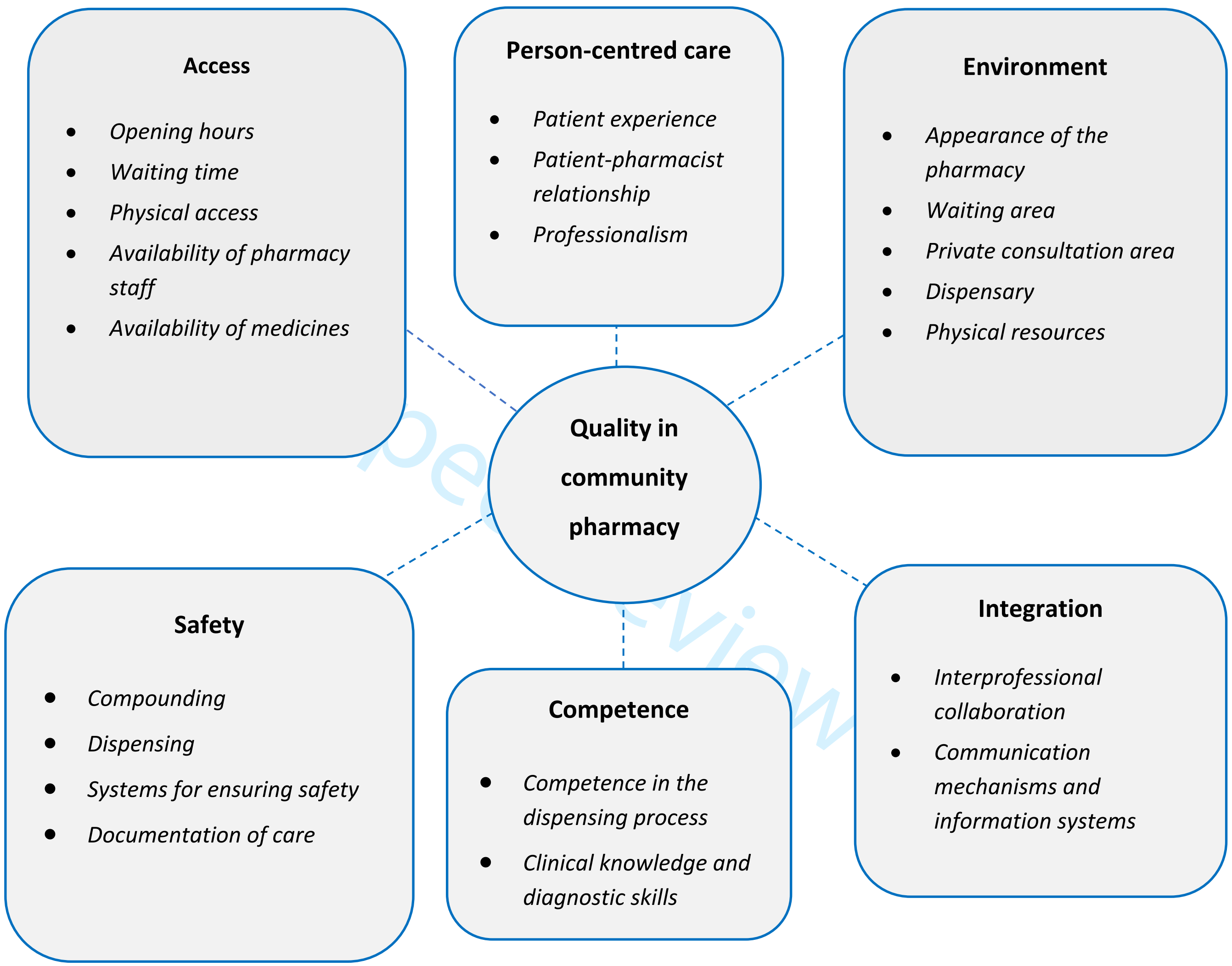

Figure 2: Overview of quality dimensions emerging from the literature

For peer review only

Supplementary File 1 – Database searches

	**PubMed**	
Search	Query	Items found
#1	"quality"[Title/Abstract]	1,287,702
#2	"healthcare quality"[Title/Abstract]	3,376
#3	"health care quality"[Title/Abstract]	3,827
#4	assessment, healthcare quality[MeSH Terms]	349,704
#5	"quality assurance"[Title/Abstract]	29,169
#6	"quality assessment"[Title/Abstract]	27,324
#7	"quality improvement"[Title/Abstract]	49,716
#8	"quality of health care"[Title/Abstract]	5,355
#9	"quality of healthcare"[Title/Abstract]	1,915
#10	#1 OR #2 OR #3 OR #4 OR #5 OR #6 OR #7 OR #8 OR #9	1,569,851
#11	"retail pharmac*"[Title/Abstract]	767
#12	"community pharmac*"[Title/Abstract]	8,652
#13	"community pharmacy services"[MeSH Terms]	5,564
#14	"pharmacies"[MeSH Terms]	9,347
#15	#11 OR #12 OR #13 OR #14	18,246
#16	#10 AND #15<br>from 2005 - 2022	**2,317**

	**Scopus**	
Search	Query	Items found
#1	TITLE-ABS-KEY (quality)	4,526,623
#2	TITLE-ABS-KEY ("community pharmac*")	14,435
#3	TITLE-ABS-KEY ("retail pharmac*")	1,182
#4	#8 OR #9	15,432
#5	#1 AND #4<br>from 2005 – 2022	**2,327**

*Using TITLE-ABS-KEY "quality" captures: TITLE-ABS-KEY ("quality improvement") TITLE-ABS-KEY ("quality assessment") TITLE-ABS-KEY ("quality assurance") TITLE-ABS-KEY (quality W/3 care)

Embase		
Search	Query	Items found
#1	(quality adj5 care).ab,kw,ti.	148,342
#2	"health care quality".mp. or health care quality/	264,992
#3	"healthcare quality".mp.	4243
#4	"quality improvement".ab,kw,ti.	77715
#5	"quality assessment".ab,kw,ti.	34799
#6	"quality assurance".ab,kw,ti.	44810
#7	quality.ab,kw,ti.	175,1710
#8	#1 or #2 or #3 or #4 or #5 or #6 or #7	191,6548
#9	community pharmacy/ or "community pharmac*".mp.	23925
#10	"retail pharmac*".ab,kw,ti.	1422
#11	#9 OR #10	24978
#13	#8 AND #11<br>from 2005-2022	**3,631**

**CINAHL**		
Search	Query	Items found
#1	(MH "Quality of Care Research") OR (MH "Quality of Health Care") OR (MH "Quality Improvement") OR (MH "Quality Assessment") OR (MH "Quality Assurance") OR "quality"	603,463
#2	"community pharmac*"	6,041
#3	(MH "Pharmacy, Retail") OR ""retail pharmac*""	8,275
#4	#2 OR #3	11,488
#5	#1 AND #4<br>Publication year 2005-2022	**1,302**

- Using “quality” as a keyword captures (MH "Quality of Care Research") OR (MH "Quality of Health Care") OR (MH "Quality Improvement") OR (MH "Quality Assessment") OR (MH "Quality Assurance")

For peer review only - http://bmjopen.bmj.com/site/about/guidelines.xhtml

PsycINFO		
Search	Query	Items found
#1	exp "Quality of Services"/ or quality.mp. or exp "Quality of Care"/	316648
#2	"community pharmac*".mp.	1245
#3	"retail pharmac*".mp. [mp=title, abstract, heading word, table of contents, key concepts, original title, tests & measures, mesh word]	143
#4	2 or 3	1366
#5	1 and 4<br>Specific range 2005-2022	**207**

- Mapping the search term "quality" to "quality of services" and "quality of care", covers: (quality adj5 care) OR "quality assessment" OR "quality improvement" OR "quality assurance"

Web of Science		
Search	Query	Items found
#1	quality (Topic)	3,205,809
#2	"community pharmac*" (Topic)	10,595
#3	"retail pharmac*" (Topic)	823
#4	#2 OR #3	11,310
#5	#1 AND #4<br>Publication years 2005-2022	**1,794**

- *Using "quality (Topic)" covers:* (quality) NEAR/3 (care or healthcare) (Topic) OR "quality assessment" (Topic) OR "quality improvement" (Topic) OR "quality assurance" (Topic)

For peer review only

Author(s) & year	Country	Purpose	Study design	Sample	Pharmacy service(s)	Key findings mapped under quality dimensions
Puumalainen et al. (2005)[96]	Finland	To develop a validated, easy-to-use patient counselling quality assurance instrument for community pharmacists.	Delphi	2 expert panels: Panel 1, consisting of experienced pharmacy practitioners (n = 10), and Panel 2, consisting of academic and professional experts (n = 10).	• Medicines supply	• **Person-centred care:** Patient experience: Indicators that enable practitioners to understand patients' specific information and communication needs.<br>• **Competence:** Competence in the dispensing process: indicators relevant to technical skills in dispensing prescriptions; incorporation of patient counselling in the dispensing process.<br>• **Environment:** Physical resources: availability and accessibility of information sources (manual, electronic); guidelines on patient counselling practices in the pharmacy; guidelines on patient counselling with local healthcare personnel
Worley (2006)[39]	USA	To test a pharmacist-patient relationship quality model in a group of older persons with diabetes from the	Survey	311 community-dwelling older persons (65 years of age and older), using at least one prescription medication and primarily	• Professional pharmacy service	• **Person-centred care** Patient-pharmacist relationship: contains measurement items from 5 study constructs -

For peer review only

		patient's perspective. Pharmacist-patient relationship commitment was the outcome of relationship quality studied.		obtaining their prescriptions from some type of nonmail order pharmacy		1. Pharmacist participative behaviour/patient-centeredness of relationship<br>2. Patient participative behaviour<br>3. Pharmacist-patient interpersonal communication<br>4. Relationship quality<br>5. Relationship commitment
Vilako et al. (2007)[76]	Estonia	To assess the preferences of pharmacy customers when choosing a pharmacy and their expectations of the service and comparing these with the opinions of pharmacists.	Survey	Patients (n=1979) in cities (in 3 community pharmacies), towns (in 2 community pharmacies), and in small towns (in 2 community pharmacies). The survey was also carried out among community pharmacists (n=135) in different regions of Estonia.	• Not specified	Survey items:<br>• **Access**<br>Physical access: Parking space near pharmacy; Comfortable entering to pharmacy<br>Availability of medicines: wide choice of products<br>Waiting time: Quick service<br>• **Environment**<br>Waiting area: Comfortable staying in pharmacy.<br>Private consultation area: privacy and discretion<br>• **Competence**<br>Clinical knowledge and diagnostic skills: Professional consulting;

For peer review only

						Help with choosing right medicine
James et al. (2008)[97]	UK	To develop explicit criteria against which the quality of Medicine Use Review (i.e. MUR; a planned patient-pharmacist consultation to discuss medicines use) referral documentation can be assessed.	Delphi	Sixteen panellists (these were 10 out of 14 MUR accreditation tutors who were invited to take part and six pharmacy practitioners from a possible 22 primary care organisations in Wales)	• Professional pharmacy service	• **Safety**<br>Documentation of care: consensus was achieved for 20 quality indicators that correspond with that of documenting a patient-pharmacist consultation.
Benrimoj et al. (2009)[110]	Australia	To implement nationally a quality improvement package in relation to the Standards of Practice for the Provision of Non-Prescription Medicines.	Randomly selected pharmacies were coached on the implementation of the Standards of Practice for the Provision of Non-Prescription Medicines. Pre and post measurements of the level of adherence to the Standards were taken.	2,706 pharmacies	• Medicines supply<br>• Professional pharmacy service (supply of OTC medicines following minor illness consultation)	Standards used in this study defined and described the professional activities required for the provision of medicines at a consistent and measurable level of practice.<br><br>**Environment:** Appearance of the pharmacy (3 statements); Physical resources (1 statement); Private consultation area (1 statement)<br><br>**Person-centred care:** Professionalism (3 statements)<br><br>**Competence:** Competence in dispensing process/clinical knowledge and diagnostic skills (8 statements)<br><br>**Safety:** Documentation of care (2 statements); Systems for

For peer review only - http://bmjopen.bmj.com/site/about/guidelines.xhtml

						ensuring safety and safety. (1 statement)
Rapport et al. (2009)[40]	UK	Identifying the extent to which pharmacy spaces are aligned to good professional practice, enhance a professional's sense of self and meet the demands of the public.	Mixed-methods approach employing biographic and photographic techniques	16 pharmacists	• Medicines supply<br>• Professional pharmacy service	• **Environment**<br>Dispensary: Essential to be well organised and to have control over the space and the way it functions. Barriers for dispensing to function in an orderly fashion: unwanted interruptions, undesired observation, lack of formality, lack of room and changes to the order and running of things brought about by others'<br><br>Private consultation area: Some have reservations about its size and positioning. In the smaller settings, particularly within Independent and dedicated pharmacies, consultation rooms are shoehorned into an already limited workspace, bringing additional pressure overflow storage.<br><br>Appearance of the pharmacy: Brings into question the pharmacist's position and professional status. The sales counter is particularly problematic when it comes to distractions with members of the public attempting to overcome

For peer review only

						the divide between pharmacist, dispensary and sales floor.
Sakurai et al. (2009)[79]	Japan	To investigate how pharmacy functions and services affect patient satisfaction	Survey	30186 Patients from 178 pharmacies whose purpose of use of the pharmacies was not only for prescription dispensing but also OTC medicines	• Medicines supply<br>• Sale of OTC medicines following minor illness consultation	Survey items:<br>• **Access:**<br>Opening hours: opening hours<br>Physical access: location<br>Waiting time: average waiting time; maximum waiting time<br>Availability of medicines: amount of pharmaceutical stock<br>• **Environment:**<br>Private consultation area: Privacy considerations<br>Waiting area: number of waiting chairs<br>Physical resources: number of blood pressure, bone density and other measuring instruments
Feletto et al. (2010)[44]	Australia	To determine the needs of pharmacies that were important and the elements requiring improvement when implementing and delivering cognitive pharmaceutical services.	Survey	355 community pharmacies	• Professional pharmacy service	Survey items covered the following areas:<br>• **Access:** opening hours (1)<br>• **Environment:** Private consultation area (3); appearance of the pharmacy (2)<br>• **Competence:** training of pharmacy staff (2)<br>• **Integration** (1)

For peer review only

Harding et al. (2010)[108]	UK	To explore existing mechanism to ensure quality assurance of medicine use reviews (MURs), and to identify those parameters of an MUR that community pharmacists consider as indicators of quality.	Survey	50 pharmacists, a third of which were from locum pharmacists.	• Professional pharmacy service	Individual survey items were not provided but the analysis of findings were mapped on the quality dimensions:<br>• **Competence:** <u>Clinical knowledge and diagnostic skills:</u> The single most frequently reported determinant for undertaking an MUR was the pharmacists' judgement (84% n = 42). Over 70% (n = 35) of respondents considered that undertaking MURs required specialist skills
Scahill et al. (2010)[41]	New Zeeland	To develop a multi-constituent model of organizational effectiveness for community pharmacy.	Face to face brainstorming to generate statements describing what constitutes an effective community pharmacy, and sorting of the statements into themes with rating of each statement for importance	14 stakeholders representing policy-makers and health care providers including; community pharmacy, professional pharmacy organizations, primary health care funders and policy-makers, general practitioners and general practice support organizations	• Medicines supply<br>• Professional pharmacy service	• **Safety:** statements addressed how an organisation could promote safe and effective workflow; and ensure safe use of medicines.<br>• **Integration:** statements addressed how an organisation could focus on patient needs; and better integrate within primary care.

For peer review only

Snyder et al (2010)[70]	USA	To describe the professional exchanges that occurred between community pharmacists and physicians engaged in successful collaborative working relationships (CWRs), using a published conceptual model and tool for quantifying the extent of collaboration	Semi structured interviews, and completion of the Pharmacist-Physician Collaborative Index	Five pairs of community pharmacists and physician colleagues	• n/a	• **Integration**<br>*Interprofessional collaboration*: Pharmacists were the primary initiator of these CWRs. Initial conversations were usually (but not always) conducted face-to-face and often scheduled in advance by the pharmacist.<br><br>Establishing trust was the provision of high-quality recommendations that improved patient outcomes. Both professionals commented on how seeing these positive outcomes was key to the success of their relationship.<br><br>Resistance manifested passively, as lack of physician response to recommendations, and actively, as refusal to provide patient laboratory data in spite of signed medical releases and hesitations to provide referrals for clinical services beyond patient education.
Trap et al. (2010)[98]	Ethiopia, Uganda and Zimbabwe.	To develop an indicator-based tool for systematic assessment and reporting of good pharmacy practice (GPP).	direct observations, record reviews, interviews and simulated clients in surveyed facilities		• Medicines supply<br>• Professional pharmacy service	Indicators developed focusing on:<br>• **Safety**<br>Documentation of care (5 indicators); Dispensing (14 indicators)<br>• **Access**

peer review only

						Availability of medicines (1 indicator)<br><br>• **Environment**<br>Appearance of the pharmacy (7 indicators); physical resources (1 indicator)<br><br>• **Competence**<br>Competence in the dispensing process (4 indicators), Clinical knowledge and diagnostic skills (2 indicators)
Urbonas et al. (2010)[42]	Lithuania	To analyse pharmacy specialists' attitudes toward the quality of pharmaceutical services at Lithuanian community pharmacies.	Survey	471 Lithuanian community pharmacy specialists	• Medicines supply	Survey covered two quality dimensions:<br><br>• **Competence**<br>Competency in the dispensing process (5 indicators covering side effects, time spent with patient, information about drug therapy and healthy lifestyle)<br><br>• **Person-centred care**<br>Patient experience (5 indicators covering consideration of financial capabilities, patient needs, helpfulness to each patient, willingness to get patient to come back to pharmacy)
White et al. (2010)[43]	Australia	To investigate the views of a range of stakeholders regarding the effectiveness of	In-depth interviews	20 in-depth interviews were conducted with various stakeholders, including community	• Medicines supply	• **Environment:**<br>Appearance of the pharmacy:<br>Maximizing the visibility of health ranges

For peer review only

		service quality as a differentiating position for community pharmacy.		pharmacists, managers of pharmacy groups, and industry advisers	• Professional pharmacy service	• **Person-centred care:** Patient experience: Greeting customers by name<br><br>• **Competence:** Competence in the dispensing process: sitting with customer to discuss prescription and health needs<br><br>Clinical knowledge and diagnostic skills: potential for community pharmacy to become more service orientated by offering home medication reviews, screening program and disease state management.
De Bie et al. (2011)[99]	Netherlands	To develop a national system of quality indicators for community pharmacy care, reported by community pharmacies.	Delphi	14 pharmacy practice experts and 76 practising pharmacists	• Medicines supply	Indicators focused on:<br>• **Competence** Competence in the dispensing process (7 indicators)<br><br>• **Safety** Systems for ensuring safety quality (15 indicators); compounding (7 indicators); dispensing (13 indictors)
Horvat et al. (2011)[78]	Slovenia	To identify content of pharmacy performance	Interviews + Delphi	Phase 1: interviews with 43 pharmacy users were conducted to identify	• Medicines supply	• **Person-centred care:**

For peer review only

		relevant to patient satisfaction.		patients' experiences and expectations relating to pharmacies.<br><br>Phase 2: a 10 member expert panel was employed in a two round Delphi technique to rate the importance of each item for the patient satisfaction.		Patient-pharmacist relationship (1 item); Professionalism (17 items)<br><br>• **Competence:**<br>Competence in the dispensing process (18 items)<br><br>• **Access:**<br>Waiting time (1); Opening hours (2); Availability of medicine (3); Physical access (3); availability of pharmacist (1)<br><br>• **Environment**: private consultation area (1); waiting area (2); appearance of the pharmacy (10); appearance of the pharmacy (4)
AHRQ (2012)[45]	USA	The Agency for Healthcare Research and Quality (AHRQ) funded the development of the Community Pharmacy Survey on Patient Safety Culture. This survey is designed specifically for community pharmacy providers and other staff and asks for their opinions about the	Survey	Details on development of survey not provided	Not specified	The survey includes 36 items that measure 11 composites of<br><br>• Safety culture:<br>Physical space and environment (3 items);<br>Teamwork (3 items);<br>Staff training and skills (4 items);<br>Communication openness (3 items);<br>Patient counselling (3 items);<br>Staffing, work pressure and pace (4 items);

For peer review only

		culture of patient safety in their pharmacy.				Communication about prescriptions across shifts (3 items);<br>Communication about mistakes (3 items);<br>Response to mistakes (4 items)<br>Organizational learning (3 items);<br>Overall perceptions of patient safety (3 items)
Halsall et al. (2012)[27]	UK	To develop a conceptual framework characterizing healthcare quality in the community pharmacy setting.	Focus groups	10 focus group discussions with 47 participants (patients and their carers, pharmacists and pharmacy staff, and NHS staff who commissioned pharmacy services) were conducted across the northwest of England, United Kingdom.	• Medicines supply	• **Access**<br>Availability of medicines: Patient awareness of available medicines<br>Physical access: whether patients could physically access care.<br><br>• **Environment**<br>Physical resources: Pharmacy personnel having access to adequate structures to provide care and that these should be continually reviewed.<br><br>• **Competence:**<br>Competence in the dispensing process: Supplying medicines appropriately and providing individualized advice to patients.<br><br>• **Person-centred care:**<br>Patient experience: Ensuring patients/carers at the point of care have a positive perception of the experience

Dadfar et al. (2012)[46]	Iran	To assess the quality of Tehran pharmacies' services and their impacts on the pharmaceutical firms, and suggest some improvements	Survey + in-depth interviews	127 pharmacy users completed the questionnaire.<br><br>32 interviews with Pharmacists (n=10),<br><br>Pharmaceutical managers<br><br>(n=8);<br><br>patient<br><br>(n=9)<br><br>Physician<br><br>(n=4);<br><br>MOH authority (n=1)	• Medicines supply	Survey covered the following dimensions of quality:<br><br>• **Competence:** Competence in the dispensing process (5 items)<br><br>• **Person-centred care:** Professionalism (5 items)<br><br>• **Environment:** Physical resources (1 item); appearance of the pharmacy (1 item); appearance of the pharmacy (1 item)<br><br>• **Access:** Opening hours (1 item); waiting time (1 item)
White et al. (2012)[47]	Australia	To explore the perceptions of pharmacy staff regarding the factors that constitute a high level of service quality using the service quality determinants proposed by the Conceptual Model of Service Quality	Structured interviews	27 pharmacy assistants and 6 pharmacists in 3 community pharmacies in Sydney.	Not specified	• **Safety:** All the participants acknowledged the existence of some form of internal quality control programs, but provided inconsistent answers and uncertainty regarding frequency, process, and content of such programs, within and across the pharmacies
Phipps et al. (2012)[48]	UK	To evaluate the internal reliability, factor structure and construct validity of the Pharmacy	Survey	A total of 4105 members of the community pharmacy workforce, all drawn from one of the	Not specified	24 items emerged relative to<br><br>• **Safety**

For peer review only

		Safety Climate Questionnaire (PSCQ) when applied to a pan-European sample of community pharmacies.		five participating countries (Denmark, Germany, the Netherlands, Portugal and Great Britain)		Safety culture: Organizational learning (13 items); Blame culture (4 items); Working conditions (4 items); Safety focus (3 items).
Rubio-Valera et al (2012)[71]	Spain	To identify and analyse factors affecting GP-CP collaboration.	Semi-structured interviews	18 GPs, 19 community pharmacists	n/a	• **Integration**<br>Communication mechanisms and information systems: 1) having a coordinator and 2) sharing a clinical chart to have access to patient information
Kelly et al. (2013)[72]	Canada	To capture the opinions of family physicians and community pharmacists in Newfoundland and Labrador (NL) regarding collaborative practice.	Survey	407 pharmacists & 462 family doctors	n/a	• **Integration**<br>Communication mechanisms and information systems: Pharmacists preferred telephone or face to-face communication over paper correspondence with GPs. GPs preferred telephone communication. Pharmacists believed that electronic transfer of information should be explored.<br><br>Interprofessional collaboration: GPs believed that the most important pharmacist functions were to help improve patient adherence and fill prescriptions. Pharmacists would like to participate more in decisions regarding identification and management of drug-related problems—managing drug interactions, providing drug

For peer review only

						information to inform decisions around patient drug therapy and assisting to modify drug therapy to resolve patient-specific problems.<br><br>Incentivisation: Lack of compensation and the need to collaborate with multiple GPs/pharmacists to provide care for patients were viewed as the 2 most significant barriers.
Patterson et al. (2013)[79]	USA	To describe and identify significant relationships among pharmacy service use, general and service-specific patient satisfaction, pharmacy patronage motives, and marketing awareness in a service-oriented, independent community pharmacy	Survey	241 patients	• Medicines supply	Survey items coverage:<br>• **Access:** Waiting time (2 items); Availability of pharmacist (1 item)<br>• **Patient-experience:** Professionalism (6 items); patient experience (2 item); Patient-pharmacist relationship (1 item)<br>• **Competence:** Competence in the dispensing process (7 items)<br>• **Environment:** Appearance of the pharmacy (1 item); private consultation area (1 item)
Merks et al. (2014)[80]	Poland	To compare factors that influence a patient's choice of pharmacy in Poland and in the UK, to identify which of them are components of pharmaceutical care, and to relate them to	Survey	417 patients from 36 pharmacies in Poland and 405 patients from 56 pharmacies in the UK.	• Not specified	• **Access**<br>Physical access: The convenient location of pharmacy was one of the most frequently reported factors by Polish and British respondents.<br>• **Person-centred care**

		patient loyalty to the same pharmacy				Professionalism: professional service was one of the most frequently reported factors by Polish and British respondents. British respondents were more likely than the Polish to choose a pharmacy because of a professional service.<br><br>• **Environment**<br>Private consultation area: British respondents were more likely than the Polish to choose a pharmacy because a possibility to discuss their health problems in a separate consultation room<br>Appearance of the pharmacy: Polish respondents were more likely than the British to base their choice of pharmacy on the aesthetic decoration of the pharmacy. Aesthetic decoration of the pharmacy was more important to respondents who often visited a pharmacy (one to two times a week) than to those who visited a pharmacy less frequently.<br><br>• **Competence**<br>Clinical knowledge and diagnostic skills: Good advice received in a pharmacy was one of the most frequently reported factors by the British respondents. British respondents were more likely

For peer review only

						than the Polish to choose a pharmacy because of a possibility to receive good advice.
McMilan et al. (2014)[81]	Australia	To explore the attributes of pharmacy choice for people with chronic conditions.	Semi-structured interviews	97 interviews (patients=70, carer n=8, patient/carer n=19)	• Not specified	• **Person-centred care**<br><br>Patient experience: Taking the time to ensure that the person's individual needs were met and not identifying people solely by their condition(s) were exemplars of caring pharmacy staff. The provision of information was viewed positively by participants and deemed essential when obtaining a new medication. Some consumers from culturally diverse backgrounds sought a pharmacy where a staff member spoke the same language or had the same cultural background<br><br>Patient-pharmacist relationship: Continuity of care was another reason for utilising a regular pharmacy, as this facilitated awareness of the person's medical history. For others, medication safety was a key priority for them and hence, using a regular pharmacy was seen as a way to optimise this via continuity of care.<br><br>Professionalism: Staff approachability facilitated a

For peer review only

						relaxed environment for consumers to ask questions and seek advice, thus supporting patient empowerment and resulting in continued use of that pharmacy.<br><br>• **Access**<br>Physical access: The majority of participants selected a conveniently located pharmacy, e.g. close to their home or doctor, to use regularly, in order to reduce the time accessing care.
Mehralian et al. (2014)[82]	Iran	To assess pharmacy customers' priorities and satisfaction with community pharmacy services in Tehran	Survey	800 pharmacy customers of 200 community pharmacies in 22 districts of Tehran	• Medicines supply<br><br>• Professional pharmacy service (Sale of D medicines following minor illness consultation)	Survey items looked at:<br>• **Person-centred care:** professionalism (2)<br>• **Competence:** competency in the dispensing process (3); Clinical knowledge and diagnostic skills (5)<br>• **Access:** Availability of medicines (3), waiting time (1)<br>• **Environment:** appearance of the pharmacy (1), waiting area (1), private consultation area (1)
Odukoya et al. (2014)[49]	USA	To examine factors influencing quality of patient interaction at community pharmacy	Non-participant observation (quantitative approach)	22 community pharmacies	• Medicines supply	• **Access:**<br>Physical access: The key enabling variables affecting amount of time pharmacists spent with

		drive-through and walk-in counselling areas				patients were location of interaction (drive-through or walk-in) and level of pharmacy busyness. Pharmacists spent less time with patients at the drive-through compared to the walk-in counselling area.
Chen et al. (2015)[83]	China	Examines the impact of service quality and the mediating effects of customer satisfaction and customer loyalty on willingness to pay more.	Survey	479 retail pharmacy users in China	• Not specified	Survey items looked at:<br>• **Environment**<br>Appearance of the pharmacy (2 items)<br>• **Access**<br>Physical location (2 items); Availability of medicines (3 items); Waiting times 3 items); Opening hours (1 item)<br>• **Person-centred care**<br>Professionalism (3 items)<br>• **Safety**<br>Documentation of care (1 item)
Hattingh et al. (2015)[50]	Australia	To explore the unique privacy and confidentiality requirements of mental health consumers and carers in the Australian community pharmacy context	In-depth interviews and focus groups	There were 98 participants consisting of consumers and carers (n = 74), health professionals (n = 13) and representatives from consumer organisations (n = 11).	• Professional pharmacy service	• **Environment:**<br>Private consultation area: Consumers and carers expressed concerns that their anonymity and right to receiving sensitive information were breached when other customers were present in the pharmacy.

For peer review only

						Due to the highly accessible nature of community pharmacy services and services being provided in a public space, there is a fear of being recognised by colleagues, friends and neighbours when collecting medication<br><br>The use of a private consultation room or area was seen as a main facilitator for overcoming privacy and confidentiality issues during pharmacy interactions.
Schoenmakers et al. (2015)[100]	Netherlands	To assess the validity of 52 quality indicators (QI) for community pharmacies using the Indicator Assessment Framework (IAF)	An expert panel applied the IAF criteria to the set of QIs collected in 1,807 Dutch community pharmacies on their performance in 2011.	Expert panel consisted of 6 pharmacists from urban as well as rural areas and from different settings, such as independent pharmacies, pharmacies in pharmacy chains, or pharmacies in health centres	• Medicines supply	Indicators focused on the following domains:<br><br>• **Competence**<br>Competence in the dispensing process (3 indicators)<br>Training of pharmacy staff (1 indicator)<br><br>• **Safety**<br>Systems for ensuring safety (21 indicators); Compounding (3 indicators); dispensing (21 indicators); documentation of care (2)<br>• **Integration** (1 indicator)

For peer review only

Alhomoud et al. (2016)[84]	UAE	Assessed patients' experiences and satisfaction with community pharmacy services in the UAE, which can be used as an indicator to improve services.	Survey	415 patients	• Medicines supply.	Questionnaire comprised of Items on:<br>• **Person-centred care:** Professionalism (4); patient experience (2)<br>• **Competence:** competency in the dispensing process (5)<br>• **Access:** Availability of pharmacy staff (1)
Arkaravichien et al. (2016)[102]	Thailand	Test a quality indicators tool for feasibility by applying it in two pharmacy settings; accredited independent community pharmacies and accredited chain community pharmacies,	Observation and interviewing pharmacist in charge	60 pharmacies enrolled in the study of which 34 were independent pharmacies and 26 chain pharmacies	• Medicines supply	The tool comprised of indicators covering:<br>• **Environment:** appearance of the pharmacy (1 indicator); appearance of the pharmacy (2 indicators); dispensary (3 indicators); physical resources (1 indicator)<br>• **Access:** availability of pharmacy staff (2 indicators), availability of medicines (7 indicators)<br>• **Competence:** Competence in the dispensing process (18 indicators)
Grey et al. (2016)[103]	UK	To ask key stakeholders to confirm, and rank the importance of, a set of characteristics of good pharmaceutical service provision	Delphi	22 participants (DPs, CPs and patients/lay member)	• Medicines supply<br>• Professional pharmacy service	A set of 23 characteristics for providing good pharmaceutical services in CPs and DPs was developed:<br>• **Safety:** medicine supply (6)<br>• **Person-centred care**: patient experience (6)<br>• **Environment**: Appearance of the pharmacy (1); waiting area (1)

For peer review only

						• **Competence:** Clinical knowledge and diagnostic skills (2)<br>• **Integration:** Interprofessional collaboration (3)
Hashemian et al. (2016)[74]	Iran	To investigate the collaborative working relationship between pharmacists and GPs in terms of their attitudes, role perceptions, experience with collaborative practice, preferred method of communication, areas of current and further collaboration, and perceived barriers to interprofessional collaboration in a sample of the Iranian population	Survey	132 pharmacists and 99 general practitioners	• n/a	• **Integration**<br>Communication mechanisms and information systems: The preferred method of communication for collaborative practice for both groups was by telephone or face to face rather than by letter.<br>Interprofessional collaboration: Both groups reported that the most frequent collaboration is to "manage drug interactions", "provide patient counselling" and "manage side-effects of medications". Both groups were willing to collaborate in decision making on patients' pharmacotherapy and management of drug interactions.<br><br>Information sharing and access: Neither group was overly concerned about "liability for shared information", "lack of compensation" or "dealing with multiple care professionals" as barriers to collaboration

Nilugal et al. (2016)[85]	Malaysia	To investigate patient's attitudes, and satisfaction towards community pharmacist's role in Selangor, Malaysia	Survey	180 patients at three different community pharmacies in three different regions of Selangor state	• Medicines supply.<br>• Professional pharmacy service	Questionnaire items covered the following:<br>• **Competence:** Competency in dispensing process (7); clinical knowledge and diagnostic skills (7)<br>• **Access:** waiting time (3)<br>• **Person-centred care:** patient experience (2); professionalism (2)
Shiyanbola et al. (2016)[51]	USA	To describe older adults' perception of a quality pharmacy including their expectations of a quality pharmacy and their preferences in a quality pharmacy.	Focus groups	Six focus groups (60 patients) held in community centres and senior residence facilities in Wisconsin	• Medicines supply	• **Access:** Opening hours; availability of pharmacy staff; physical access<br>• **Person-centred care:** Patient-pharmacist relationship: Interpersonal relationship with pharmacist/pharmacy staff; familiarity with pharmacy/pharmacist staff;<br>Professionalism: friendliness and helpfulness of staff; pharmacist courtesy<br>• **Competence:** Competence in the dispensing process: ensuring medication safety; facilitates medication adherence; readily available to clarify questions

Teichert et al. (2016)[101]	Netherlands	To present a comprehensive quality indicator set for community pharmacies and to report the scores for these indicators as supplied by the majority of Dutch community pharmacies	Community pharmacists in the Netherlands were invited in 2013 to provide information for the set of 2012.Quality indicators were mapped by categories relevant for pharmaceutical care and defined for structures, processes and dispensing outcomes	Information was provided by 1739 of the 1981 Dutch community pharmacies (88 %)	• Medicines supply	Indicators focused on the following domains:<br>• **Competence**<br>Competence in the dispensing process (27 indicators); Training of pharmacy staff (5 indicators)<br>• **Safety**<br>Systems for ensuring safety quality (24 indicators); Compounding (4 indicators); medicine supply (3 indicators); documentation of care (1 indicator)<br>• **Integration** (3 indicators)
Weiss et al (2016)[52]	UK	To investigate the similarities and differences in how pharmaceutical services are provided by community pharmacies (CPs) and dispensing doctor practices (DPs) and (b) to identify the issues relevant to determining the quality of pharmaceutical services in these settings.	Mixed methods: A postal questionnaire of DPs and CPs. A subsection<br>of questionnaire respondent sites were selected<br>d to take part in case studies, which involved<br>documentary analyses, observation and staff interviews	Questionnaire: 52 CPs, 31 DPs<br>There were three CP and four DP case study sites, with 17 staff interviews	• Medicines supply	• **Person-centred care:**<br>Patient experience - providers' underlying values and commitment to providing patient-centred care. At the supermarket pharmacy, for example, staff would always strive to fulfil a patient's needs as they saw this as not only good for business but also their duty as a service provider.<br>• **Safety**<br>Medicine supply: Effective systems of work in relation to the checking of prescribed items.<br>Systems for ensuring safety: Effective systems of work in

For peer review only

						relation to the way in which dispensing errors were managed.
Koster et al. (2016)[53]	Netherlands	To provide insight into the agreement about quality of pharmaceutical care, measured both by a patient questionnaire and video observations	Pharmaceutical encounters in four pharmacies were video-recorded. Patients completed a questionnaire based upon the Consumer Quality Index. An observation protocol was used to code the recorded encounters. Agreement between video observation and patients' experiences was calculated.	109 encounters were included for analysis	• Medicines supply	• **Competence:** Competence in the dispensing process: Information provision (3 items); Medication counselling (4 items)<br>• **Person-centred care:** Professionalism: Pharmacy staff's communication style (8 items)
Feehan et al. (2017)[86]	USA	To gauge patient preferences explicitly for primary healthcare services that could be delivered through community pharmacy set-tings in the USA	Questionnaire (Discrete Choice Experiment)	10006 adults who had to have a minimal repeat use of a pharmacy for health care needs—defined as filled at least three or more prescriptions for	• Medicines supply.<br>• Professional pharmacy service	Attributes covered:<br>• **Access** Opening hours: Hours of operation Availability of medicines: Prescription ordering, availability and information

For peer review only - http://bmjopen.bmj.com/site/about/guidelines.xhtml

				themselves, at a pharmacy in the past 12months		Waiting time: Service logistics (I.e. walk in vs appointment)<br>• **Integration:** pharmacy has access to and can enter prescriptions and health information into your (the patient's) electronic medical record<br>• **Competence:**<br>Clinical and diagnostic skills: Physical examinations; Diagnostic testing; Preventive services; prescribing; Medication review services
Hipolito Junior et al. (2017)[104]	Brazil	To characterize the profiles and activities of community pharmacists, as well as the quality indicators of private community pharmacies in Paraná State - Brazil	Survey	533 pharmacists in Paraná State - Brazil	• Medicines supply<br>• Professional pharmacy service	• Five indicators relative to **environment:** Waiting area (1); Private consultation area (1); Physical resources (3).
Löffler et al. (2017)[73]	Germany	Investigating pharmacists' and general practitioners' views on barriers to interprofessional collaboration in the German health care system.	Interviews and focus groups	Six pharmacists were interviewed and four pharmacists took part in the focus group discussion.<br>Seven GPs were interviewed and eight	• n/a	• **Integration**<br>Communication mechanisms and information systems: The majority of pharmacists stated to encounter recurring difficulties getting GPs on the phone and receiving an answer to their query.<br>Interprofessional collaboration: GPs felt challenged in means of treating patients under time

For peer review only

				GPs participated in the focus group discussions.		constraints and avoiding or limiting polypharmacy. Most physicians perceived that community pharmacists were not able to respond to this challenge. GPs felt that pharmacists don't have background information on patients' medical history and/or professional knowledge to understand and reconstruct physicians' reasoning in many cases.<br>Proximity: Pharmacists employed in rural and provincial regions often experienced long-lasting working relationships to local GPs that were mostly characterized by mutual trust and appreciation. In contrast, in cities interprofessional collaboration was constrained by urban anonymity: Quite often, pharmacists hardly knew the GPs they tried to contact.<br>Incentivisation: Some GPs thought that pharmacists would have their own agenda trying to profit from patients with long-term conditions
Aziz et al. (2018)[87]	Pakistan	To assess pharmacies services with regard to patient's need	Survey	1088 patients of 544 community pharmacies	• Medicines supply.	41 Items on satisfaction covering the following dimensions:

						• **Access:** Physical access (3); opening hours (2); availability of medicines (3); waiting time (1)<br>• **Environment:** appearance of the pharmacy (1), waiting area (1), private consultation area (2); appearance of the pharmacy (1)<br>• **Person-centred care:** professionalism (2); patient experience (1)<br>• **Competence:** competency in the dispensing process (19)
Jacobs et al. (2018)[15]	UK	To explore stakeholder perceptions of the organisational and extra-organisational factors associated with service quality and quantity in community pharmacy as an established exemplar of private sector organisations providing publicly-funded healthcare.	Semi-structured interviews	Forty semi-structured interviews were conducted with service commissioners, superintendent and front-line pharmacists, purposively selected from across nine geographical areas and a range of community pharmacy organisational types in England.	• Medicines supply<br>• Professional pharmacy service	• **Competence:** Competence in the dispensing process: For dispensing, speed and accuracy were the most commonly cited elements of service quality. However, for pharmacists themselves, and for many service commissioners, accuracy was paramount.<br>Clinical knowledge and diagnostic skills: clinical aspects were considered by a number of pharmacists and commissioners to be an important element of quality either through counselling or the clinical check.<br>• **Integration** Interprofessional collaboration: Positive relationships between

For peer review only

						community pharmacies and local GP surgeries were seen to help nurture interdisciplinary practice, foster closer working around patients, increase effective signposting and improving communication.
Newlands et al. (2018)[54]	UK	To systematically identify and prioritise community pharmacy services in Scotland which required improvement and/or guideline development	A modified nominal group technique (NGT) was used for topic generation followed by an electronic Delphi survey	Stakeholder group comprising community pharmacists, policy makers, lay and pharmacy organisation representatives.	• Medicines supply<br>• Professional pharmacy service	Consensus reached on guideline development for:<br>• **Competence:** Clinical knowledge and diagnostic skills: promoting the appropriate sale and supply of over-the-counter medicines; promotion and delivery of a Minor Ailment Scheme.<br>Competence in the dispensing process: Patient counselling for prescribed medication; evidence-based strategies to promote medication adherence; enhancing medication use for vulnerable patients (including high risk, sheltered housing residents, immigrants, homeless)<br>•
Tran et al. (2018)[94]	Vietnam	To determine the pattern of pharmacy customers' viewpoints regarding their	Q-methodology	144 pharmacy customers from 40 pharmacies in four Vietnamese cities.	• Medicines supply.	Statements covered the following dimensions:<br>• **Competence:** Competency in the dispensing process (22)

		satisfaction with the quality of services of community pharmacies in Vietnam				• **Environment:** Appearance of the pharmacy (1); waiting area (1); private consultation area (1); appearance of the pharmacy (1); dispensing (1)<br>• **Access:** opening hours (3); availability of medicines (2); physical access (1); availability of pharmacist (1)<br>• **Person-centred care:** professionalism (3)
Watson et al. (2018)[29]	UK	Exploration of service providers' attitudes and beliefs of quality and quality improvement in the community pharmacy setting in the UK.	Semi-structured interviews and focus groups	42 service providers. Four focus group discussions were undertaken with 38 pharmacists/pharmacy support staff and semi-structured interviews with four key informants from pharmacy organisations across the UK	• Medicines supply<br>• Professional pharmacy service(Supply of OTC medicines following minor illness consultation)	Quality was described in terms of:<br>• **Person-centred care:** Professionalism: showing empathy Patient-pharmacist relationship: developing rapport. The issue of good continuity of staff was also identified as being associated with better quality because it was believed to be important for developing trust and rapport between patients and pharmacy personnel.<br>• **Competence** Clinical knowledge and diagnostic skills: eliciting specific information during consultations; providing the right information/advice; prompt resolution of symptoms<br>• **Environment**

For peer review only

						<u>Private consultation area:</u> The physical environment of pharmacies such as having a counter was also identified as a potential barrier to asking questions.
Fujita et al. (2019)[55]	Japan	To establish the quality dimensions of home pharmaceutical care (HPC) from the perspectives of home healthcare professionals	Semi-structured interviews and focus groups	Semi-structured interviews and focus groups were carried out with nine home healthcare teams, comprising 61 multidisciplinary professionals including pharmacists, doctors, nurses, care managers, home helpers, medical social workers and other relevant stakeholders involved in home healthcare.	• Professional pharmacy service	• **Environment:** physical resources<br>• **Competence:**<br>*<u>Clinical knowledge and diagnostic skills</u>:* pharmacist factors (professionalism, effectiveness, experience); during home pharmaceutical care (provision of medication review; frequency of visiting home; time spent at home)<br>Impact on patients (humanistic outcomes; clinical outcomes, economic outcomes); impact on other healthcare professional (task shifting, operational efficiency); recognition of benefits of home pharmaceutical care.<br>• **Integration:** <u>Communication mechanisms and information systems:</u> Before home pharmaceutical care (attendance at meetings; collaborative visiting schedule

For peer review only

						arrangements); after home pharmaceutical care (information sharing, timeliness)
Guhl et al (2019)[56]	Germany	Examines the value created by community pharmacies-defined as perceived customer value-in the prescription drug market through varying elements of service quality.	Survey	289 pharmacy users	• Medicines supply	Dimensions covered in the survey:<br>• **Environment:** physical resources (1 item); appearance of the pharmacy (2 items), cleanliness & hygiene (1 item)<br>• **Person-centred care:** professionalism (4 items);<br>• **Access:** Waiting time (1 item); availability of medicines (2 items), opening hours (1 item); physical access (3 items)<br>• **Competence:** Competence in the dispensing process (4 items)<br>• **Safety:** medicine supply (1 item)
Halit et al. (2019)[109]	Lebanon	developing good pharmacy practice(GPP) guidelines to be applied by community pharmacists for services' quality improvement	In January 2018, the OPL Scientific Committee decided to elaborate GPP guidelines for community pharmacists and created the Community Pharmacy		• Medicines supply<br>• Professional pharmacy service (sale of OTC medicines following minor illness consultation)	The GPP standards comprised of sections that addressed the following dimensions:<br>• **Environment:** Appearance of the pharmacy; appearance of the pharmacy; Private consultation area; dispensary.

For peer review only

			Practice Subcommittee, which was in charge of this project. To create the Lebanese GPP guidelines, the committee relied on the guidelines already implemented in several countries and tailored them to the Lebanese situation			Physical resources (availability of a refrigerator and other equipment, equipment status, routine maintenance, Availability of drug information systems; availability of medical devices and complementary medicines)<br><br>• **Access**<br>Availability of medicines: purchasing, storage, and maintenance of quality<br><br>• **Safety:**<br>Compounding; operating procedures, documentation, and raw material handling.<br>Documentation of care: Documentation systems (patient medication profile, formulary systems, policies and standard operating procedures, documentation of interventions)<br><br>• **Competence:**<br>Competence in the dispensing process:<br>Provision of medicines (prescription availability, patient identification, and dispensing).<br>Interaction and communication (communication skills of pharmacist and staff, provision of advice, promotion of good

For peer review only

						health, and provision of written information)<br><br><u>Clinical knowledge and diagnostic skills:</u><br>Supply of OTC medicines (advice on selection and use, responding to minor ailments)<br><br>Health promotion (engagement in health promotion, participation in health promotion campaigns)<br><br>Diagnostics (provision of diagnostic tests, documentation of tests done)<br><br><u>Training of pharmacy staff:</u><br>Research and professional development (participation in research projects, participation in continuing education)<br>Trainees (acceptance of trainees, monitoring and documentation, activity description)<br><br>• **Integration:**<br>Interprofessional collaboration: Development of pharmaceutical care plans, patient monitoring, identification of medication-related problems, interaction with other prescribers, and other healthcare professionals

For peer review only

Hindi et al. (2019)[75]	UK	To examine the views of patients, pharmacists and GPs on how community pharmacies are currently used and to identify how community pharmacy services may be better integrated within the primary care pathway for people with long-term conditions	Focus groups	Two focus groups were conducted with respiratory patients (n=6, 5) and two with type 2 diabetes patients (both= 5). Two focus groups were held with pharmacists (n=7, 5) and two with GPs (both n = 5).	• n/a	• **Integration** Communication mechanisms and information systems: All stakeholder groups believed pharmacists required more access to patient information (i.e. medical records) to have a better overall understanding of patients' conditions.
Hindi et al. (2019)[88]	UK	Identify factors that could influence patients to make better use of community pharmacies within the primary care pathway	Survey	289 Patients with asthma or chronic obstructive pulmonary disease registered at two GP practices.	• Medicines supply<br>• Professional pharmacy service	Questionnaire Items looked at factors influencing patient's likelihood to use community pharmacy services:<br>• **Person-centred care:** Professionalism (3); patient-pharmacist relationship (2)<br>• **Environment:** private consultation area (3)<br>• **Access:** physical access (1)<br>• **Integration:** communication mechanisms and information systems – information sharing (6); proximity (2)
Mirzaei et al (2019)[57]	Australia	To build a theory-grounded model of service quality in community pharmacies and to create a valid survey instrument to	Stage 1 dealt with item generation using theory, prior research and qualitative interviews with		• Medicines supply	Dimensions of service quality covered:<br>• **Person-centred care:** Relationship: Trusting relationship (Relationship)

		measure consumers' perceptions of service quality.	pharmacy consumers. Selected items were then subjected to content validity and face validity. Stages 2 and 3 included psychometric testing among English-speaking adult consumers of Australian pharmacies. Exploratory factor analysis was used for item reduction and to explain the domains of SQ			Professionalism: Friendliness/Helpfulness<br>• **Access:** Availability of the pharmacist, waiting time<br>• **Competence:** Competence in the dispensing process: advice, expertise, effectiveness/knowledge, patient health outcome Clinical knowledge and diagnostic skills: services such as blood pressure testing or diabetes support services were minimally discussed and of least importance to patients<br>• **Environment:** physical resources, appearance of the pharmacy; appearance of the pharmacy.
Patterson et al. (2019)[113]	USA	To measure the relative strength of patient preferences for community pharmacy attributes and to describe associations between patient sociodemographic and health characteristics and pharmacy preferences	Questionnaire (Discrete Choice Experiment)	773 American adults (≥18 years) who had filled a prescription at a pharmacy, other than a mail-order pharmacy, within the last 12 months.	• Medicines supply	Quality dimensions covered:<br>• **Access**: opening hours (1)<br>• **Person-centred care:** professionalism (1); patient-pharmacist relationship (1).<br>• **Competence:** Competence in the dispensing process (2)

For peer review only

Watson et al (2019)[28]	UK	Explored citizens' perspectives about the quality of community pharmacy services in the UK and whether and how the quality of community pharmacy services should be measured.	Semi-structured interviews and focus groups	20 individuals participated (Scotland (n=7) all interviewed individually; England (one focus group (n=4) and four individual interviews); and Wales (one focus group (n=5))	• Medicines supply<br>• Professional pharmacy service (supply of OTC medicines following minor illness consultation)	• **Person-centred care:**<br>Patient experience: Friendly caring service,<br>Patient pharmacist relationship: continuity of care, and staff knowing the individual.<br>Professionalism: Professional approach in customer appearance, including behaviour and appearance<br>• **Environment:**<br>Private consultation area: Physical characteristics of the pharmacy in supporting privacy, with either a separate consultation room or dedicated private area, and the need to have confidential conversations with the pharmacist.
Abu Hagar et al (2020)[58]	UAE	To evaluate the present status of risk<br>occurrence in community pharmacies in Abu Dhabi and investigate the protective plans that are followed in risky<br>cases to generate an overall view of risk management plans	Survey	322 licensed community pharmacists in<br>Abu Dhabi	• Medicines supply	• **Environment**<br>Private consultation area: Provide a private consultation area.<br>Appearance of the pharmacy: temperature should be maintained to avoid discomfort.<br>Appearance of the pharmacy: Sufficient and well distributed lights across<br>• **Safety**<br>Systems for ensuring safety: Keep patient data in separate filing; do not share patient information;

		in concurrent pharmacy practice.				discard documents containing patients' data in a proper way<br>• **Competence**<br>Clinical knowledge and diagnostic skills: The most reported reason for adverse drug reactions was lack of knowledge about side effects (cannot recognize ADR cases to report them)<br>• **Integration**<br>The most reported protective measure to avoid medication errors was contacting the GP
Al-Jumaili et al. (2020)[90]	Iraq	To evaluate patient satisfaction with community pharmacy services and measure the relationships between patient satisfaction and pharmacy/pharmacist characteristics and patient quality of life	Survey	400 patients at 20 community pharmacies in 10 different geographical areas in Baghdad city	• Medicines supply.	The questionnaire included:<br>• **Access:** Availability of pharmacist (1)<br>• **Person-centred care:** professionalism (2), patient-pharmacist relationship (1); patient experience (3)<br>• **Competence:** competence in the dispensing process (11)<br>• **Environment:** appearance of the pharmacy (1); private consultation area (1)
Aizpurua-Arruti et al (2020)[61]	Spain	Confirm if the elderly people who go to the pharmacies still think that the commitments that define the Friendly	Focus group	10 elderly people in San Sebastian	• Medicines supply	• **Person-centred care**<br>Patient experience: Patients valued patient experience based on trust and intimacy

For peer review only

		Pharmacy are the ones previously identified				• **Environment** Private consultation area: Participants highlight the positive aspects of accessible spaces with personalised service areas Waiting area: Participants highlight the positive aspects seating areas that facilitate the wait.<br>• **Competence** Competence in the dispensing process: Patients valued advice on use of medicines<br>• **Integration:** Communication mechanisms and information systems: Patients valued good communication with other health care settings; and referral to health and social care.
Badro et al. (2020)[111]	Lebanon	To assess good pharmacy practice (GPP) aspects and compare GPP scores among community pharmacies in Lebanon, using a tool developed jointly by the International Pharmaceutical Federation (FIP) and the World Health Organization (WHO) to	Survey which included 109 questions	A team of 10 licensed inspectors who work at the Lebanese Order of Pharmacists and visited 276 community<br>pharmacies across Lebanon	• Medicines supply<br>• Professional pharmacy service	The questionnaire was adapted to the Lebanese context and included questions on:<br>• **Safety:** Documentation of care (25); Systems for ensuring safety (5)<br>• **Competence**: competence in the dispensing process (51); clinical knowledge and diagnostic skills (19)

For peer review only - http://bmjopen.bmj.com/site/about/guidelines.xhtml

		improve and maintain standards of pharmacy practice				• **Environment:** appearance of the pharmacy (1); private consultation area (1)<br>• **Access:** availability of medicines (5); physical resources (3); availability of pharmacy staff (1); appearance of the pharmacy (7)
Bratkowska et al. (2020)[89]	Poland	To evaluate patient satisfaction with services provided in independent pharmacies and pharmacy chains in Poland	Survey	117 patients randomly selected from four community pharmacies in Poland (2 chain pharmacies and 2 independent pharmacies)	• Medicines supply	items divided into the following:<br>• **Access:** Waiting time (1); availability of medicines (2)<br>• **Environment:** Appearance of the pharmacy (1); private consultation area (1)<br>• **Competence:** Competency in the dispensing process (6)<br>• **Person-centred care:** Patient-pharmacist relationship (1); patient experience (2); professionalism (1)<br>•
Goto et al (2020)[60]	Japan	To examine how a patient's continuity with the same pharmacist and pharmacy is associated with their evaluation of the quality of pharmacy services.	Questionnaire	3,492 Patients who regularly visited pharmacies	• Medicines supply<br>• Professional pharmacy service	Survey items covered the following dimensions:<br>• **Person-centred care:**<br>Patient experience (3 items); patient-pharmacist relationship (1 item); professionalism (2 items)<br>• **Competence:**

For peer review only

						Competence in the dispensing process (1 item); Clinical knowledge and diagnostic skills (1 item)<br><br>• **Access:**<br><br>Availability of pharmacy staff (1 item), availability of medicines (1 item)
Jacobs et al (2020)[26]	UK	To investigate organisational factors associated with variation in safety climate, patient satisfaction and self-reported medicines adherence in English community pharmacies.	Multivariable regressions were conducted using data from two cross-sectional surveys (1. PSCQ and 2. patient satisfaction with visit)	277 pharmacists and 971 patients visiting 39 pharmacies, across 9 diverse geographical areas.	• Medicines supply<br><br>• Professional pharmacy service	• **Safety:** Safety climate was associated with organisational culture.
Sato et al (2020)[59]	Japan	In Japan, a new system called Health Support Pharmacy (HSP) was introduced in 2016, to promote responsible self-medication with non-prescription medicines and increase awareness of public health activities provided through community pharmacies. This study aimed to identify factors that can impact on the quality of	Semi-structured interviews	Twenty-four community pharmacists from across Japan.	• Professional pharmacy service	• **Pharmacy environment:** physical resources<br><br>• **Competence:**<br><br>Clinical knowledge and diagnostic skills: professional expertise (effectiveness, professionalism, teamwork, scope and duration of expertise).<br><br>Provision of community health education and other events

		HSP services provided by community pharmacists in Japan.				(health promotion techniques, event planning).<br><br>Impact on individuals and the general public (economic outcomes; clinical outcomes humanistic outcomes; health behaviour chance, pharmacy as a community hub); impact on other professional (reassurance and operation efficiency)<br><br>• **Integration**<br>Interprofessional collaboration: interactions between the community and the pharmacy; collaboration with other professionals
Tran et al. (2020)[95]	Vietnam	To understand elderly pharmacy users' satisfaction on the community pharmacy services in Ho Chi Minh City, Vietnam.	Q- methodology	32 pharmacy users, aged over 60, was recruited in four pharmacies in Ho Chi Minh City, Vietnam,	• Medicines supply.	Statements divided into the dimensions of quality:<br><br>• **Competence:** Competency in the dispensing process (22)<br>• **Environment:** Appearance of the pharmacy (1); waiting area (1); private consultation area (1); appearance of the pharmacy (1); dispensing (1)<br>• **Access:** opening hours (3); availability of medicines (2); physical access (1); availability of pharmacist (1)

For peer review only

						• **Person-centred care:** professionalism (3)
Waltering et al. (2020)[105]	Germany	To develop indicators for assessing the quality of medication review in public pharmacies	Delphi	The expert group in the Delphi survey consisted of 22 participants. These were pharmacists, representatives of the health insurance companies, scientific Staff members of various institutes and one member each from an association of panel doctors and a chamber of pharmacists	• Professional pharmacy service	After two rounds of Delphi, a final set of indicators consisting of<br>• **Safety:** Documentation of care (1); Systems for ensuring safety (1)<br>• **Competence:** clinical knowledge and diagnostic skills (4)
Watson et al (2020)[30]	UK	To conceptualise GPs' perceptions and beliefs about the quality of community pharmacy services in general and, more specifically, using the concept of 'always events' and the management of acute consultations.	Semi-structured interviews	20 GPs (Scotland n=12, England n= 8)	• Medicines supply<br>• Professional pharmacy service (sale of OTC medicines following minor illness consultation)	• **Access**<br>Physical access: GPs suggested that pharmacies should be accessible and near to the population that they serve<br>Opening hours: Pharmacies should have extended opening hours for the convenience of patients and known to GPs.<br>Availability of medicines: Most GPs said that pharmacies should hold an adequate, well managed stock of medication (and alternatives) and other medical devices or be able to obtain them quickly.<br>• **Person-centred care:**

						Patient experience: GPs suggested that pharmacists should offer personalised care, with good listening and communication skills and all staff should have a positive orientation to patients/customers. They should involve people in decisions, treat them with sincerity, and always check their understanding of treatment; they should be kind but not "paternalistic".<br><br>• **Competence:** Clinical knowledge and diagnostic skills: pharmacists should identity problems relating to drug efficacy, side effects and compliance.<br><br>• **Environment:** Private consultation area: have at least on dedicated room, or at least a spate/private area, and to take a proactive approach in offering it to patients/customers.<br><br>• **Integration:** Communication mechanisms and information systems: There were mixed views around the recording and sharing patient information.

For peer review only

Abebe et al. (2021)[64]	USA	To characterize documentations in community pharmacies and to examine factors that contribute to perceived documentation of care quality.	Survey	445 community pharmacists in Wisconsin	• Medicines supply	• **Safety** Documentation of care: 20 Survey items covered: Handover procedures; frequency of handovers; Handover training; Handover outcomes; Technology; Handover resolution
Clabaugh et al (2021)[62]	USA	To determine pharmacists' perceptions of working conditions while controlling for respondent (years of experience, degree, work status) and workplace variables (prescription volume, type of community setting).	Survey	1222 pharmacists<br>48 of 50 states	Not specified	Qualitative analysis of survey identified features relative to<br>• **Safety:** Supervision, work design, quality emphasis, group behaviour.<br>• **Person-centred care:** patient expectations.
Thang et al. (2021)[91]	Vietnam	To identify factors that affect the overall satisfaction of customers visiting community pharmacies in Vietnam.	Survey	354 patients at 13 randomly selected community pharmacies in five districts in Hanoi, Vietnam.	• Medicines supply.<br>• Professional pharmacy service (sale of OTC medicines following minor illness consultation)	Questionnaire items covering:<br>• **Person-centred care:** professionalism (2); personalised care (1)<br>• **Competence:** competence in the dispensing process (6); clinical knowledge and diagnostic skills (2)<br>• **Access:** physical location (2); opening hours (1); availability of medicines (1)<br>• **Environment:** appearance of the pharmacy (1); appearance of the

						pharmacy and hygiene (1); private consultation area (1)
Fernandes et al. (2021)[107]	Brazil	To develop an instrument to evaluate the quality of services provided in community pharmacies, as well as to test its application through a geographic information system for the visualization of the results	After a review of the scientific literature, a set of quality indicators was submitted to expert analysis. From the final constructed matrix, observational and self-administered questionnaires were elaborated and applied in pharmacies belonging to a city of the South eastern region of Brazil.		• Medicines supply	Items covered 5 dimensions of quality:<br>• **Access:** opening hours; physical access<br>• **Environment:** Physical resources; dispensary; private consultation area; appearance of the pharmacy<br>• **Competence:** Competency in the dispensing process<br>• **Safety:** Systems for ensuring safety; dispensing
Loo et al (2021)[63]	UK	Explored the content of online feedback provided by patients from across the UK in relation to their experiences of their interaction with pharmacy staff and pharmacy services	Patient stories published on Care Opinion, a national online patient feedback platform, for a one-year period were searched for all content relating to patients' pharmacy experiences.	237 patient stories	• Medicines supply<br>• Professional pharmacy service	Online feedback provided by patients mapped on the following dimensions of quality:<br>• **Person-centred care:** Made up the highest proportion of patient feedback relating to community pharmacy with most feedback being positive. Helpfulness, professionalism, kindness, friendliness, politeness were common terms used to describe pharmacy staff across all settings.

For peer review only - http://bmjopen.bmj.com/site/about/guidelines.xhtml

						• **Competence:** Patient feedback was overall positive regarding the services that pharmacies offer such as healthcare advice, clinical services (e.g. community pharmacy blood pressure checks, minor ailment services) as well as ordering repeat prescriptions and delivery services.<br><br>• **Safety:** Medication errors were only mentioned in community pharmacy related stories whereby medications were mistakenly dispensed.<br><br>• **Access:** Patient stories often related to the accessibility of the pharmacy. Overall, accessibility was positively (28/40, 70%) described by patients. Several stories related to the convenience of community pharmacies attached to or within GP surgeries. Opening times of community pharmacies were appreciated in a small number of stories.<br><br>• **Environment:** A minority of patient feedback related to the environment of the pharmacy and these were mainly negative. Patients complained of crowding within some community pharmacies

For peer review only

Mohamud et al. (2021)[92]	Sudan	To explore patients' satisfaction level with pharmacist's communication, consulting and service delivery qualities.	Survey	385 patients from 229 community pharmacies in Khartoum	• Medicines supply.	Questionnaire items covered:<br>• **Person-centred care:** patient experience (3); professionalism (3)<br>• **Access:** waiting time (1); availability of pharmacy staff (1)<br>• **Competence:** competency in the dispensing process (6)<br>• **Environment:** private consultation area (1)
Nneoma et al. (2021)[106]	Nigeria	To develop quality indicators for assessing pharmaceutical care performance in the Nigerian community pharmacies.	Delphi	A panel of 10 pharmacy experts in Nigeria	• Medicines supply	Indicators categorised under the following dimensions:<br>• **Safety:** Systems for ensuring safety (9); Documentation of care (4); medicine supply (3)<br>• **Competence:** Competency in the dispensing process (5); Training of pharmacy staff (2)
Sepp et al. (2021)[112]	Estonia	To identify the implementation of the Community Pharmacy	Questionnaire	The cross-sectional electronic surveys were conducted among community pharmacies	• Medicines supply	Items covered 4 dimensions of quality:<br>• **Safety:** Documentation of care

For peer review only

		Services Quality Guidelines (CPSQG) as a profession-driven initiative towards improving and harmonizing community pharmacy services in Estonia.		in Estonia in 2014 (N = 478 pharmacies), 2016 (N = 493), and 2019 (N = 494)	• Professional pharmacy service	• **Competence:** Competency in the dispensing process ; clinical and diagnostic skills<br>• **Environment**: Private consultation area indicators; waiting area
Wongvedvanij et al (2021)[65]	Thailand	To investigate how patients perceive different dimensions of service quality, especially for non-prescription medicines during the COVID-19 outbreak.	Survey	378 Thai patients during the spread of COVID-19.	• Professional pharmacy service (sale of OTC medicines following minor illness consultation)	Survey items covered 4 domains of quality:<br>• **Competence**: Clinical knowledge and diagnostic skills (6 items)<br>• **Access:** opening hours (1 item)<br>• **Safety:** documentation of care (2 items)<br>• **Person-centred care**: Patient experience (7 items); professionalism (6 items)
Schomer et al (2022)[66]	USA	This study applied a human factors and ergonomics approach to describe community-based pharmacy personnel perspectives regarding how work environment characteristics affect the ability to perform the duties necessary for optimal patient care and how contributors to	Survey	4606 pharmacists and pharmacy technicians	Not specified	There were 12 items developed for the survey that focused on **safety**

		stress affect the ability to ensure patient safety.				
Wongvedvanij et al (2022)[67]	Thailand	To explore pharmacists' and patients' perception of potential pharmacy service quality for dispensing non-prescription medicines.	Semi-structured interviews	14 pharmacists and 20 patients.	• Professional pharmacy service (sale of OTC medicines following minor illness consultation)	• **Competence:**<br>Clinical knowledge and diagnostic skills:<br>ability, skills, knowledge, expertise to diagnose patient symptoms before dispensing non-prescription medicines, pharmacist must obtain accurate personal information using different communication channels<br>• **Person-centred care:**<br>Patient-pharmacist relationship: pharmacists having ongoing interactions and developing personal relationships with their patients over a period of time. This relationship encompasses mutual trust, loyalty respect and knowledge.<br>Patient experience: Pharmacists pay attention to individual patients and treat them based on their personal health conditions and requirements<br>• **Environment:**<br>Cleanliness & hygiene: clean and hygienic physical space and equipment used to handle pharmaceutical products

For peer review only

Kummer et al (2022)[68]	Serbia	To examine patients' perceptions of an incident that occurred in community pharmacies using CIT and determine recommendations for improving the quality of pharmacy services.	Interviews	20 patients from 3 community pharmacies in Serbia	• Medicines supply	A total of 68 critical incidents were collected and divided into two groups: positive (37) and negative (31), depending on patients' satisfaction/dissatisfaction with community pharmacy services. Critical incidents covered:<br>• **Access:** opening hours, availability of pharmacy staff, physical access, waiting time; availability of medicines<br>• **Competence:** Competence in the dispensing process<br>• **Person-centred care**: professionalism; patient experience<br>• **Safety:** Compounding
Parinyarux et al. (2022)[93]	Thailand	to explore the satisfaction of the community pharmacy users with the facilities and services received from drugstores under the GPP standards and examine the impact of satisfaction toward each GPP domain on overall satisfaction (OS) and the intention to receive the pharmacy services as the first choice in the	Survey	388 community pharmacy users	• Medicines supply.<br>• Professional pharmacy service	Questionnaire items categorised under the following dimensions:<br>• **Safety:** Systems for ensuring safety (2); medicine supply (4)<br>• **Environment:** dispensary (3); private consultation area (1); physical resources (2)<br>• **Competence:** competence in the dispensing process (4); clinical knowledge and diagnostic skills (1)

For peer review only

		case of common and non-serious illnesses (IntR).				• **Person-centred care:** professionalism (4)
Sepp et al (2022)[69]	Estonia	To evaluate to what extent the patient-centred care (PCC) principles are included in the Community Pharmacy Services Quality Guidelines (CPSQG) in Estonia	Deductive content analysis was performed using the PCC framework developed by Santana et al.		• Medicines supply<br>• Professional pharmacy service	• **Access**. This included availability of medicines and more broadly access to diagnostic testing such as Blood pressure blood sugar measurement<br>• **Person-centred care:** Cultivating communication; respectful and compassionate care; engaging patients and managing their care; integration of care

For peer review only

Supplementary file 3: Input from PPI group summarised under the dimensions/sub-dimensions of quality framework

Dimensions	Input from PPI Group	Incorporation of PPI input into framework dimensions
**Patient Experience**	<u>Professionalism:</u><br>- Mannerisms of staff in some pharmacies is poor.<br>- Anyone who has any interaction with customers' needs to be polite.<br>- Staff do not have IDs which makes it hard to distinguish between staff.<br><u>Patient experience:</u><br>- Patients need to be put at the forefront of decision making – where is the patient voice?	<u>Professionalism:</u><br>- *Pharmacy staff being distinguishable by wearing a name badge with their role.*<br>- *Mannerisms of pharmacy staff*<br><u>*Patient Experience:*</u><br>- *Involving patients in decision making*
**Integration**	- Integration is a huge element of quality. No sense that GPs/pharmacists speak to each other. GPs direct patients to pharmacies but pharmacies seem to be unaware. Pharmacies then direct patients back to GPs.<br>.	<u>Communication mechanisms and information systems:</u><br>- *Bi-directional communication between CPs and other providers.*
**Access**	<u>Waiting times</u><br>- Services have gone downhill at community pharmacy. It takes longer to get a prescription.<br><u>Availability of medicines</u><br>- Difficult to encourage public to go to pharmacy for advice if they can't even get medications<br>- Free prescription is not always available<br><u>Availability of pharmacy staff</u><br>- Huge tensions around single pharmacists having to do everything but not accessible to patients.	<u>Waiting times</u><br>- *Importance of waiting time for receiving prescriptions*<br><u>Availability of medicines</u><br>- *Availability of medicines and offering alternatives.*<br><u>Availability of pharmacy staff</u><br>- *Availability of pharmacy staff.*<br>- *Having adequate numbers and appropriately qualified pharmacy staff*

For peer review only

	- Training received by pharmacy staff questionable.<br>- There needs to be more training of pharmacy staff and this needs to be funded.	
**Environment**	Appearance of the pharmacy<br>Unlike GP or dentist, going to community pharmacy is like going to a shop. Not a healthcare environment.<br><br>Private consultation area<br>- Privacy issues – asking details such as address, DOB in front of people.<br>- Providing room in pharmacies for consultations is a good thing	Private consultation area<br>- *The importance of privacy and having a consultation area.*

For peer review only

Supplementary File 4: Summary of key dimensions identified in this review

Dimension	Type
**Access**	
• **Opening hours:** availability of pharmacy services during stated opening hours and extended opening hours	S
• **Waiting time:** minimising wait times for healthcare services	P
• **Availability of pharmacy staff:** *Having adequate numbers and appropriately qualified pharmacy staff for the community pharmacy to operate*	S
• **Physical access:** *parking space near the pharmacy; accessibility for people with special needs (e.g. visually impaired, people with baby carriages); geographical proximity and location (e.g. ease of access of community pharmacies via public transportation; work/home; other healthcare facilities)*	S
• **Availability of medicines:** *maintaining an adequate, well managed stock of essential medicines as well as other medical devices. Pharmacists providing patients with information about alternative medicines and their prices.*	S
**Environment**	
• **Appearance of the pharmacy:** p*harmacy appearing health service orientated by clearly displaying medicines and informational materials. Pharmacy having sufficient counters for dispensing medicines and adequate physical space for pharmacy staff to provide health promotion, education, consultation or screening services to individuals or groups.* Cleanliness & hygiene *of the pharmacy maintained to promote a good impression of the pharmacy.*	S
• **Waiting area:** *Ensuring that the waiting area has sufficient space and seating. Informing patients of waiting times and the reasons for any delays.*	S
• **Dispensary:** *Well organised and spacious designed to ensure efficient processing of prescriptions. Storage shelves clearly labelled with drug classifications and medicines are kept according to the drug classifications*	S
• **Physical resources:** *having drug information systems and resources to ensure provision of additional healthcare services.*	S
• **Private consultation area:** *Having a sufficiently sized dedicated area for consultations in the pharmacy and proactively offering it to patients.*	S
**Competence**	
• **Competence in the dispensing process:** *Accuracy of dispensing; appropriate advice on medication (usage, storage etc.) and non-medication (diet, exercise) aspects; gathering essential patient information as part of the dispensing process.*	P
• **Clinical knowledge and diagnostic skills:** *Having knowledge in disease areas and diagnostic skills to assess patients and provide effective treatment. To be able to assess and refer patients to a GP or other health care provider if necessary.*	P

For peer review only

Person-centred care	
• **Patient experience:** *understanding individual needs, involving patient in decision around medications, tailoring delivery of services to people with special needs or minority groups. Involving patients in decision making*	**P**
• **Patient-pharmacist relationship** *built on trust, friendliness/helpfulness, continuity of care, and availability of the pharmacist.*	**P**
• **Professionalism:** *pharmacy staff treating patients with respect, showing empathy, expressing honest opinions, regarding patient benefit as top priority. Pharmacy staff being distinguishable by wearing a name badge with their role.*	**P**
**Safety**	
• **Compounding:** *Availability of standard operating procedures to ensure accuracy in compounding.*	**S**
• **Dispensing:** *having clear standard operating procedure for checking prescriptions; dispensing medications (particularly high-risk medications); availability of protocols and guidelines for asking patients about potential drug contraindications and drug-drug interactions. Having structured safety protocols for OTC consultations, including safeguarding.*	**S**
• **Systems for ensuring safety:** *having a quality and safety management system in place for: registering errors made during dispensing; handling near-misses and dispensing errors; evaluating patient experiences and recording the number of patient complaints.*	**S**
• **Documentation of care:** Accurate *recording of relevant information such as medical history and use of medication in a way that can be read and interpreted by other healthcare professionals. Ensuring patient personal information is stored in a confidential manner and discarded properly. Ensuring exchange of information, responsibility, and accountability when a pharmacist concludes a shift, and another replaces this outgoing pharmacist at the beginning of a new shift within the same pharmacy*	**P**
**Integration**	
• **Interprofessional collaboration**: *Establishing an active relationship between community pharmacy and wider healthcare team based on a shared understanding of competences, roles and responsibilities.*	**P**
• **Communication mechanisms and information systems:** having predefined and clear ways to communicate with other healthcare providers. Having shared communication systems between community pharmacy and the rest of the healthcare system. Ensuring bi-directional communication with other healthcare providers	**P**

For peer review only

For peer review only

PRISMA 2020 Checklist

Section and Topic	Item #	Checklist item	Location where item is reported
**TITLE**			
Title	1	Identify the report as a systematic review.	p.1
**ABSTRACT**			
Abstract	2	See the PRISMA 2020 for Abstracts checklist.	p.2
**INTRODUCTION**			
Rationale	3	Describe the rationale for the review in the context of existing knowledge.	p.3-5
Objectives	4	Provide an explicit statement of the objective(s) or question(s) the review addresses.	p.5
**METHODS**			
Eligibility criteria	5	Specify the inclusion and exclusion criteria for the review and how studies were grouped for the syntheses.	Table 2
Information sources	6	Specify all databases, registers, websites, organisations, reference lists and other sources searched or consulted to identify studies. Specify the date when each source was last searched or consulted.	p.5, table 1
Search strategy	7	Present the full search strategies for all databases, registers and websites, including any filters and limits used.	Suppl. 1
Selection process	8	Specify the methods used to decide whether a study met the inclusion criteria of the review, including how many reviewers screened each record and each report retrieved, whether they worked independently, and if applicable, details of automation tools used in the process.	p.6
Data collection process	9	Specify the methods used to collect data from reports, including how many reviewers collected data from each report, whether they worked independently, any processes for obtaining or confirming data from study investigators, and if applicable, details of automation tools used in the process.	p.6
Data items	10a	List and define all outcomes for which data were sought. Specify whether all results that were compatible with each outcome domain in each study were sought (e.g. for all measures, time points, analyses), and if not, the methods used to decide which results to collect.	N/A
	10b	List and define all other variables for which data were sought (e.g. participant and intervention characteristics, funding sources). Describe any assumptions made about any missing or unclear information.	N/A
Study risk of bias assessment	11	Specify the methods used to assess risk of bias in the included studies, including details of the tool(s) used, how many reviewers assessed each study and whether they worked independently, and if applicable, details of automation tools used in the process.	N/A
Effect measures	12	Specify for each outcome the effect measure(s) (e.g. risk ratio, mean difference) used in the synthesis or presentation of results.	N/A
Synthesis methods	13a	Describe the processes used to decide which studies were eligible for each synthesis (e.g. tabulating the study intervention characteristics and comparing against the planned groups for each synthesis (item #5)).	Suppl. 3
	13b	Describe any methods required to prepare the data for presentation or synthesis, such as handling of missing summary statistics, or data conversions.	p.7
	13c	Describe any methods used to tabulate or visually display results of individual studies and syntheses.	p.7
	13d	Describe any methods used to synthesize results and provide a rationale for the choice(s). If meta-analysis was performed, describe the model(s), method(s) to identify the presence and extent of statistical heterogeneity, and software package(s) used.	p.7
	13e	Describe any methods used to explore possible causes of heterogeneity among study results (e.g. subgroup analysis, meta-regression).	N/A
	13f	Describe any sensitivity analyses conducted to assess robustness of the synthesized results.	N/A
Reporting bias assessment	14	Describe any methods used to assess risk of bias due to missing results in a synthesis (arising from reporting biases).	N/A
Certainty	15	Describe any methods used to assess certainty (or confidence) in the body of evidence for an outcome.	N/A

For peer review only - http://bmjopen.bmj.com/site/about/guidelines.xhtml

PRISMA 2020 Checklist

Section and Topic	Item #	Checklist item	Location where item is reported
assessment			
**RESULTS**			
Study selection	16a	Describe the results of the search and selection process, from the number of records identified in the search to the number of studies included in the review, ideally using a flow diagram.	Fig. 1
	16b	Cite studies that might appear to meet the inclusion criteria, but which were excluded, and explain why they were excluded.	Fig. 1
Study characteristics	17	Cite each included study and present its characteristics.	p.8-9
Risk of bias in studies	18	Present assessments of risk of bias for each included study.	N/A
Results of individual studies	19	For all outcomes, present, for each study: (a) summary statistics for each group (where appropriate) and (b) an effect estimate and its precision (e.g. confidence/credible interval), ideally using structured tables or plots.	N/A
Results of syntheses	20a	For each synthesis, briefly summarise the characteristics and risk of bias among contributing studies.	Suppl. 3
	20b	Present results of all statistical syntheses conducted. If meta-analysis was done, present for each the summary estimate and its precision (e.g. confidence/credible interval) and measures of statistical heterogeneity. If comparing groups, describe the direction of the effect.	N/A
	20c	Present results of all investigations of possible causes of heterogeneity among study results.	N/A
	20d	Present results of all sensitivity analyses conducted to assess the robustness of the synthesized results.	N/A
Reporting biases	21	Present assessments of risk of bias due to missing results (arising from reporting biases) for each synthesis assessed.	N/A
Certainty of evidence	22	Present assessments of certainty (or confidence) in the body of evidence for each outcome assessed.	N/A
**DISCUSSION**			
Discussion	23a	Provide a general interpretation of the results in the context of other evidence.	p.17-18
	23b	Discuss any limitations of the evidence included in the review.	p.19
	23c	Discuss any limitations of the review processes used.	p.19
	23d	Discuss implications of the results for practice, policy, and future research.	p.17-19
**OTHER INFORMATION**			
Registration and protocol	24a	Provide registration information for the review, including register name and registration number, or state that the review was not registered.	p.20
	24b	Indicate where the review protocol can be accessed, or state that a protocol was not prepared.	p.20
	24c	Describe and explain any amendments to information provided at registration or in the protocol.	N/A
Support	25	Describe sources of financial or non-financial support for the review, and the role of the funders or sponsors in the review.	p.19
Competing interests	26	Declare any competing interests of review authors.	p.19
Availability of data, code and other materials	27	Report which of the following are publicly available and where they can be found: template data collection forms; data extracted from included studies; data used for all analyses; analytic code; any other materials used in the review.	p.20

From: Page MJ, McKenzie JE, Bossuyt PM, Boutron I, Hoffmann TC, Mulrow CD, et al. The PRISMA 2020 statement: an updated guideline for reporting systematic reviews. BMJ 2021;372:n71. doi: 10.1136/bmj.n71

For peer review only

PRISMA 2020 Checklist

For more information, visit: http://www.prisma-statement.org/

For peer review only

Developing a quality framework for community pharmacy: A systematic review of international literature

Journal:	*BMJ Open*
Manuscript ID	bmjopen-2023-079820.R1
Article Type:	Original research
Date Submitted by the Author:	12-Dec-2023
Complete List of Authors:	Hindi, Ali; The University of Manchester, Centre for Pharmacy Workforce Studies, Division of Pharmacy and Optometry<br>Campbell, Stephen; University of Manchester, Centre for Primary Care; Sefako Makgatho Health Sciences University, Department of Public Health Pharmacy and Management, School of Pharmacy<br>Jacobs, Sally; Manchester Univ.<br>Schafheutle, Ellen; The University of Manchester, Stopford Building, Oxford Road, Manchester, Division of Pharmacy, School of Health Sciences, Faculty of Biology Medicine and Health
**Primary Subject Heading**:	Health policy
Secondary Subject Heading:	Health policy, Health services research, Pharmacology and therapeutics, Patient-centred medicine, Research methods
Keywords:	Health policy < HEALTH SERVICES ADMINISTRATION & MANAGEMENT, Quality in health care < HEALTH SERVICES ADMINISTRATION & MANAGEMENT, Systematic Review, Pharmacists, Patient Satisfaction, Primary Care < Primary Health Care

I, the Submitting Author has the right to grant and does grant on behalf of all authors of the Work (as defined in the below author licence), an exclusive licence and/or a non-exclusive licence for contributions from authors who are: i) UK Crown employees; ii) where BMJ has agreed a CC-BY licence shall apply, and/or iii) in accordance with the terms applicable for US Federal Government officers or employees acting as part of their official duties; on a worldwide, perpetual, irrevocable, royalty-free basis to BMJ Publishing Group Ltd ("BMJ") its licensees and where the relevant Journal is co-owned by BMJ to the co-owners of the Journal, to publish the Work in this journal and any other BMJ products and to exploit all rights, as set out in our licence.

The Submitting Author accepts and understands that any supply made under these terms is made by BMJ to the Submitting Author unless you are acting as an employee on behalf of your employer or a postgraduate student of an affiliated institution which is paying any applicable article publishing charge ("APC") for Open Access articles. Where the Submitting Author wishes to make the Work available on an Open Access basis (and intends to pay the relevant APC), the terms of reuse of such Open Access shall be governed by a Creative Commons licence – details of these licences and which Creative Commons licence will apply to this Work are set out in our licence referred to above.

Other than as permitted in any relevant BMJ Author's Self Archiving Policies, I confirm this Work has not been accepted for publication elsewhere, is not being considered for publication elsewhere and does not duplicate material already published. I confirm all authors consent to publication of this Work and authorise the granting of this licence.

For peer review only

Developing a quality framework for community pharmacy: A systematic review of international literature

Authors: Ali M.K. Hindi* (PhD),[1] Stephen M. Campbell (PhD),[2,3] Sally Jacobs (PhD),[1] Ellen I. Schafheutle (PhD)[1]

*Author for correspondence

Full postal address for corresponding author:

1. Centre for Pharmacy Workforce Studies, Division of Pharmacy and Optometry; School of Health Sciences; Faculty of Biology, Medicine and Health, The University of Manchester, Oxford Road, Manchester M13 9PT, United Kingdom

   Email: ali.hindi@manchester.ac.uk

2. School of Health Sciences; Faculty of Biology, Medicine and Health, The University of Manchester, Oxford Road, Manchester M13 9PT, United Kingdom

3. Department of Public Health Pharmacy and Management, School of Pharmacy, Sefako Makgatho Health Sciences University, Molotlegi Street, Pretoria 0208, South Africa

For peer review only - http://bmjopen.bmj.com/site/about/guidelines.xhtml

Abstract

Objective: To identify the defining features of the quality of CP services and synthesise these into an evidence-based quality framework.

Design: Systematic review following the Preferred Reporting Items for Systematic Reviews and Meta-Analyses (PRISMA) guidelines.

Data sources: International research evidence (2005 onwards) identified from six electronic databases (Embase, PubMed, Scopus, CINAHL, Web of Science, PsycINFO) was reviewed systematically from October 2022 - January 2023. Search terms related to "community pharmacy" and "quality".

Eligibility criteria for selecting studies: Titles and abstracts were screened against inclusion/exclusion criteria followed by full-text screening by at least two authors. Qualitative, quantitative, and mixed methods studies relevant to quality in community pharmacy were included.

Data extraction and synthesis: A narrative synthesis was undertaken. Following narrative synthesis, a patient and public involvement event was held to further refine the quality framework.

Results: Following title and abstract screening of 11,493 papers, a total of 81 studies (qualitative and quantitative) included. Of the 81 included studies, 43 investigated quality dimensions and/or factors influencing CP service quality; 21 studies assessed patient satisfaction with and/or preferences for CP; 17 studies reported development/assessment of quality indicators/standards/guidelines for CPs; which can help define quality.

The quality framework emerging from the global literature consisted of six dimensions: person-centred care, access, environment, safety, competence, and integration within local health care systems. Quality was defined as having timely and physical access to personalised care in a suitable environment, which is safe and effective with staff competent in the dispensing process, and pharmacy professionals' possessing clinical knowledge and diagnostic skills to assess and advise patients relative to pharmacists' increasingly clinical roles.

Conclusion: The emerging framework could be used to measure and improve the quality of CP services. Further research and feasibility testing are needed to validate the framework according to local healthcare context.

For peer review only - http://bmjopen.bmj.com/site/about/guidelines.xhtml

Strengths and limitations of this study:

This section should be no more than 5 bullet points relating specifically to the methods - not the results of the study. This will be published as a summary box after the abstract in the final published article.

- This review deployed a comprehensive and systematic search of the international literature which sought to identify defining features of quality of community pharmacy healthcare services and synthesise these into a quality framework.
- For data extraction, a two-step selection process was conducted: two authors (AMKH, SMC) screened all 11,493 papers independently of each other, and the two other authors (SJ, EIS) reviewed all papers with discrepancies and/or queries.
- To ensure relevance of findings to patients, members of the public who use community pharmacy services were consulted on the findings and their feedback was used to further refine the dimensions/sub-dimensions of the quality framework.
- Quality of the papers was critically appraised, but studies were not excluded based on their quality

Background

Faced with growing patient needs, workforce shortages and financial constraints, the necessity for healthcare systems worldwide to focus on delivering “high quality care” and meeting demand for primary care has never been greater with evidence of wide variation in quality between and within countries.[1, 2] Health policy in the past few decades has focused on measuring and improving the quality and safety of healthcare services[3] as well as on improving the quality of care via a wider workforce approach (i.e. distribution of clinical responsibilities between professions) and local integration of health and social care globally[4]. The aim is to improve and strengthen a quality health and care system by joining up planning, commissioning and delivery of health and care services to provide seamless locally based integrated care that meets people’s needs promptly and effectively.[3, 5, 6]

In relation to this, in the past two decades, policymakers have increased the range of healthcare services provided by community pharmacies (CP), over and above their more traditional medicines supply function, to relieve burden on general medical practice and expand capacity within primary care systems.[7] Community pharmacies are accessible and convenient, offering extended and weekend opening hours. Unlike other primary care providers, patients can access community pharmacies without the need for an appointment.

For peer review only - http://bmjopen.bmj.com/site/about/guidelines.xhtml

Hence, community pharmacies are well positioned to improve patient access to care and may assist in reaching patients in deprived areas.[8]

With a view to increasing patient access and choice, healthcare systems worldwide, most notably in countries such as the UK,[9,10] Canada,[11] United States,[12] Australia,[13] and New Zealand,[14] have invested in expanding the range of healthcare (i.e. medicines-related and public health) services offered by CP alongside the sale of over-the-counter (OTC) medicines and other items. However, the quality of some CP services, for example dispensing and medication review services, has been inconsistent.[15-17] Given the increasing range and volume of services provided by CP, it is important to consider how the quality of care can be improved and made equitably accessible. To be able to assess the quality of healthcare provided by CP, an agreed definition and framework are needed.[16]

Different definitions and frameworks of healthcare quality have emerged across healthcare over the years. One of the most influential models stems from Donabedian's structure-process-outcome framework (1980).[18] "Structure" involves the setting of care (e.g. physical facility, human resources, equipment), "process" encompasses the actions taken during service provision (e.g. diagnosis, treatment), and "outcome" is the results of actions taken (e.g. clinical changes to health, patient satisfaction). Donabedian proposed that structure, process, and outcomes are closely linked and influence each other, and his three components are the basis for many quality frameworks.[19-22]

In 2001, the US Institute of Medicine (IOM) developed a health care quality framework which involved six dimensions (i.e. safety, effectiveness, patient-centredness, timely, efficient, and equitable).[23] The IOM's framework has been widely recognised and since its inception different organisations have proposed quality frameworks which often use a combination of these six dimensions. Notably, the Organization for Economic Cooperation and Development (OECD) Health Care Quality Indicators Project (2006)[24] and Lord Darzi's Next Stage Review (2008)[25] defined quality under the three dimensions of safety, effectiveness and patient-centredness. More recently, similar to the IOM's quality framework but also acknowledging the importance of integration, the World Health Organization (WHO) Framework on Integrated People-centred Health Services (2018) described high quality care as care that is safe, effective, people-centred, timely, efficient, equitable and integrated.[3]

Since the early 2000s, definitions of quality in healthcare have been developed and continue to be refined. However, quality is still not well defined in CP,[15, 26] with little known about what quality in CP means or how to measure it.[26] In 2012, Halsall et al. characterized healthcare quality in UK community pharmacy under three dimensions: "accessibility"; "effectiveness"; and "positive perceptions of the experience".[27] More recently, Watson et al.

For peer review only

characterised quality under dimensions of: person-centred; professionalism; and privacy.[28-30] A US based study looking at patients' understanding of what constitutes a "quality pharmacy" identified themes focusing on patient care and trust in pharmacists.[31] However, the dimensions of quality proposed in these studies were mainly related to pharmacists' more traditional role of medicines supply. Furthermore, these studies did not seek to develop a quality framework for CP health service provision as part of an integrated primary healthcare system. As the expansion of community pharmacy away from a primarily medicines supply role and into an extended range of professional services gathers pace,[32] there is a need to shed light on ways community pharmacies could work effectively with other primary care providers to provide better quality healthcare services.

Community pharmacy provides an exemplar of a (part) publicly funded private sector provider, in a mixed market healthcare system. Similar to community pharmacy, quality is poorly defined in other private sector primary care providers such as dentistry[22, 33] and optometry.[34] As stated in the WHO report, *"For if quality of care is not ensured, what is the point of expanding access to care*?".[1] In line with the policy drive to increase patient choice and access to a wider range of services and service providers, it is important to develop a better understanding of quality in these sectors.[10, 35]

> *"We cannot assess quality until we have decided with what meanings to invest the concept. A clear definition of quality is the foundation upon which everything is built". (Donabedian, 1985)*

The **aim of this study** is to identify the defining features of the quality of CP services and synthesise these into an evidence-based CP quality framework.

Methods

This systematic review is reported according to the Preferred Reporting Items for Systematic Reviews and Meta-Analyses (PRISMA) statement.[36]

Search Strategy

Six electronic databases were searched (i.e. Embase, PubMed, Scopus, CINAHL, Web of Science, PsycINFO) using search terms relating to "community pharmacy" and "quality" **(Table 1)**. Specific search strategies for each database are provided in **Supplementary File 1**. Database searches were reviewed with The University of Manchester library's team. In

For peer review only - http://bmjopen.bmj.com/site/about/guidelines.xhtml

addition, references of included studies were scanned for further relevant studies. The search strategy included studies published between 2005 and January 2023. The date limitation, set from 2005 onward, corresponds to the initiation of the revised pharmacy contract in the UK, who are at the forefront of international developments, introducing novel pharmacy services and advancing pharmacist roles.

[INSERT TABLE 1 HERE]

Data screening

A two-step selection process was conducted by two reviewers (AH and SC) independently of each other (conventional double screening). Non-English papers were translated. Titles and abstracts were initially screened against the inclusion/exclusion criteria by AH and SC followed by subsequent full-text screening **(Table 2)**. During the double-screening process, two additional reviewers (SJ and ES) were consulted where there was discrepancy between AH and SC, and/or queries arose.

[INSERT TABLE 2 HERE]

Data extraction and synthesis of results

Data from included papers were extracted using NVivo as a data extraction grid. The process of synthesising the literature was iterative as follows. The first author (AH) initially catalogued the different dimensions/theoretical concepts of quality arising from the literature. Data relevant to quality of community pharmacy healthcare services generated from the literature were then categorised across these identified dimensions of quality. All authors independently assessed each dimension. Iterative revisions were made based on discussions between all authors.

A narrative synthesis was then undertaken by the first author, to provide a descriptive account of both qualitative and quantitative research evidence. Synthesis involved integrating and drawing on findings from studies that addressed: quality dimensions; factors influencing the quality of community pharmacy healthcare services; factors influencing integration of services with wider healthcare system. Synthesis also involved studies which developed quality indicators/standards for community pharmacy, and studies which assessed patient satisfaction with and/or preferences for community pharmacy, when they provide findings of relevance to the aim of the review. As the focus of this review was to synthesise findings into dimensions which are relevant to quality, findings emerging from the data from different methodological approaches were combined to contribute to an emerging quality framework.

For peer review only - http://bmjopen.bmj.com/site/about/guidelines.xhtml

Critical Appraisal

As the included articles used qualitative, quantitative, or mixed methods approaches, different methodological quality assessment tools were employed. Qualitative studies were assessed using JBI checklist for qualitative research. The tool consists of 10-point checklist, each requiring a response of 'Yes' (1), 'No' (0), 'Unclear' (0), 'Not Applicable'.[37] Cross-sectional studies were assessed using JBI checklist for cross sectional studies. The tool consists of an 8-point checklist, each requiring a response 'Yes' (1), 'No' (0), 'Unclear' (0), 'Not Applicable'. [38]

The Mixed Methods Appraisal Tool (MMAT) was employed to evaluate mixed methods studies, enabling the assessment of their methodological quality. Seventeen criteria were considered, each requiring a response Yes = 1, No and Cannot tell = 0. [39] The Conducting and Reporting Delphi Studies (CREDES) checklist was utilised for Delphi studies. It's important to highlight that this checklist primarily serves as a reporting tool rather than a methodological one. Nonetheless, for consistency, we employed a criterion to assess the 9 items on the checklist (Yes = 1, No and Cannot tell = 0). [40]

Quality assessment checklists selection was done by AH and SC. The quality assessment process was carried out by AH who has conducted quality appraisal for two previous published systematic reviews. Overall quality of the literature was evaluated based on the total score for each checklist. Studies were not excluded based on quality, but the score helped to critically appraise findings. Total scores are reported without classification of the studies based on specific quality thresholds as the authors of these tools did not suggest cut-offs,

Patient and Public Involvement

Following synthesis of findings, an online patient and public involvement event was held in April 2023 with seven members of the public who use community pharmacies. These members were recruited via patient charity organisations where the lead author provided a summary of the study with contact details for dissemination. This event was held to ensure the incorporation of the patient perspective in ongoing discussions about quality initiatives in community pharmacy. At the event, a summary of findings was presented by the lead author under the dimensions of the quality framework. Following the presentation, members of the public were asked:

- Do the initial findings make sense?

For peer review only - http://bmjopen.bmj.com/site/about/guidelines.xhtml

- Does the "person-centred care" dimension cover the important aspects of quality in community pharmacy?
- Is there anything important missing from the framework in general?

The event gathered feedback on the dimensions/sub-dimensions of the quality framework emerging from the review. The lead author took notes of the discussion, summarised key points and sent them to participants via email to ensure all important information was captured. Any additional suggestions provided by participants via email were considered. The feedback provided was used to further refine the dimensions/sub-dimensions of the quality framework.

Results

Study selection

A total of 11,493 papers were identified for initial screening after duplicates had been removed. Following title and abstract screening, 165 papers were assessed for eligibility via full-text reading, with 74 studies included in the review. Manual searching of reference lists identified 7 additional studies after eligibility screening **(Figure 1).**

[INSERT FIGURE 1]

For peer review only

Definition of pharmacy services

Multiple terms were used in the literature to describe aspects of community pharmacy practice and healthcare service provision. For consistency, we have broken down community pharmacy healthcare services into 1) medicines supply 2) professional pharmacy services **(Table 3)**.

[INSERT TABLE 3] [41-43]

Some medicines are available to buy without a prescription, commonly referred to as **over the counter (OTC) medicines**. Data from studies which focused on sale/ supply (be that on prescription or in response to a request for sale) of OTC medicines were grouped under "medicines supply". Data from studies which looked at sale/ supply of OTC medicines involving professional/clinical judgement, e.g. as part of a service, were included under "professional pharmacy service".

Study characteristics

Of the 81 studies included in the review, 43 investigated quality dimensions and/or factors influencing the quality of community pharmacy services.[15, 26-30, 44-80] Twenty-one studies assessed patient satisfaction with and/or preferences for community pharmacy.[81-101] Thirteen studies reported development/assessment of quality indicators for community pharmacies.[102-114] Four studies described and defined standards or guidelines for good pharmacy practice which can be used to help define quality.[115-118]

Multiple methods were used including: surveys (n=46);[26, 44, 47, 49, 50, 53, 59, 61, 63, 65, 67, 69-71, 77, 79, 81-85, 87-98, 101-103, 105, 108-114, 117, 118] qualitative interviews (n=9);[15, 30, 48, 52, 64, 72, 73, 76, 86] focus groups;[27, 56, 66, 80] pre- and post-measurement of adherence to standards;[116] biographic and photographic techniques;[45] participant observations;[54] nominal group technique;[46] applying indicators in practice;[106, 107] Q-methodology[99,100] stakeholder event;[115] deductive content analysis;[74] patient stories[68] and mixed methods (n=4).[51, 57, 62, 75] The remaining studies used two or more qualitative methods (n=5),[28, 29, 55, 60, 78] and two or more quantitative methods (n=2).[58 104]

Most of the studies were from UK (n=15),[15, 26-30, 45, 57, 59, 68, 80, 93, 103, 109, 114] USA (n=11),[44, 50, 54, 56, 67, 69, 71, 75, 84, 91, 101] and Australia (n=7).[48, 49, 52, 55, 62, 86, 116] Of the remaining studies, four each were from: Japan,[60, 64, 65, 82] the

For peer review only - http://bmjopen.bmj.com/site/about/guidelines.xhtml

Netherlands,[58, 105-107] Thailand,[70, 72, 98, 108] three each from Germany,[61, 78, 111] Estonia,[74, 81, 118] Iran,[51, 79, 87] Vietnam,[96, 99, 100] two each were from Lebanon,[115 117] UAE,[63, 89] Brazil,[110, 113] and Spain.[66, 76] One each were from Canada,[77] Finland,[102] New Zealand,[46] Lithuania,[47] Malaysia,[90] Poland,[94] Slovenia,[83] Serbia,[73] Sudan,[97] Nigeria,[112] Iraq,[95] Pakistan,[92] and China.[88] One study involved five European countries (Denmark, Germany, Netherlands, Poland, Great Britain) to validate a pan-European questionnaire.[53] One study was conducted amongst three African countries: Ethiopia, Uganda, and Zimbabwe,[104] and another compared questionnaire findings between community pharmacy users in Poland and UK.[85]

Most of the literature explored the views/expectations of community pharmacy staff [15, 26, 27, 29, 45, 47-53, 57-59, 63, 64, 67, 69, 71, 72, 80-83, 102, 105, 106, 109-114, 118] and patients.[26-28, 44, 50, 51, 55, 56, 58, 59, 61, 62, 65, 66, 68, 70, 72, 73, 80-90, 92-101, 109] GPs' views on quality in community pharmacy were explored in seven studies.[46, 51, 57, 60, 80, 83, 109] The views of pharmacy organizations and primary healthcare funders and policy makers were explored in just seven studies.[15, 27, 29, 30, 46, 59, 111, 117] Five studies which developed quality indicators explored the views of pharmacy academics.[102, 103, 105, 111, 113] Summary of study characteristics are provided in **Supplementary File 2,** where they are ordered chronologically.

Critical appraisal of studies

Nine studies were excluded from critical appraisal as their methods were outside the remit of the quality assessment checklists. These included Q methodology,[99, 119] survey tool user guide,[50] assessment of indicator validity through a systematic framework,[74, 104, 106, 107, 116] and a scientific committee meeting for guideline development.[115]

Of the 72 studies that were critically appraised, cross-sectional quantitative studies scored an average of 61%, qualitative studies scored an average of 75%, Delphi studies scored an average of 72%, and mixed methods studies scored an average of 76% **(Supplementary File 3).** However, most cross-sectional studies did not investigate confounding factors. Furthermore, only three[30 76 80] of the twenty-one qualitative studies reported on the influence of the researcher on the research (i.e. reflexivity). Whilst the methods used for all studies were appropriate, only three[103 105 120] of out the nine Delphi studies fully described the stages of the Delphi process, including a preparatory phase, the actual 'Delphi rounds', interim steps of data processing and analysis, and concluding steps. Furthermore, two of the four mixed methods studies excelled in only one aspect of the mixed methods design. For example, Snyder et al.[75] achieved high quality in the qualitative elements but demonstrated

For peer review only - http://bmjopen.bmj.com/site/about/guidelines.xhtml

limitations in the quantitative domain. In contrast, Dadfar et al.[51] scored high in the quantitative aspect but lacked in the qualitative dimension.

Quality framework

Data relevant to identifying concepts and dimensions of quality of care for CP identified from the literature were synthesised and themed under six dimensions (access, environment, competence, person-centred care, safety, integration) to develop a quality framework **(Figure 2)**. The narrative synthesis below is themed under these six dimensions.

[INSERT FIGURE 2]

For peer review only

For peer review only - http://bmjopen.bmj.com/site/about/guidelines.xhtml

Access: structural and procedural components of quality such as opening hours, waiting time, physical access, availability of medicines and availability of pharmacy staff to provide services

Opening hours

Availability of pharmacy services during stated and extended opening hours are commonly identified as key features of quality in CP. [51, 61, 65, 70, 73, 82, 92, 93, 99-101, 113] Patients, pharmacists and GPs suggest that CPs should aim to offer extended opening hours outside regular hours.[27, 28, 30, 56, 80]

Waiting time

Minimal waiting time for pharmacy services (particularly for picking up medicines dispensed on prescriptions) is commonly cited as an important procedural feature of quality of care in CP .[27, 51, 68, 99, 100] Studies exploring the views of patients on quality of care in CP suggest that pharmacies should aim to minimise wait times to get medicines dispensed.[68, 73]

Physical access

Five studies describe "parking space near the pharmacy" as a feature of quality in community pharmacy.[28, 73, 81, 83, 92] Three studies highlight the importance of CPs being accessible for people with special needs such as the elderly, visually impaired, people with baby carriages.[73, 83, 113] Ease of access of community pharmacies via public transportation,[61, 88] work/home,[56, 99] and other healthcare facilities are important features of quality as perceived by patients.[73, 82]

Availability of pharmacy staff

Having adequate numbers and appropriately qualified pharmacy staff is described as a hallmark characteristic of a quality CP.[56, 62, 73, 92, 95, 101, 108] Studies commonly measure the availability of a pharmacist (on-site) to provide advice and answer medication-relates queries.[92, 95, 108, 118] The availability of pharmacy staff on the phone is addressed in two studies.[65, 73]

Availability of medicines

Studies in this review indicate that pharmacies should hold an adequate, well managed stock of medicines as well as medical devices.[64, 82, 115] Studies also emphasise on pharmacies having a stock management system that helps control stock orders and expiry dates and using contingency plans for purchases in an emergency.[82, 106, 108, 112, 117] Furthermore, community pharmacies should have available records for expired drugs, as well as having specific procedures for disposal of expired products.[98, 104, 106, 112, 117]

For peer review only

Patients, pharmacists and GPs highlight the importance of pharmacies maintaining adequate stock and/or being able to obtain medicines quickly, to avoid patients having to return. [26, 30, 62] Patients also perceive reasonable/affordable cost of medications and notification of discounts as an important determinant of CP service quality.[56, 61, 73, 86, 88, 91, 92, 94, 96 100] Patients expect pharmacists to provide them with information about alternative medicines and their prices.[99, 100]

Environment: the impact of facilities, equipment, and pharmacy layout on the quality of healthcare service provision

Appearance of the pharmacy

The appearance of the community pharmacy is an important structural feature of quality health service provision. Studies suggest that community pharmacies need to appear health service orientated by clearly displaying medicines and informational material (such as adverts, leaflets).[83, 84, 98] The pharmacy should also be positioned in a manner which is visible and accessible to patients with clearly defined boundaries. In supermarkets, it should be clear where the general shop or supermarket ends and the pharmacy begins.[45]

Studies also highlight that every pharmacy should have sufficient counters for dispensing medicines[100] and adequate physical space for pharmacy staff to provide professional services (health promotion, education, consultation or screening services to individuals or groups).[45, 108] It is also important to ensure that premises are tidy[51] and lightning of the pharmacy is well distributed.[63]

Cleanliness and hygiene of the pharmacy is commonly highlighted as a feature of quality of care.[62, 88, 92, 94, 96, 100, 108, 115] A few studies specifically mention “ensuring room/air temperature is appropriate”[63, 72] and “avoidance of unpleasant smells”[28, 62, 72, 83] as a means to promote a good first impression of the pharmacy.

Waiting area

Studies suggest that a good quality pharmacy should ensure that the waiting area has sufficient space and seating.[57, 66, 81-83, 87, 100, 118] The importance of informing patients of waiting times and the reasons for any delays was addressed in one study.[57]

Dispensary

Studies suggest that the dispensary should be well organised and spacious designed to ensure efficient processing of prescriptions.[45] Storage shelves/drawers should be clearly labelled with drug classifications and medicines are kept according to the drug

For peer review only

classifications.[108] Pharmacies are required to have a system in place to prevent unauthorized access into areas where controlled drugs are stored.[108,116]

Physical resources (equipment)

Studies highlight the importance of having drug information systems and resources to ensure provision of high quality services.[27, 51, 60, 61, 70, 115, 116] Only two studies specifically mention resources needed to provide professional pharmacy services, such as scales, digital blood pressure monitoring equipment, finger tip sugar equipment.[98, 108]

Private consultation area

Having a private area for consultations is perceived to be a key facilitator for overcoming privacy issues.[28, 30, 45, 46, 55, 56, 63, 66, 80, 92, 93, 96, 98-100, 116-118] Pharmacies without a designated consultation room increase the risk of patient conversations being overheard.[30, 55, 63, 93, 99, 100] Pharmacies in countries such as the UK are required to have at least one dedicated consultation room and it is noted that pharmacists should be proactive in offering it to patients.[30, 45, 80] Relative to pharmacy size, where possible, the room should be spacious, ensuring it is clutter-free and gives the impression of a professional consultation room.[45, 80]

Competence: of pharmacy staff in the dispensing process, pharmacy professionals' clinical knowledge and diagnostic skills to assess and refer patients

Competence in the dispensing process

Pharmacists' ability, knowledge and expertise (i.e. competence) to deliver counselling on prescription medicines is often used to describe quality of health service delivery in community pharmacy. [27, 29, 30, 46, 51, 60, 62, 64, 70, 72, 84, 95, 96, 98, 100, 101, 107, 112, 113, 115, 116] Patients and community pharmacists suggest that providing high quality care requires pharmacists having knowledge and skills to dispense the most effective medicines and provide accurate, clear, and complete information for a specific medicine. [15, 28, 30, 64, 72] Studies also commonly mention speed of dispensing,[92, 112] accuracy of dispensing,[73, 87, 89, 92, 104, 106-108, 117] and gathering essential patient information as elements of an effective dispensing process.[58, 92, 98, 106-108, 117]

Clinical knowledge and diagnostic skills

Only four studies (three of which looked at OTC consultations and one at home care supply) describe competence as knowledge and skills which extends beyond traditional dispensing and medicine supply and are particularly relevant for pharmacists' increasingly clinical roles

For peer review only - http://bmjopen.bmj.com/site/about/guidelines.xhtml

and professional pharmacy services. These studies emphasise on the need for pharmacists to have knowledge in specific disease areas[64] and diagnostic skills to provide effective treatment options with correct instructions for medicine usage and storage.[30, 60, 72] Moreover, GPs expect pharmacists providing professional services to be competent to assess and refer patients to a GP or other health care provider if necessary.[30, 46]

Some studies highlight pharmacy staff needing more opportunities to enhance clinical knowledge via participation in training programs, CPD courses and/or seminars.[15, 49, 82, 108, 112, 115, 118] Making use of all the skill sets of employees (i.e. skill mix) was suggested as important for improving the quantity and quality of professional services in community pharmacy.[15, 46, 80] Upskilling pharmacy technicians to free up pharmacists to move from medicine supply to professional pharmacy services was suggested in one study.[67]

Person-centred care: pharmacy staff providing patients with a positive patient experience; establishing a patient-pharmacist relationship; and demonstrating professionalism at all times

Patient experience

Many studies identified in this review highlight the importance of a positive patient experience when looking at quality of care in CP. A positive patient experience is often described by patients as pharmacists taking the time to understand patients' individual needs and involving patients in decisions around their medications.[15, 30, 44, 46, 47, 56, 65, 70, 73, 80, 96, 99, 100, 109, 113] This includes tailoring delivery of services to people with special needs or minority groups[56, 73, 113] for example by *"adjusting the tone of voice when addressing patients with hearing difficulty"* or *"using capital letters on written materials if the patient has vision problems"*.[73] Patients, pharmacists and GPs perceived sole trader (independent) CPs to provide more personalised care compared to pharmacy chains due to greater pharmacist autonomy in the former. [15, 30, 67, 94]

Professionalism

Professionalism shown by pharmacy staff was perceived by patients as a hallmark feature of good quality service provision. Professionalism encompassed attributes such as courtesy, empathy and trustworthiness.[28, 51, 56, 60, 61, 64, 68, 70, 73, 83, 84, 86, 93-96, 100, 112, 116] Studies suggest that patients expect pharmacy staff to treat them with courtesy and respect and spend as much time as necessary during each encounter.[56, 60, 68, 73, 83, 84, 86, 93-95, 100, 116] However, patients perceive a lack of empathy shown by pharmacy staff to reduce service quality.[56, 73] Patients valued pharmacists expressing honest opinions regarding patient benefit as a high priority.[61, 70, 112] In terms of professional appearance,

For peer review only - http://bmjopen.bmj.com/site/about/guidelines.xhtml

two studies suggest that pharmacists should be distinguishable from the rest of the staff for example by wearing a name badge with their role.[28, 108]

Patient-pharmacist relationship

Studies investigating the views of patients, pharmacists and GPs on community pharmacy frequently cite the patient-pharmacist relationship as an important feature of service quality. Trust, friendliness/helpfulness and availability of the pharmacist have been found to influence the quality of the patient-pharmacist relationship as perceived by patients.[29, 44, 56, 60, 62, 66, 70, 80, 86] Continuity of care (i.e. patients seeing the same pharmacist over time), is perceived to facilitate development of trust and rapport between patients and pharmacists.[26, 29, 30, 86]

Safety: identifying errors and intervening; accuracy in dispensing and compounding; adequate information sharing between pharmacy staff when exchanging shifts; and having systems for ensuring safety

Compounding

Studies suggest labelling of compounded preparations (i.e. preparation of a custom medication) with detailed instructions and clear expiry dates,[73, 115] as well as availability of standard operating procedure (SOPs) to ensure accuracy in compounding.[105-107]

Dispensing

Studies commonly mention ensuring accuracy of dispensing so errors are prevented.[73, 87, 89, 92, 104, 106-108, 117] Identifying and resolving dispensing errors is also seen a key characteristic of good quality health service provision in community pharmacy. This requires pharmacies having clear standard operating procedure (SOPs) for checking prescriptions and dispensing medications (particularly high-risk medications).[106, 107, 109] Studies also suggest having protocols and guidelines for asking patients about potential drug contraindications and drug-drug interactions. [105-107, 112]

Systems for ensuring safety

Recording prescription data and patient information on computer systems to avoid errors and safety incidents are mentioned in included papers.[104,107] The literature also suggests that pharmacies should have an internal quality and safety management system in place for: registering errors made during dispensing, evaluating patient experiences, and recording the number of patient complaints.[105-107, 112] Three studies also highlight the importance of investigating and learning from incidents, education and training about safety, staffing, and management commitment to patient safety. [46, 50, 53]

For peer review only - http://bmjopen.bmj.com/site/about/guidelines.xhtml

Documentation of care

Studies looking at documentation of patient care focus on accurate recording of relevant information such as medical history and medication [30, 64, 70, 115-117] in a way that can be read and interpreted by other healthcare professionals.[113] Furthermore, these studies measure whether patients' personal information is stored and disposed of in a confidential manner.[63, 64, 91]

One study measured handovers defined as "exchange of information, responsibility, and accountability when a pharmacist concludes a shift and another replaces them at the beginning of a new shift within the same pharmacy".[69] The study identified that in almost half of the time, handoffs that occur in a community pharmacy setting were inaccurate or incomplete.[69]

Integration: ways for CP to establish and sustain relationships with the wider healthcare team by having interprofessional collaboration, communication mechanisms and information systems.

Interprofessional collaboration

The ability of community pharmacists to establish a relationship with the local GP was perceived as a fundamental part of community pharmacy integration with the wider healthcare system.[15, 57, 75, 76, 80] Building a relationship required a shared understanding of competences, roles and responsibilities.[77, 78, 80] The perceived benefit of having closer CP-GP working relationships was improved communication, effective signposting and prompt resolution of prescription issues,[15] handling near-misses and dispensing errors, and ensuring errors and near misses are recorded and disused regularly.[106, 107, 109]

Communication mechanisms and information systems

GPs' and community pharmacists' preference for communication methods (e.g. telephone, face-to-face) has been explored but findings are inconclusive.[77, 79] One study highlights that pharmacists express preference for predefined and clear ways to communicate with GPs, given difficulties getting GPs on the phone and receiving an answer to their query.[78] Having a lead responsible for linking GP and CPs is suggested in one study as a potential way to facilitate CP-GP collaboration.[76]

Whether the community pharmacy should have not only read but also write access to shared medical records has been debated. This would allow pharmacists to view relevant information about a patient's medical history to inform their assessment and clinical judgement, enable them to add prescription and medical/ intervention details in the patient's medical record, so

For peer review only

doctors and the wider general practice team are aware.[30, 60, 76, 77, 79, 80, 91, 93] Pharmacists in some studies argue they require better access to patient information to provide safe and effective health care services.[77, 79, 80, 91] Equally, in the UK, patients and GPs have raised concerns over read/write access to medical records, considering the sharing of patient information with commercial organisations, with limited control over who has access, as problematic.[30, 77, 80]

Three Commonwealth studies highlight the importance of having shared communication systems between community pharmacy and the rest of the healthcare system to facilitate community pharmacy integration.[46, 68, 80] In one of these studies, GPs argue that it is difficult to refer patients to community pharmacy given that interactions at community pharmacy are not documented or communicated to them.[80]

Framework refinement based on patient and public involvement

When members of the public were presented with findings and asked for input on dimensions/sub-dimensions of the quality framework emerging from the review, most were dissatisfied with waiting times at CP to collect their medicines. There were tensions around the only pharmacist on site not being accessible to patients.

In addition, the CP retail environment was a perceived barrier to good quality service provision mainly due to privacy issues (e.g. asking details such as address, date of birth in front of customers). All members highlighted the importance of CP staff being professional and distinguishable by wearing a name badge with their role.

Furthermore, integration was seen as a key element of quality where members described the lack of collaboration/communication between GPs and pharmacists. Lastly, members of the public mentioned that CPs are unaware when patients are directed towards them by GPs and vice-versa. This input from PPI group was used to further refine the dimensions/sub-dimensions of the quality framework **(Supplementary file 4)**.

Definition of quality of care in community pharmacy

Based on findings in this review, quality of care in CP can be defined as having timely and physical access to person-centred professional services in a suitable environment, which is safe, integrated and effective. **Supplementary file 5** summarises key dimensions in this review linked to Donabedian's structure-process-outcome components.

For peer review only - http://bmjopen.bmj.com/site/about/guidelines.xhtml

Discussion

In the absence of a universally agreed quality framework looking at health service provision in community pharmacy, this review aimed to collate and synthesise concepts explored in the literature which are relevant to defining quality of care in CP. Upon synthesising the findings of 81 papers, quality was conceptualised by the inter-related dimensions of person-centred care, access, environment, competence, safety, and integration.

The dimensions of quality identified in this review resonate with the IOM's six dimensions of quality,[23] OECD's proposed definition of quality,[24] and the WHO Framework on Integrated People-centred Health Services.[121] The dimensions common to all frameworks were person centeredness, effectiveness, access, and safety. In line with the WHO framework, the framework developed here for quality in CP also included an integration dimension, the importance and relevance of which for community pharmacy is discussed below.[3,122] Unlike these other frameworks, however, "environment" was conceptualised as a separate dimension. Lack of privacy in community pharmacy was commonly highlighted by this review as a barrier to providing high quality healthcare services. The "shop" appearance of community pharmacies and whether premises are fit-for-purpose may prohibit some CPs from meeting all aspects of the framework.[45] One way of being able to ensure privacy when appropriate (e.g for professional services) is having a dedicated consultation area with adequate space.[123]

This review, which adopts a broad view of features of quality of care in CP, draws out important considerations for defining quality community pharmacy to ensure high quality patient care, experience and outcomes. To begin with, CP is one of the most accessible settings in which to receive healthcare services.[17] However, geography alone does not guarantee patients will receive the healthcare services they need. Corroborating findings from this review, previous literature reviews suggest that improving access further involves having adequate staffing levels, strategies for managing medicines supply as well as shortages, and efficient workflow procedures to reduce waiting times.[8, 124-126]

The responsiveness of health systems to the needs of the population is a central pillar of healthcare quality and a crucial perspective is through patients' evaluations of the care they receive.[127] In line with findings from the wider primary and secondary care literature,[128, 129] the person-centred care dimension in this review highlights a positive patient experience, good patient-pharmacist relationship, relational continuity of care, and professionalism as key attributes of quality from a patient perspective. A systematic review looking at a wide range of primary and secondary care settings found that patient experience is positively associated with clinical effectiveness and safety.[128] Moving forward, quality initiatives in CP need to

For peer review only

prioritise collecting patient feedback with emphasis on organisations using that data as one aspect of ongoing quality improvement.

In this review, the competence dimension mainly covered pharmacy staff's ability to effectively perform the dispensing procedure, with dispensing remaining a significant part of community pharmacies, even where (funded) professional services are emerging. Although many studies did not cover professional services, much of the medicine supply process is now expected be performed by the pharmacy support team, and important part of freeing pharmacists' time for professional services. As the scope of professional community pharmacy services continues to expand in many countries, more research is needed to develop quality indicators which consider pharmacy professionals' clinical knowledge and diagnostic skills for providing professional (clinical and public health) services.

The dimensions of access, person-centred care, competence, and environment mirrored those of existing CP frameworks by Halsall[27] and Watson.[28-30] However, compared to previous studies conceptualising quality in CP, the "integration" dimension was unique in our framework. Six studies synthesised in this review and PPI members describe community pharmacy integration within the wider healthcare system as an important dimension of a quality framework. Our study suggests that an integration dimension needs to consider interprofessional collaborations and information sharing between community pharmacy and other primary care providers such as general practice. The 'interprofessional collaboration' element of our integration dimension resembles Valentijin's taxonomy of integrated primary care[130] where the term 'professional integration' is used to describe "*inter-professional partnerships based on shared competences, roles, responsibilities and accountability to deliver a comprehensive continuum of care to a defined population*". The communication mechanisms and information systems of our integration dimension closely aligns with Valentijin's 'functional integration', defined as "*key support functions and activities (i.e. financial, management and information systems) structured around the primary process of service delivery, to coordinate and support accountability and decision making between organisations and professionals to add overall value to the system".*[130]

To the authors' knowledge, this is the first systematic review of the international literature which sought to identify defining features of the quality of community pharmacy healthcare services and synthesise these into a quality framework. The framework emerging from this review contributes to knowledge of improving access to, and the healthcare of, the population through privately owned businesses which provide publicly funded primary healthcare services. The strength of this paper is the comprehensive and systematic search of the international literature deployed by the lead author (AH) with conventional double screening by an expert in quality of care (SC). Furthermore, an expert in community pharmacy policy

For peer review only - http://bmjopen.bmj.com/site/about/guidelines.xhtml

research (ES) reviewed all papers at the full-text review stage where there were disagreements/ uncertainty between AH and SC. Another expert in community pharmacy policy research (SJ) undertook this process on all papers where discrepancies remained. Moreover, input from public contributors was used to further refine the dimensions/sub-dimensions of the quality framework. In terms of limitations, only one author critically appraised the findings due to time constraints. Given this review sought to develop a broad framework covering different dimensions of healthcare quality, the word “integration” was not used as a key word in the search strategy which could explain the low number of papers identified relative to integration.

Conclusion

This review defines quality of CP and provides a dimensional framework of quality of CP services consisting of six dimensions: patient experience, access, environment, safety, competence, integration. As CP expands in the UK and other countries beyond a primarily medicines supply function, the quality dimensions need to be validated and refined locally with a particular emphasis on integration. Integration is particularly relevant for professional services, where roles and responsibilities for joined-up services are shared across primary care providers, making collaboration and two-directional information sharing particularly important. Once quality dimensions are validated and refined, the next step will be using the framework to develop and feasibility test summative “quality assurance” and formative “quality improvement” mechanisms.

Funding statement: This work was funded by The National Institute for Health and Care Research (NIHR) School of Primary Care (Grant reference:**C066)**.

Competing interest: None.

Acknowledgements: We would like to thank The National Institute for Health and Care Research (NIHR) School of Primary Care for funding the fellowship We would also like to thank our patient and public contributors for providing their input on the quality framework emerging from this systematic review.

Contributors: AH, ES, SC and SJ conceptualised the study.

AH ran database searches, title and abstract screening. AH and SC undertook independent full-text review. ES reviewed all papers at the full-text review stage, where there were disagreements/ uncertainty between AH and SC; SJ undertook this process on all papers where discrepancies remained. All authors discussed and agreed inclusion and exclusion criteria, and judgements on all papers at the full-text review stage, to reach a final decision.

For peer review only - http://bmjopen.bmj.com/site/about/guidelines.xhtml

AH ran the data extraction process which was refined by SC, SJ and ES. AH facilitated the patient and public involvement event, which SJ co-facilitated.

AH wrote the first full draft of the manuscript. All co-authors reviewed and discussed drafts iteratively, providing critical and intellectual contributions to analysis, interpretation, and framing.

Ethics Approval: Not required

Patient consent form: As formal consent form is not required for PPI events, PPI contributors were informed of the purpose of the study and their contribution, and their consent was confirmed verbally.

Data sharing statement: Data are available on reasonable request, by contacting the corresponding author.

Registration and protocol: The review was not registered.

References

1. World Health Organization. Delivering quality health services: a global imperative for universal health coverage. 2019. Available: https://www.who.int/publications/i/item/9789241513906
2. Schneider EC, Shah A, Doty MM, et al. Mirror, Mirror 2021 — Reflecting Poorly: Health Care in the U.S. Compared to Other High-Income Countries: The Commonwealth Fund, 2021. Available: https://www.commonwealthfund.org/sites/default/files/2021-08/Schneider_Mirror_Mirror_2021.pdf
3. World Health Organization. Handbook for national quality policy and strategy: A practical approach for developing policy and strategy to improve quality of care. 2018. https://www.who.int/publications-detail-redirect/9789241565561
4. World Health O. Global strategy on human resources for health: workforce 2030. Geneva: World Health Organization. Available from: https://apps.who.int/iris/bitstream/handle/10665/250368/9789241511131-eng.pdf
5. Department of Health & Social Care. Joining up care for people, places and populations: The government's proposals for health and care integration, 2022. Available from: https://www.gov.uk/government/publications/health-and-social-care-integration-joining-up-care-for-people-places-and-populations/health-and-social-care-integration-joining-up-care-for-people-places-and-populations
6. World Health Organization. MONITORING THE BUILDING BLOCKS OF HEALTH SYSTEMS: A HANDBOOK OF INDICATORS AND THEIR MEASUREMENT STRATEGIES. 2010. Available from: https://apps.who.int/iris/bitstream/handle/10665/258734/9789241564052-eng.pdf
7. The Pharmaceutical Group of the European Union. PHARMACY 2030: A Vision for Community Pharmacy in Europe. Available from: https://www.pgeu.eu/wp-

For peer review only - http://bmjopen.bmj.com/site/about/guidelines.xhtml

content/uploads/2019/04/Pharmacy-2030_-A-Vision-for-Community-Pharmacy-in-Europe.pdf.

8. Pantasri T. Expanded roles of community pharmacists in COVID-19: A scoping literature review. *J Am Pharm* 2022;62:649-57. doi: https://doi.org/10.1016/j.japh.2021.12.013

9. PSNC. Community Pharmacy: FORWARD VIEW. 2016. Available from: https://cpe.org.uk/wp-content/uploads/2016/08/CPFV-Aug-2016.pdf

10. GOV UK. New pharmacy referral service to help patients avoid hospital readmission 2020. Available from: https://www.gov.uk/government/news/new-pharmacy-referral-service-to-help-patients-avoid-hospital-readmission.

11. Raiche T, Pammett R, Dattani S, et al. Community pharmacists' evolving role in Canadian primary health care: a vision of harmonization in a patchwork system. *Pharm Pract (Granada)* 2020;18:2171. doi: 10.18549/PharmPract.2020.4.2171

12. Salgado TM, Rosenthal MM, Coe AB, et al. Primary healthcare policy and vision for community pharmacy and pharmacists in the United States. *Pharm Pract (Granada)* 2020;18:2160. doi: 10.18549/PharmPract.2020.3.2160

13. Dineen-Griffin S, Benrimoj SI, Garcia-Cardenas V. Primary health care policy and vision for community pharmacy and pharmacists in Australia. *Pharm Pract (Granada)* 2020;18:1967. doi: 10.18549/PharmPract.2020.2.1967

14. Smith AJ, Scahill SL, Harrison J, et al. Service provision in the wake of a new funding model for community pharmacy. *BMC Health Serv Res* 2018;18:307. doi: 10.1186/s12913-018-3120-z

15. Jacobs S, Fegan T, Bradley F, et al. How do organisational configuration and context influence the quantity and quality of NHS services provided by English community pharmacies? A qualitative investigation. *PLOS ONE* 2018;13:e0204304. doi: 10.1371/journal.pone.0204304

16. Hann M, Schafheutle EI, Bradley F, et al. Organisational and extraorganisational determinants of volume of service delivery by English community pharmacies: a cross-sectional survey and secondary data analysis. *BMJ Open* 2017;7. doi: 10.1136/bmjopen-2017-017843

17. Valliant SN, Burbage SC, Pathak S, et al. Pharmacists as accessible health care providers: quantifying the opportunity. *J Manag Care Spec Pharm* 2022;28:85-90. doi: 10.18553/jmcp.2022.28.1.85

18. Donabedian A. The Definition of Quality and Approaches to Its Assessment. Ann Arbor: Health Administration Press 1980.

19. Campbell SM, Roland MO, Buetow SA. Defining quality of care. *Soc Sci Med* 2000;51:1611-25. doi: 10.1016/s0277-9536(00)00057-5

20. Santana MJ, Manalili K, Jolley RJ, et al. How to practice person-centred care: A conceptual framework. *Health Expect* 2018;21:429-40. doi: 10.1111/hex.12640

21. Lau P, Ryan S, Abbott P, et al. Protocol for a Delphi consensus study to select indicators of high-quality general practice to achieve Quality Equity and Systems Transformation in Primary Health Care (QUEST-PHC) in Australia. *PLoS One* 2022;17:e0268096. doi: 10.1371/journal.pone.0268096

22. Campbell S, Tickle M. What is quality primary dental care? *Br Dent J* 2013;215:135-9. doi: 10.1038/sj.bdj.2013.740

23. Institute of Medicine Committee on Quality of Health Care in A. Crossing the Quality Chasm: A New Health System for the 21st Century. Crossing the Quality Chasm: A New Health System for the 21st Century. Washington (DC): National Academies Press (US)

For peer review only - http://bmjopen.bmj.com/site/about/guidelines.xhtml

24. Arah OA, Westert GP, Hurst J, et al. A conceptual framework for the OECD Health Care Quality Indicators Project. *Int J Qual Health Care*2006;18(suppl_1):5-13. doi: 10.1093/intqhc/mzl024

25. Darzi A. High Quality Care For All: NHS Next Stage Review Final Report, 2008. Available from: https://assets.publishing.service.gov.uk/government/uploads/system/uploads/attachment_data/file/228836/7432.pdf

26. Jacobs S, Hann M, Bradley F, et al. Organisational factors associated with safety climate, patient satisfaction and self-reported medicines adherence in community pharmacies. *Res Social Adm Pharm* 2020;16:895-903. doi: https://dx.doi.org/10.1016/j.sapharm.2019.09.058

27. Halsall D, Noyce PR, Ashcroft DM. Characterizing healthcare quality in the community pharmacy setting: Insights from a focus group study. *Res Social Adm Pharm* 2012;8:360-70. doi: https://dx.doi.org/10.1016/j.sapharm.2011.09.001

28. Watson MC, Silver K, Watkins R. How does the public conceptualise the quality of care and its measurement in community pharmacies in the UK: A qualitative interview study. *BMJ Open* 2019;9:e027198. doi: https://dx.doi.org/10.1136/bmjopen-2018-027198

29. Watson MC, Skea ZC. Jugglers and tightrope walkers: The challenge of delivering quality community pharmacy services. *PLOS ONE* 2018;13:e0200610. doi: 10.1371/journal.pone.0200610

30. Watson M, Silver K, Watkins R. "What counts can't always be measured": a qualitative exploration of general practitioners' conceptualisation of quality for community pharmacy services. *BMC Fam Pract* 2020;21:244. doi: https://dx.doi.org/10.1186/s12875-020-01319-2

31. Warholak TL, Patel M, Rosenthal M, et al. Patient perceptions of a pharmacy star rating model. *J Am Pharm* 2017;57:311-17. doi: https://doi.org/10.1016/j.japh.2017.01.011

32. Piquer-Martinez C, Urionagüena A, Benrimoj SI, et al. Integration of community pharmacy in primary health care: The challenge. *Res Social Adm Pharm* 2022;18:3444-47. doi: https://doi.org/10.1016/j.sapharm.2021.12.005

33. Byrne MJ, Tickle M, Glenny AM, et al. A systematic review of quality measures used in primary care dentistry. *Int Dent J* 2019;69:252-64. doi: 10.1111/idj.12453

34. National Academies of Sciences E, Medicine, Health, et al. The National Academies Collection: Reports funded by National Institutes of Health. In: Welp A, Woodbury RB, McCoy MA, et al., eds. Making Eye Health a Population Health Imperative: Vision for Tomorrow. Washington (DC): National Academies Press (US)

35. Royal College of General Practitioners. Fit for the Future: A vision for general practice. 2019. Available from: https://www.rcgp.org.uk/getmedia/ff0f6ea4-bce1-4d4e-befc-d8337db06d0e/RCGP-fit-for-the-future-report-may-2019.pdf

36. Page MJ, McKenzie JE, Bossuyt PM, et al. The PRISMA 2020 statement: an updated guideline for reporting systematic reviews. *BMJ* 2021;372:n71. doi: 10.1136/bmj.n71

37. Lockwood C, Munn Z, Porritt K. Qualitative research synthesis: methodological guidance for systematic reviewers utilizing meta-aggregation. *JBI Evidence Implementation* 2015;13(3):179-87. doi: 10.1097/xeb.0000000000000062

38. Moola S, Munn Z, Sears K, et al. Conducting systematic reviews of association (etiology): The Joanna Briggs Institute's approach. *Int J Evid Based Healthc* 2015;13(3):163-9. doi: 10.1097/xeb.0000000000000064

39. Hong QN, Pluye P, Fàbregues S, et al. Improving the content validity of the mixed methods appraisal tool: a modified e-Delphi study. *J Clin Epidemiol* 2019;111:49-59.e1. doi: 10.1016/j.jclinepi.2019.03.008

For peer review only

40. Jünger S, Payne SA, Brine J, et al. Guidance on Conducting and REporting DElphi Studies (CREDES) in palliative care: Recommendations based on a methodological systematic review. *Palliat Med* 2017;31(8):684-706. doi: 10.1177/0269216317690685

41. PSNC. NHS Community Pharmacy Contractual Framework Essential Service – Dispensing [Available from: https://psnc.org.uk/wp-content/uploads/2013/07/service20spec20es12020dispensing20_v1201020oct2004_.pdf.

42. Paulino E, Thomas D, Lee SWH, et al. Chapter 8 - Dispensing Process, Medication Reconciliation, Patient Counseling, and Medication Adherence. In: Thomas D, ed. Clinical Pharmacy Education, Practice and Research: Elsevier 2019:109-20.

43. Moullin JC, Sabater-Hernández D, Fernandez-Llimos F, et al. Defining professional pharmacy 43. Moullin JC, Sabater-Hernández D, Fernandez-Llimos F, et al. Defining professional pharmacy services in community pharmacy. *Res Social Adm Pharm* 2013;9:989-95. doi: https://doi.org/10.1016/j.sapharm.2013.02.005

44. Worley MM. Testing a pharmacist-patient relationship quality model among older persons with diabetes. *Res Social Adm Pharm* 2006;2:1-21. doi: 10.1016/j.sapharm.2005.12.006

45. Rapport F, Doel MA, Jerzembek GS. "Convenient space" or "a tight squeeze": insider views on the community pharmacy. *Health & place* 2009;15:315-22. doi: 10.1016/j.healthplace.2008.06.002

46. Scahill SL, Harrison J, Carswell P. What constitutes an effective community pharmacy?--development of a preliminary model of organizational effectiveness through concept mapping with multiple stakeholders. *Int J Qual Health Care* 2010;22:324-32. doi: 10.1093/intqhc/mzq033

47. Urbonas G, Jakušovaitė I, Savickas A. Pharmacy specialists' attitudes toward pharmaceutical service quality at community pharmacies. *Medicina (Kaunas)* 2010;46:686-92.

48. White L, Clark C. Stakeholders' views of service quality in community pharmacy: a qualitative study. *Health Mark Q* 2010;27(1):48-65. doi: 10.1080/07359680903519750

49. Feletto E, Wilson LK, Roberts AS, et al. Building capacity to implement cognitive pharmaceutical services: Quantifying the needs of community pharmacies. *Res Social Adm Pharm* 2010;6:163-73. doi: 10.1016/j.sapharm.2009.08.003

50. AHRQ. Community Pharmacy Survey on Patient Safety Culture, 2012. Available at: www.ahrq.gov/qual/patientsafetyculture/pharmsurvindex.htm.

51. Dadfar H, Brege S. Differentiation by improving quality of services at the last touch point. *Int J Qual Serv* 2012;4:345-63. doi: 10.1108/17566691211288331

52. White L, Klinner C. Service quality in community pharmacy: an exploration of determinants. *Res Social Adm Pharm* 2012;8:122-32. doi: 10.1016/j.sapharm.2011.01.002

53. Phipps DL, De Bie J, Herborg H, et al. Evaluation of the Pharmacy Safety Climate Questionnaire in European community pharmacies. *Int J Qual Health Care* 2012;24:16-22. doi: https://dx.doi.org/10.1093/intqhc/mzr070

54. Odukoya OK, Chui MA, Pu J. Factors influencing quality of patient interaction at community pharmacy drive-through and walk-in counselling areas. *Int J Pharm Pract* 2014;22:246-56. doi: 10.1111/ijpp.12073

For peer review only

55. Hattingh HL, Knox K, Fejzic J, et al. Privacy and confidentiality: perspectives of mental health consumers and carers in pharmacy settings. *Int J Pharm Pract* 2015;23:52-60. doi: 10.1111/ijpp.12114

56. Shiyanbola OO, Mott DA, Croes KD. The structural and process aspects of pharmacy quality: older adults' perceptions. *Int J Clin Pharm* 2016;38:96-106. doi: 10.1007/s11096-015-0211-3

57. Weiss MC, Grey E, Harris M, et al. Dispensing doctor practices and community pharmacies: exploring the quality of pharmaceutical services. *Prim Health Care Res Dev* 2016;17:42-55. doi: 10.1017/s1463423615000092

58. Koster ES, Blom L, Overbeeke MR, et al. Quality of pharmaceutical care at the pharmacy counter: patients' experiences versus video observation. *Patient Prefer Adherence* 2016;10:363-9. doi: 10.2147/ppa.S102032

59. Newlands RS, Power A, Young L, et al. Quality improvement of community pharmacy services: a prioritisation exercise. *Int J Pharm Pract* 2018;26:39-48. doi: 10.1111/ijpp.12354

60. Fujita K, Kushida K, Moles RJ, et al. Home healthcare professionals' perspectives on quality dimensions for home pharmaceutical care in Japan. *Geriatr Gerontol Int* 2019;19:35-43. doi: 10.1111/ggi.13562

61. Guhl D, Blankart KE, Stargardt T. Service quality and perceived customer value in community pharmacies. *Health Serv Manage Res* 2019;32:36-48. doi: 10.1177/0951484818761730

62. Mirzaei A, Carter SR, Chen JY, et al. Development of a questionnaire to measure consumers' perceptions of service quality in community pharmacies. *Res Social Adm Pharm* 2019;15:346-57. doi: 10.1016/j.sapharm.2018.05.005

63. Abu Hagar R, El-Dahiyat F, El Refae G. Risk management in community pharmacy practice in Abu Dhabi Region: a cross-sectional study. *J Pharm Health Serv Res* 2020;11:275-85. doi: 10.1111/jphs.12364

64. Sato N, Fujita K, Kushida K, et al. Exploring the factors influencing the quality of "Health Support Pharmacy" services in Japan: Perspectives of community pharmacists. *Res Social Adm Pharm* 2020;16:1686-93. doi: 10.1016/j.sapharm.2020.02.012

65. Goto K, Sato T, Shikamura Y, et al. Examining the association between the "My Pharmacist" model and the service quality of community pharmacies. *Res Social Adm Pharm* 2020;16:958-66. doi: 10.1016/j.sapharm.2019.10.012

66. aizpurua arruti x. Confirmación de los compromisos de una Farmacia Amigable. *Pharmaceutical Care España* 2020;22:25-33.

67. Clabaugh M, Newlon JL, Illingworth Plake KS. Perceptions of working conditions and safety concerns in community pharmacy. *J Am Pharm Assoc (2003)* 2021;61:761-71. doi: 10.1016/j.japh.2021.06.011

68. Loo J, Greaves G, Lewis PJ. Exploring patients' pharmacy stories: an analysis of online feedback. *Int J Clin Pharm* 2021;43:1584-93. doi: 10.1007/s11096-021-01287-2

69. Abebe E, Stone JA, Lester CA, et al. Quality of Handoffs in Community Pharmacies. *J Patient Saf* 2021;17(6):405-11. doi: 10.1097/pts.0000000000000382

70. Wongvedvanij R, Darawong C. Patients' perception toward the service quality of community pharmacies for non-prescription medicines during the COVID-19 pandemic. *Int J Healthc Manag* 2022:1-7. doi: 10.1080/20479700.2022.2105900

71. Schommer JC, Lee S, Gaither CA, et al. Improving the Experience of Providing Care in Community-Based Pharmacies. *Pharmacy (Basel)* 2022;10(4) doi: 10.3390/pharmacy10040067

For peer review only

72. Wongvedvanij R, Darawong C. Enhancing the service quality of community pharmacies for non-prescription medicines based on the perspectives of pharmacists and patients: a qualitative study. *J Commun Healthc* 2022:1-8. doi: 10.1080/17538068.2022.2026057

73. Kummer I, Mudric J, Cikaric T, et al. Patients' perception of the quality of community pharmacy services using the critical incident technique. *Vojnosanitetski Pregled* 2022;79:605-12. doi: https://dx.doi.org/10.2298/VSP200518123K

74. Sepp K, Cavaco A, Volmer D. The principles of person-centredness in quality patient care-Evaluation of the Community Pharmacy Services Quality Guidelines in Estonia. *Int J Health Plann Manage* 2022;37 Suppl 1:101-14. doi: 10.1002/hpm.3567

75. Snyder ME, Zillich AJ, Primack BA, et al. Exploring successful community pharmacist-physician collaborative working relationships using mixed methods. *Res Social Adm Pharm* 2010;6:307-23. doi: 10.1016/j.sapharm.2009.11.008

76. Rubio-Valera M, Jové AM, Hughes CM, et al. Factors affecting collaboration between general practitioners and community pharmacists: a qualitative study. *BMC Health Serv Res* 2012;12:188. doi: 10.1186/1472-6963-12-188

77. Kelly DV, Bishop L, Young S, et al. Pharmacist and physician views on collaborative practice: Findings from the community pharmaceutical care project. *Can Pharm J (Ott)* 2013;146:218-26. doi: 10.1177/1715163513492642

78. Löffler C, Koudmani C, Böhmer F, et al. Perceptions of interprofessional collaboration of general practitioners and community pharmacists - a qualitative study. *BMC Health Serv Res* 2017;17:224. doi: 10.1186/s12913-017-2157-8

79. Hashemian F, Emadi F, Roohi E. Collaboration between pharmacists and general practitioners in the health care system in the Islamic Republic of Iran. *East Mediterr Health J* 2016;22:375-82. doi: 10.26719/2016.22.6.375

80. Hindi AMK, Schafheutle EI, Jacobs S. Community pharmacy integration within the primary care pathway for people with long-term conditions: a focus group study of patients', pharmacists' and GPs' experiences and expectations. *BMC Fam Pract* 2019;20 doi: 10.1186/s12875-019-0912-0

81. Villako P, Raal A. A survey of Estonian consumer expectations from the pharmacy service and a comparison with the opinions of pharmacists. *Pharm World Sci* 2007;29:546-50. doi: 10.1007/s11096-007-9102-6

82. Sakurai H, Nakajima F, Tada Y, et al. An investigation on pharmacy functions and services affecting satisfaction of patients with prescriptions in community pharmacies. *Yakugaku Zasshi* 2009;129:581-91. doi: 10.1248/yakushi.129.581

83. Horvat N, Kos M. Slovenian pharmacy performance: a patient-centred approach to patient satisfaction survey content development. *Int J Clin Pharm* 2011;33:985-96. doi: 10.1007/s11096-011-9572-4

84. Patterson BJ, Doucette WR, Urmie JM, et al. Exploring relationships among pharmacy service use, patronage motives, and patient satisfaction. *J Am Pharm Assoc (2003)* 2013;53:382-9. doi: 10.1331/JAPhA.2013.12100

85. Merks P, Kaźmierczak J, Olszewska AE, et al. Comparison of factors influencing patient choice of community pharmacy in Poland and in the UK, and identification of components of pharmaceutical care. *Patient Prefer Adherence* 2014;8:715-26. doi: 10.2147/ppa.S53829

For peer review only

86. McMillan SS, Sav A, Kelly F, et al. How to attract them and keep them: the pharmacy attributes that matter to Australian residents with chronic conditions. *Int J Pharm Pract* 2014;22:238-45. doi: 10.1111/ijpp.12075

87. Mehralian G, Rangchian M, Rasekh HR. Client priorities and satisfaction with community pharmacies: the situation in Tehran. *Int J Clin Pharm* 2014;36:707-15. doi: 10.1007/s11096-014-9928-7

88. Chen Y, Fu FQ. The behavioral consequences of service quality: an empirical study in the Chinese retail pharmacy industry. *Health Mark Q* 2015;32:14-30. doi: 10.1080/07359683.2015.1000706

89. Alhomoud F, Kunbus A, Ameer A, et al. Quality Assessment of community pharmacy services provided in the United Arab Emirates: patient experience and satisfaction. *J Appl Pharm Sci* 2016;6:17-23. doi: 10.7324/JAPS.2016.60303

90. Nilugal KC, Kaur MJ, Molugulu N, et al. Patients' attitudes and satisfaction towards community pharmacy in Selangor, Malaysia. *Le Pharmacien Hospitalier et Clinicien* 2016;51:210-21. doi: https://doi.org/10.1016/j.phclin.2015.08.005

91. Feehan M, Walsh M, Godin J, et al. Patient preferences for healthcare delivery through community pharmacy settings in the USA: A discrete choice study. *J Clin Pharm Ther* 2017;42:738-49. doi: 10.1111/jcpt.12574

92. Aziz MM, Ji W, Masood I, et al. Patient Satisfaction with Community Pharmacies Services: A Cross-Sectional Survey from Punjab; Pakistan. *Int J Environ Res Public Health* 2018;15 doi: 10.3390/ijerph15122914

93. Hindi AMK, Schafheutle EI, Jacobs S. Applying a whole systems lens to the general practice crisis: Cross-sectional survey looking at usage of community pharmacy services in England by patients with long-term respiratory conditions. *BMJ Open* 2019;9:e032310. doi: https://dx.doi.org/10.1136/bmjopen-2019-032310

94. Bratkowska K, Religioni U, Krysiński J, et al. Quality of Pharmaceutical Services in Independent Pharmacies and Pharmacy Chains in Poland from the Patient Perspective. *Patient Prefer Adherence* 2020;14:2459-67. doi: 10.2147/ppa.S284014

95. Al-Jumaili AA, Ameen IA, Alzubaidy DA. Influence of Pharmacy Characteristics and Customer Quality of Life on Satisfaction of Community Pharmacy Customers. *Innov Pharm* 2020;11 doi: 10.24926/iip.v11i1.2434

96. Thang D, Thuy L, Tien L, et al. Factors impacting on customer satisfaction with community pharmacies in vietnam. *Res Social Adm Pharm* 2018;14:e55-e56. doi: 10.1016/j.sapharm.2018.05.123

97. Mohamud A, Shire F, Hussain K, et al. Assessing Patient Satisfaction with the Service Quality Provided in Community Pharmacies: Khartoum Locality, Sudan. *J Young Pharm* 2021;13:405-10. doi: 10.5530/jyp.2021.13.98

98. Parinyarux P, Yotsombut K. Customers' satisfaction toward drugstore facilities and services based on the good pharmacy practice standard in Thailand. *Pharm Pract (Granada)* 2022;20:2601. doi: 10.18549/PharmPract.2022.1.2601

99. Tran VD, Dorofeeva V. Applying Q-methodology to study customer satisfaction with quality of community pharmacy services in Vietnam. *Trop J Pharm Res* 2018;17:2281-89. doi: 10.4314/tjpr.v17i11.25

For peer review only

100. Tran VD, Dorofeeva VV, Loskutova EE, et al. Elderly consumers' satisfaction with the quality of community pharmacy services in Ho Chi Minh City, Vietnam: a Q-methodology study. *Pharmacia* 2020;67(4):303-09.

101. Patterson JA, Holdford DA, Harpe SE. Patient preferences for objective quality metrics during community pharmacy selection: A discrete choice experiment. *Res Social Adm Pharm* 2019;15(6):641-49. doi: 10.1016/j.sapharm.2018.08.002

102. Puumalainen, II, Kause JM, Airaksinen MS. Quality assurance instrument focusing on patient counseling. *Ann Pharmacother* 2005;39:1220-6. doi: 10.1345/aph.1E629 [published

103. James DH, Hatten S, Roberts D, et al. Identifying criteria for assessing the quality of medicines use review referral documentation by community pharmacists. *Int J Pharm Pract* 2008;16:365-74. doi: https://dx.doi.org/10.1211/ijpp.16.6.0005

104. Trap B, Hansen E, Trap R, et al. A new indicator based tool for assessing and reporting on good pharmacy practice. *Southern Med Review* 2010;3:4-11.

105. De Bie J, Kijlstra NB, Daemen BJG, et al. The development of quality indicators for community pharmacy care. *BMJ Qual Safe* 2011;20:666. doi: 10.1136/bmjqs.2010.045237

106. Schoenmakers TW, Teichert M, Braspenning J, et al. Evaluation of quality indicators for Dutch community pharmacies using a comprehensive assessment framework. *J Manag Care Spec Pharm* 2015;21(2):144-52. doi: 10.18553/jmcp.2015.21.2.144

107. Teichert M, Schoenmakers T, Kylstra N, et al. Quality indicators for pharmaceutical care: a comprehensive set with national scores for Dutch community pharmacies. *Int J Clin Pharm* 2016;38:870-9. doi: 10.1007/s11096-016-0301-x

108. Arkaravichien W, Wongpratat A, Lertsinudom S. Quality indicators to compare accredited independent pharmacies and accredited chain pharmacies in Thailand. *Int J Clin Pharm* 2016;38:899-907. doi: 10.1007/s11096-016-0307-4

109. Grey E, Harris M, Rodham K, et al. Characteristics of good quality pharmaceutical services common to community pharmacies and dispensing general practices. *Int J Pharm Pract* 2016;24:311-8. doi: 10.1111/ijpp.12253 [published Online First: 2016/03/05]

110. Júnior E, Halila G, Reis W, et al. Quality indicators of pharmacists' services in community pharmacies in Paraná State, Brazil. *Braz J Pharm Sci* 2017;53 doi: 10.1590/s2175-97902017000116021

111. Waltering I, Scheppe S, Kurth V, et al. [Quality indicators for medication reviews in community pharmacies]. *Zeitschrift fur Evidenz, Fortbildung und Qualitat im Gesundheitswesen* 2020;153-154:44-53. doi: 10.1016/j.zefq.2020.03.007

112. Okpalanma N, Obinna E. DEVELOPMENT OF QUALITY INDICATORS FOR PHARMACEUTICAL CARE PERFORMANCE ASSESSMENT IN NIGERIAN COMMUNITY PHARMACIES: A DELPHI PANEL. *Int J Pharm Sci* 2021:50-55. doi: 10.22159/ijpps.2021v13i2.39179

113. Fernandes B, Freitas R, Melchiors A, et al. Proposal of a quality assessment tool intended for community pharmacies with the use of a geographic information system. *Revista de Gestão em Sistemas de Saúde* 2021;10:253-74. doi: 10.5585/rgss.v10i3.17863

114. Harding G, Wilcock M. Community pharmacists' perceptions of medicines use reviews and quality assurance by peer review. *Pharm World Sci* 2010;32:381-5. doi: 10.1007/s11096-010-9381-1

115. Hallit S, Sacre H, Sarkis H, et al. Good Pharmacy Practice Standardized for Community Pharmacists: The Lebanese Order of Pharmacists Initiative. *J Res Pharm Pract* 2019;8:29-32. doi: 10.4103/jrpp.JRPP_18_96

For peer review only

116. Benrimoj SI, Gilbert AL, de Almeida Neto AC, et al. National implementation of standards of practice for non-prescription medicines in Australia. *Pharm World Sci* 2009;31:230-7. doi: 10.1007/s11096-008-9266-8

117. Badro DA, Sacre H, Hallit S, et al. Good pharmacy practice assessment among community pharmacies in Lebanon. *Pharm Pract (Granada)* 2020;18:1745. doi: 10.18549/PharmPract.2020.1.1745

118. Sepp K, Cavaco AM, Raal A, et al. Profession Driven Improvement of the Quality of Pharmacy Practice-Implementation of Community Pharmacy Services Quality Guidelines in Estonia. *Healthcare (Basel, Switzerland)* 2021;9 doi: 10.3390/healthcare9070804

119. De Tran V, Dorofeeva VV, Loskutova EE, et al. Elderly consumers' satisfaction with the quality of community pharmacy services in Ho Chi Minh City, Vietnam: a Q-methodology study. *Pharmacia* 2020;67:303-09. doi: https://dx.doi.org/10.3897/pharmacia.67.e56511

120. Waltering I, Scheppe S, Kurth V, et al. Quality indicators for medication reviews in community pharmacies. *Zeitschrift fur Evidenz, Fortbildung und Qualitat im Gesundheitswesen* 2020;153-154:44-53. doi: https://dx.doi.org/10.1016/j.zefq.2020.03.007

121. World Health Organization. Regional Office for E, European Observatory on Health S, Policies, et al. Improving healthcare quality in Europe: characteristics, effectiveness and implementation of different strategies. Copenhagen: World Health Organization. Regional Office for Europe 2019:419 p.

122. World Health Organization. Framework on integrated, people-centred health services: Report by the Secretariat, 2016.

123. Sim TF, Wright B, Hattingh L, et al. A cross-sectional survey of enhanced and extended professional services in community pharmacies: A pharmacy perspective. *Res Social Adm Pharm* 2020;16(4):511-21. doi: 10.1016/j.sapharm.2019.07.001

124. Alam S, Osama M, Iqbal F, et al. Reducing pharmacy patient waiting time. *Int J Health Care Qual Assur* 2018;31:834-44. doi: 10.1108/IJHCQA-08-2017-0144

125. Patel PM, Vaidya V, Osundina F, et al. Determining patient preferences of community pharmacy attributes: A systematic review. *J. Am. Pharm. Assoc* 2020;60:397-404. doi: https://doi.org/10.1016/j.japh.2019.10.008

126. Hindi AMK, Jacobs S, Schafheutle EI. Solidarity or dissonance? A systematic review of pharmacist and GP views on community pharmacy services in the UK. *Health Soc Care Community* 2019;27:565-98. doi: 10.1111/hsc.12618

127. Manary MP, Boulding W, Staelin R, et al. The Patient Experience and Health Outcomes. *N Engl J Med* 2012;368:201-03. doi: 10.1056/NEJMp1211775

128. Cathal D, Laura L, Derek B. A systematic review of evidence on the links between patient experience and clinical safety and effectiveness. *BMJ Open* 2013;3:e001570. doi: 10.1136/bmjopen-2012-001570

129. Nadia S, Claire G-S, Susan EB, et al. Quality indicator framework for primary care of patients with dementia. *Can Fam Physician* 2022;68:e270. doi: 10.46747/cfp.6809e270

130. Valentijn PP, Vrijhoef HJM, Ruwaard D, et al. Towards an international taxonomy of integrated primary care: a Delphi consensus approach. *BMC Fam Pract* 2015;16:64. doi: 10.1186/s12875-015-0278-x

For peer review only

Tables

Table 1: Search strategy

Concept	Key terms*
Healthcare quality	“Quality” OR “healthcare quality” OR “quality of health care” OR “quality improvement” OR “quality assessment” OR “quality assurance”
AND	“Community pharmacy” OR “retail pharmacy”
Community pharmacy	

*Different wildcards and truncations were used depending on the database

Table 2: Inclusion and exclusion criteria

Inclusion criteria	Exclusion criteria
**Setting:** Community pharmacy	non-community pharmacy setting
**Design/Study type:** Empirical studies	**Design/Study type:** Literature reviews.
**Location:** All regions	
**Publication date:** 2005 onwards	
**Publication type:**<br>Peer reviewed journal papers,<br>Reports on QI indicator development	**Publication type:**<br>Conference abstracts.<br>Commentary/opinion pieces /editorials<br>Reviews
**Focus of study:**<br>• Definitions/dimensions of quality in community pharmacy (including patient experience, environment, safety).<br>• Development/assessment of quality indicators/standards for community pharmacy healthcare services<br>• Patient satisfaction with community pharmacy healthcare services<br>• Factors influencing quality of care in	**Focus of study:**<br>• Advancing the scope of pharmacists and/or pharmacy technicians in practice<br>• Integrating pharmacists/pharmacy technicians in other healthcare settings.<br>• Pilot community pharmacy interventions/services<br>• Evaluations of individual services<br>• Impact of training<br>• Evaluations of pay-for-performance

For peer review only - http://bmjopen.bmj.com/site/about/guidelines.xhtml

community pharmacy	schemes<br><br>• Assessing approaches to measure quality (e.g. quality inspection reports, quality cards, administrative claims)

Table 3: Definition of pharmacy healthcare services

**Medicines supply**	*"the time between when the prescription is received by the pharmacy and the prescribed medicine(s) is supplied to the patient".[41]*<br><br>The dispensing process involves:<br>○ Receiving and validating the prescription<br>○ Assessing and reviewing the prescribed medicine<br>○ Selecting/preparing, packaging and checking the medicine<br>○ Labelling<br>○ Supplying and counselling the patients<br>○ Recording the intervention.[42]
**Professional pharmacy services**	*"A professional pharmacy service is an action or set of actions undertaken in or organized by a pharmacy, delivered by a pharmacist or other health practitioner, who applies their specialized health knowledge personally or via an intermediary, with a patient/client, population or other health professional, to optimize the process of care, with the aim to improve health outcomes and the value of healthcare".[43]*

For peer review only

Figure Legend

Figure 1: Flow diagram demonstrating the search procedure

Figure 2: Overview of quality dimensions emerging from the literature

For peer review only

For peer review only - http://bmjopen.bmj.com/site/about/guidelines.xhtml

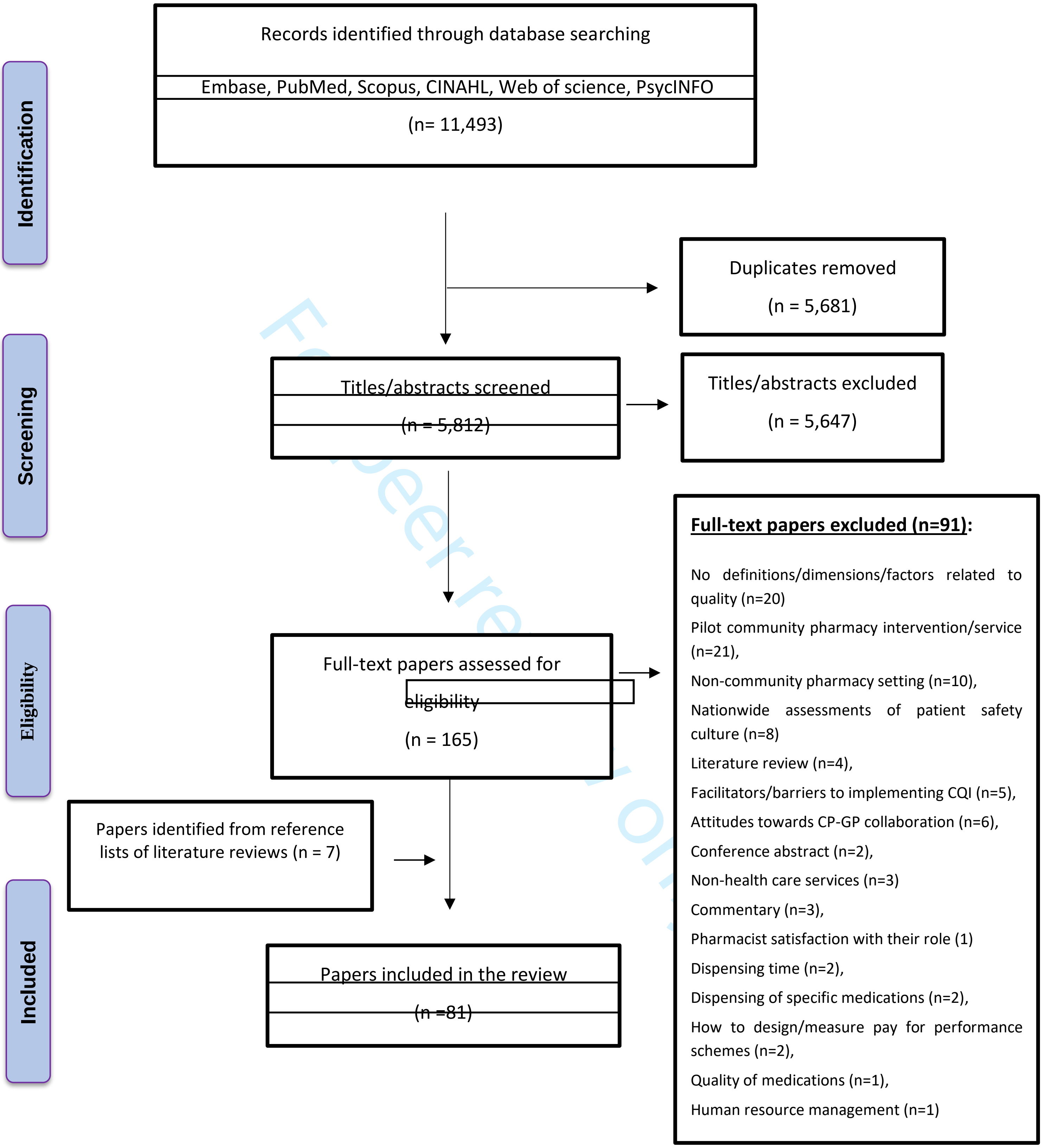

Figure 1: Flow diagram demonstrating the search procedure

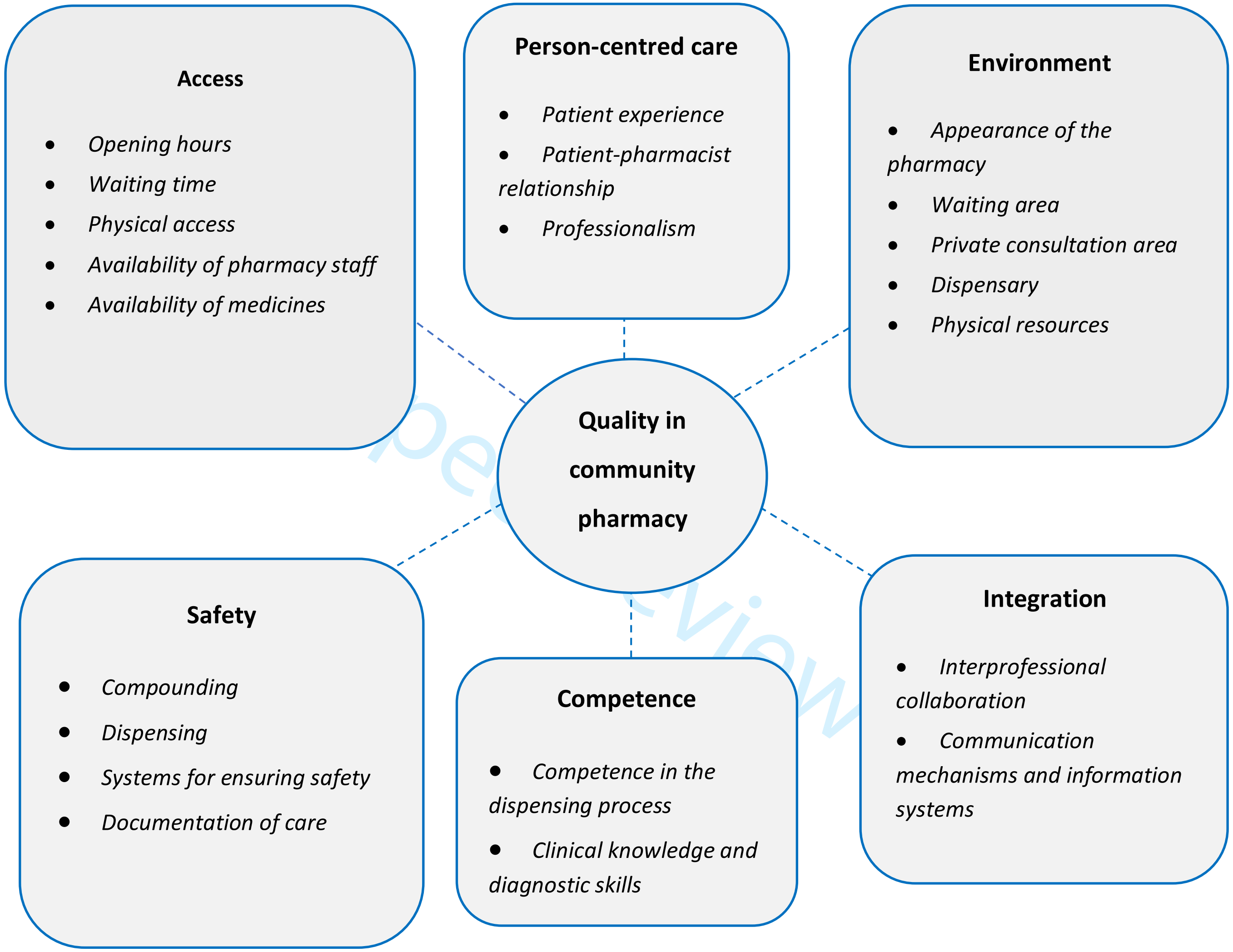

Figure 2: Overview of quality dimensions emerging from the literature

Supplementary File 1 – Database searches

PubMed		
Search	Query	Items found
#1	"quality"[Title/Abstract]	1,287,702
#2	"healthcare quality"[Title/Abstract]	3,376
#3	"health care quality"[Title/Abstract]	3,827
#4	assessment, healthcare quality[MeSH Terms]	349,704
#5	"quality assurance"[Title/Abstract]	29,169
#6	"quality assessment"[Title/Abstract]	27,324
#7	"quality improvement"[Title/Abstract]	49,716
#8	"quality of health care"[Title/Abstract]	5,355
#9	"quality of healthcare"[Title/Abstract]	1,915
#10	#1 OR #2 OR #3 OR #4 OR #5 OR #6 OR #7 OR #8 OR #9	1,569,851
#11	"retail pharmac*"[Title/Abstract]	767
#12	"community pharmac*"[Title/Abstract]	8,652
#13	"community pharmacy services"[MeSH Terms]	5,564
#14	"pharmacies"[MeSH Terms]	9,347
#15	#11 OR #12 OR #13 OR #14	18,246
#16	#10 AND #15<br>from 2005 - 2022	**2,317**

Scopus		
Search	Query	Items found
#1	TITLE-ABS-KEY (quality)	4,526,623
#2	TITLE-ABS-KEY ("community pharmac*")	14,435
#3	TITLE-ABS-KEY ("retail pharmac*")	1,182
#4	#8 OR #9	15,432
#5	#1 AND #4<br>from 2005 – 2022	**2,327**

*Using TITLE-ABS-KEY “quality” captures: TITLE-ABS-KEY ("quality improvement") TITLE-ABS-KEY ("quality assessment") TITLE-ABS-KEY ("quality assurance") TITLE-ABS-KEY (quality W/3 care)

Embase		
Search	Query	Items found
#1	(quality adj5 care).ab,kw,ti.	148,342
#2	"health care quality".mp. or health care quality/	264,992
#3	"healthcare quality".mp.	4243
#4	"quality improvement".ab,kw,ti.	77715
#5	"quality assessment".ab,kw,ti.	34799
#6	"quality assurance".ab,kw,ti.	44810
#7	quality.ab,kw,ti.	175,1710
#8	#1 or #2 or #3 or #4 or #5 or #6 or #7	191,6548
#9	community pharmacy/ or "community pharmac*".mp.	23925
#10	"retail pharmac*".ab,kw,ti.	1422
#11	#9 OR #10	24978
#13	#8 AND #11<br>from 2005-2022	**3,631**

**CINAHL**		
Search	Query	Items found
#1	(MH "Quality of Care Research") OR (MH "Quality of Health Care") OR (MH "Quality Improvement") OR (MH "Quality Assessment") OR (MH "Quality Assurance") OR "quality"	603,463
#2	"community pharmac*"	6,041
#3	(MH "Pharmacy, Retail") OR ""retail pharmac*""	8,275
#4	#2 OR #3	11,488
#5	#1 AND #4<br>Publication year 2005-2022	**1,302**

- Using “quality” as a keyword captures (MH "Quality of Care Research") OR (MH "Quality of Health Care") OR (MH "Quality Improvement") OR (MH "Quality Assessment") OR (MH "Quality Assurance")

For peer review only

PsycINFO		
Search	Query	Items found
#1	exp "Quality of Services"/ or quality.mp. or exp "Quality of Care"/	316648
#2	"community pharmac*".mp.	1245
#3	"retail pharmac*".mp. [mp=title, abstract, heading word, table of contents, key concepts, original title, tests & measures, mesh word]	143
#4	2 or 3	1366
#5	1 and 4<br>Specific range 2005-2022	**207**

- Mapping the search term “quality” to “quality of services” and “quality of care”, covers: (quality adj5 care) OR "quality assessment" OR "quality improvement" OR "quality assurance"

Web of Science		
Search	Query	Items found
#1	quality (Topic)	3,205,809
#2	"community pharmac*" (Topic)	10,595
#3	"retail pharmac*" (Topic)	823
#4	#2 OR #3	11,310
#5	#1 AND #4<br>Publication years 2005-2022	**1,794**

- *Using “quality (Topic)” covers:* (quality) NEAR/3 (care or healthcare) (Topic) OR "quality assessment" (Topic) OR "quality improvement" (Topic) OR "quality assurance" (Topic)

For peer review only

Author(s) & year*	Country	Purpose	Study design	Sample	Pharmacy service(s)	Key findings mapped under quality dimensions
Puumalainen et al. (2005)[102]	Finland	To develop a validated, easy-to-use patient counselling quality assurance instrument for community pharmacists.	Delphi	2 expert panels: Panel 1, consisting of experienced pharmacy practitioners (n = 10), and Panel 2, consisting of academic and professional experts (n = 10).	• Medicines supply	• **Person-centred care:** Patient experience: Indicators that enable practitioners to understand patients' specific information and communication needs.<br>• **Competence:** Competence in the dispensing process: indicators relevant to technical skills in dispensing prescriptions; incorporation of patient counselling in the dispensing process.<br>• **Environment:** Physical resources: availability and accessibility of information sources (manual, electronic); guidelines on patient counselling practices in the pharmacy; guidelines on patient counselling with local healthcare personnel
Worley (2006)[44]	USA	To test a pharmacist-patient relationship quality model in a group of older persons with diabetes from the	Survey	311 community-dwelling older persons (65 years of age and older), using at least one prescription medication and primarily	• Professional pharmacy service	• **Person-centred care** Patient-pharmacist relationship: contains measurement items from 5 study constructs -

For peer review only - http://bmjopen.bmj.com/site/about/guidelines.xhtml

		patient's perspective. Pharmacist-patient relationship commitment was the outcome of relationship quality studied.		obtaining their prescriptions from some type of nonmail order pharmacy		1. Pharmacist participative behaviour/patient-centeredness of relationship<br>2. Patient participative behaviour<br>3. Pharmacist-patient interpersonal communication<br>4. Relationship quality<br>5. Relationship commitment
Vilako et al. (2007)[81]	Estonia	To assess the preferences of pharmacy customers when choosing a pharmacy and their expectations of the service and comparing these with the opinions of pharmacists.	Survey	Patients (n=1979) in cities (in 3 community pharmacies), towns (in 2 community pharmacies), and in small towns (in 2 community pharmacies). The survey was also carried out among community pharmacists (n=135) in different regions of Estonia.	• Not specified	Survey items:<br>• **Access**<br>Physical access: Parking space near pharmacy; Comfortable entering to pharmacy<br>Availability of medicines: wide choice of products<br>Waiting time: Quick service<br>• **Environment**<br>Waiting area: Comfortable staying in pharmacy.<br>Private consultation area: privacy and discretion<br>• **Competence**<br>Clinical knowledge and diagnostic skills: Professional consulting;

For peer review only

						Help with choosing right medicine
James et al. (2008)[103]	UK	To develop explicit criteria against which the quality of Medicine Use Review (i.e. MUR; a planned patient-pharmacist consultation to discuss medicines use) referral documentation can be assessed.	Delphi	Sixteen panellists (these were 10 out of 14 MUR accreditation tutors who were invited to take part and six pharmacy practitioners from a possible 22 primary care organisations in Wales)	• Professional pharmacy service	• **Safety** Documentation of care: consensus was achieved for 20 quality indicators that correspond with that of documenting a patient-pharmacist consultation.
Benrimoj et al. (2009)[116]	Australia	To implement nationally a quality improvement package in relation to the Standards of Practice for the Provision of Non-Prescription Medicines.	Randomly selected pharmacies were coached on the implementation of the Standards of Practice for the Provision of Non-Prescription Medicines. Pre and post measurements of the level of adherence to the Standards were taken.	2,706 pharmacies	• Medicines supply<br>• Professional pharmacy service (supply of OTC medicines following minor illness consultation)	Standards used in this study defined and described the professional activities required for the provision of medicines at a consistent and measurable level of practice.<br><br>**Environment:** Appearance of the pharmacy (3 statements); Physical resources (1 statement); Private consultation area (1 statement)<br><br>**Person-centred care:** Professionalism (3 statements)<br><br>**Competence:** Competence in dispensing process/clinical knowledge and diagnostic skills (8 statements)<br><br>**Safety:** Documentation of care (2 statements); Systems for

For peer review only

						ensuring safety and safety. (1 statement)
Rapport et al. (2009)[45]	UK	Identifying the extent to which pharmacy spaces are aligned to good professional practice, enhance a professional's sense of self and meet the demands of the public.	Mixed-methods approach employing biographic and photographic techniques	16 pharmacists	• Medicines supply<br>• Professional pharmacy service	• **Environment**<br>Dispensary: Essential to be well organised and to have control over the space and the way it functions. Barriers for dispensing to function in an orderly fashion: unwanted interruptions, undesired observation, lack of formality, lack of room and changes to the order and running of things brought about by others'<br><br>Private consultation area: Some have reservations about its size and positioning. In the smaller settings, particularly within Independent and dedicated pharmacies, consultation rooms are shoehorned into an already limited workspace, bringing additional pressure overflow storage.<br><br>Appearance of the pharmacy: Brings into question the pharmacist's position and professional status. The sales counter is particularly problematic when it comes to distractions with members of the public attempting to overcome

For peer review only

						the divide between pharmacist, dispensary and sales floor.
Sakurai et al. (2009)[82]	Japan	To investigate how pharmacy functions and services affect patient satisfaction	Survey	30186 Patients from 178 pharmacies whose purpose of use of the pharmacies was not only for prescription dispensing but also OTC medicines	• Medicines supply<br>• Sale of OTC medicines following minor illness consultation	Survey items:<br>• **Access:**<br>Opening hours: opening hours<br>Physical access: location<br>Waiting time: average waiting time; maximum waiting time<br>Availability of medicines: amount of pharmaceutical stock<br>• **Environment:**<br>Private consultation area: Privacy considerations<br>Waiting area: number of waiting chairs<br>Physical resources: number of blood pressure, bone density and other measuring instruments
Feletto et al. (2010)[49]	Australia	To determine the needs of pharmacies that were important and the elements requiring improvement when implementing and delivering cognitive pharmaceutical services.	Survey	355 community pharmacies	• Professional pharmacy service	Survey items covered the following areas:<br>• **Access:** opening hours (1)<br>• **Environment:** Private consultation area (3); appearance of the pharmacy (2)<br>• **Competence:** training of pharmacy staff (2)<br>• **Integration** (1)

For peer review only

Harding et al. (2010)[114]	UK	To explore existing mechanism to ensure quality assurance of medicine use reviews (MURs), and to identify those parameters of an MUR that community pharmacists consider as indicators of quality.	Survey	50 pharmacists, a third of which were from locum pharmacists.	• Professional pharmacy service	Individual survey items were not provided but the analysis of findings were mapped on the quality dimensions:<br>• **Competence:** <u>Clinical knowledge and diagnostic skills:</u> The single most frequently reported determinant for undertaking an MUR was the pharmacists' judgement (84% n = 42). Over 70% (n = 35) of respondents considered that undertaking MURs required specialist skills
Scahill et al. (2010)[46]	New Zeeland	To develop a multi-constituent model of organizational effectiveness for community pharmacy.	Face to face brainstorming to generate statements describing what constitutes an effective community pharmacy, and sorting of the statements into themes with rating of each statement for importance	14 stakeholders representing policy-makers and health care providers including; community pharmacy, professional pharmacy organizations, primary health care funders and policy-makers, general practitioners and general practice support organizations	• Medicines supply<br>• Professional pharmacy service	• **Safety:** statements addressed how an organisation could promote safe and effective workflow; and ensure safe use of medicines.<br>• **Integration:** statements addressed how an organisation could focus on patient needs; and better integrate within primary care.

For peer review only - http://bmjopen.bmj.com/site/about/guidelines.xhtml

Snyder et al (2010)[75]	USA	To describe the professional exchanges that occurred between community pharmacists and physicians engaged in successful collaborative working relationships (CWRs), using a published conceptual model and tool for quantifying the extent of collaboration	Semi structured interviews, and completion of the Pharmacist-Physician Collaborative Index	Five pairs of community pharmacists and physician colleagues	• n/a	• **Integration**<br>_Interprofessional collaboration_: Pharmacists were the primary initiator of these CWRs. Initial conversations were usually (but not always) conducted face-to-face and often scheduled in advance by the pharmacist.<br><br>Establishing trust was the provision of high-quality recommendations that improved patient outcomes. Both professionals commented on how seeing these positive outcomes was key to the success of their relationship.<br><br>Resistance manifested passively, as lack of physician response to recommendations, and actively, as refusal to provide patient laboratory data in spite of signed medical releases and hesitations to provide referrals for clinical services beyond patient education.
Trap et al. (2010)[104]	Ethiopia, Uganda and Zimbabwe.	To develop an indicator-based tool for systematic assessment and reporting of good pharmacy practice (GPP).	direct observations, record reviews, interviews and simulated clients in surveyed facilities		• Medicines supply<br>• Professional pharmacy service	Indicators developed focusing on:<br>• **Safety**<br>Documentation of care (5 indicators); Dispensing (14 indicators)<br>• **Access**

peer review only

						Availability of medicines (1 indicator)<br>• **Environment**<br>Appearance of the pharmacy (7 indicators); physical resources (1 indicator)<br>• **Competence**<br>Competence in the dispensing process (4 indicators), Clinical knowledge and diagnostic skills (2 indicators)
Urbonas et al. (2010)[47]	Lithuania	To analyse pharmacy specialists' attitudes toward the quality of pharmaceutical services at Lithuanian community pharmacies.	Survey	471 Lithuanian community pharmacy specialists	• Medicines supply	Survey covered two quality dimensions:<br>• **Competence**<br>Competency in the dispensing process (5 indicators covering side effects, time spent with patient, information about drug therapy and healthy lifestyle)<br>• **Person-centred care**<br>Patient experience (5 indicators covering consideration of financial capabilities, patient needs, helpfulness to each patient, willingness to get patient to come back to pharmacy)
White et al. (2010)[48]	Australia	To investigate the views of a range of stakeholders regarding the effectiveness of	In-depth interviews	20 in-depth interviews were conducted with various stakeholders, including community	• Medicines supply	• **Environment:**<br>Appearance of the pharmacy:<br>Maximizing the visibility of health ranges

For peer review only - http://bmjopen.bmj.com/site/about/guidelines.xhtml

		service quality as a differentiating position for community pharmacy.		pharmacists, managers of pharmacy groups, and industry advisers	• Professional pharmacy service	• **Person-centred care:** Patient experience: Greeting customers by name<br><br>• **Competence:** Competence in the dispensing process: sitting with customer to discuss prescription and health needs<br><br>Clinical knowledge and diagnostic skills: potential for community pharmacy to become more service orientated by offering home medication reviews, screening program and disease state management.
De Bie et al. (2011)[105]	Netherlands	To develop a national system of quality indicators for community pharmacy care, reported by community pharmacies.	Delphi	14 pharmacy practice experts and 76 practising pharmacists	• Medicines supply	Indicators focused on:<br>• **Competence** Competence in the dispensing process (7 indicators)<br><br>• **Safety** Systems for ensuring safety quality (15 indicators); compounding (7 indicators); dispensing (13 indictors)
Horvat et al. (2011)[83]	Slovenia	To identify content of pharmacy performance	Interviews + Delphi	Phase 1: interviews with 43 pharmacy users were conducted to identify	• Medicines supply	• **Person-centred care:**

For peer review only - http://bmjopen.bmj.com/site/about/guidelines.xhtml

		relevant to patient satisfaction.		patients' experiences and expectations relating to pharmacies.<br><br>Phase 2: a 10 member expert panel was employed in a two round Delphi technique to rate the importance of each item for the patient satisfaction.		Patient-pharmacist relationship (1 item); Professionalism (17 items)<br><br>• **Competence:**<br>Competence in the dispensing process (18 items)<br><br>• **Access:**<br>Waiting time (1); Opening hours (2); Availability of medicine (3); Physical access (3); availability of pharmacist (1)<br><br>• **Environment**: private consultation area (1); waiting area (2); appearance of the pharmacy (10); appearance of the pharmacy (4)
AHRQ (2012)[50]	USA	The Agency for Healthcare Research and Quality (AHRQ) funded the development of the Community Pharmacy Survey on Patient Safety Culture. This survey is designed specifically for community pharmacy providers and other staff and asks for their opinions about the	Survey	Details on development of survey not provided	Not specified	The survey includes 36 items that measure 11 composites of<br><br>• Safety culture:<br>Physical space and environment (3 items);<br>Teamwork (3 items);<br>Staff training and skills (4 items);<br>Communication openness (3 items);<br>Patient counselling (3 items);<br>Staffing, work pressure and pace (4 items);

For peer review only

		culture of patient safety in their pharmacy.				Communication about prescriptions across shifts (3 items);<br>Communication about mistakes (3 items);<br>Response to mistakes (4 items)<br>Organizational learning (3 items);<br>Overall perceptions of patient safety (3 items)
Halsall et al. (2012)[27]	UK	To develop a conceptual framework characterizing healthcare quality in the community pharmacy setting.	Focus groups	10 focus group discussions with 47 participants (patients and their carers, pharmacists and pharmacy staff, and NHS staff who commissioned pharmacy services) were conducted across the northwest of England, United Kingdom.	• Medicines supply	• **Access**<br>Availability of medicines: Patient awareness of available medicines<br>Physical access: whether patients could physically access care.<br>• **Environment**<br>Physical resources: Pharmacy personnel having access to adequate structures to provide care and that these should be continually reviewed.<br>• **Competence:**<br>Competence in the dispensing process: Supplying medicines appropriately and providing individualized advice to patients.<br>• **Person-centred care:**<br>Patient experience: Ensuring patients/carers at the point of care have a positive perception of the experience

For peer review only

Dadfar et al. (2012)[51]	Iran	To assess the quality of Tehran pharmacies' services and their impacts on the pharmaceutical firms, and suggest some improvements	Survey + in-depth interviews	127 pharmacy users completed the questionnaire.<br><br>32 interviews with Pharmacists (n=10),<br><br>Pharmaceutical managers<br><br>(n=8);<br><br>patient<br><br>(n=9)<br><br>Physician<br><br>(n=4);<br><br>MOH authority (n=1)	• Medicines supply	Survey covered the following dimensions of quality:<br><br>• **Competence:** Competence in the dispensing process (5 items)<br><br>• **Person-centred care:** Professionalism (5 items)<br><br>• **Environment:** Physical resources (1 item); appearance of the pharmacy (1 item); appearance of the pharmacy (1 item)<br><br>• **Access:** Opening hours (1 item); waiting time (1 item)
White et al. (2012)[52]	Australia	To explore the perceptions of pharmacy staff regarding the factors that constitute a high level of service quality using the service quality determinants proposed by the Conceptual Model of Service Quality	Structured interviews	27 pharmacy assistants and 6 pharmacists in 3 community pharmacies in Sydney.	Not specified	• **Safety:** All the participants acknowledged the existence of some form of internal quality control programs, but provided inconsistent answers and uncertainty regarding frequency, process, and content of such programs, within and across the pharmacies
Phipps et al. (2012)[53]	UK	To evaluate the internal reliability, factor structure and construct validity of the Pharmacy	Survey	A total of 4105 members of the community pharmacy workforce, all drawn from one of the	Not specified	24 items emerged relative to<br><br>• **Safety**

		Safety Climate Questionnaire (PSCQ) when applied to a pan-European sample of community pharmacies.		five participating countries (Denmark, Germany, the Netherlands, Portugal and Great Britain)		Safety culture: Organizational learning (13 items); Blame culture (4 items); Working conditions (4 items); Safety focus (3 items).
Rubio-Valera et al (2012)[71]	Spain	To identify and analyse factors affecting GP-CP collaboration.	Semi-structured interviews	18 GPs, 19 community pharmacists	n/a	• **Integration**<br>Communication mechanisms and information systems: 1) having a coordinator and 2) sharing a clinical chart to have access to patient information
Kelly et al. (2013)[76]	Canada	To capture the opinions of family physicians and community pharmacists in Newfoundland and Labrador (NL) regarding collaborative practice.	Survey	407 pharmacists & 462 family doctors	n/a	• **Integration**<br>Communication mechanisms and information systems: Pharmacists preferred telephone or face to-face communication over paper correspondence with GPs. GPs preferred telephone communication. Pharmacists believed that electronic transfer of information should be explored.<br><br>Interprofessional collaboration: GPs believed that the most important pharmacist functions were to help improve patient adherence and fill prescriptions. Pharmacists would like to participate more in decisions regarding identification and management of drug-related problems—managing drug interactions, providing drug

For peer review only

						information to inform decisions around patient drug therapy and assisting to modify drug therapy to resolve patient-specific problems.<br><br>Incentivisation: Lack of compensation and the need to collaborate with multiple GPs/pharmacists to provide care for patients were viewed as the 2 most significant barriers.
Patterson et al. (2013)[84]	USA	To describe and identify significant relationships among pharmacy service use, general and service-specific patient satisfaction, pharmacy patronage motives, and marketing awareness in a service-oriented, independent community pharmacy	Survey	241 patients	• Medicines supply	Survey items coverage:<br>• **Access:** Waiting time (2 items); Availability of pharmacist (1 item)<br>• **Patient-experience:** Professionalism (6 items); patient experience (2 item); Patient-pharmacist relationship (1 item)<br>• **Competence:** Competence in the dispensing process (7 items)<br>• **Environment:** Appearance of the pharmacy (1 item); private consultation area (1 item)
Merks et al. (2014)[85]	Poland	To compare factors that influence a patient's choice of pharmacy in Poland and in the UK, to identify which of them are components of pharmaceutical care, and to relate them to	Survey	417 patients from 36 pharmacies in Poland and 405 patients from 56 pharmacies in the UK.	• Not specified	• **Access**<br><br>Physical access: The convenient location of pharmacy was one of the most frequently reported factors by Polish and British respondents.<br><br>• **Person-centred care**

For peer review only

		patient loyalty to the same pharmacy				Professionalism: professional service was one of the most frequently reported factors by Polish and British respondents. British respondents were more likely than the Polish to choose a pharmacy because of a professional service.<br><br>• **Environment**<br>Private consultation area: British respondents were more likely than the Polish to choose a pharmacy because a possibility to discuss their health problems in a separate consultation room<br>Appearance of the pharmacy: Polish respondents were more likely than the British to base their choice of pharmacy on the aesthetic decoration of the pharmacy. Aesthetic decoration of the pharmacy was more important to respondents who often visited a pharmacy (one to two times a week) than to those who visited a pharmacy less frequently.<br><br>• **Competence**<br>Clinical knowledge and diagnostic skills: Good advice received in a pharmacy was one of the most frequently reported factors by the British respondents. British respondents were more likely

For peer review only

						than the Polish to choose a pharmacy because of a possibility to receive good advice.
McMilan et al. (2014)[86]	Australia	To explore the attributes of pharmacy choice for people with chronic conditions.	Semi-structured interviews	97 interviews (patients=70, carer n=8, patient/carer n=19)	• Not specified	• **Person-centred care**<br><br>Patient experience: Taking the time to ensure that the person's individual needs were met and not identifying people solely by their condition(s) were exemplars of caring pharmacy staff. The provision of information was viewed positively by participants and deemed essential when obtaining a new medication. Some consumers from culturally diverse backgrounds sought a pharmacy where a staff member spoke the same language or had the same cultural background<br><br>Patient-pharmacist relationship: Continuity of care was another reason for utilising a regular pharmacy, as this facilitated awareness of the person's medical history. For others, medication safety was a key priority for them and hence, using a regular pharmacy was seen as a way to optimise this via continuity of care.<br><br>Professionalism: Staff approachability facilitated a

For peer review only

						relaxed environment for consumers to ask questions and seek advice, thus supporting patient empowerment and resulting in continued use of that pharmacy.<br><br>• **Access**<br>Physical access: The majority of participants selected a conveniently located pharmacy, e.g. close to their home or doctor, to use regularly, in order to reduce the time accessing care.
Mehralian et al. (2014)[87]	Iran	To assess pharmacy customers' priorities and satisfaction with community pharmacy services in Tehran	Survey	800 pharmacy customers of 200 community pharmacies in 22 districts of Tehran	• Medicines supply<br><br>• Professional pharmacy service (Sale of D medicines following minor illness consultation)	Survey items looked at:<br>• **Person-centred care:** professionalism (2)<br>• **Competence:** competency in the dispensing process (3); Clinical knowledge and diagnostic skills (5)<br>• **Access:** Availability of medicines (3), waiting time (1)<br>• **Environment:** appearance of the pharmacy (1), waiting area (1), private consultation area (1)
Odukoya et al. (2014)[54]	USA	To examine factors influencing quality of patient interaction at community pharmacy	Non-participant observation (quantitative approach)	22 community pharmacies	• Medicines supply	• **Access:**<br><br>Physical access: The key enabling variables affecting amount of time pharmacists spent with

		drive-through and walk-in counselling areas				patients were location of interaction (drive-through or walk-in) and level of pharmacy busyness. Pharmacists spent less time with patients at the drive-through compared to the walk-in counselling area.
Chen et al. (2015)[88]	China	Examines the impact of service quality and the mediating effects of customer satisfaction and customer loyalty on willingness to pay more.	Survey	479 retail pharmacy users in China	• Not specified	Survey items looked at:<br>• **Environment** Appearance of the pharmacy (2 items)<br>• **Access** Physical location (2 items); Availability of medicines (3 items); Waiting times 3 items); Opening hours (1 item)<br>• **Person-centred care** Professionalism (3 items)<br>• **Safety** Documentation of care (1 item)
Hattingh et al. (2015)[55]	Australia	To explore the unique privacy and confidentiality requirements of mental health consumers and carers in the Australian community pharmacy context	In-depth interviews and focus groups	There were 98 participants consisting of consumers and carers (n = 74), health professionals (n = 13) and representatives from consumer organisations (n = 11).	• Professional pharmacy service	• **Environment:**<br>Private consultation area: Consumers and carers expressed concerns that their anonymity and right to receiving sensitive information were breached when other customers were present in the pharmacy.

For peer review only

						Due to the highly accessible nature of community pharmacy services and services being provided in a public space, there is a fear of being recognised by colleagues, friends and neighbours when collecting medication<br><br>The use of a private consultation room or area was seen as a main facilitator for overcoming privacy and confidentiality issues during pharmacy interactions.
Schoenmakers et al. (2015)[106]	Netherlands	To assess the validity of 52 quality indicators (QI) for community pharmacies using the Indicator Assessment Framework (IAF)	An expert panel applied the IAF criteria to the set of QIs collected in 1,807 Dutch community pharmacies on their performance in 2011.	Expert panel consisted of 6 pharmacists from urban as well as rural areas and from different settings, such as independent pharmacies, pharmacies in pharmacy chains, or pharmacies in health centres	• Medicines supply	Indicators focused on the following domains:<br><br>• **Competence**<br>Competence in the dispensing process (3 indicators)<br>Training of pharmacy staff (1 indicator)<br><br>• **Safety**<br>Systems for ensuring safety (21 indicators); Compounding (3 indicators); dispensing (21 indicators); documentation of care (2)<br>• **Integration** (1 indicator)

For peer review only

Alhomoud et al. (2016)[89]	UAE	Assessed patients' experiences and satisfaction with community pharmacy services in the UAE, which can be used as an indicator to improve services.	Survey	415 patients	• Medicines supply.	Questionnaire comprised of Items on:<br>• **Person-centred care:** Professionalism (4); patient experience (2)<br>• **Competence:** competency in the dispensing process (5)<br>• **Access:** Availability of pharmacy staff (1)
Arkaravichien et al. (2016)[108]	Thailand	Test a quality indicators tool for feasibility by applying it in two pharmacy settings; accredited independent community pharmacies and accredited chain community pharmacies,	Observation and interviewing pharmacist in charge	60 pharmacies enrolled in the study of which 34 were independent pharmacies and 26 chain pharmacies	• Medicines supply	The tool comprised of indicators covering:<br>• **Environment:** appearance of the pharmacy (1 indicator); appearance of the pharmacy (2 indicators); dispensary (3 indicators); physical resources (1 indicator)<br>• **Access:** availability of pharmacy staff (2 indicators), availability of medicines (7 indicators)<br>• **Competence:** Competence in the dispensing process (18 indicators)
Grey et al. (2016)[103]	UK	To ask key stakeholders to confirm, and rank the importance of, a set of characteristics of good pharmaceutical service provision	Delphi	22 participants (DPs, CPs and patients/lay member)	• Medicines supply<br>• Professional pharmacy service	A set of 23 characteristics for providing good pharmaceutical services in CPs and DPs was developed:<br>• **Safety:** medicine supply (6)<br>• **Person-centred care**: patient experience (6)<br>• **Environment**: Appearance of the pharmacy (1); waiting area (1)

						• **Competence:** Clinical knowledge and diagnostic skills (2)<br>• **Integration:** Interprofessional collaboration (3)
Hashemian et al. (2016)[79]	Iran	To investigate the collaborative working relationship between pharmacists and GPs in terms of their attitudes, role perceptions, experience with collaborative practice, preferred method of communication, areas of current and further collaboration, and perceived barriers to interprofessional collaboration in a sample of the Iranian population	Survey	132 pharmacists and 99 general practitioners	• n/a	• **Integration**<br>Communication mechanisms and information systems: The preferred method of communication for collaborative practice for both groups was by telephone or face to face rather than by letter.<br>Interprofessional collaboration: Both groups reported that the most frequent collaboration is to "manage drug interactions", "provide patient counselling" and "manage side-effects of medications". Both groups were willing to collaborate in decision making on patients' pharmacotherapy and management of drug interactions.<br><br>Information sharing and access: Neither group was overly concerned about "liability for shared information", "lack of compensation" or "dealing with multiple care professionals" as barriers to collaboration

For peer review only

Nilugal et al. (2016)[90]	Malaysia	To investigate patient's attitudes, and satisfaction towards community pharmacist's role in Selangor, Malaysia	Survey	180 patients at three different community pharmacies in three different regions of Selangor state	• Medicines supply.<br>• Professional pharmacy service	Questionnaire items covered the following:<br>• **Competence:** Competency in dispensing process (7); clinical knowledge and diagnostic skills (7)<br>• **Access:** waiting time (3)<br>• **Person-centred care:** patient experience (2); professionalism (2)
Shiyanbola et al. (2016)[56]	USA	To describe older adults' perception of a quality pharmacy including their expectations of a quality pharmacy and their preferences in a quality pharmacy.	Focus groups	Six focus groups (60 patients) held in community centres and senior residence facilities in Wisconsin	• Medicines supply	• **Access:** Opening hours; availability of pharmacy staff; physical access<br>• **Person-centred care:** Patient-pharmacist relationship: Interpersonal relationship with pharmacist/pharmacy staff; familiarity with pharmacy/pharmacist staff;<br>Professionalism: friendliness and helpfulness of staff; pharmacist courtesy<br>• **Competence:** Competence in the dispensing process: ensuring medication safety; facilitates medication adherence; readily available to clarify questions

For peer review only

Teichert et al. (2016)[107]	Netherlands	To present a comprehensive quality indicator set for community pharmacies and to report the scores for these indicators as supplied by the majority of Dutch community pharmacies	Community pharmacists in the Netherlands were invited in 2013 to provide information for the set of 2012.Quality indicators were mapped by categories relevant for pharmaceutical care and defined for structures, processes and dispensing outcomes	Information was provided by 1739 of the 1981 Dutch community pharmacies (88 %)	• Medicines supply	Indicators focused on the following domains:<br>• **Competence**<br>Competence in the dispensing process (27 indicators); Training of pharmacy staff (5 indicators)<br>• **Safety**<br>Systems for ensuring safety quality (24 indicators); Compounding (4 indicators); medicine supply (3 indicators); documentation of care (1 indicator)<br>• **Integration** (3 indicators)
Weiss et al (2016)[57]	UK	To investigate the similarities and differences in how pharmaceutical services are provided by community pharmacies (CPs) and dispensing doctor practices (DPs) and (b) to identify the issues relevant to determining the quality of pharmaceutical services in these settings.	Mixed methods: A postal questionnaire of DPs and CPs. A subsection<br>of questionnaire respondent sites were selected<br>d to take part in case studies, which involved<br>documentary analyses, observation and staff interviews	Questionnaire: 52 CPs, 31 DPs<br>There were three CP and four DP case study sites, with 17 staff interviews	• Medicines supply	• **Person-centred care:**<br>Patient experience - providers' underlying values and commitment to providing patient-centred care. At the supermarket pharmacy, for example, staff would always strive to fulfil a patient's needs as they saw this as not only good for business but also their duty as a service provider.<br>• **Safety**<br>Medicine supply: Effective systems of work in relation to the checking of prescribed items.<br>Systems for ensuring safety: Effective systems of work in

						relation to the way in which dispensing errors were managed.
Koster et al. (2016)[58]	Netherlands	To provide insight into the agreement about quality of pharmaceutical care, measured both by a patient questionnaire and video observations	Pharmaceutical encounters in four pharmacies were video-recorded. Patients completed a questionnaire based upon the Consumer Quality Index. An observation protocol was used to code the recorded encounters. Agreement between video observation and patients' experiences was calculated.	109 encounters were included for analysis	• Medicines supply	• **Competence:** Competence in the dispensing process: Information provision (3 items); Medication counselling (4 items)<br>• **Person-centred care:** Professionalism: Pharmacy staff's communication style (8 items)
Feehan et al. (2017)[91]	USA	To gauge patient preferences explicitly for primary healthcare services that could be delivered through community pharmacy set-tings in the USA	Questionnaire (Discrete Choice Experiment)	10006 adults who had to have a minimal repeat use of a pharmacy for health care needs—defined as filled at least three or more prescriptions for	• Medicines supply.<br>• Professional pharmacy service	Attributes covered:<br>• **Access** Opening hours: Hours of operation Availability of medicines: Prescription ordering, availability and information

For peer review only - http://bmjopen.bmj.com/site/about/guidelines.xhtml

				themselves, at a pharmacy in the past 12months		Waiting time: Service logistics (I.e. walk in vs appointment)<br>• **Integration:** pharmacy has access to and can enter prescriptions and health information into your (the patient's) electronic medical record<br>• **Competence:**<br>Clinical and diagnostic skills: Physical examinations; Diagnostic testing; Preventive services; prescribing; Medication review services
Júnior et al. (2017)[110]	Brazil	To characterize the profiles and activities of community pharmacists, as well as the quality indicators of private community pharmacies in Paraná State - Brazil	Survey	533 pharmacists in Paraná State - Brazil	• Medicines supply<br>• Professional pharmacy service	• Five indicators relative to **environment:** Waiting area (1); Private consultation area (1); Physical resources (3).
Löffler et al. (2017)[78]	Germany	Investigating pharmacists' and general practitioners' views on barriers to interprofessional collaboration in the German health care system.	Interviews and focus groups	Six pharmacists were interviewed and four pharmacists took part in the focus group discussion.<br><br>Seven GPs were interviewed and eight	• n/a	• **Integration**<br>Communication mechanisms and information systems: The majority of pharmacists stated to encounter recurring difficulties getting GPs on the phone and receiving an answer to their query.<br>Interprofessional collaboration: GPs felt challenged in means of treating patients under time

				GPs participated in the focus group discussions.		constraints and avoiding or limiting polypharmacy. Most physicians perceived that community pharmacists were not able to respond to this challenge. GPs felt that pharmacists don't have background information on patients' medical history and/or professional knowledge to understand and reconstruct physicians' reasoning in many cases.<br>Proximity: Pharmacists employed in rural and provincial regions often experienced long-lasting working relationships to local GPs that were mostly characterized by mutual trust and appreciation. In contrast, in cities interprofessional collaboration was constrained by urban anonymity: Quite often, pharmacists hardly knew the GPs they tried to contact.<br>Incentivisation: Some GPs thought that pharmacists would have their own agenda trying to profit from patients with long-term conditions
Aziz et al. (2018)[92]	Pakistan	To assess pharmacies services with regard to patient's need	Survey	1088 patients of 544 community pharmacies	• Medicines supply.	41 Items on satisfaction covering the following dimensions:

						• **Access:** Physical access (3); opening hours (2); availability of medicines (3); waiting time (1)<br>• **Environment:** appearance of the pharmacy (1), waiting area (1), private consultation area (2); appearance of the pharmacy (1)<br>• **Person-centred care:** professionalism (2); patient experience (1)<br>• **Competence:** competency in the dispensing process (19)
Jacobs et al. (2018)[15]	UK	To explore stakeholder perceptions of the organisational and extra-organisational factors associated with service quality and quantity in community pharmacy as an established exemplar of private sector organisations providing publicly-funded healthcare.	Semi-structured interviews	Forty semi-structured interviews were conducted with service commissioners, superintendent and front-line pharmacists, purposively selected from across nine geographical areas and a range of community pharmacy organisational types in England.	• Medicines supply<br>• Professional pharmacy service	• **Competence:**<br>Competence in the dispensing process: For dispensing, speed and accuracy were the most commonly cited elements of service quality. However, for pharmacists themselves, and for many service commissioners, accuracy was paramount.<br><br>Clinical knowledge and diagnostic skills: clinical aspects were considered by a number of pharmacists and commissioners to be an important element of quality either through counselling or the clinical check.<br>• **Integration**

For peer review only

						<u>Interprofessional collaboration:</u> Positive relationships between community pharmacies and local GP surgeries were seen to help nurture interdisciplinary practice, foster closer working around patients, increase effective signposting and improving communication.
Newlands et al. (2018)[59]	UK	To systematically identify and prioritise community pharmacy services in Scotland which required improvement and/or guideline development	A modified nominal group technique (NGT) was used for topic generation followed by an electronic Delphi survey	Stakeholder group comprising community pharmacists, policy makers, lay and pharmacy organisation representatives.	• Medicines supply<br>• Professional pharmacy service	Consensus reached on guideline development for:<br>• **Competence:** <u>Clinical knowledge and diagnostic skills:</u> promoting the appropriate sale and supply of over-the-counter medicines; promotion and delivery of a Minor Ailment Scheme.<br><br><u>Competence in the dispensing process:</u> Patient counselling for prescribed medication; evidence-based strategies to promote medication adherence; enhancing medication use for vulnerable patients (including high risk, sheltered housing residents, immigrants, homeless)<br>•

For peer review only

Tran et al. (2018)[99]	Vietnam	To determine the pattern of pharmacy customers' viewpoints regarding their satisfaction with the quality of services of community pharmacies in Vietnam	Q-methodology	144 pharmacy customers from 40 pharmacies in four Vietnamese cities.	• Medicines supply.	Statements covered the following dimensions:<br>• **Competence:** Competency in the dispensing process (22)<br>• **Environment:** Appearance of the pharmacy (1); waiting area (1); private consultation area (1); appearance of the pharmacy (1); dispensing (1)<br>• **Access:** opening hours (3); availability of medicines (2); physical access (1); availability of pharmacist (1)<br>• **Person-centred care:** professionalism (3)
Watson et al. (2018)[29]	UK	Exploration of service providers' attitudes and beliefs of quality and quality improvement in the community pharmacy setting in the UK.	Semi-structured interviews and focus groups	42 service providers. Four focus group discussions were undertaken with 38 pharmacists/pharmacy support staff and semi-structured interviews with four key informants from pharmacy organisations across the UK	• Medicines supply<br>• Professional pharmacy service(Supply of OTC medicines following minor illness consultation)	Quality was described in terms of:<br>• **Person-centred care:** Professionalism: showing empathy Patient-pharmacist relationship: developing rapport. The issue of good continuity of staff was also identified as being associated with better quality because it was believed to be important for developing trust and rapport between patients and pharmacy personnel.<br>• **Competence** Clinical knowledge and diagnostic skills: eliciting specific information during consultations; providing the right

						information/advice; prompt resolution of symptoms<br>• **Environment**<br>Private consultation area: The physical environment of pharmacies such as having a counter was also identified as a potential barrier to asking questions.
Fujita et al. (2019)[60]	Japan	To establish the quality dimensions of home pharmaceutical care (HPC) from the perspectives of home healthcare professionals	Semi-structured interviews and focus groups	Semi-structured interviews and focus groups were carried out with nine home healthcare teams, comprising 61 multidisciplinary professionals including pharmacists, doctors, nurses, care managers, home helpers, medical social workers and other relevant stakeholders involved in home healthcare.	• Professional pharmacy service	• **Environment:** physical resources<br>• **Competence:**<br>*Clinical knowledge and diagnostic skills*: pharmacist factors (professionalism, effectiveness, experience); during home pharmaceutical care (provision of medication review; frequency of visiting home; time spent at home)<br>Impact on patients (humanistic outcomes; clinical outcomes, economic outcomes); impact on other healthcare professional (task shifting, operational efficiency); recognition of benefits of home pharmaceutical care.<br>• **Integration:**

For peer review only

						Communication mechanisms and information systems: Before home pharmaceutical care (attendance at meetings; collaborative visiting schedule arrangements); after home pharmaceutical care (information sharing, timeliness)
Guhl et al (2019)[61]	Germany	Examines the value created by community pharmacies-defined as perceived customer value-in the prescription drug market through varying elements of service quality.	Survey	289 pharmacy users	• Medicines supply	Dimensions covered in the survey:<br>• **Environment:** physical resources (1 item); appearance of the pharmacy (2 items), cleanliness & hygiene (1 item)<br>• **Person-centred care:** professionalism (4 items);<br>• **Access:** Waiting time (1 item); availability of medicines (2 items), opening hours (1 item); physical access (3 items)<br>• **Competence:** Competence in the dispensing process (4 items)<br>• **Safety:** medicine supply (1 item)
Halit et al. (2019)[115]	Lebanon	developing good pharmacy practice(GPP) guidelines to be applied by community pharmacists for	In January 2018, the OPL Scientific Committee decided to elaborate GPP guidelines for		• Medicines supply<br>• Professional pharmacy service (sale of	The GPP standards comprised of sections that addressed the following dimensions:<br>• **Environment:**

For peer review only

		services' quality improvement	community pharmacists and created the Community Pharmacy Practice Subcommittee, which was in charge of this project. To create the Lebanese GPP guidelines, the committee relied on the guidelines already implemented in several countries and tailored them to the Lebanese situation		OTC medicines following minor illness consultation)	Appearance of the pharmacy; appearance of the pharmacy; Private consultation area; dispensary.<br><br>Physical resources (availability of a refrigerator and other equipment, equipment status, routine maintenance, Availability of drug information systems; availability of medical devices and complementary medicines)<br><br>• **Access**<br>Availability of medicines: purchasing, storage, and maintenance of quality<br><br>• **Safety:**<br>Compounding; operating procedures, documentation, and raw material handling.<br>Documentation of care: Documentation systems (patient medication profile, formulary systems, policies and standard operating procedures, documentation of interventions)<br><br>• **Competence:**<br>Competence in the dispensing process:<br>Provision of medicines (prescription availability, patient identification, and dispensing).

For peer review only

						Interaction and communication (communication skills of pharmacist and staff, provision of advice, promotion of good health, and provision of written information)<br><br>Clinical knowledge and diagnostic skills:<br>Supply of OTC medicines (advice on selection and use, responding to minor ailments)<br><br>Health promotion (engagement in health promotion, participation in health promotion campaigns)<br><br>Diagnostics (provision of diagnostic tests, documentation of tests done)<br><br>Training of pharmacy staff:<br>Research and professional development (participation in research projects, participation in continuing education)<br>Trainees (acceptance of trainees, monitoring and documentation, activity description)<br><br>• **Integration:**<br>Interprofessional collaboration: Development of pharmaceutical care plans, patient monitoring, identification of medication-

For peer review only

						related problems, interaction with other prescribers, and other healthcare professionals
Hindi et al. (2019)[75]	UK	To examine the views of patients, pharmacists and GPs on how community pharmacies are currently used and to identify how community pharmacy services may be better integrated within the primary care pathway for people with long-term conditions	Focus groups	Two focus groups were conducted with respiratory patients (n=6, 5) and two with type 2 diabetes patients (both= 5). Two focus groups were held with pharmacists (n=7, 5) and two with GPs (both n = 5).	• n/a	• **Integration** Communication mechanisms and information systems: All stakeholder groups believed pharmacists required more access to patient information (i.e. medical records) to have a better overall understanding of patients' conditions.
Hindi et al. (2019)[93]	UK	Identify factors that could influence patients to make better use of community pharmacies within the primary care pathway	Survey	289 Patients with asthma or chronic obstructive pulmonary disease registered at two GP practices.	• Medicines supply<br>• Professional pharmacy service	Questionnaire Items looked at factors influencing patient's likelihood to use community pharmacy services:<br>• **Person-centred care:** Professionalism (3); patient-pharmacist relationship (2)<br>• **Environment:** private consultation area (3)<br>• **Access:** physical access (1)<br>• **Integration:** communication mechanisms and information systems – information sharing (6); proximity (2)
Mirzaei et al (2019)[62]	Australia	To build a theory-grounded model of service quality in	Stage 1 dealt with item generation using theory,		• Medicines supply	Dimensions of service quality covered:

		community pharmacies and to create a valid survey instrument to measure consumers' perceptions of service quality.	prior research and qualitative interviews with pharmacy consumers. Selected items were then subjected to content validity and face validity. Stages 2 and 3 included psychometric testing among English-speaking adult consumers of Australian pharmacies. Exploratory factor analysis was used for item reduction and to explain the domains of SQ			• **Person-centred care:** Relationship: Trusting relationship (Relationship) Professionalism: Friendliness/Helpfulness<br>• **Access:** Availability of the pharmacist, waiting time<br>• **Competence:** Competence in the dispensing process: advice, expertise, effectiveness/knowledge, patient health outcome Clinical knowledge and diagnostic skills: services such as blood pressure testing or diabetes support services were minimally discussed and of least importance to patients<br>• **Environment**: physical resources, appearance of the pharmacy; appearance of the pharmacy.
Patterson et al. (2019)[101]	USA	To measure the relative strength of patient preferences for community pharmacy attributes and to describe associations between patient sociodemographic and health characteristics	Questionnaire (Discrete Choice Experiment)	773 American adults (≥18 years) who had filled a prescription at a pharmacy, other than a mail-order pharmacy, within the last 12 months.	• Medicines supply	Quality dimensions covered:<br>• **Access**: opening hours (1)<br>• **Person-centred care:** professionalism (1); patient-pharmacist relationship (1).<br>• **Competence:** Competence in the dispensing process (2)

For peer review only

		and pharmacy preferences				
Watson et al (2019)[28]	UK	Explored citizens' perspectives about the quality of community pharmacy services in the UK and whether and how the quality of community pharmacy services should be measured.	Semi-structured interviews and focus groups	20 individuals participated (Scotland (n=7) all interviewed individually; England (one focus group (n=4) and four individual interviews); and Wales (one focus group (n=5))	• Medicines supply<br>• Professional pharmacy service (supply of OTC medicines following minor illness consultation)	• **Person-centred care:** Patient experience: Friendly caring service, Patient pharmacist relationship: continuity of care, and staff knowing the individual. Professionalism: Professional approach in customer appearance, including behaviour and appearance<br>• **Environment:** Private consultation area: Physical characteristics of the pharmacy in supporting privacy, with either a separate consultation room or dedicated private area, and the need to have confidential conversations with the pharmacist.
Abu Hagar et al (2020)[63]	UAE	To evaluate the present status of risk<br><br>occurrence in community pharmacies in Abu Dhabi and investigate the protective plans that are followed in risky<br><br>cases to generate an overall view of risk management plans	Survey	322 licensed community pharmacists in<br><br>Abu Dhabi	• Medicines supply	• **Environment** Private consultation area: Provide a private consultation area. Appearance of the pharmacy: temperature should be maintained to avoid discomfort. Appearance of the pharmacy: Sufficient and well distributed lights across<br>• **Safety**

For peer review only

		in concurrent pharmacy practice.				Systems for ensuring safety: Keep patient data in separate filing; do not share patient information; discard documents containing patients' data in a proper way<br><br>• **Competence**<br>Clinical knowledge and diagnostic skills: The most reported reason for adverse drug reactions was lack of knowledge about side effects (cannot recognize ADR cases to report them)<br><br>• **Integration**<br>The most reported protective measure to avoid medication errors was contacting the GP
Al-Jumaili et al. (2020)[95]	Iraq	To evaluate patient satisfaction with community pharmacy services and measure the relationships between patient satisfaction and pharmacy/pharmacist characteristics and patient quality of life	Survey	400 patients at 20 community pharmacies in 10 different geographical areas in Baghdad city	• Medicines supply.	The questionnaire included:<br>• **Access:** Availability of pharmacist (1)<br>• **Person-centred care:** professionalism (2), patient-pharmacist relationship (1); patient experience (3)<br>• **Competence:** competence in the dispensing process (11)<br>• **Environment:** appearance of the pharmacy (1); private consultation area (1)
Aizpurua-Arruti et al (2020)[66]	Spain	Confirm if the elderly people who go to the pharmacies still think	Focus group	10 elderly people in San Sebastian	• Medicines supply	• **Person-centred care**

		that the commitments that define the Friendly Pharmacy are the ones previously identified				Patient experience: Patients valued patient experience based on trust and intimacy<br><br>• **Environment**<br>Private consultation area: Participants highlight the positive aspects of accessible spaces with personalised service areas<br>Waiting area: Participants highlight the positive aspects seating areas that facilitate the wait.<br><br>• **Competence**<br>Competence in the dispensing process: Patients valued advice on use of medicines<br><br>• **Integration:**<br>Communication mechanisms and information systems: Patients valued good communication with other health care settings; and referral to health and social care.
Badro et al. (2020)[117]	Lebanon	To assess good pharmacy practice (GPP) aspects and compare GPP scores among community pharmacies in Lebanon, using a tool developed jointly by the International Pharmaceutical	Survey which included 109 questions	A team of 10 licensed inspectors who work at the Lebanese Order of Pharmacists and visited 276 community<br><br>pharmacies across Lebanon	• Medicines supply<br>• Professional pharmacy service	The questionnaire was adapted to the Lebanese context and included questions on:<br><br>• **Safety:** Documentation of care (25); Systems for ensuring safety (5)<br>• **Competence**: competence in the dispensing process (51); clinical

For peer review only

		Federation (FIP) and the World Health Organization (WHO) to improve and maintain standards of pharmacy practice				knowledge and diagnostic skills (19)<br>• **Environment:** appearance of the pharmacy (1); private consultation area (1)<br>• **Access:** availability of medicines (5); physical resources (3); availability of pharmacy staff (1); appearance of the pharmacy (7)
Bratkowska et al. (2020)[94]	Poland	To evaluate patient satisfaction with services provided in independent pharmacies and pharmacy chains in Poland	Survey	117 patients randomly selected from four community pharmacies in Poland (2 chain pharmacies and 2 independent pharmacies)	• Medicines supply	items divided into the following:<br>• **Access:** Waiting time (1); availability of medicines (2)<br>• **Environment:** Appearance of the pharmacy (1); private consultation area (1)<br>• **Competence:** Competency in the dispensing process (6)<br>• **Person-centred care:** Patient-pharmacist relationship (1); patient experience (2); professionalism (1)<br>•
Goto et al (2020)[65]	Japan	To examine how a patient's continuity with the same pharmacist and pharmacy is associated with their evaluation of the quality of pharmacy services.	Questionnaire	3,492 Patients who regularly visited pharmacies	• Medicines supply<br>• Professional pharmacy service	Survey items covered the following dimensions:<br>• **Person-centred care:**<br>Patient experience (3 items); patient-pharmacist relationship (1 item); professionalism (2 items)

						• **Competence:**<br>Competence in the dispensing process (1 item); Clinical knowledge and diagnostic skills (1 item)<br>• **Access:**<br>Availability of pharmacy staff (1 item), availability of medicines (1 item)
Jacobs et al (2020)[26]	UK	To investigate organisational factors associated with variation in safety climate, patient satisfaction and self-reported medicines adherence in English community pharmacies.	Multivariable regressions were conducted using data from two cross-sectional surveys (1. PSCQ and 2. patient satisfaction with visit)	277 pharmacists and 971 patients visiting 39 pharmacies, across 9 diverse geographical areas.	• Medicines supply<br>• Professional pharmacy service	• **Safety:** Safety climate was associated with organisational culture.
Sato et al (2020)[64]	Japan	In Japan, a new system called Health Support Pharmacy (HSP) was introduced in 2016, to promote responsible self-medication with non-prescription medicines and increase awareness of public health activities provided through community pharmacies.	Semi-structured interviews	Twenty-four community pharmacists from across Japan.	• Professional pharmacy service	• **Pharmacy environment:** physical resources<br>• **Competence:**<br>Clinical knowledge and diagnostic skills: professional expertise (effectiveness, professionalism, teamwork, scope and duration of expertise).

For peer review only

		This study aimed to identify factors that can impact on the quality of HSP services provided by community pharmacists in Japan.				Provision of community health education and other events (health promotion techniques, event planning).<br><br>Impact on individuals and the general public (economic outcomes; clinical outcomes humanistic outcomes; health behaviour chance, pharmacy as a community hub); impact on other professional (reassurance and operation efficiency)<br><br>• **Integration**<br>Interprofessional collaboration: interactions between the community and the pharmacy; collaboration with other professionals
Tran et al. (2020)[100]	Vietnam	To understand elderly pharmacy users' satisfaction on the community pharmacy services in Ho Chi Minh City, Vietnam.	Q- methodology	32 pharmacy users, aged over 60, was recruited in four pharmacies in Ho Chi Minh City, Vietnam,	• Medicines supply.	Statements divided into the dimensions of quality:<br><br>• **Competence:** Competency in the dispensing process (22)<br>• **Environment:** Appearance of the pharmacy (1); waiting area (1); private consultation area (1); appearance of the pharmacy (1); dispensing (1)<br>• **Access:** opening hours (3); availability of medicines (2);

For peer review only

						physical access (1); availability of pharmacist (1)<br>• **Person-centred care:** professionalism (3)
Waltering et al. (2020)[111]	Germany	To develop indicators for assessing the quality of medication review in public pharmacies	Delphi	The expert group in the Delphi survey consisted of 22 participants. These were pharmacists, representatives of the health insurance companies, scientific Staff members of various institutes and one member each from an association of panel doctors and a chamber of pharmacists	• Professional pharmacy service	After two rounds of Delphi, a final set of indicators consisting of<br>• **Safety:** Documentation of care (1); Systems for ensuring safety (1)<br>• **Competence:** clinical knowledge and diagnostic skills (4)
Watson et al (2020)[30]	UK	To conceptualise GPs' perceptions and beliefs about the quality of community pharmacy services in general and, more specifically, using the concept of 'always events' and the management of acute consultations.	Semi-structured interviews	20 GPs (Scotland n=12, England n= 8)	• Medicines supply<br>• Professional pharmacy service (sale of OTC medicines following minor illness consultation)	• **Access**<br>Physical access: GPs suggested that pharmacies should be accessible and near to the population that they serve<br>Opening hours: Pharmacies should have extended opening hours for the convenience of patients and known to GPs.<br>Availability of medicines: Most GPs said that pharmacies should hold an adequate, well managed stock of medication (and alternatives) and other medical devices or be able to obtain them quickly.

For peer review only - http://bmjopen.bmj.com/site/about/guidelines.xhtml

						• **Person-centred care:** Patient experience: GPs suggested that pharmacists should offer personalised care, with good listening and communication skills and all staff should have a positive orientation to patients/customers. They should involve people in decisions, treat them with sincerity, and always check their understanding of treatment; they should be kind but not "paternalistic".<br><br>• **Competence:** Clinical knowledge and diagnostic skills: pharmacists should identity problems relating to drug efficacy, side effects and compliance.<br><br>• **Environment:** Private consultation area: have at least on dedicated room, or at least a spate/private area, and to take a proactive approach in offering it to patients/customers.<br><br>• **Integration:** Communication mechanisms and information systems: There were mixed views around the recording and sharing patient information.

For peer review only

Abebe et al. (2021)[69]	USA	To characterize documentations in community pharmacies and to examine factors that contribute to perceived documentation of care quality.	Survey	445 community pharmacists in Wisconsin	• Medicines supply	• **Safety**<br>Documentation of care: 20<br>Survey items covered: Handover procedures; frequency of handovers; Handover training; Handover outcomes; Technology; Handover resolution
Clabaugh et al (2021)[67]	USA	To determine pharmacists' perceptions of working conditions while controlling for respondent (years of experience, degree, work status) and workplace variables (prescription volume, type of community setting).	Survey	1222 pharmacists<br>48 of 50 states	Not specified	Qualitative analysis of survey identified features relative to<br>• **Safety:** Supervision, work design, quality emphasis, group behaviour.<br>• **Person-centred care:** patient expectations.
Thang et al. (2021)[96]	Vietnam	To identify factors that affect the overall satisfaction of customers visiting community pharmacies in Vietnam.	Survey	354 patients at 13 randomly selected community pharmacies in five districts in Hanoi, Vietnam.	• Medicines supply.<br>• Professional pharmacy service (sale of OTC medicines following minor illness consultation)	Questionnaire items covering:<br>• **Person-centred care:** professionalism (2); personalised care (1)<br>• **Competence:** competence in the dispensing process (6); clinical knowledge and diagnostic skills (2)<br>• **Access:** physical location (2); opening hours (1); availability of medicines (1)

For peer review only

						• **Environment:** appearance of the pharmacy (1); appearance of the pharmacy and hygiene (1); private consultation area (1)
Fernandes et al. (2021)[113]	Brazil	To develop an instrument to evaluate the quality of services provided in community pharmacies, as well as to test its application through a geographic information system for the visualization of the results	After a review of the scientific literature, a set of quality indicators was submitted to expert analysis. From the final constructed matrix, observational and self-administered questionnaires were elaborated and applied in pharmacies belonging to a city of the South eastern region of Brazil.		• Medicines supply	Items covered 5 dimensions of quality:<br>• **Access:** opening hours; physical access<br>• **Environment:** Physical resources; dispensary; private consultation area; appearance of the pharmacy<br>• **Competence:** Competency in the dispensing process<br>• **Safety:** Systems for ensuring safety; dispensing
Loo et al (2021)[68]	UK	Explored the content of online feedback provided by patients from across the UK in relation to their experiences of their interaction with pharmacy staff and pharmacy services	Patient stories published on Care Opinion, a national online patient feedback platform, for a one-year period were searched for all content relating to patients'	237 patient stories	• Medicines supply<br>• Professional pharmacy service	Online feedback provided by patients mapped on the following dimensions of quality:<br>• **Person-centred care:** Made up the highest proportion of patient feedback relating to community pharmacy with most feedback being positive. Helpfulness, professionalism, kindness, friendliness, politeness were

For peer review only - http://bmjopen.bmj.com/site/about/guidelines.xhtml

			pharmacy experiences.			common terms used to describe pharmacy staff across all settings.<br><br>• **Competence:** Patient feedback was overall positive regarding the services that pharmacies offer such as healthcare advice, clinical services (e.g. community pharmacy blood pressure checks, minor ailment services) as well as ordering repeat prescriptions and delivery services.<br><br>• **Safety:** Medication errors were only mentioned in community pharmacy related stories whereby medications were mistakenly dispensed.<br><br>• **Access:** Patient stories often related to the accessibility of the pharmacy. Overall, accessibility was positively (28/40, 70%) described by patients. Several stories related to the convenience of community pharmacies attached to or within GP surgeries. Opening times of community pharmacies were appreciated in a small number of stories.<br><br>• **Environment:** A minority of patient feedback related to the environment of the pharmacy and these were mainly negative.

For peer review only

						Patients complained of crowding within some community pharmacies
Mohamud et al. (2021)[97]	Sudan	To explore patients' satisfaction level with pharmacist's communication, consulting and service delivery qualities.	Survey	385 patients from 229 community pharmacies in Khartoum	• Medicines supply.	Questionnaire items covered:<br>• **Person-centred care:** patient experience (3); professionalism (3)<br>• **Access:** waiting time (1); availability of pharmacy staff (1)<br>• **Competence:** competency in the dispensing process (6)<br>• **Environment:** private consultation area (1)
Nneoma et al. (2021)[112]	Nigeria	To develop quality indicators for assessing pharmaceutical care performance in the Nigerian community pharmacies.	Delphi	A panel of 10 pharmacy experts in Nigeria	• Medicines supply	Indicators categorised under the following dimensions:<br>• **Safety:** Systems for ensuring safety (9); Documentation of care (4); medicine supply (3)<br>• **Competence:** Competency in the dispensing process (5); Training of pharmacy staff (2)
Sepp et al. (2021)[118]	Estonia	To identify the implementation of the Community Pharmacy	Questionnaire	The cross-sectional electronic surveys were conducted among community pharmacies	• Medicines supply	Items covered 4 dimensions of quality:<br>• **Safety:** Documentation of care

For peer review only

		Services Quality Guidelines (CPSQG) as a profession-driven initiative towards improving and harmonizing community pharmacy services in Estonia.		in Estonia in 2014 (N = 478 pharmacies), 2016 (N = 493), and 2019 (N = 494)	• Professional pharmacy service	• **Competence:** Competency in the dispensing process ; clinical and diagnostic skills<br>• **Environment**: Private consultation area indicators; waiting area
Wongvedvanij et al (2022)[70]	Thailand	To investigate how patients perceive different dimensions of service quality, especially for non-prescription medicines during the COVID-19 outbreak.	Survey	378 Thai patients during the spread of COVID-19.	• Professional pharmacy service (sale of OTC medicines following minor illness consultation)	Survey items covered 4 domains of quality:<br>• **Competence**: Clinical knowledge and diagnostic skills (6 items)<br>• **Access:** opening hours (1 item)<br>• **Safety:** documentation of care (2 items)<br>• **Person-centred care**: Patient experience (7 items); professionalism (6 items)
Schomer et al (2022)[71]	USA	This study applied a human factors and ergonomics approach to describe community-based pharmacy personnel perspectives regarding how work environment characteristics affect the ability to perform the duties necessary for optimal patient care and how contributors to	Survey	4606 pharmacists and pharmacy technicians	Not specified	There were 12 items developed for the survey that focused on **safety**

For peer review only

		stress affect the ability to ensure patient safety.				
Wongvedvanij et al (2022)[72]	Thailand	To explore pharmacists' and patients' perception of potential pharmacy service quality for dispensing non-prescription medicines.	Semi-structured interviews	14 pharmacists and 20 patients.	• Professional pharmacy service (sale of OTC medicines following minor illness consultation)	• **Competence:**<br>Clinical knowledge and diagnostic skills:<br>ability, skills, knowledge, expertise to diagnose patient symptoms before dispensing non-prescription medicines, pharmacist must obtain accurate personal information using different communication channels<br><br>• **Person-centred care:**<br>Patient-pharmacist relationship: pharmacists having ongoing interactions and developing personal relationships with their patients over a period of time. This relationship encompasses mutual trust, loyalty respect and knowledge.<br>Patient experience: Pharmacists pay attention to individual patients and treat them based on their personal health conditions and requirements<br><br>• **Environment:**<br>Cleanliness & hygiene: clean and hygienic physical space and equipment used to handle pharmaceutical products

For peer review only

Kummer et al (2022)[73]	Serbia	To examine patients' perceptions of an incident that occurred in community pharmacies using CIT and determine recommendations for improving the quality of pharmacy services.	Interviews	20 patients from 3 community pharmacies in Serbia	• Medicines supply	A total of 68 critical incidents were collected and divided into two groups: positive (37) and negative (31), depending on patients' satisfaction/dissatisfaction with community pharmacy services. Critical incidents covered:<br>• **Access:** opening hours, availability of pharmacy staff, physical access, waiting time; availability of medicines<br>• **Competence:** Competence in the dispensing process<br>• **Person-centred care**: professionalism; patient experience<br>• **Safety:** Compounding
Parinyarux et al. (2022)[98]	Thailand	to explore the satisfaction of the community pharmacy users with the facilities and services received from drugstores under the GPP standards and examine the impact of satisfaction toward each GPP domain on overall satisfaction (OS) and the intention to receive the pharmacy services as the first choice in the	Survey	388 community pharmacy users	• Medicines supply.<br>• Professional pharmacy service	Questionnaire items categorised under the following dimensions:<br>• **Safety:** Systems for ensuring safety (2); medicine supply (4)<br>• **Environment:** dispensary (3); private consultation area (1); physical resources (2)<br>• **Competence:** competence in the dispensing process (4); clinical knowledge and diagnostic skills (1)

For peer review only

		case of common and non-serious illnesses (IntR).				• **Person-centred care:** professionalism (4)
Sepp et al (2022)[74]	Estonia	To evaluate to what extent the patient-centred care (PCC) principles are included in the Community Pharmacy Services Quality Guidelines (CPSQG) in Estonia	Deductive content analysis was performed using the PCC framework developed by Santana et al.		• Medicines supply<br>• Professional pharmacy service	• **Access**. This included availability of medicines and more broadly access to diagnostic testing such as Blood pressure blood sugar measurement<br>• **Person-centred care:** Cultivating communication; respectful and compassionate care; engaging patients and managing their care; integration of care

**studies are ordered chronologically*

For peer review only

Supplementary File 3: Critical Appraisal

Critical appraisal of qualitative studies using the JBI checklist for qualitative research

- No, Unclear = 0
- Yes = 1

	**1** *Philosophical perspective*	**2.** *Research question*	**3.** *Methods*	**4.** *Data analysis*	**5.** *Results*	**6.** Researcher orientation	**7.** *Researcher influence*	**8.** *Participants*	**9.** *Ethics*	**10.** *Conclusions*	**Total score (max: 10)**
Aizpurua-Arruti et al (2020)[66]	N	Y	Y	N	N	N	N	Y	N	N	3
White et al. (2010)[48]	Y	Y	Y	Y	N	N	N	N	N	N	4
Scahill et al. (2010)[46]	Y	Y	Y	Y	Y	N	N	N	N	Y	6
Löffler et al. (2017)[78]	Y	Y	Y	Y	N	N	N	N	Y	Y	6
Loo et al (2021)[68]	Y	N	N	Y	Y	N	N	Y	Y	Y	6
Shiyanbola et al. (2016)[56]	Y	Y	Y	Y	Y	N	N	N	Y	Y	7

For peer review only

Watson et al (2019)[28]	Y	Y	Y	Y	Y	N	N	N	Y	Y	7
Kummer et al (2022)[73]	N	Y	Y	Y	Y	N	N	Y	Y	Y	7
Rapport et al. (2009)[45]	Y	Y	Y	Y	Y	Y	N	Y	N	Y	8
Halsall et al. (2012)[27]	Y	Y	Y	Y	Y	N	N	Y	Y	Y	8
White et al. (2012)[52]	Y	Y	Y	Y	Y	N	N	Y	Y	Y	8
Jacobs et al. (2018)[15]	Y	Y	Y	Y	Y	N	N	Y	Y	Y	8
Fujita et al. (2019)[60]	Y	Y	Y	Y	Y	N	N	Y	Y	Y	8
Sato et al (2020)[64]	Y	Y	Y	Y	Y	N	N	Y	Y	Y	8
Wongvedvanij et al (2022)[72]	Y	Y	Y	Y	Y	N	N	Y	Y	Y	8
Rubio-Valera et al (2012)[71].	Y	Y	Y	N	Y	Y	Y	Y	Y	Y	9

For peer review only

McMilan et al. (2014)[86]	Y	Y	Y	Y	Y	Y	N	Y	Y	Y	9
Hattingh et al. (2015)[55]	Y	Y	Y	Y	Y	N	Y	Y	Y	Y	9
Watson et al. (2018)[29]	Y	Y	Y	Y	Y	Y	N	Y	Y	Y	9
Hindi et al. (2019)[75]	Y	Y	Y	Y	Y	Y	Y	Y	Y	Y	10
Watson et al (2020)[30]	Y	Y	Y	Y	Y	Y	Y	Y	Y	Y	10
**Mean**	**0.9**	**0.9**	**0.9**	**0.9**	**0.9**	**0.3**	**0.2**	**0.8**	**0.8**	**0.9**	**7.5**

Description of criteria

1. *Congruity between the stated philosophical perspective and the research methodology.*
2. *Congruity between the research methodology and the research question or objectives.*
3. *Congruity between the research methodology and the methods used to collect data.*
4. *Congruity between the research methodology and the representation and analysis of data.*
5. *There is congruence between the research methodology and the interpretation of results.*
6. *Locating the researcher culturally or theoretically.*
7. *Influence of the researcher on the research, and vice-versa, is addressed.*
8. *Representation of participants and their voices.*
9. *Ethical approval by an appropriate body.*
10. *Relationship of conclusions to analysis, or interpretation of the data.*

For peer review only

Critical appraisal of quantitative studies using the JBI checklist for analytical cross-sectional studies

- No, Unclear = 0
- Yes = 1

	1. Sample	2. Subjects and setting	3. Exposure	4. Standard criteria	5. Confounding	6. Cofounding effects	7. Outcomes	8. statistical analysis	Total score (max: 8)
Harding et al. (2010)[114]	N	Y	N	N	N	N	N	Y	2
Al-Jumaili et al. (2020)[95]	Y	N	N	N	N	N	N	Y	2
Wongvedvanij et al (2022)[70]	N	N	N	N	N	N	Y	Y	2
Júnior et al. (2017)[110]	Y	Y	N	N	N	N	N	N	2
Vilako et al. (2007)[81]	Y	Y	N	N	N	N	N	Y	3
Hashemian et al. (2016)[79]	N	N	Y	Y	N	N	Y	N	3
Bratkowska et al. (2020)[94]	Y	Y	N	N	N	N	N	Y	3
Arkaravichien et al. (2016)[108]	Y	N	N	N	N	N	Y	Y	3

For peer review only

Kelly et al. (2013)[76]	Y	Y	N	N	N	N	Y	Y	4
Odukoya et al. (2014)[54]	N	Y	N	N	Y	Y	N	Y	4
Chen et al. (2015)[88]	N	Y	Y	N	Y	Y	N	N	4
Alhomoud et al. (2016)[89]	Y	Y	Y	Y	N	N	N	N	4
Parinyarux et al. (2022)[98]	N	N	N	N	Y	Y	Y	Y	4
Badro et al. (2020)[117]	Y	Y	N	N	Y	Y	N	N	4
Urbonas et al. (2010)[47]	N	Y	Y	Y	N	N	Y	Y	5
Schomer et al (2022)[71]	Y	Y	Y	Y	N	N	Y	N	5
Sepp et al. (2021)[118]	Y	N	Y	Y	N	N	Y	Y	5
Nilugal et al. (2016)[90]	Y	Y	Y	Y	N	N	Y	N	5
Hindi et al. (2019)[93]	Y	Y	N	N	Y	Y	N	Y	5
Jacobs et al 2020	Y	Y	N	N	Y	Y	N	Y	5

For peer review only

Clabaugh et al (2021)[67]	Y	Y	N	N	Y	Y	N	Y	5
Thang et al. (2021)[96]	Y	Y	N	N	Y	Y	Y	N	5
Feehan et al. (2017)[91]	Y	Y	Y	Y	N	N	N	Y	5
Phipps et al. (2012)[53]	Y	Y	Y	Y	N	N	Y	Y	6
Worley (2006)[44]	Y	Y	Y	Y	N	N	Y	Y	6
Feletto et al. (2010)[49]	Y	Y	Y	Y	N	N	Y	Y	6
Patterson et al. (2013)[84]	Y	Y	Y	Y	N	N	Y	Y	6
Aziz et al. (2018)[92]	Y	Y	Y	Y	N	N	Y	Y	6
Abu Hagar et al (2020)[63]	Y	Y	Y	Y	N	N	Y	Y	6
Goto et al (2020)[65]	Y	Y	Y	Y	N	N	Y	Y	6
Mohamud et al. (2021)[97]	Y	Y	Y	Y	N	N	Y	Y	6
Patterson et al. (2019)[101]	Y	N	Y	Y	Y	Y	N	Y	6

For peer review only

Koster et al	Y	Y	Y	Y	N	N	Y	Y	6
Sakurai et al. (2009)[82]	Y	Y	Y	Y	Y	N	Y	Y	7
Mehralian et al. (2014)[87]	N	Y	Y	Y	Y	Y	Y	Y	7
Abebe et al. (2021)[69]	Y	Y	Y	Y	Y	Y	N	Y	7
Merks et al. (2014)[85]	Y	Y	Y	Y	Y	Y	Y	Y	8
Guhl et al (2019)[61]	Y	Y	Y	Y	Y	Y	Y	Y	8
**Mean**	**0.8**	**0.8**	**0.6**	**0.6**	**0.4**	**0.3**	**0.6**	**0.8**	**4.9**

For peer review only

Description of criteria

1. Were the criteria for inclusion in the sample clearly defined?
2. Were the study subjects and the setting described in detail?
3. Was the exposure measured in a valid and reliable way?
4. Were objective, standard criteria used for measurement of the condition?
5. Were confounding factors identified?
6. Were strategies to deal with confounding factors stated?
7. Were the outcomes measured in a valid and reliable way?
8. Was appropriate statistical analysis used?

Critical appraisal of Delphi studies using the Conducting and REporting of DElphi Studies (CREDES) checklist

- No, Unclear = 0
- Yes = 1
- N.A = Not counted in total score

	**1.** Rationale	**2.** Expert panel	**3.** Methods	**4.** Procedure	**5.** Consensus	**6.** Results	**7.** Discussion	**8.** Conclusions	**9.** Dissemination	**Total score (max: 9)**
Nneoma et al. (2021)[112]	Y	N	Y	N	Y	N	N	Y	N	4
Horvat et al. (2011)[83]	Y	Y	N	N	Y	Y	N	N	Y	5
Fernandes et al. (2021)[113]	Y	N	Y	N	N	N	Y	Y	Y	5
Grey et al. (2016)[103]	Y	N	N	N	Y	Y	Y	Y	Y	6
Puumalainen et al. (2005)[102]	Y	N	Y	N	Y	Y	Y	Y	Y	7
Waltering et al. (2020)[111]	Y	N	N	Y	Y	Y	Y	Y	Y	7
James et al. (2008)[103]	Y	Y	Y	Y	Y	Y	Y	Y	N	8
Newlands et al. (2018)[59]	Y	Y	Y	N	Y	Y	Y	Y	Y	8
De Bie et al. (2011)[105]	Y	Y	Y	Y	Y	Y	Y	Y	Y	9
**Mean**	1	0.4	0.7	0.3	0.9	0.8	0.8	0.9	0.8	**6.6**

For peer review only

Description of criteria:

1. *Purpose and rationale: The purpose of the study should be clearly defined and demonstrate the appropriateness of the use of the Delphi technique as a method to achieve the research aim. A rationale for the choice of the Delphi technique as the most suitable method needs to be provided.*
2. *Expert Panel: Criteria for the selection of experts and transparent information on recruitment of the expert panel, sociodemographic details including information on expertise regarding the topic in question, (non)response and response rates over the ongoing iterations should be reported.*
3. *Description and methods: The methods employed need to be comprehensible; this includes information on preparatory steps (How was available evidence on the topic in question synthesised?), piloting of material and survey instruments, design of the survey instrument(s), the number and design of survey rounds, methods of data analysis, processing and synthesis of experts' responses to inform the subsequent survey round and methodological decisions taken by the research team throughout the process.*
4. *Procedure: Flow chart to illustrate the stages of the Delphi process, including a preparatory phase, the actual 'Delphi rounds', interim steps of data processing and analysis, and concluding steps*
5. *Definition and attainment of consensus: It needs to be comprehensible to the reader how consensus was achieved throughout the process, including strategies to deal with non-consensus.*
6. *Results: Reporting of results for each round separately is highly advisable in order to make the evolving of consensus over the rounds transparent. This includes figures showing the average group response, changes between rounds, as well as any modifications of the survey instrument such as deletion, addition or modification of survey items based on previous rounds.*
7. *Discussion and limitation: Reporting should include a critical reflection of potential limitations and their impact of the resulting guidance.*
8. *Adequacy of conclusions: The conclusions should adequately reflect the outcomes of the Delphi study with a view to the scope and applicability of the resulting practice guidance.*

9. *Publication and dissemination: The resulting guidance on good practice should be clearly identifiable from the publication, including recommendations for transfer into practice and implementation.*

Critical appraisal of mixed methods studies using the Mixed Methods Appraisal Tool (MATT)

- No, Can't tell [CT] = 0
- Yes = 1

	**Screening**		**Qualitative part**				**Quantitative part**					**Mixed Methods Part**						
**Study**	**1**	**2.**	**3.**	**4.**	**5.**	**6.**	**7.**	**8.**	**9.**	**10.**	**11.**	**12.**	**13.**	**14**	**15**	**16.**	**17.**	**Total score (Max: 17)**
Dadfar et al. (2012)[51]	Y	Y	N	Y	N	N	Y	CT	N	Y	CT	Y	Y	N	N	Y	N	8
Snyder et al (2010)[75]	Y	Y	Y	Y	Y	Y	N	N	Y	N	N	Y	Y	N	N	Y	N	10
Weiss et al (2016)[57]	Y	Y	Y	Y	Y	Y	Y	Y	Y	Y	Y	Y	Y	Y	Y	Y	Y	17
Mirzaei et al (2019)[62]	Y	Y	Y	Y	Y	Y	Y	Y	Y	Y	Y	Y	Y	Y	Y	Y	Y	17
**Mean**	1	1	0.75	1	0.75	0.75	0.75	0.5	0.75	0.75	0.5	1	1	0.5	0.5	1	0.5	**13**

Screening questions

1. Are there clear research questions?

2. Do the collected data allow to address the research questions?

Qualitative questions

For peer review only

3. Is the qualitative approach appropriate to answer the research question?
4. Are the qualitative data collection methods adequate to address the research question?
5. Are the findings adequately derived from the data?
6. Is the interpretation of results sufficiently substantiated by data?
7. Is there coherence between qualitative data sources, collection, analysis and interpretation?

<u>Quantitative questions</u>

8. Is the sampling strategy relevant to address the research question?
9. Is the sample representative of the target population?
10. Are the measurements appropriate?
11. Is the risk of nonresponse bias low?
12. Is the statistical analysis appropriate to answer the research question?

<u>Mixed Methods questions</u>

13. Is there an adequate rationale for using a mixed methods design to address the research question?
14. Are the different components of the study effectively integrated to answer the research question?
15. Are the outputs of the integration of qualitative and quantitative components adequately interpreted?
16. Are divergences and inconsistencies between quantitative and qualitative results adequately addressed?
17. Do the different components of the study adhere to the quality criteria of each tradition of the methods involved?

For peer review only

Supplementary file 4: Input from PPI group summarised under the dimensions/sub-dimensions of quality framework

Dimensions	Input from PPI Group	Incorporation of PPI input into framework dimensions
**Patient Experience**	Professionalism:<br>- Mannerisms of staff in some pharmacies is poor.<br>- Anyone who has any interaction with customers' needs to be polite.<br>- Staff do not have IDs which makes it hard to distinguish between staff.<br>Patient experience:<br>- Patients need to be put at the forefront of decision making – where is the patient voice?	Professionalism:<br>- *Pharmacy staff being distinguishable by wearing a name badge with their role.*<br>- *Mannerisms of pharmacy staff*<br>*Patient Experience:*<br>- *Involving patients in decision making*
**Integration**	- Integration is a huge element of quality. No sense that GPs/pharmacists speak to each other. GPs direct patients to pharmacies but pharmacies seem to be unaware. Pharmacies then direct patients back to GPs.<br>.	Communication mechanisms and information systems*:*<br>- *Bi-directional communication between CPs and other providers.*
**Access**	Waiting times<br>- Services have gone downhill at community pharmacy. It takes longer to get a prescription.<br>Availability of medicines<br>- Difficult to encourage public to go to pharmacy for advice if they can't even get medications<br>- Free prescription is not always available<br>Availability of pharmacy staff<br>- Huge tensions around single pharmacists having to do everything but not accessible to patients.	Waiting times<br>- *Importance of waiting time for receiving prescriptions*<br>Availability of medicines<br>- *Availability of medicines and offering alternatives.*<br>Availability of pharmacy staff<br>- *Availability of pharmacy staff.*<br>- *Having adequate numbers and appropriately qualified pharmacy staff*

For peer review only

	- Training received by pharmacy staff questionable.<br>- There needs to be more training of pharmacy staff and this needs to be funded.	
**Environment**	<u>Appearance of the pharmacy</u><br>Unlike GP or dentist, going to community pharmacy is like going to a shop. Not a healthcare environment.<br><br><u>Private consultation area</u><br>- Privacy issues – asking details such as address, DOB in front of people.<br>- Providing room in pharmacies for consultations is a good thing	<u>Private consultation area</u><br>- *The importance of privacy and having a consultation area.*

For peer review only

Supplementary File 5: Summary of key dimensions identified in this review

Dimension	Type*
**Access**	
• **Opening hours:** availability of pharmacy services during stated opening hours and extended opening hours	**S**
• **Waiting time:** minimising wait times for healthcare services	**P**
• **Availability of pharmacy staff:** *Having adequate numbers and appropriately qualified pharmacy staff for the community pharmacy to operate*	**S**
• **Physical access:** *parking space near the pharmacy; accessibility for people with special needs (e.g. visually impaired, people with baby carriages); geographical proximity and location (e.g. ease of access of community pharmacies via public transportation; work/home; other healthcare facilities)*	**S**
• **Availability of medicines:** *maintaining an adequate, well managed stock of essential medicines as well as other medical devices. Pharmacists providing patients with information about alternative medicines and their prices.*	**S**
**Environment**	
• **Appearance of the pharmacy:** *pharmacy appearing health service orientated by clearly displaying medicines and informational materials. Pharmacy having sufficient counters for dispensing medicines and adequate physical space for pharmacy staff to provide health promotion, education, consultation or screening services to individuals or groups.* Cleanliness & hygiene *of the pharmacy maintained to promote a good impression of the pharmacy.*	**S**
• **Waiting area:** *Ensuring that the waiting area has sufficient space and seating. Informing patients of waiting times and the reasons for any delays.*	**S**
• **Dispensary:** *Well organised and spacious designed to ensure efficient processing of prescriptions. Storage shelves clearly labelled with drug classifications and medicines are kept according to the drug classifications*	**S**
• **Physical resources:** *having drug information systems and resources to ensure provision of additional healthcare services.*	**S**
• **Private consultation area:** *Having a sufficiently sized dedicated area for consultations in the pharmacy and proactively offering it to patients.*	**S**
**Competence**	
• **Competence in the dispensing process:** *Accuracy of dispensing; appropriate advice on medication (usage, storage etc.) and non-medication (diet, exercise) aspects; gathering essential patient information as part of the dispensing process.*	**P**
• **Clinical knowledge and diagnostic skills:** *Having knowledge in disease areas and diagnostic skills to assess patients and provide effective treatment. To be able to assess and refer patients to a GP or other health care provider if necessary.*	**P**

For peer review only

**Person-centred care**	
• **Patient experience:** *understanding individual needs, involving patient in decision around medications, tailoring delivery of services to people with special needs or minority groups. Involving patients in decision making*	**P**
• **Patient-pharmacist relationship** *built on trust, friendliness/helpfulness, continuity of care, and availability of the pharmacist.*	**P**
• **Professionalism:** *pharmacy staff treating patients with respect, showing empathy, expressing honest opinions, regarding patient benefit as top priority. Pharmacy staff being distinguishable by wearing a name badge with their role.*	**P**
**Safety**	
• **Compounding:** *Availability of standard operating procedures to ensure accuracy in compounding.*	**S**
• **Dispensing:** *having clear standard operating procedure for checking prescriptions; dispensing medications (particularly high-risk medications); availability of protocols and guidelines for asking patients about potential drug contraindications and drug-drug interactions. Having structured safety protocols for OTC consultations, including safeguarding.*	**S**
• **Systems for ensuring safety:** *having a quality and safety management system in place for: registering errors made during dispensing; handling near-misses and dispensing errors; evaluating patient experiences and recording the number of patient complaints.*	**S**
• **Documentation of care:** Accurate *recording of relevant information such as medical history and use of medication in a way that can be read and interpreted by other healthcare professionals. Ensuring patient personal information is stored in a confidential manner and discarded properly. Ensuring exchange of information, responsibility, and accountability when a pharmacist concludes a shift, and another replaces this outgoing pharmacist at the beginning of a new shift within the same pharmacy*	**P**
**Integration**	
• **Interprofessional collaboration**: *Establishing an active relationship between community pharmacy and wider healthcare team based on a shared understanding of competences, roles and responsibilities.*	**P**
• **Communication mechanisms and information systems:** having predefined and clear ways to communicate with other healthcare providers. Having shared communication systems between community pharmacy and the rest of the healthcare system. Ensuring bi-directional communication with other healthcare providers	**P**

**S= structure, P= process*

For peer review only

PRISMA 2020 Checklist

Section and Topic	Item #	Checklist item	Location where item is reported
**TITLE**			
Title	1	Identify the report as a systematic review.	p.1
**ABSTRACT**			
Abstract	2	See the PRISMA 2020 for Abstracts checklist.	p.2
**INTRODUCTION**			
Rationale	3	Describe the rationale for the review in the context of existing knowledge.	p.3-5
Objectives	4	Provide an explicit statement of the objective(s) or question(s) the review addresses.	p.5
**METHODS**			
Eligibility criteria	5	Specify the inclusion and exclusion criteria for the review and how studies were grouped for the syntheses.	Table 2
Information sources	6	Specify all databases, registers, websites, organisations, reference lists and other sources searched or consulted to identify studies. Specify the date when each source was last searched or consulted.	p.5, table 1
Search strategy	7	Present the full search strategies for all databases, registers and websites, including any filters and limits used.	Suppl. 1
Selection process	8	Specify the methods used to decide whether a study met the inclusion criteria of the review, including how many reviewers screened each record and each report retrieved, whether they worked independently, and if applicable, details of automation tools used in the process.	p.6
Data collection process	9	Specify the methods used to collect data from reports, including how many reviewers collected data from each report, whether they worked independently, any processes for obtaining or confirming data from study investigators, and if applicable, details of automation tools used in the process.	p.6
Data items	10a	List and define all outcomes for which data were sought. Specify whether all results that were compatible with each outcome domain in each study were sought (e.g. for all measures, time points, analyses), and if not, the methods used to decide which results to collect.	N/A
	10b	List and define all other variables for which data were sought (e.g. participant and intervention characteristics, funding sources). Describe any assumptions made about any missing or unclear information.	N/A
Study risk of bias assessment	11	Specify the methods used to assess risk of bias in the included studies, including details of the tool(s) used, how many reviewers assessed each study and whether they worked independently, and if applicable, details of automation tools used in the process.	N/A
Effect measures	12	Specify for each outcome the effect measure(s) (e.g. risk ratio, mean difference) used in the synthesis or presentation of results.	N/A
Synthesis methods	13a	Describe the processes used to decide which studies were eligible for each synthesis (e.g. tabulating the study intervention characteristics and comparing against the planned groups for each synthesis (item #5)).	Suppl. 3
	13b	Describe any methods required to prepare the data for presentation or synthesis, such as handling of missing summary statistics, or data conversions.	p.7
	13c	Describe any methods used to tabulate or visually display results of individual studies and syntheses.	p.7
	13d	Describe any methods used to synthesize results and provide a rationale for the choice(s). If meta-analysis was performed, describe the model(s), method(s) to identify the presence and extent of statistical heterogeneity, and software package(s) used.	p.7
	13e	Describe any methods used to explore possible causes of heterogeneity among study results (e.g. subgroup analysis, meta-regression).	N/A
	13f	Describe any sensitivity analyses conducted to assess robustness of the synthesized results.	N/A
Reporting bias assessment	14	Describe any methods used to assess risk of bias due to missing results in a synthesis (arising from reporting biases).	N/A
Certainty	15	Describe any methods used to assess certainty (or confidence) in the body of evidence for an outcome.	N/A

For peer review only
For peer review only - http://bmjopen.bmj.com/site/about/guidelines.xhtml

PRISMA 2020 Checklist

Section and Topic	Item #	Checklist item	Location where item is reported
assessment			
**RESULTS**			
Study selection	16a	Describe the results of the search and selection process, from the number of records identified in the search to the number of studies included in the review, ideally using a flow diagram.	Fig. 1
	16b	Cite studies that might appear to meet the inclusion criteria, but which were excluded, and explain why they were excluded.	Fig. 1
Study characteristics	17	Cite each included study and present its characteristics.	p.8-9
Risk of bias in studies	18	Present assessments of risk of bias for each included study.	N/A
Results of individual studies	19	For all outcomes, present, for each study: (a) summary statistics for each group (where appropriate) and (b) an effect estimate and its precision (e.g. confidence/credible interval), ideally using structured tables or plots.	N/A
Results of syntheses	20a	For each synthesis, briefly summarise the characteristics and risk of bias among contributing studies.	Suppl. 3
	20b	Present results of all statistical syntheses conducted. If meta-analysis was done, present for each the summary estimate and its precision (e.g. confidence/credible interval) and measures of statistical heterogeneity. If comparing groups, describe the direction of the effect.	N/A
	20c	Present results of all investigations of possible causes of heterogeneity among study results.	N/A
	20d	Present results of all sensitivity analyses conducted to assess the robustness of the synthesized results.	N/A
Reporting biases	21	Present assessments of risk of bias due to missing results (arising from reporting biases) for each synthesis assessed.	N/A
Certainty of evidence	22	Present assessments of certainty (or confidence) in the body of evidence for each outcome assessed.	N/A
**DISCUSSION**			
Discussion	23a	Provide a general interpretation of the results in the context of other evidence.	p.17-18
	23b	Discuss any limitations of the evidence included in the review.	p.19
	23c	Discuss any limitations of the review processes used.	p.19
	23d	Discuss implications of the results for practice, policy, and future research.	p.17-19
**OTHER INFORMATION**			
Registration and protocol	24a	Provide registration information for the review, including register name and registration number, or state that the review was not registered.	p.20
	24b	Indicate where the review protocol can be accessed, or state that a protocol was not prepared.	p.20
	24c	Describe and explain any amendments to information provided at registration or in the protocol.	N/A
Support	25	Describe sources of financial or non-financial support for the review, and the role of the funders or sponsors in the review.	p.19
Competing interests	26	Declare any competing interests of review authors.	p.19
Availability of data, code and other materials	27	Report which of the following are publicly available and where they can be found: template data collection forms; data extracted from included studies; data used for all analyses; analytic code; any other materials used in the review.	p.20

For peer review only - http://bmjopen.bmj.com/site/about/guidelines.xhtml

From: Page MJ, McKenzie JE, Bossuyt PM, Boutron I, Hoffmann TC, Mulrow CD, et al. The PRISMA 2020 statement: an updated guideline for reporting systematic reviews. BMJ 2021;372:n71. doi: 10.1136/bmj.n71

For peer review only

PRISMA 2020 Checklist

For more information, visit: http://www.prisma-statement.org/

For peer review only
